# Effect of the vaginal live biotherapeutic LACTIN-V (*Lactobacillus crispatus* CTV-05) on vaginal microbiota and genital tract inflammation among women at high risk of HIV acquisition in South Africa: a phase 2, randomised, placebo-controlled trial

**DOI:** 10.1016/j.lanmic.2024.101037

**Published:** 2025-04-04

**Authors:** Anke Hemmerling, Caroline M Mitchell, Suuba Demby, Musie Ghebremichael, Joseph Elsherbini, Jiawu Xu, Nondumiso Xulu, Johnathan Shih, Krista Dong, Vaneshree Govender, Vanessa Pillay, Nasreen Ismail, Gardenia Casillas, Jayajothi Moodley, Agnes Bergerat, Tess Brunner, Lenine Liebenberg, Sinaye Ngcapu, Ian Mbano, Laurel Lagenaur, Thomas P Parks, Thumbi Ndung’u, Douglas S Kwon, Craig R Cohen

**Affiliations:** Department of Obstetrics, Gynecology & Reproductive Sciences, University of California San Francisco, San Francisco, CA, USA (A Hemmerling MD PhD, G Casillas BA, Prof C R Cohen MD MPH); Department of Obstetrics & Gynecology, Massachusetts General Hospital, Boston, MA, USA (C M Mitchell MD MPH, A Bergerat PhD); Harvard Medical School, Boston, MA, USA (C M Mitchell, K Dong MD, D S Kwon MD PhD); Ragon Institute of Mass General, MIT, and Harvard, Cambridge, MA, USA (S Demby BA, M Ghebremichael PhD, J Elsherbini PhD, J Xu PhD, J Shih BSc, K Dong, T Brunner BA, Prof T Ndung’u PhD, D S Kwon); HIV Pathogenesis Programme, The Doris Duke Medical Research Institute, University of KwaZulu-Natal, Durban, South Africa (N Xulu MMedSc, N Ismail MMed, I Mbano PhD, Prof T Ndung’u); FRESH Clinic South Africa, Umlazi, South Africa (K Dong, V Pillay BNAP); Aurum Institute, Johannesburg, South Africa (V Govender MBBCh, J Moodley BPHARM MPH); Centre for the AIDS Programme of Research in South Africa, Durban, South Africa (L Liebenberg PhD, S Ngcapu PhD); Center for Epidemic Response and Innovation, Stellenbosch University, Stellenbosch, South Africa (L Liebenberg); School of Laboratory Medicine and Medical Sciences, University of KwaZulu-Natal, Durban, South Africa (L Liebenberg); Osel, Mountain View, CA, USA (L Lagenaur PhD, T P Parks PhD)

## Abstract

**Background:**

Absence of vaginal lactobacilli and accompanying genital inflammation is associated with HIV acquisition. We aimed to assess how a vaginal live biotherapeutic containing *Lactobacillus crispatus* affects cervicovaginal microbiota and markers of HIV susceptibility in South African women.

**Methods:**

This randomised, placebo-controlled, phase 2 trial evaluated LACTIN-V (*L crispatus* CTV-05), a vaginal live biotherapeutic, compared with placebo in cisgender women in South Africa, aged 18–23 years, recruited at a community-based research clinic. Eligible participants with a Nugent score of 4–10 (indicating intermediate vaginal microbiota or bacterial vaginosis) completed 7 days of oral metronidazole and were randomly assigned (2:1) to LACTIN-V (2 × 10^9^ colony forming units per dose) or placebo (the substrate alone) via an independently generated randomisation sequence. Pharmacists, participants, and investigators were masked to treatment assignment. The study product (or placebo) was dosed daily for 5 days in week 1, then twice per week for an additional 3 weeks. Adverse events were evaluated 4 weeks and 8 weeks after starting the study product. Vaginal swabs (for 16S rRNA sequencing of the vaginal microbiome) and cervicovaginal lavage (for Luminex analysis of immune markers) were collected before metronidazole treatment, before study product (or placebo) administration, and at the week 4 and week 8 follow-up visits. An endocervical cytobrush for flow cytometry analysis of immune cell populations (including CD3^+^CD4^+^ T cells, and presence of CCR5 and the activation markers CD38 or HLA-DR) was collected before study product use and at 4 weeks and 8 weeks after study product use. The coprimary outcomes for the trial were (1) safety and acceptability of LACTIN-V, as measured by number of adverse events and a validated questionnaire; (2) presence of a *Lactobacillus*-dominant vaginal microbial community by 16S rRNA gene sequencing at week 4 and week 8; and (3) comparison of change in genital tract inflammatory markers from before metronidazole treatment to week 4 and week 8 between groups. Safety analyses were done in the intention-to-treat population and efficacy analyses in a modified intent-to-treat population (ie, excluding one person assigned placebo who erroneously received LACTIN-V). This trial is completed and registered on ClinicalTrials.gov (NCT05022212).

**Findings:**

45 Black South African women were randomly assigned to receive LACTIN-V (n=32) or placebo (n=13). One woman in each group discontinued the trial during the intervention and two women discontinued during the follow-up. No severe or serious adverse events were observed. Solicited adverse events occurred in 35 (78%) of 45 participants with no significant difference by group (risk ratio 1·17, 95% CI 0·79–1·75; p=0·44). All local solicited adverse events were mild. 32 (71%) of 45 participants strongly agreed or agreed they would use the product again. *L crispatus* dominant microbiomes were identified in 13 (41%) of 32 participants in the LACTIN-V group at week 4 and eight (26%) of 31 at week 8, compared with none at week 4 and one (9%) of 11 in week 8 in the placebo group (week 4 p=0·0088; week 8 p=0·40). The proportion of activated endocervical HIV target cells out of total T cells increased from after metronidazole treatment to week 4 in the placebo group (median log_2_ fold change 1·891, IQR 1·731–4·018) but not in the LACTIN-V group (1·062, 0·449–1·424; p=0·016). Changes in the concentrations of 13 immune markers from before metronidazole treatment to week 4 or week 8 were not significantly different by group.

**Interpretation:**

The use of LACTIN-V after metronidazole significantly increased vaginal *L crispatus* colonisation during 4 weeks of use, although this increase was transient, and women in the placebo group had an increase in endocervical CD4^+^ HIV target cells during recovery compared with the LACTIN-V group. These results show that vaginal colonisation with an *L crispatus* live biotherapeutic is possible in an African context, and that optimisation of this strategy might be a way to decrease risk for HIV.

**Funding:**

US National Institute of Child Health and Human Development and US National Institute of Allergy and Infectious Diseases.

## Introduction

Dominance of the vaginal microbial community by *Lactobacillus* species is associated with reduced risk for HIV acquisition, human papilloma virus persistence, and preterm birth.^1–3^ A low *Lactobacillus* spp microbial community (<50% relative abundance) is present in a quarter of women worldwide and up to half of women in sub-Saharan Africa.^4^ Bacterial vaginosis is a clinical syndrome characterised by a high relative abundance of multiple types of anaerobic bacteria and low relative abundance of lactobacilli, and it is associated with prevalent HIV in women^5^ and incident HIV in women and their male partners.^6,7^ These highly diverse vaginal bacterial communities are associated with elevated amounts of inflammatory cytokines and chemokines in vaginal secretions and increased numbers of activated endocervical CD4^+^ T cells, thought to be the first cells to be infected by HIV after mucosal exposure.^1,8^

Antibiotic treatment of bacterial vaginosis reduces the abundance of bacterial species associated with this syndrome, but the post-treatment vaginal microbial community is most commonly dominated by *Lactobacillus iners*, a pattern associated with a high rate of transition back to bacterial communities that are similar to those in bacterial vaginosis.^9,10^ Colonisation with more beneficial *Lactobacillus* species, such as *Lactobacillus crispatus*, is believed to be necessary to maintain a low-diversity, *Lactobacillus*-dominant community and decrease risk for bacterial vaginosis and its associated sequelae.^11^ A randomised, controlled, phase 2b trial in the USA of a vaginal live biotherapeutic product, LACTIN-V (*L crispatus* CTV-05), showed colonisation with the live biotherapeutic product in up to 84% of participants during dosing and a reduction in recurrent bacterial vaginosis compared with placebo 3 months after stopping treatment with LACTIN-V.^12^

Subsequent subset analyses from the phase 2b LACTIN-V trial showed a reduction in the proinflammatory cytokine IL-1α and the epithelial disruption marker soluble E-cadherin in the LACTIN-V group compared with placebo.^13^ However, previous studies have not examined the effect on genital immune cells. We aimed to test the hypothesis that use of LACTIN-V by young women at high risk of HIV infection in South Africa would both increase vaginal abundance of *L crispatus* and decrease genital tract inflammation associated with increased HIV acquisition.

## Methods

### Study design and participants

From May 11, 2021, to April 18, 2023, we conducted a single-site, randomised, double-blind, placebo-controlled, phase 2 trial to evaluate LACTIN-V in a clinic in Umlazi, South Africa (study site activated May 4, 2021, study posted to ClinicalTrials.gov on May 7, and first patient screened on May 11). The protocol was approved by institutional review boards at the University of KwaZulu Natal (160/19), the University of California San Francisco (19–27732), Massachusetts General Hospital (2020P002237), and by the South African Health Products Regulatory Authority (20190519). The trial protocol and statistical analysis plan are available in the appendix (pp 24–271).

Cisgender women aged 18–23 years who were enrolled in the Females Rising through Education, Support and Health (FRESH)^14^ cohort in Umlazi, South Africa, were screened for eligibility through testing from a regular FRESH clinic visit. HIV incidence rates in FRESH participants are more than 8 per 100 person-years, despite extensive education regarding HIV transmission, free access to condoms, and HIV pre-exposure prophylaxis.^14^ Initial screening consisted of Nugent score; nucleic acid amplification testing for the sexually transmitted infections *Neisseria gonorrhoeae*, *Chlamydia trachomatis*, *Trichomonas vaginalis*, and *Mycoplasma genitalium* done by a local diagnostic laboratory; and a urine specimen to test for urinary tract infection and pregnancy. The Nugent score counts bacterial morphologies on a Gram-stained slide of vaginal fluid; 0–3 is normal, 4–6 is intermediate, and 7–10 is bacterial vaginosis. FRESH participants who were not pregnant, were HIV-uninfected, had a Nugent score of 4–10, and had negative results for bacterial sexually transmitted infections were invited to enrol in the LACTIN-V study. The full list of inclusion and exclusion criteria is provided in the appendix (pp 14–17). Eligible participants provided written informed consent.

### Randomisation and masking

Women were randomly assigned 2:1 to receive vaginal applicators prefilled with LACTIN-V or matching placebo. The randomisation sequence was prepared for a sample size of 60, with an additional six participants available in case of participant withdrawal, by a statistician not affiliated with the trial with randtreat in Stata, version 17. Participants, study personnel, and laboratory staff analysing samples were masked to treatment allocation. The list of randomised treatment assignments was provided to RenaClinical (now Eramol UK; Sevenoaks, UK), who labelled applicator pouches and cartons with the randomisation number and shipped them to the study site. Trial personnel enrolling the participants were unaware of group assignments. At the time of randomisation, the masked site pharmacist selected the next identical-appearing box of study product in sequential order and distributed to masked study personnel.

### Procedures

At enrolment, participants completed questionnaires about demographic and health information. Eligible women were given a 7-day course of metronidazole (Kharwastan Pharmaceuticals and Allied Wholesalers, Durban, South Africa) of 400 mg twice daily within 30 days of screening. Those who completed this course of metronidazole were randomly assigned and were given the study product (or placebo) to use within 8–48 h of their last metronidazole dose. The applicators contained LACTIN-V at 2 × 10^9^ colony forming units (CFU) per dose or matching placebo, both supplied by Osel (List Biological Laboratories, Campbell, CA, USA). The placebo formulation contained the same inactive ingredients as LACTIN-V (ie, trehalose, xylitol, sodium ascorbate, colloidal silicon dioxide, and maltodextrin), without *L crispatus* CTV-05. Participants were instructed on how to insert self-administered, prefilled applicators in the vagina. During week 1, the study product was administered daily for 5 days (2 × 10^9^ CFU/dose), and then two doses per week during weeks 2–4. Except for the second, third, and fifth dose, which were administered at home during week 1 and were stored at room temperature after being dispensed, all doses were stored in the clinic pharmacy at 5° C (range 2–8) and self-administered at the clinic during participants’ twice per week visits. Staff were available to assist if needed and could confirm adherence to dosing by checking that the medication had been dispensed from the applicator. At each visit during the 4-week dosing period, participants were asked about menstruation, sexual activity, condom use, concomitant medications, and adverse events. At the visits before metronidazole (baseline), after metronidazole (randomisation), during week 4 (immediately after cessation of product), and during week 8 (4 weeks after cessation of product), participants had a physical and gynaecological examination to collect vaginal swabs, cervical swabs, cervicovaginal lavage, and lastly (to avoid any contamination of the cervicovaginal lavage with blood) an endocervical cyto-brush (not collected at the visit before metronidazole) to assess microbial composition, cytokines, mucosal markers of epithelial integrity, and endocervical HIV target cells. Samples were transported to the laboratory cold (2–8° C) and stored at −80° C until analysis. Participants were asked about presence and intensity of expected and prespecified symptoms, which were recorded as solicited local (involving the genitourinary tract), solicited systemic, or unsolicited adverse events at the twice per week visits until the end of week 4, and the week 8 follow-up visit.

Taxonomic classification of bacterial strains present in the cervicovaginal samples was identified through sequencing the 16S rRNA V4 gene region.^15^ Denoising and removal of sequencing errors from the Illumina amplicon reads was done with DADA2 (version 1.26). Taxonomic assignments were made with the Genome Taxonomy Database (version R207) and curated taxonomy from a previous vaginal microbiome study.^16^ Samples were categorised according to the relative abundance of *Lactobacillus* spp and *L crispatus* (>50% *L crispatus*; >50% *Lactobacillus* spp but <50% *L crispatus*; or <50% *Lactobacillus* spp). The quantitative PCR assays targeting *L crispatus*, *L iners*, total 16S rRNA copies, and CTV-05 were done as described previously.^12,17,18^ Cytobrush samples were processed on the day of collection, stained with fluorescently labelled monoclonal antibodies for the surface markers HLA-DR, CD8, CD45, EpCAM, CD4, CCR5, CD14, CD19, CD38, CD11c, and CD66b and viability dye, and underwent flow cytometric analysis as previously described^1,8^ (antibody details in the appendix pp 21–22; gating strategy included in the appendix p 2). We measured concentrations of 20 cytokines or chemokines in undiluted cervicovaginal lavage fluid with a Custom Milliplex High Sensitivity 20-Plex Luminex Kit (EMD Millipore Sigma, Burlington, MA, USA), as previously described.^1,8^ The analyte panel included CXCL10, IL-8, IL-6, CXCL9, CXCL11, IL-1α, IL-1β, MIP-1α, MIP-1β, MIP-3α, TNFα, IL-21, IL-17, IFNγ, IL-23, IL-12(p70), IL-13, IL-10, IL-4, and IL-5. Complete details of laboratory methods are in the appendix (pp 18–23).

### Outcomes

Our primary safety outcome was the proportion of participants reporting product-related adverse events until the end of week 8. Adverse events were graded per either the Division of AIDS Female Genital Grading Table for Use in Microbicide Studies^19^ or the Division of AIDS Toxicity Table for Grading the Severity of Adult and Pediatric Adverse Events (version 2.1).^20^ Our primary microbiological outcome was the proportion of participants with a *Lactobacillus*-dominant vaginal microbiome at week 4 and week 8. Our first immunology coprimary outcome was change in vaginal fluid immune markers between baseline (before metronidazole) and week 4, specifically the proportion of people with at least a 1 log10 decrease in at least three of eight markers previously associated with HIV acquisition. A coprimary immunological outcome was the change in the proportion of endocervical HIV target cells (ie, CD4+CCR5+HLA-DR+CD38+ T cells) between randomisation (ie, after completion of metronidazole treatment) and week 4. Although we had proposed examining change in immune cells from before metronidazole and week 4, we were unable to conduct flow cytometry on samples from the baseline visits. Thus, we compared the change in immune cells between the randomisation visit and week 4 visit.

Secondary microbiological outcomes included comparison of *L crispatus* versus *L iners* prevalence between treatment groups; persistence of colonisation with *L crispatus* CTV-05 after stopping therapy; the proportion of participants with a positive bacterial vaginosis diagnosis in each study group by week 8; stability of vaginal microbial communities over time in the treatment group compared with the placebo group; the detection of CTV-05 by quantitative PCR at week 4 and week 8; and the proportion of participants with cervicotypes 1–4 compared by study group (for this analysis we merged cervicotypes 3 and 4 into a single <50% *Lactobacillus* group).

Secondary immunological outcomes comprised change in genital tract immune markers between baseline and week 8; change in genital tract immune markers between randomisation and week 4 or week 8; and change in immune cell populations between randomisation and week 8. An additional secondary outcome was acceptability of the intervention.

Additionally, we propose the following secondary outcomes that are not reported in this Article but will be included in future papers: anti-HIV effect of vaginal secretions relative to placebo (experiments still ongoing); effect of sex or vaginal hygiene practices (or both) on colonisation by lactobacilli (analysis ongoing); and participants’ stated willingness to use this type of product in the future (analysis ongoing).

### Statistical analysis

The sample size calculation was based on comparing the proportion of participants in each group with at least a 1 log_10_ decrease in at least three of eight cytokines in cervicovaginal lavage specimens at week 4 (ie, the first immunology coprimary outcome). This endpoint is adapted from a study showing an increased risk of HIV acquisition in women with the highest concentrations of at least three of nine vaginal fluid cytokines.^21^ With the target sample size of 60 women (a third in the placebo group and two-thirds in the LACTIN-V group), there would be more than 88% power to detect a 40% absolute difference (10% *vs* 50%) in the proportion of women with decreased inflammation between the two groups. This power calculation was based on a simulation study with 10 000 Monte Carlo samples and two-sided Fisher’s exact test with type 1 error rate of 5%.

Progress of the trial was delayed (1-year delay because of COVID-19 in 2020, a 4-month pause because of damage to the clinic site during local social unrest in July, 2021, and a 2-week pause due to severe flooding of the area in 2022), and after consultation with investigators, the data safety and monitoring board, and staff at the US National Institute of Child Health and Human Development, the intended sample size was reduced. The data safety and monitoring board met before the trial start, after 30 participants were enrolled, and after trial conclusion. A sample size of 37 participants provided adequate power (>80%) to detect effect sizes (differences) of 50% (control 10% *vs* intervention 60%). By the agreed-upon date to close enrolment, we had randomly assigned 45 participants into groups in the trial.

Safety analyses were done in the intention-to-treat population. We estimated a risk ratio for overall report of solicited adverse events. CIs for adverse event rates were estimated with methods for exact binomial CIs. All comparisons (not related to safety) of the LACTIN-V group versus placebo group were done in a modified intention-to-treat population, excluding a placebo participant who was erroneously dispensed one dose of LACTIN-V (appendix p 17). Wilcoxon rank-sum was used to compare continuous participant characteristics and Fisher’s exact tests for categorical characteristics between the two groups.

Baseline and post-baseline differences in continuous outcomes were compared with Wilcoxon signed-rank test. p values are two sided and are not corrected for multiple hypothesis testing; a p value of less than 0·05 was considered statistically significant. All listed tests were run with the gtsummary package in R (version 1.7.2). Benefit ratios and CIs were calculated with the riskratimo.wald() in the epitools package in R (version 0.5.10.1). For Luminex data, some analytes had a large proportion of values less than the lower limit of detection, creating a skewed dataset. For analytes in which less than 65% of values were quantifiable, we decided to analyse the data as a categorical outcome: detected versus not detected. We did an exploratory analysis to assess the correlation between relative abundance of *Lactobacillus* and each analyte with Spearman rank correlation.

### Role of the funding source

The funders of the study had no role in study design, data collection, data analysis, data interpretation, or writing of the report. Staff members at Osel reviewed portions of the protocol that described LACTIN-V and placebo, including storage requirements, and were involved in writing the manuscript. Osel also provided both LACTIN-V and placebo filled vaginal applicators.

## Results

**Figure 1:**
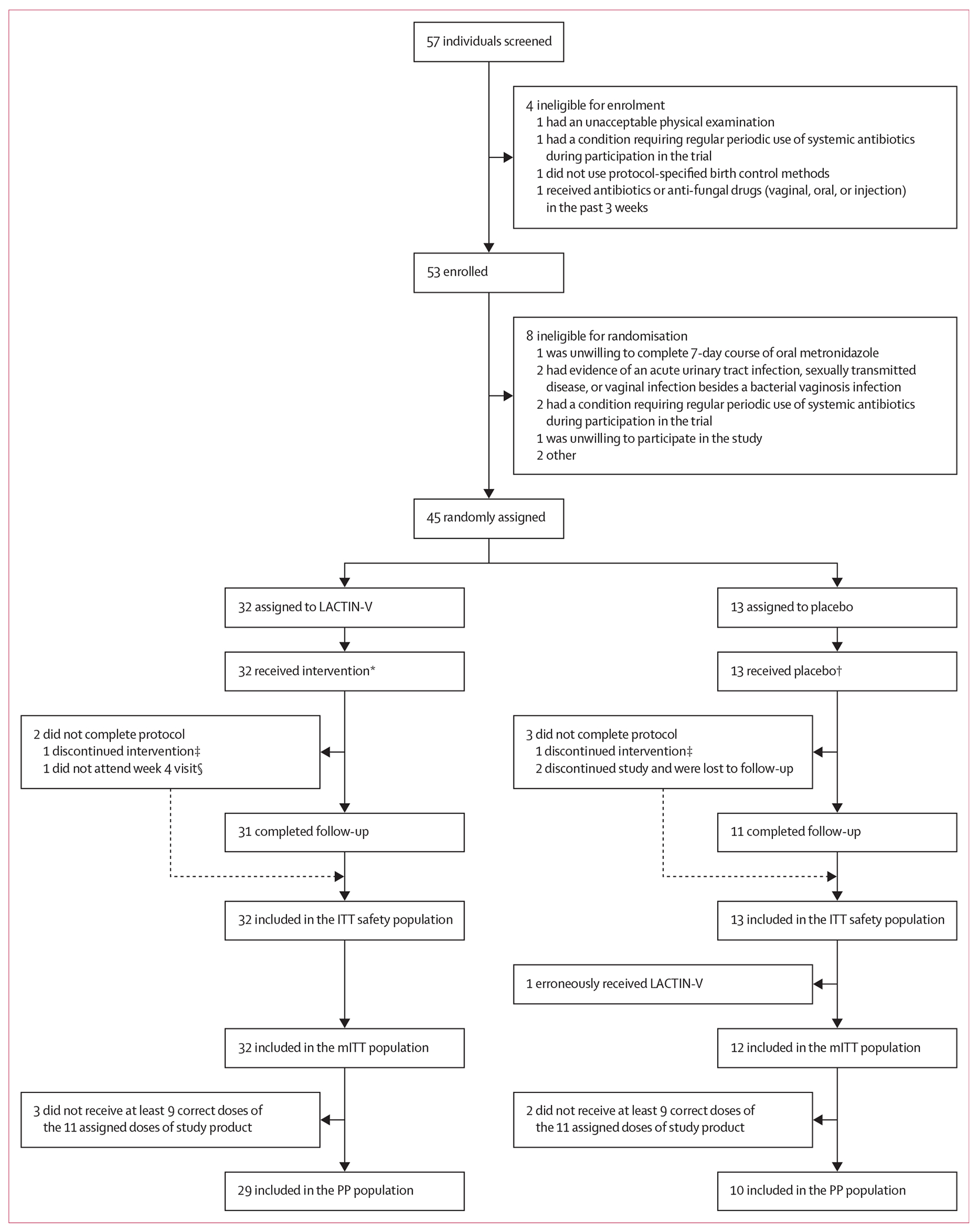
Study profile *Three people received one or two applicators containing placebo, dispensed in error. †One person received one applicator containing LACTIN-V, dispensed in error. ‡Participant continued follow-up despite discontinuing intervention. §Participant still attended week 8 appointment. ITT=intention to treat. mITT=modified ITT. PP=per protocol.

Among 57 women screened between May 11, 2021, and Dec 5, 2022, 53 women were eligible for enrolment and 45 were eligible for randomisation after completion of metronidazole. 32 women were assigned to the LACTIN-V group and 13 to the placebo group (figure 1). The mean age of participants in the LACTIN-V group was 21·3 years and in placebo group was 20·4 years (table 1). All participants were using long-acting, injectable, progestogen-only contraception for at least 30 days. Demographic and clinical characteristics were balanced between treatment groups (table 1).

All 45 participants who were randomly assigned received the study product or placebo and were included in the safety analysis. During interruption of clinic visits and damage to the study site due to social unrest, one woman in the LACTIN-V group and one in the placebo group discontinued the intervention, and two more women in the placebo group discontinued the study follow-up during the post-dosing phase. 28 (88%) of 32 participants in the LACTIN-V group adhered to study protocol (ie, took all 11 doses) and 12 (92%) of 13 in the placebo group adhered (one participant missed a single dose).

26 (81%) of 32 participants in the LACTIN-V group had at least one solicited adverse event compared with nine (69%) of 13 participants in the placebo group (risk ratio 1·17, 95% CI 0·79–1·75; p=0·44). All solicited genitourinary (ie, local) adverse events were mild. In both groups, the most common solicited local adverse events were abnormal vaginal discharge, genital itching or burning, vaginal odour, and vaginal bleeding other than menstruation (table 2). The most common solicited systemic adverse events were abdominal pain or cramps and headaches (appendix p 7).

Bleeding other than menstruation occurred in 23 (51%) of 45 participants; however, ten (44%) of these 23 women had already had such bleeding in the months before joining the study. In the LACTIN-V group, ten (31%) of 32 women had new non-menstrual bleeding, but six (60%) of these ten had only started long-acting, progestogen-only contraception 1–3 months before starting the study. In the placebo group, three (23%) of 13 women had new non-menstrual bleeding, of whom one (33%) had started long-acting progestogen-only contraception 1–3 months before starting the study.

Unsolicited adverse events were reported by 11 (34%) of 32 participants in the LACTIN-V group and by none of the participants in the placebo group. Among unsolicited adverse events, all were mild except for five moderate adverse events in the LACTIN-V group. One papular rash on the lower abdominal flank was deemed related to the study product, and the remaining four moderate adverse events unrelated to study product (one case each of subcutaneous abscess, folliculitis, toothache, and genital ulceration). The genital ulceration was diagnosed as probable genital herpes recurrence and resolved within 6 days. No grade 3 adverse events or serious adverse events occurred (appendix pp 8–9). No participants acquired HIV during the trial.

**Figure 2:**
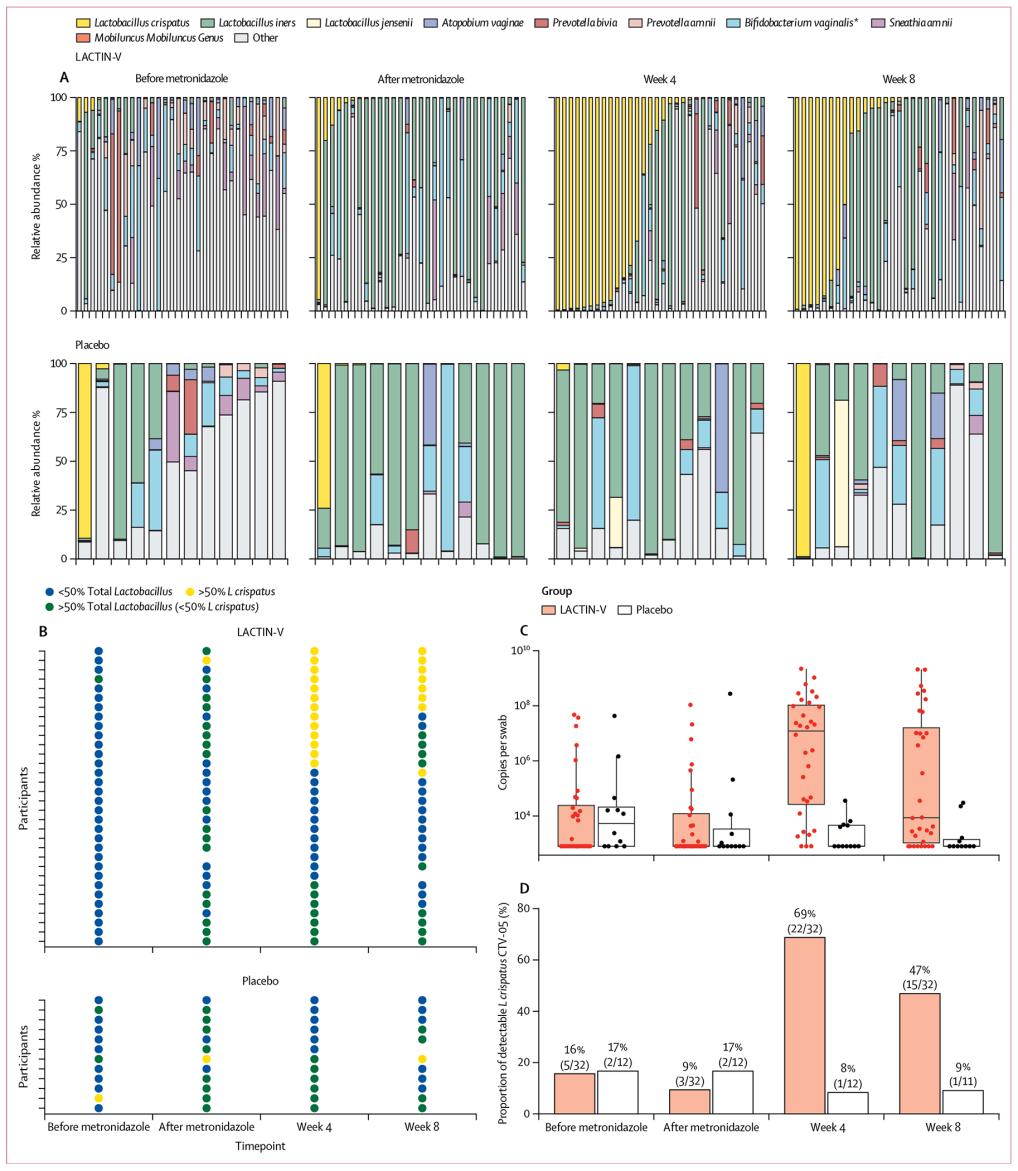
Microbiological outcomes (A) The vaginal microbiome was characterised by 16S rRNA sequencing at 4 timepoints: before metronidazole treatment; after metronidazole treatment and before LACTIN-V or placebo administration; after 4 weeks of LACTIN-V or placebo administration (week 4); and at 8 weeks (4 weeks after stopping LACTIN-V or placebo administration; week 8). Each column on the graphs represents an individual participant. (B) The vaginal microbiome was assigned to one of three categories based on relative abundance of *Lactobacillus crispatus* and *Lactobacillus* genus. (C) Quantitative PCR measured the number of *L crispatus* 16S rRNA gene copies per swab. (D) Quantitative PCR also detected *L crispatus* CTV-05 among those with *L crispatus* present. *Bifidobacterium vaginalis is the new taxonomy for organisms formerly called *Gardnerella vaginalis*.

Before treatment with metronidazole, a diverse bacterial community (marked by <50% *Lactobacillus* spp relative abundance) was the predominant community state in both the LACTIN-V group (30/31; 97%) and placebo group (9/12; 75%), although this was more prevalent in the LACTIN-V group (p=0·027). Notably, one participant who was later randomly assigned to the placebo group had an *L crispatus* dominant community despite having a Nugent score of 8 at screening (figure 2A; appendix p 3). After metronidazole treatment, most participants in both groups had a bacterial community dominated by *L iners*: 17 (55%) of 31 women in the treatment group and eight (67%) of 12 in the placebo group.

After completing the 4 weeks of treatment, participants receiving LACTIN-V were more likely to have an *L crispatus* dominant bacterial community: 13 (41%) of 32 women compared with none in the placebo group (p=0·0088; figure 2B). Dominance of *L crispatus* at week 8 (4 weeks after cessation of product) was observed in eight (26%) of 31 participants who received LACTIN-V compared with one (9%) of 11 in the placebo group (p=0·40). The median absolute quantity of *L crispatus* was significantly higher at week 4 in participants treated with LACTIN-V (1·3 × 10^7^ copies per swab, IQR 2·9 × 10^4^ to 1·1 × 10^8^) than placebo (0 copies per swab, 0 to 4·7 × 10^3^; p=0·0003; figure 2C; appendix p 10). This difference was persistent at week 8, with a median quantity of 8·6 × 10^3^ copies per swab (IQR 8·7 × 10^2^ to 2·3 × 10^7^) in the treatment group versus 0 copies per swab (0 to 1·4 × 10^3^; p=0·0066) in the placebo group. A significantly higher proportion in the LACTIN-V group (22/32; 69%) had detection of *L crispatus* CTV-05 at week 4 than in the placebo group (1/12; 8%; p=0·0004) and this persisted at week 8: 15 (47%) of 32 in the treatment group versus one (9%) 11 in the placebo group (p=0·033; figure 2D; appendix p 10).

At week 4, 12 (39%) of 31 participants in the LACTIN-V group compared with six (50%) of 12 in the placebo group had Nugent scores of 4 or higher (p=0·50). By week 8, 15 (47%) of 32 LACTIN-V participants compared with six (50·0%) of 12 in the placebo group had Nugent scores of 4 or higher (p=0·66). At week 8, nine (28%) of 32 LACTIN-V participants had bacterial vaginosis by Nugent score (ie, ≥7) compared with four (36%) of 11 in the placebo group (p=0·60). When considering dominance of the community by any *Lactobacillus* (>50% relative abundance in 16S rRNA results), there was no significant difference between participants treated with LACTIN-V and placebo at week 4 (LACTIN-V 23/32 [72%]; placebo 6/12 [50%]; p=0·51) and week 8 (LACTIN-V 14/31 [45%]; placebo 6/11 [55%]; p=0·73).

**Figure 3:**
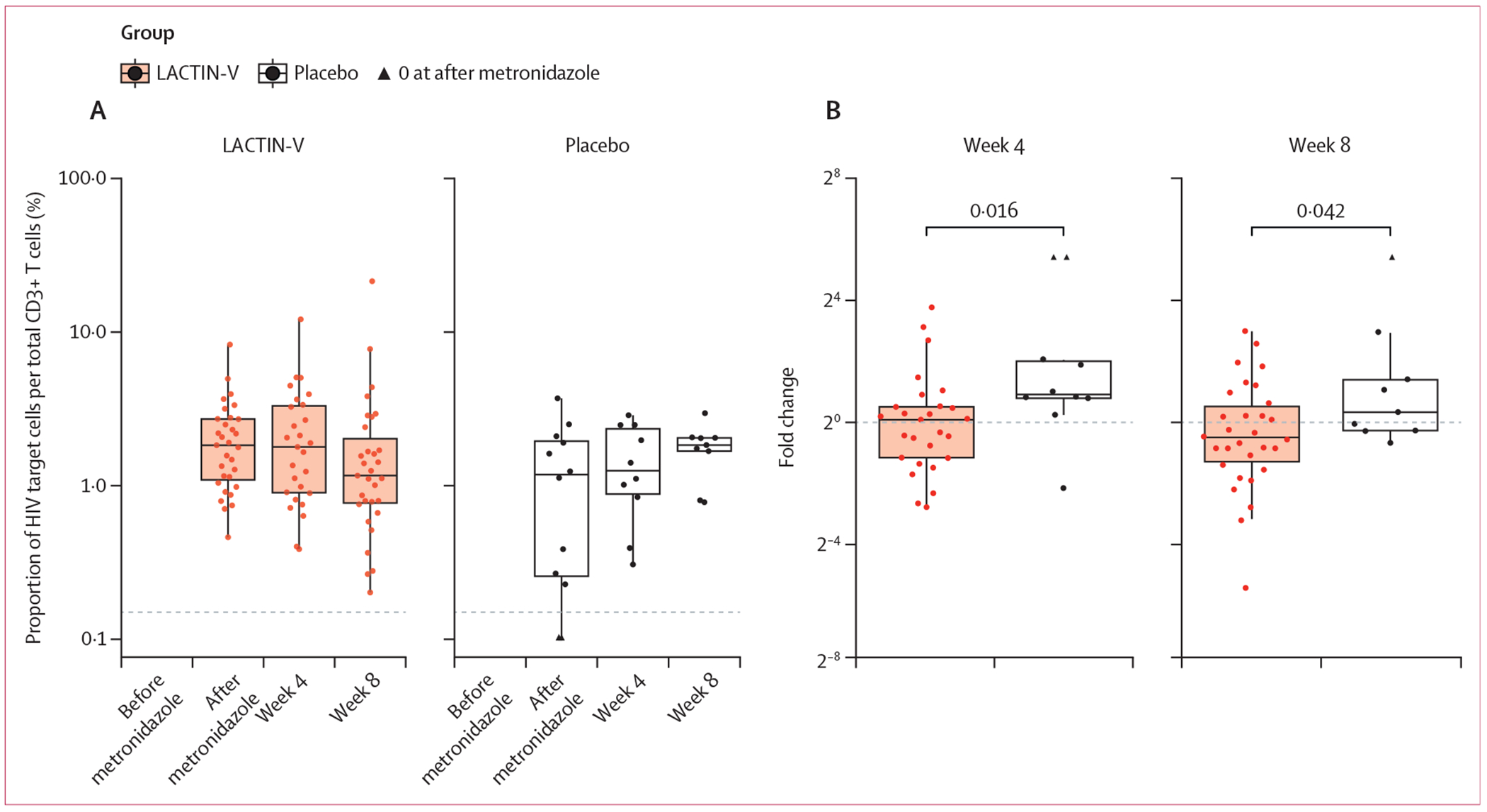
Immunological outcomes for immune cell analysis (A) The proportion of HIV target cells (CD3^+^CD4^+^CD38^+^HLA-DR^+^CCR5^+^ T cells) within the total T-cell population (CD3^+^) was measured in endocervical cytobrush samples through flow cytometry after metronidazole treatment, at 4 weeks, and at 8 weeks. (B) Fold change between the visit after metronidazole treatment and week 4 or week 8. For plotting, in people without the cell type detected during the visit after metronidazole treatment, we set the value for that visit at 0·001; their week 4 values are denoted with black triangles.

Among 37 participants with samples from after metronidazole treatment (ie, at randomisation) and week 4 visits, the change in the proportion of activated HIV target cells (CD45^+^CD3^+^CD4^+^CD38^+^HLA-DR^+^CCR5^+^) out of total T cells (CD45^+^CD3^+^) was higher in the placebo group (1·891, IQR 1·731–4·018) than in the LACTIN-V group (1·062, 0·449–1·424; p=0·016; figure 3A, B; appendix p 10). A similar pattern was seen at week 8. Samples from before metronidazole treatment (ie, baseline) were not collected for flow cytometry.

On evaluation of the prespecified decreased inflammation outcome (via a measure of ≥1 log_10_ decrease in three of eight markers between samples before metronidazole treatment and week 8), we found that two (18%) of 11 participants in the placebo group and three (10%) of 30 in the LACTIN-V group met this metric (p=0·60). At week 4, there were none in the placebo group and four (13%) of 31 women in the LACTIN-V group who met this metric (p=0·56). There was no difference in these findings if we compared change between the visit after metronidazole treatment and week 4 or week 8 (placebo 3/11 [27%], LACTIN-V 3/31 [10%] at both timepoints; p=0·60).

**Figure 4:**
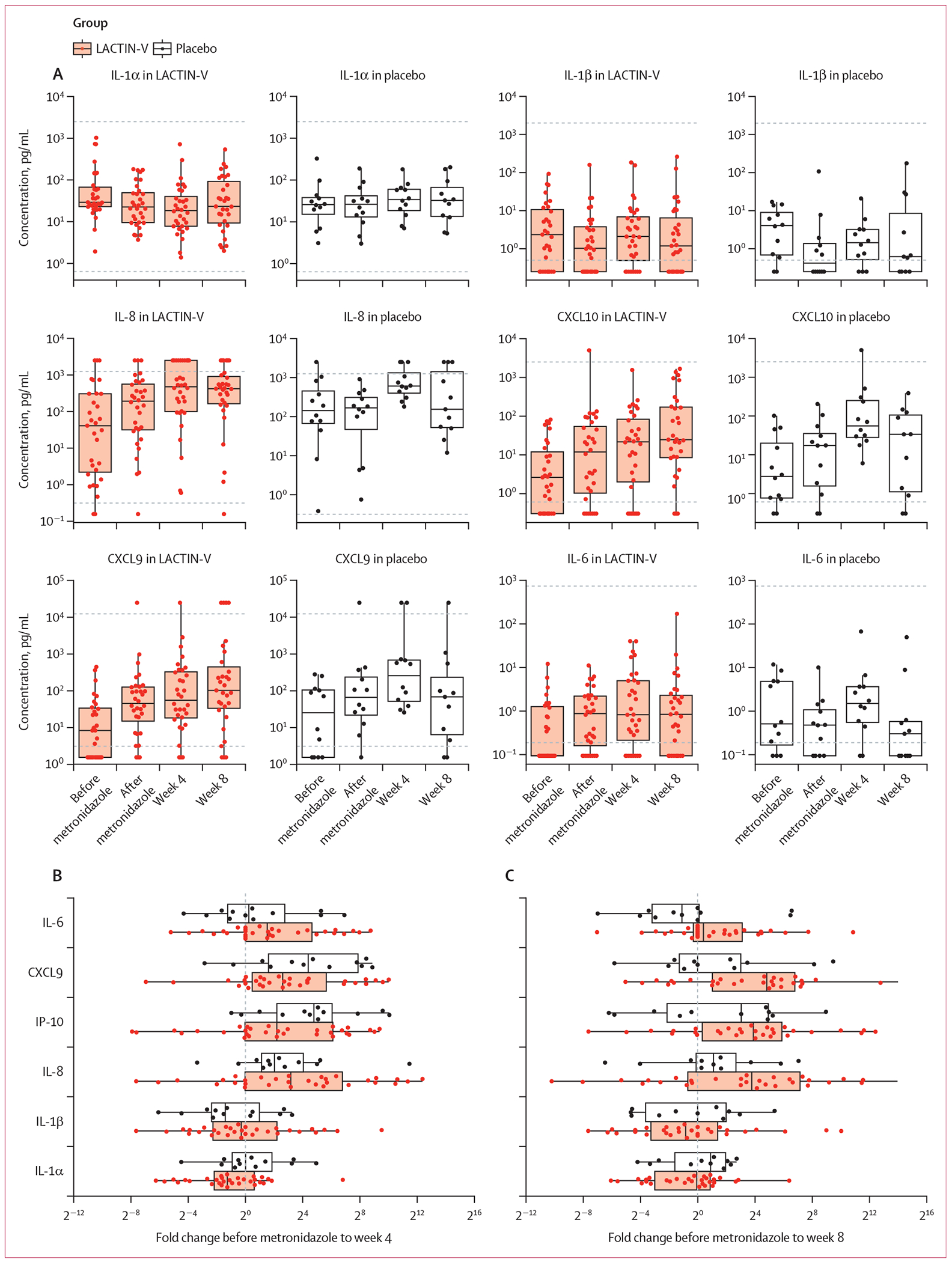
Immunological outcomes for immune marker analysis (A) 12 vaginal fluid immune markers were measured by Luminex at the visits before metronidazole treatment, after metronidazole treatment, at week 4, and week 8. The change between the visit before metronidazole treatment and week 4 (B) or week 8 (C) was calculated for those analytes quantifiable in 65% or more of samples.

At least two patterns of change in soluble immune markers were seen: some decreased or remained unchanged after antibiotic treatment and study product and some increased during the same period (figure 4A). For within-person quantitative change, we analysed six analytes (IL-1α, IL-1β, IL-6, IL-8, CXCL9, and CXCL10) that were quantifiable in at least 65% of participant samples. At week 4, LACTIN-V participants had a median fold change in IL-1α from the visit before metronidazole of 0·415 (IQR 0·221–1·532) compared with 1·048 (0·539–4·334) in the placebo, although this difference did not reach statistical significance (p=0·076; figure 4B, appendix p 11). By week 8, the median log_2_ fold change was 0·963 (IQR 0·134–1·835) in the LACTIN-V group compared with 1·870 (0·437–3·878) in the placebo group (p=0·29; figure 4C; appendix p 11). None of the other five quantifiable factors showed a statistically different change between groups from baseline to week 4 or week 8. We categorised the remaining markers as detectable or undetectable and compared the change between those groups; there were no significant differences between groups (appendix pp 5, 12–13). In a post-hoc analysis, in which we evaluated the associations between concentrations of individual cytokines and the relative abundance of *Lactobacillus*, we found weak negative associations for IL-1α, IL-1β, IL-23, MIP-1α, and TNFα and weak positive associations for CXCL10, CXCL9, CXCL11, and MIP-3α (appendix p 5).

38 (88%) of 45 participants across both groups were satisfied with use of the applicator, 37 (82%) of 45 felt comfortable inserting it, and 41 (91%) of 45 stated that it was easy to use. Overall, 32 (71%) of 45 women strongly agreed or agreed they would use the product again whereas eight (18%) of 45 remained neutral and three (7%) of 45 disagreed or strongly disagreed; two (4%) of 45 did not provide responses. We did not ask for the reasons underlying these ratings.

## Discussion

In this cohort of young South African women at high risk for HIV acquisition and with an atypical Nugent score (4–10), we found significantly more women treated with antibiotics plus LACTIN-V for 4 weeks had an *L crispatus* dominant vaginal microbial community than those who received placebo, although this difference was no longer significant 4 weeks after product cessation. After this short treatment course, activated endocervical CD4^+^ HIV target cells increased significantly more in the placebo group than in the LACTIN-V group, both immediately after the cessation of product use and 4 weeks after cessation. We did not find a difference in the change in vaginal fluid immune markers between the two groups. Importantly, we found this vaginally delivered intervention was highly acceptable and safe for young African participants.

Although the CTV-05 strain was isolated from an American woman’s vaginal microbiome, it colonised most South African participants who received the study product, showing potential efficacy across geographies and populations. Studies in Africa of vaginal probiotics containing *Lactobacillus* species that are more commonly found in the gut have not shown evidence of vaginal colonisation or reduction in bacterial vaginosis recurrence, and they are usually assessed as food supplements and do not use effectiveness standards required for investigational drugs.^22^ Thus, our results are the first demonstration of significant vaginal colonisation with a live biotherapeutic *Lactobacillus* among women in Africa. The goal of a live biotherapeutic product in this context would be to deliver a self-renewing intervention to reduce the risk for HIV acquisition after a limited duration of dosing. Although the durability of this intervention needs to be improved, these results are a promising first step.

We have previously shown higher frequencies and proportions of endocervical activated CD4^+^ and CD8^+^ T cells among young women in Africa than in the USA, which might reflect the increased susceptibility and HIV incidence among young women in Africa.^23^ Similarly, in the FRESH cohort of young women we showed a significantly lower risk for HIV acquisition, lower soluble vaginal fluid immune markers, and fewer endocervical CD4^+^ T cells in people with an endogenous *L crispatus* dominant vaginal microbial community than people with a diverse microbiome.^1^ By contrast, a cross-sectional analysis of Black South African women and Black Canadian women found no difference in HIV target cells between women with *L crispatus* dominance versus other microbial communities.^24,25^ In this study, people treated with LACTIN-V had no change in the median proportion of endocervical activated CD4^+^ T cells from after metronidazole treatment to week 4 or week 8 visits, whereas the placebo group had an increase. Few other studies have looked at endocervical immune cell populations after treatment for bacterial vaginosis; in a population of young South African women, people who had persistent bacterial vaginosis after treatment had a higher proportion of activated CD4^+^ T cells than those who no longer had bacterial vaginosis.^26^ A study in Kenya found metronidazole therapy was associated with a significant decrease in the proportion of CD69^+^CD4^+^ activated T cells, although not the proportion of CD4^+^ T cells expressing CCR5, α4β7, or α4β1.^27^ More participants in the LACTIN-V group had *L crispatus*-dominant communities at week 4 and week 8, suggesting that the presence of *L crispatus* could prevent a rebound in the proportion of activated HIV target cells.

Our participants were sexually active and were using injectable contraceptives, primarily depot medroxyprogesterone acetate. One study found increased proportions of cervical HIV target cells after penile–vaginal sex, especially condomless sex, which persisted for 72 hours after intercourse.^28^ Several studies have found that initiation or use of depot medroxyprogesterone acetate increases the proportion of endocervical HIV target cells.^29,30^ Thus, the absence of increase in the proportion of cervical HIV target cells in the LACTIN-V group during follow-up is all the more biologically important.

Our data are consistent with previous studies showing a complex association between soluble vaginal inflammatory markers and the vaginal microbiota. There is substantial interindividual variation in starting values and in the direction of change in values between visits. In the USA, LACTIN-V taken for 11 weeks was associated with a sustained reduction of IL-1α and soluble E-cadherin compared with placebo at 24 weeks.^13^ However, in this current study no difference in any of the evaluable markers was observed between groups in the change from values before antibiotic treatment to after LACTIN-V administration. In previous studies, people at the highest risk for HIV had the highest quartile values for a subset of nine vaginal fluid markers of inflammation.^1,21^ We measured eight of the same markers but did not observe a consistent pattern in change from before to after treatment among participants treated with LACTIN-V compared with placebo. Some markers that were part of the HIV-risk group in the CAPRISA study, such as IP-10 and IL-8, actually increased after treatment for bacterial vaginosis, both in this and other studies.^27^ Thus, identifying a means to follow inflammation longitudinally is challenging, as there is no single marker of inflammation that captures all patients with greater risk for adverse outcomes, nor is there a consistent trajectory across markers.^21^

Our participants were young women in the age group at highest risk for HIV acquisition, recruited from a large periurban area, and reporting little use of vaginal products before participation in the study. Despite their inexperience with vaginal products, this group found the product highly acceptable. There was no significant difference in potential adverse events, such as vaginal discharge. Of note, the high incidence of irregular bleeding in both the LACTIN-V and placebo groups was probably due to the high proportion of participants that initiated depot medroxyprogesterone acetate just 1–3 months before enrolment.

Our study has limitations. The design of our study limits direct comparison with the previous phase 2b trial in the USA in several ways. A smaller sample size, combined with shorter dosing and follow-up intervals, prevents assessment of long-term effects of LACTIN-V treatment in this population, something we hope to address with a larger follow-up study. The standard of care for bacterial vaginosis treatment in South Africa is oral metronidazole, which was used in this study, rather than vaginal metronidazole gel used in the US phase 2b study. Our reliance on Nugent scoring alone, as compared with the combination of Gram stain and point-of-care Amsel criteria on wet mount, is not the standard US Food and Drug Administration criterion for studies of bacterial vaginosis, and limits comparability. The placebo group was more likely to have a non-diverse microbial community at the baseline visit before metronidazole, which could have biased results towards the null. We were unable to adjust for behavioural risk factors in this small study, so we cannot comment on whether bleeding or contraceptive choice or intercourse had an important effect on outcomes such as colonisation with *L crispatus*. Last, study logistics at the site prevented the collection and immediate preparation of endocervical cytobrush samples at the baseline visit before metronidazole.

We calculated our sample size on the basis of being able to detect decreased inflammation in a higher proportion of participants treated with LACTIN-V, as a proxy for HIV acquisition risk. However, although cross-sectional analyses show an association between low *Lactobacillus*, higher levels of multiple soluble immune markers, and HIV acquisition, those markers did not consistently decrease after treatment for bacterial vaginosis or colonisation with *L crispatus*. There was more *L crispatus* dominance in the LACTIN-V group, but there were similar amounts of total *Lactobacillus* dominance due to participants with high concentrations of *L iners*. Additionally, for some of the 8 markers we used to define inflammation, we could not measure quantifiable concentrations in a moderate proportion of participants, which limited the utility of those markers. Our results suggest that the use of genital immune markers to track HIV risk during interventions to alter the vaginal microbiome is not straightforward and needs further investigation and the development of a standardised approach.

In conclusion, despite a low prevalence of *L crispatus* dominant vaginal microbial communities in young South African women, *L crispatus* CTV-05 colonised the majority of participants in the LACTIN-V group. This single-strain product, used intermittently for 4 weeks, was detected in 69% of the participants and transiently transformed more than 40% of participants’ vaginal microbial communities to the pattern associated with the lowest risk for HIV acquisition. Thus, we believe that the cumulative evidence supports further exploration of *L crispatus* live biotherapeutics as a female-controlled intervention to reduce HIV acquisition in cisgender women.

## Supplementary Material

1

See **Online** for appendix

## Figures and Tables

**Table 1:** Baseline characteristics in the intention-to-treat population

	LACTIN-V (n=32)	Placebo (n=13)
Age, years		
Mean (SD)	21·3 (1·4)	20·4 (1·3)
Median (IQR)	22 (20–22)	20 (20–21)

Race or ethnic group		
Black African	32 (100%)	13 (100%)

Completed high school	22 (69%)	9 (69%)

Current relationship status		
Married*	0	0
Single (no current partner)	3 (9%)	0
Steady partner, cohabitating	4 (13%)	1 (8%)
Steady partner, not cohabitating	22 (69%)	11 (85%)
Casual partner	3 (9%)	1 (8%)

Sexual intercourse in the previous 30 days	26 (81%)	12 (92%)

Number of previous pregnancies	1 (0–2)	1 (0–2)

Number of male sexual partners during their lifetime	3 (1–8)	2 (1–4)

Number of male sexual partners in the past 6 months	1 (1–3)	1 (1–1)

Data are median (range) or n (%), unless otherwise indicated.

* Married group includes those who are divorced, separated, or widowed.

**Table 2:** Number and percentage of participants experiencing local solicited adverse events by symptom and treatment group

	LACTIN-V (n=32)	Placebo (n=13)	All participants (n=45)
**Solicited adverse events**			
Any solicited adverse event	26 (81%; 64·4–91·5)	9 (69%; 41·3–88·7)	35 (78%; 63·6–88·5)

**Solicited local adverse events**			
Any local adverse event	25 (78%; 61·2–90·1)	8 (62%; 33·7–83·4)	33 (73%; 58·9–85·2)
Grading			
Mild	25 (78%; 61·2–90·1)	8 (62%; 33·7–83·4)	33 (73%; 58·9–85·2)
Moderate	0	0	0
Severe	0	0	0
Abnormal vaginal discharge	9 (28%; 14·8–46·2)	2 (15%; 2·8–43·4)	11 (24%; 13·8–38·7)
Abnormal vaginal odour	3 (9%; 2·6–24·3)	0	3 (7%; 1·8–18·2)
External genital irritation	1 (3%; 0·2–16·2)	0	1 (2%; 0·1–11·5)
External genital swelling	1 (3%; 0·2–16·2)	0	1 (2%; 0·1–11·5)
Genital itching or burning	8 (25%; 12·2–42·3)	4 (31%; 11·3–58·7)	12 (27%; 14·8–41·1)
Vaginal bleeding other than menstruation	18 (56%; 38·8–72·4)	5 (38%; 16·6–66·3)	23 (51%; 36·4–65·9)

Data are n (%; 95% CI). The denominator for percentages is based on the number of participants in the safety population. A participant is only counted once within each solicited adverse event.

## Data Availability

The study protocol and statistical analysis plan are included in the appendix (pp 25, 125). The 16S rRNA sequences generated for this analysis have been deposited in the NCBI Short Read Archive (BioProject 1085249). Access to additional data from this study can be requested from the corresponding authors, although it would require participants’ approval and a signed data access agreement.

## Research in context

### Evidence before this study

On Oct 1, 2024, we searched PubMed since database inception with the terms (((Lactobacillus) AND (vaginal)) AND (probiotic OR live biotherapeutic) AND (bacterial vaginosis) AND (randomized trial) NOT (review[Publication Type]) AND (humans[Filter])), which returned 74 results. Of these, eight papers were from phase 1, 2a, and 2b studies of LACTIN-V done in the USA. The phase 2a and 2b studies showed 70–80% *Lactobacillus crispatus* CTV-05 colonisation during use of the product, and retention of *L crispatus* CTV-05 in approximately 48% of participants 13 weeks after cessation of product use. Of the other seven studies that evaluated a product containing *L crispatus* in people with bacterial vaginosis (six oral products and one vaginal), none assessed vaginal colonisation with the probiotic isolate, and only two used molecular characterisation of the vaginal microbiome to assess the outcome. In one, no difference in *L crispatus* relative abundance was seen between groups after treatment, whereas the other showed an increase in *L crispatus* relative abundance after treatment but did not show results for the placebo group. We also searched PubMed with the terms (africa OR rwanda OR kenya OR uganda OR south africa OR nigeria OR ethiopia OR zimbabwe OR ghana) AND (probiotic) AND (vaginal) AND (humans[Filter]), which returned 31 responses. Of the seven articles describing interventional studies of vaginal probiotics in Africa, none included *Lactobacillus* species native to the human vaginal microbiota. Finally, we searched PubMed ((probiotic) AND (immune cells)) AND (vagina OR endocervix OR cervix), which returned 31 responses, one of which was an in vivo human study, but which did not assess endocervical immune cells.

### Added value of this study

To our knowledge, this is the first study of a vaginal live biotherapeutic containing *L crispatus* in an African context. Additionally, this is one of few studies that uses molecular methods to assess change in the microbiome after use of an *L crispatus* live biotherapeutic, or to assess vaginal colonisation with the product isolate. This is also one of the first studies to assess endocervical immune cell populations after treatment with a vaginal *L crispatus* live biotherapeutic. Despite a low prevalence of *L crispatus* dominant vaginal microbial communities in young South African women, the live biotherapeutic product LACTIN-V was able to colonise the majority of people who used it and establish dominance that persisted after cessation of treatment in a quarter of participants. Treatment was associated with no rebound in the proportion of HIV target cells in the endocervix compared with placebo.

### Implications of all the available evidence

These results show that vaginal colonisation with an *L crispatus* live biotherapeutic strain is possible in an African population. If colonisation by exogenous vaginal *L crispatus* is associated with the same benefit as endogenous *L crispatus*, use of an *L crispatus* live biotherapeutic could be useful as an intervention to reduce the risk of HIV acquisition and other adverse women’s health outcomes. In a context in which durability of colonisation could be improved, use of an *L crispatus* live biotherapeutic would be a self-renewing prevention strategy to improve reproductive health outcomes.
