# Supplementary material for: Effect of the vaginal live biotherapeutic LACTIN-V (Lactobacillus crispatus CTV-05) on vaginal microbiota and genital tract inflammation among women at high risk of HIV acquisition in South Africa: a phase 2, randomised, placebo-controlled trial
Source: Lancet Microbe. Author manuscript; Available in PMC 2025 Jun 17. (PMC12173475; doi:10.1016/j.lanmic.2024.101037)
Supplement: 1 [file NIHMS2087678-supplement-1.pdf]

THE LANCET
Microbe

Supplementary appendix

This appendix formed part of the original submission and has been peer reviewed. We post it as supplied by the authors.

Supplement to: Hemmerling A, Mitchell CM, Demby S, et al. Effect of the vaginal live biotherapeutic LACTIN-V (*Lactobacillus crispatus* CTV-05) on vaginal microbiota and genital tract inflammation among women at high risk of HIV acquisition in South Africa: a phase 2, randomised, placebo-controlled trial. *Lancet Microbe* 2025. https://doi.org/10.1016/j.lanmic.2024.101037

Supplemental Material

Phase 2 randomized placebo-controlled trial of the vaginal live biotherapeutic LACTIN-V (*Lactobacillus crispatus* CTV-05) among women at high risk of HIV acquisition in South Africa

Anke Hemmerling*, Caroline M. Mitchell*, Suuba Demby*, Musie Gebremichael, Joseph Elsherbini, Jiawu Xu, Nondumiso Xulu, Johnathan Shih, Krista Dong, Vaneshree Govender, Vanessa Pillay, Nasreen Ismail, Gardenia Casillas, Jayajothi Moodley, Agnes Bergerat, Tess Brunner, Lenine Liebenberg, Sinaye Ngcapu, Ian Mbano, Laurel Lagenaur, Thomas P. Parks, Thumbi Ndung'u, Douglas S. Kwon+, Craig R. Cohen+

Table of contents:

**1**	Supplemental Figure 1: Gating strategy for flow cytometry……………………………………..	**2**
**2**	Supplemental Figure 2: Stacked abundance plot of 16S rRNA sequencing at all timepoints…….	**3**
**3**	Supplemental Figure 3: Values for a subset of CVL immune marker values at all visits……….	**4**
**4**	Supplemental Figure 4: Change in proportion of endocervical HIV target cells………..	**5**
**5**	Supplemental Figure 5: Change in detection of a subset of cytokines over time………	**6**
**6**	Supplemental Figure 6: Correlation between *Lactobacillus* relative abundance and individual cytokine values………………………………………………………………………………….	**7**
**7**	Supplemental Table 1: Solicited adverse events……………………………………………………	**8**
**8**	Supplemental Table 2: Unsolicited adverse events…………………………………………………	**9**
**9**	Supplemental Table 3: Comparison of median qPCR values at Week 4 and Week 8….	**11**
**10**	Supplemental Table 4: Detection of LACTIN-V at all visits………………………………………	**11**
**11**	Supplemental Table 5: Median vaginal fluid immune marker concentrations pre-MTZ and at week 4………………………………………………………………………………………………..	**12**
**12**	Supplemental Table 6: Median vaginal fluid immune marker concentrations pre-MTZ and at week 8………………………………………………………………………………………………..	**12**
**13**	Supplemental Table 7: Proportion of samples with detectable immune marker values pre-MTZ and at week 4……………………………………………………………………………..	**13**
**14**	Supplemental Table 8: Proportion of samples with detectable immune marker values pre-MTZ and at week 8………………………………………………………………………………	**14**
**15**	Supplementary Methods………………………………………………………………………………………	**15**
**16**	Study protocol…………………………………………………………………………………………………..	**25**
**17**	Statistical analysis plan………………………………………………………………………………………	**150**

1. **Supplemental Figure 1**: Gating strategy for flow cytometry analysis of endocervical cells

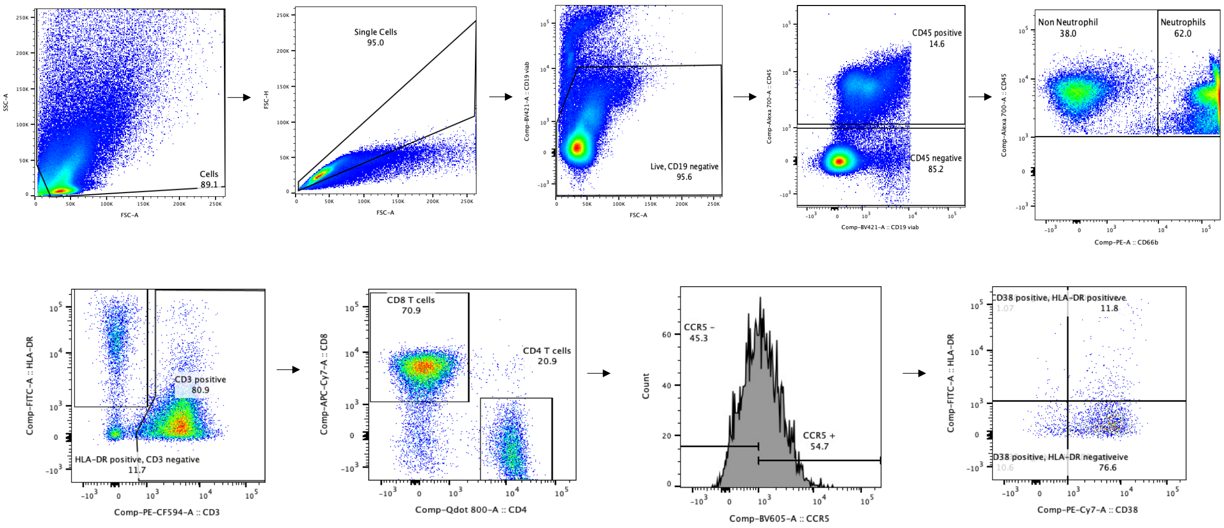

2. **Supplemental Figure 2**: Stacked abundance bar plots of 16S rRNA sequencing of vaginal swab samples at all time points, with identification of all taxa found in taxa at least 5% relative abundance in at least one sample. Of note, *Gardnerella vaginalis* is referred to here using the new nomenclature *Bifidobacterium vaginalis*.

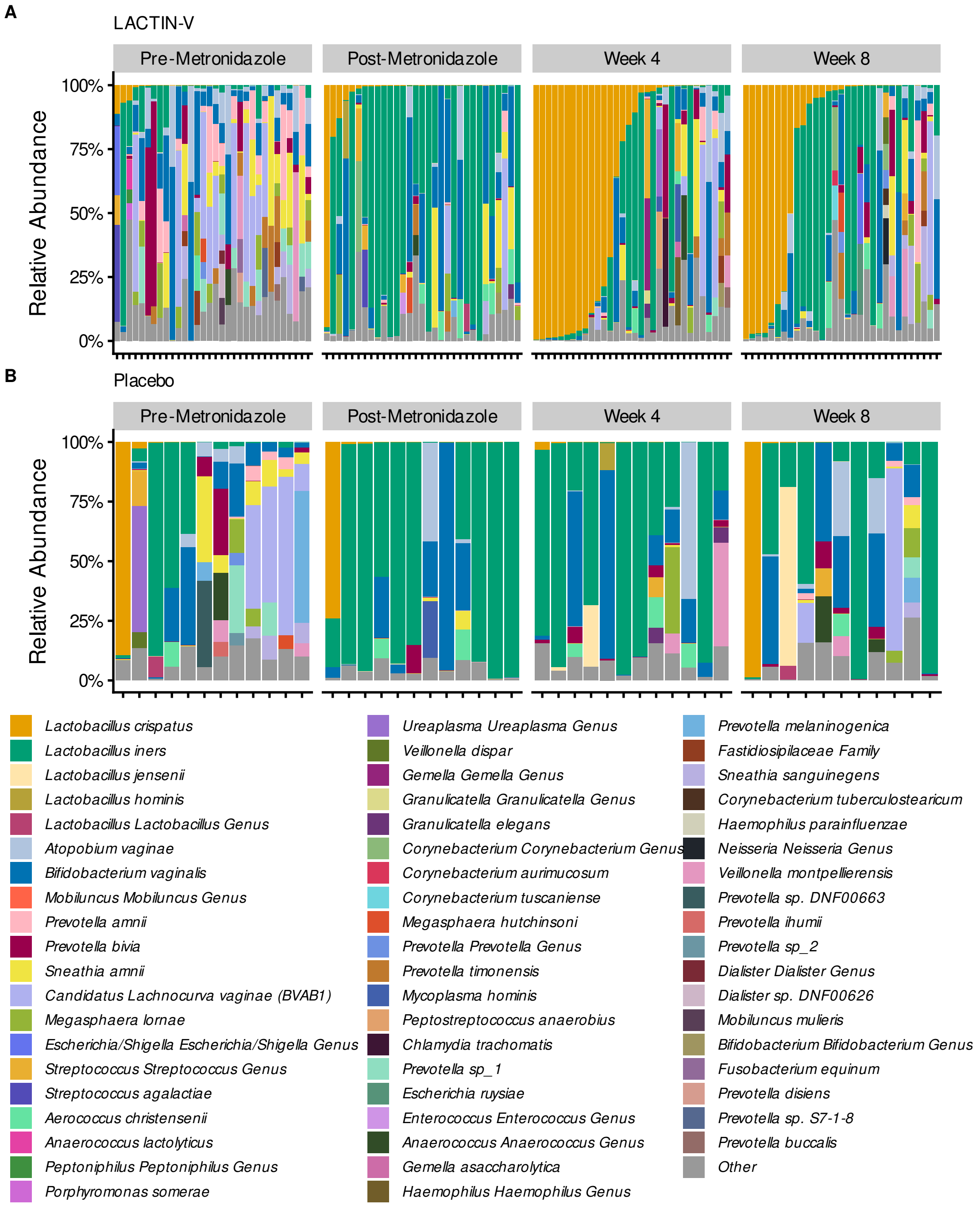

3. **Supplemental Figure 3:** Cervicovaginal lavage immune marker values at each visit, by study arm

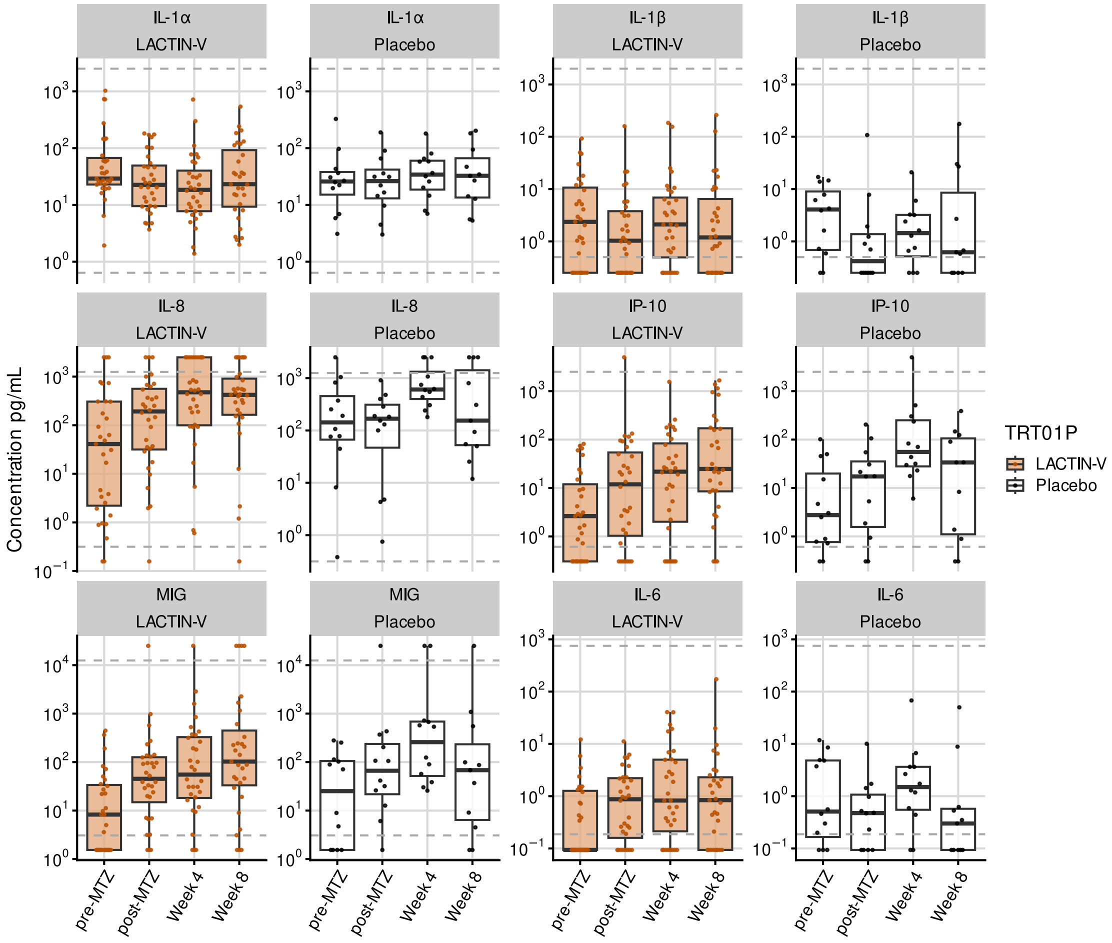

4. **Supplemental Figure 4:** Change in proportion of HIV Target cells (CD3+/CD4+/CD38+/HLA-DR+/CCR5+ T cells) out of total T cells between post-metronidazole visit and (a) week 4 and (b) week 8.

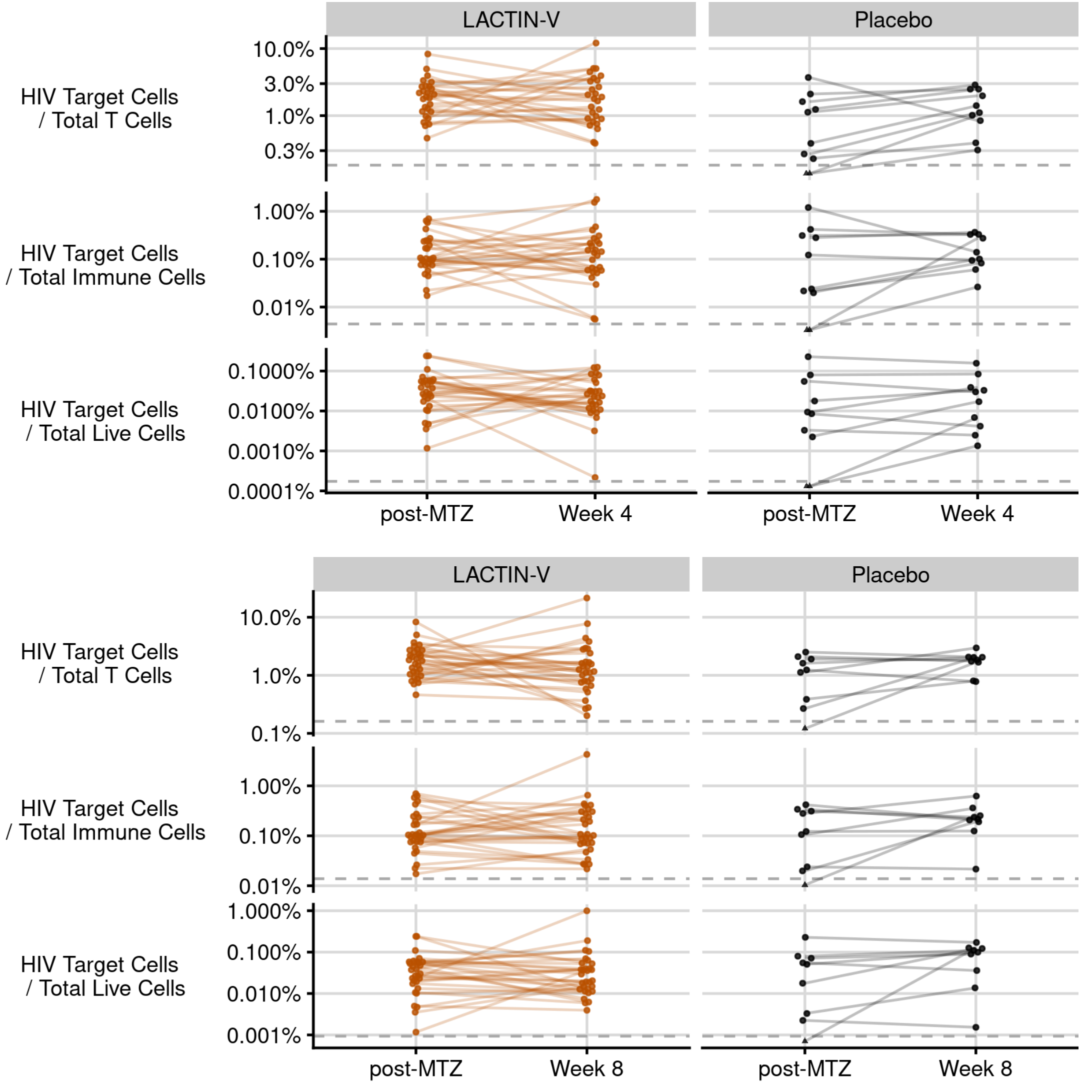

5. **Supplemental Figure 5:** For the seven immune markers with values that were below the limit of detection in >35% of participants, we dichotomized the outcome into detectable vs. undetectable. There were no significant differences between arms in the change from detectable to undetectable (or vice versa) at week 4 (a) or week 8 (b).

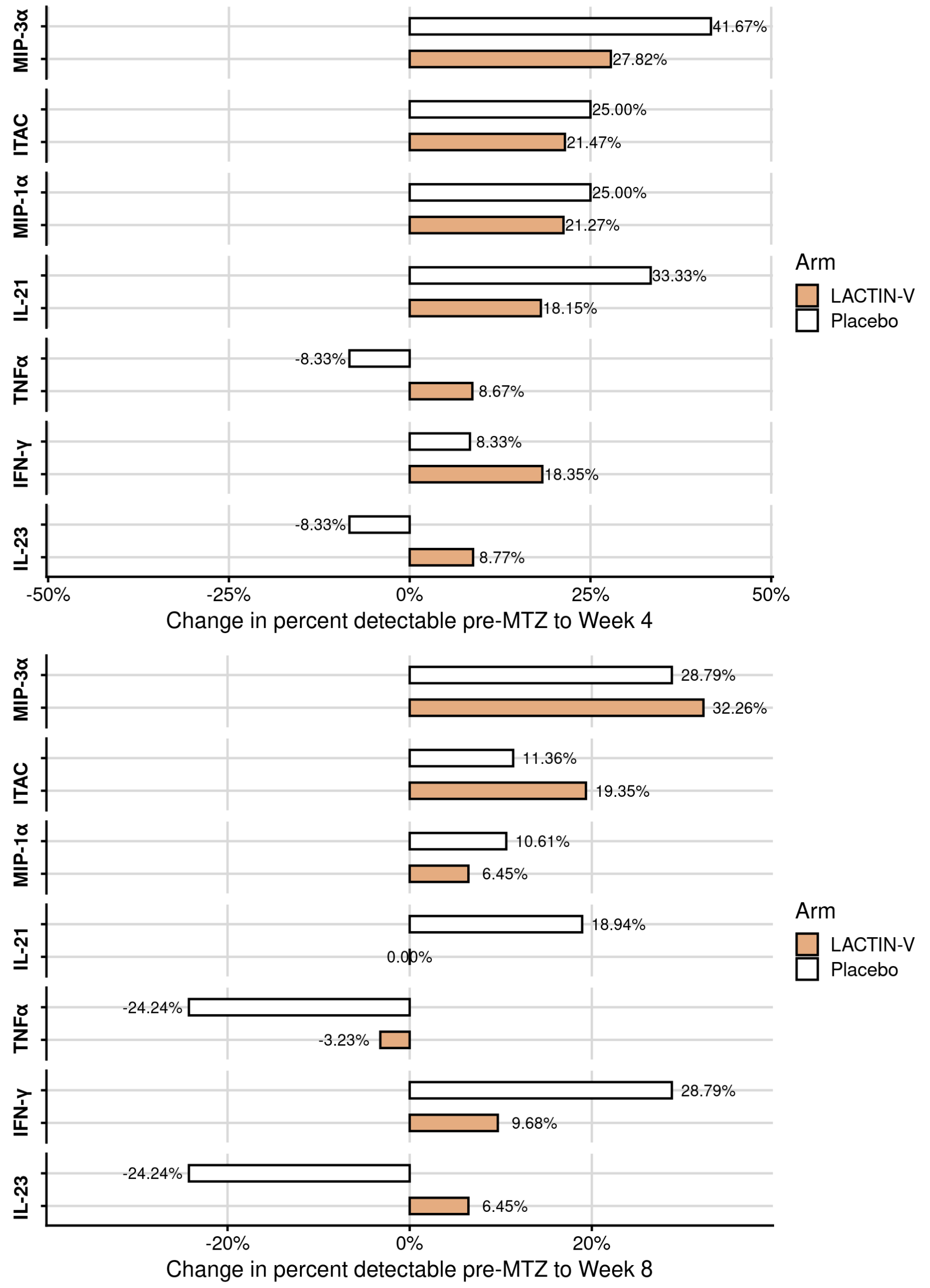

6. **Supplemental figure 6:** We assessed correlation between individual cytokine values and the relative abundance of *Lactobacillus* genus using Spearman rank correlation.

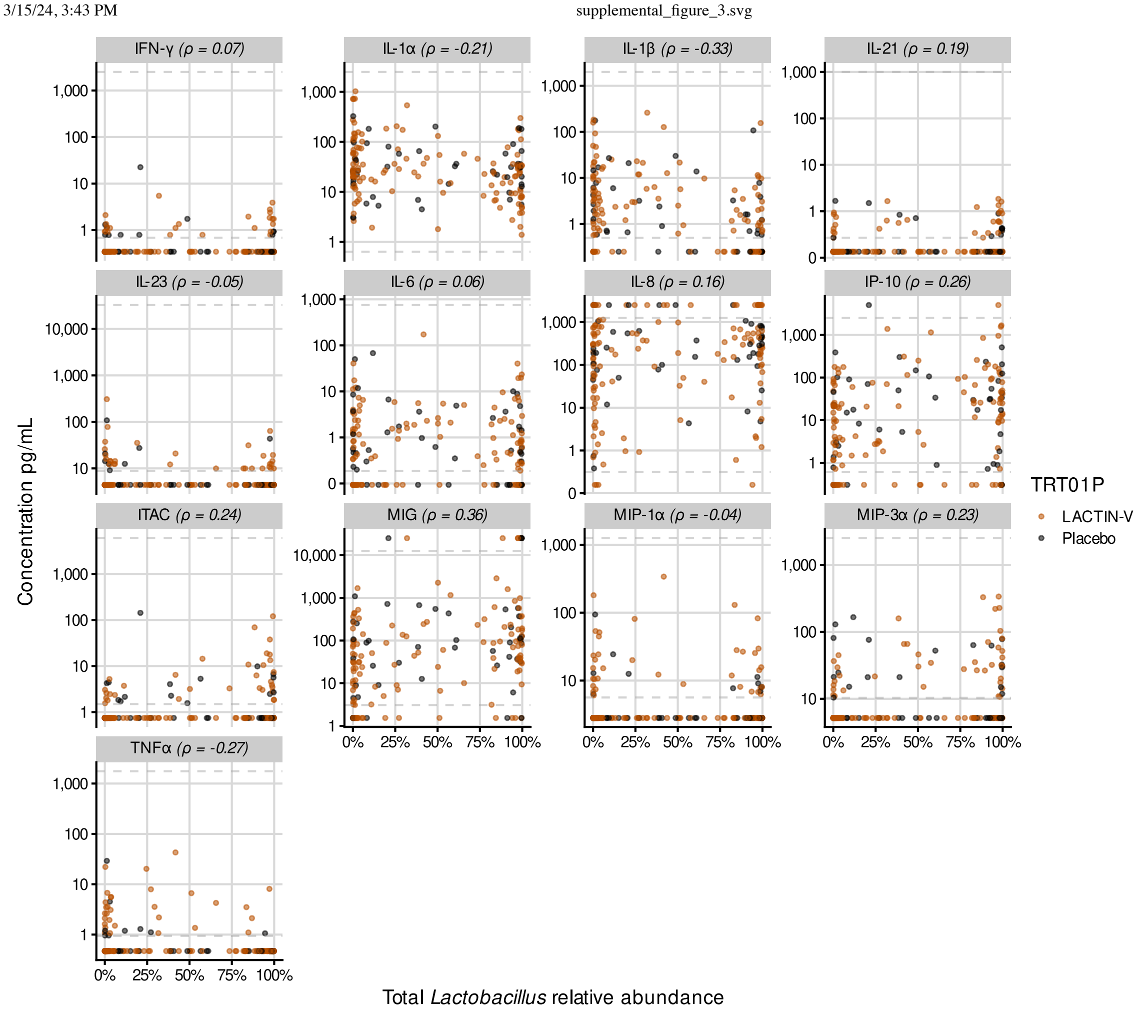

7. **Supplemental Table 1**: Number and Percentage of Participants Experiencing Systemic Solicited Adverse Events with 95% Confidence Intervals by Symptom and Treatment Group – Safety Population

Category	Solicited Adverse Event	LACTIN-V (N=32) n (%) [95% CI]	Placebo (N=13) n (%) [95% CI]	All Participants (N=45) n (%) [95% CI]
Solicited Systemic Adverse Events	Any Systemic Adverse Event	12 (37.5%) [21.7-55.4]	3 (23.1%) [6.6-52.0]	15 (33.3%) [20.6-48.9]
	Grading: Mild	11 (34.4%) [19.6-52.7]	3 (23.1%) [6.6-52.0]	14 (31.1%) [18.2-46.6]
	Grading: Moderate	1 (3.1%) [0.2-16.2]	0	1 (2.2%) [0.1-11.5]
	Grading: Severe	0	0	0
	Abdominal pain/cramps	6 (18.8%) [8.5-35.6]	1 (7.7%) [0.4-33.7]	7 (15.6%) [7.1-28.6]
	Constipation	1 (3.1%) [0.2-16.2]	0	1 (2.2%) [0.1-11.5]
	Diarrhoea	1 (3.1%) [0.2-16.2]	1 (7.7%) [0.4-33.7]	2 (4.4%) [0.8-14.8]
	Frequent urination	1 (3.1%) [0.2-16.2]	0	1 (2.2%) [0.1-11.5]
	Headache	6 (18.8%) [8.5-35.6]	0	6 (13.3%) [6.0-26.2]
	Nausea	2 (6.3%) [1.1-19.6]	0	2 (4.4%) [0.8-14.8]
	Pain/burning with urination	0	2 (15.4%) [2.8-43.4]	2 (4.4%) [0.8-14.8]
	Vomiting	0	1 (7.7%) [0.4-33.7]	1 (2.2%) [0.1-11.5]

Notes:

CI = Confidence Interval. 95% CI = 95% Blaker Confidence Interval.

n (%) = the number (percentage) of participants in each solicited adverse event.

The denominator for percentages is based on the number of participants in the Safety population (N).

A participant is only counted once within each solicited adverse event.

^ Relative risk ratio (95% CI), Pearson Chi-Square p-value

8. Supplemental Table 2: Number and Percentage of Participants Experiencing Unsolicited Adverse Events with 95% Confidence Intervals by MedDRA System Organ Class and Preferred Term by Treatment Group – Safety Population

**MedDRA System Organ Class**<br>**MedDRA Preferred Term**	**LACTIN-V (N=32) n (%) E [CI]**	**Placebo (N=13) n (%) E [CI]**	**All Participants (N=45) n (%) E [CI]**
Any unsolicited adverse event	11 (34.4%) 15 [19.6-52.7]	0	11 (24.4%) 15 [13.8-38.7]
Grading: Mild	6 (18.8%) 10	0	6 (18.8%) 10
Grading: Moderate	5 (15.6%) 5	0	5 (15.6%) 5
Grading: Severe	0	0	0
Gastrointestinal disorders	1 (3.1%) 1 [0.2-16.2]	0	1 (2.2%) 1 [0.1-11.5]
Toothache	1 (3.1%) 1 [0.2-16.2]	0	1 (2.2%) 1 [0.1-11.5]
Infections and infestations	5 (15.6%) 6 [6.4-32.3]	0	5 (11.1%) 6 [4.5-23.9]
Chlamydial infection	1 (3.1%) 1 [0.2-16.2]	0	1 (2.2%) 1 [0.1-11.5]
Eye infection viral	1 (3.1%) 1 [0.2-16.2]	0	1 (2.2%) 1 [0.1-11.5]
Folliculitis	1 (3.1%) 1 [0.2-16.2]	0	1 (2.2%) 1 [0.1-11.5]
Pustule	1 (3.1%) 1 [0.2-16.2]	0	1 (2.2%) 1 [0.1-11.5]
Subcutaneous abscess	1 (3.1%) 1 [0.2-16.2]	0	1 (2.2%) 1 [0.1-11.5]
Viral skin infection	1 (3.1%) 1 [0.2-16.2]	0	1 (2.2%) 1 [0.1-11.5]
Nervous system disorders	2 (6.3%) 2 [1.1-19.6]	0	2 (4.4%) 2 [0.8-14.8]
Dizziness	1 (3.1%) 1 [0.2-16.2]	0	1 (2.2%) 1 [0.1-11.5]
Headache	1 (3.1%) 1 [0.2-16.2]	0	1 (2.2%) 1 [0.1-11.5]
Renal and urinary disorders	1 (3.1%) 1 [0.2-16.2]	0	1 (2.2%) 1 [0.1-11.5]

MedDRA System Organ Class<br>MedDRA Preferred Term	LACTIN-V<br>(N=32)<br>n (%) E [CI]	Placebo<br>(N=13)<br>n (%) E [CI]	All Participants<br>(N=45)<br>n (%) E [CI]
Urine odor abnormal	1 (3.1%) 1 [0.2-16.2]	0	1 (2.2%) 1 [0.1-11.5]
Reproductive system and breast disorders	3 (9.4%) 3 [2.6-24.3]	0	3 (6.7%) 3 [1.8-18.2]
Genital ulceration	1 (3.1%) 1 [0.2-16.2]	0	1 (2.2%) 1 [0.1-11.5]
Vaginal discharge	2 (6.3%) 2 [1.1-19.6]	0	2 (4.4%) 2 [0.8-14.8]
Respiratory, thoracic and mediastinal disorders	1 (3.1%) 1 [0.2-16.2]	0	1 (2.2%) 1 [0.1-11.5]
Nasal congestion	1 (3.1%) 1 [0.2-16.2]	0	1 (2.2%) 1 [0.1-11.5]
Skin and subcutaneous tissue disorders	1 (3.1%) 1 [0.2-16.2]	0	1 (2.2%) 1 [0.1-11.5]
Rash papular	1 (3.1%) 1 [0.2-16.2]*	0	1 (2.2%) 1 [0.1-11.5]

Notes:

CI = Confidence Interval.

n (%) = the number (percentage) of participants in each MedDRA system organ class or preferred term.

E = the number of adverse events in each MedDRA system organ class or preferred term.

The denominator for percentages is based on the number of participants in the Safety population (N).

A participant is only counted once within each MedDRA system organ class or preferred term.

95% CI = 95% Blaker Confidence Interval.

* The only case of an unsolicited AE deemed related to study product

9. **Supplemental Table 3**: Comparison of median qPCR values between arms at Week 4 and Week 8

Characteristic[1]	Week 4			Week 8		
	**LACTIN-V**, N = 32[2]	**Placebo**, N = 11[2]	**p-value**[3]	**LACTIN-V**, N = 32[2]	**Placebo**, N = 11[2]	**p-value**[3]
bacteria	3.7e+08 (5e+07, 7.4e+08)	3.1e+08 (2e+08, 9.9e+08)	0.57	1.9e+08 (3.6e+07, 1.1e+09)	7.3e+08 (1.6e+08, 1.5e+09)	0.18
crispatus	1.3e+07 (2.9e+04, 1.1e+08)	0.0e+00 (0.0e+00, 4.7e+03)	0.0003	8.6e+03 (8.7e+02, 2.3e+07)	0.0e+00 (0.0e+00, 1.4e+03)	0.0066
ctv05	2.5e+06 (7.7e+02, 1.1e+07)	1.3e+02 (1.3e+01, 5.6e+02)	0.024	3.2e+04 (3e+01, 2.4e+06)	8.9e+01 (2e+01, 1.2e+02)	0.21
iners	1.3e+06 (1.5e+04, 3.1e+07)	8.6e+07 (2.5e+07, 4.6e+08)	0.011	3.1e+06 (6.3e+03, 2.2e+07)	3.4e+07 (1.8e+07, 8.5e+07)	0.0063

[1]copies/swab

[2]Median (IQR)

[3]Wilcoxon rank sum exact test; Wilcoxon rank sum test

10. **Supplemental Table 4:** Comparison of detection of LACTIN-V by qPCR between arms at each visit

**Visit**	**LACTIN-V**, N = 32[1]	**Placebo**, N = 12[1]	**p-value**[2]
pre-MTZ			>0.9
detectable	5 / 32 (16%)	2 / 12 (17%)	
non-detectable	27 / 32 (84%)	10 / 12 (83%)	
post-MTZ			0.60
detectable	3 / 32 (9.4%)	2 / 12 (17%)	
non-detectable	29 / 32 (91%)	10 / 12 (83%)	
Week 4			0.0004
detectable	22 / 32 (69%)	1 / 12 (8.3%)	
non-detectable	10 / 32 (31%)	11 / 12 (92%)	
Week 8			0.033
detectable	15 / 32 (47%)	1 / 11 (9.1%)	
non-detectable	17 / 32 (53%)	10 / 11 (91%)	

[1]n / N (%)

[2]Fisher's exact test; Pearson's Chi-squared test

11. Supplemental Table 5: Median vaginal fluid immune marker concentrations at pre-MTZ and week 4 visits in LACTIN-V and placebo arms for the 6 analytes that were quantifiable in > 65% of samples

Analyte	LACTIN-V			Placebo		
	**pre-MTZ**, N = 31[1]	**Week 4**, N = 31[1]	**p-value**[2]	**pre-MTZ**, N = 12[1]	**Week 4**, N = 12[1]	**p-value**[2]
IL-1α pg/mL	29 (23, 67)	19 (8, 46)	0.039	26 (17, 38)	34 (19, 60)	0.6
IL-8 pg/mL	41 (2, 306)	517 (98, 2,500)	0.008	149 (68, 485)	606 (403, 1,429)	0.064
IP-10 pg/mL	3 (0, 12)	21 (2, 90)	0.003	3 (1, 23)	57 (28, 252)	0.009
MIG pg/mL	8 (2, 34)	44 (18, 340)	<0.001	40 (2, 105)	330 (52, 686)	0.016
IL-1β pg/mL	2 (0, 11)	2 (0, 8)	0.5	4.1 (0.7, 9.3)	1.5 (0.6, 3.2)	0.4
IL-6 pg/mL	0.1 (0.1, 1.3)	0.8 (0.2, 5.0)	0.024	0.5 (0.2, 4.8)	1.5 (0.6, 3.6)	0.9

[1]Median (IQR)

[2]Wilcoxon signed rank exact test; Wilcoxon signed rank test with continuity correction

12. **Supplemental Table 6:** Median vaginal fluid immune marker concentrations at pre-MTZ and week 8 visits in LACTIN-V and placebo arms for the 6 analytes that were quantifiable in > 65% of samples

Analyte	LACTIN-V			Placebo		
	**pre-MTZ**, N = 30[1]	**Week 8**, N = 30[1]	**p-value**[2]	**pre-MTZ**, N = 11[1]	**Week 8**, N = 11[1]	**p-value**[2]
IL-1α pg/mL	33 (23, 71)	27 (11, 103)	0.7	27 (21, 40)	33 (14, 71)	0.6
IL-8 pg/mL	45 (3, 307)	427 (159, 946)	0.020	190 (60, 598)	154 (53, 1,650)	0.3
IP-10 pg/mL	3 (0, 14)	25 (8, 174)	<0.001	3 (1, 30)	34 (1, 107)	0.3
MIG pg/mL	8 (2, 35)	122 (32, 538)	0.002	71 (3, 108)	68 (7, 325)	0.5
IL-1β pg/mL	3 (0, 11)	1 (0, 8)	0.4	4 (1, 7)	1 (0, 15)	0.7
IL-6 pg/mL	0.09 (0.09, 1.30)	0.87 (0.09, 2.42)	0.12	0.5 (0.1, 4.3)	0.3 (0.1, 0.6)	0.6

[1]Median (IQR)

[2]Wilcoxon signed rank exact test; Wilcoxon signed rank test with continuity correction

13. **Supplemental Table 7:** Proportion of samples with detectable values at pre-MTZ and week 4 visits for the 7 vaginal fluid analytes that were not quantifiable in > 35% of participants

**Analyte**	**LACTIN-V**, N = 32[1]	**Placebo**, N = 12[1]
ITAC		
Detectable	11 / 32 (34%)	6 / 12 (50%)
Undetectable	21 / 32 (66%)	6 / 12 (50%)
IFN-γ		
Detectable	10 / 32 (31%)	3 / 12 (25%)
Undetectable	22 / 32 (69%)	9 / 12 (75%)
MIP-3α		
Detectable	12 / 32 (38%)	7 / 12 (58%)
Undetectable	20 / 32 (63%)	5 / 12 (42%)
IL-21		
Detectable	12 / 32 (38%)	5 / 12 (42%)
Undetectable	20 / 32 (63%)	7 / 12 (58%)
IL-23		
Detectable	9 / 32 (28%)	3 / 12 (25%)
Undetectable	23 / 32 (72%)	9 / 12 (75%)
MIP-1α		
Detectable	13 / 32 (41%)	5 / 12 (42%)
Undetectable	19 / 32 (59%)	7 / 12 (58%)
TNFα		
Detectable	10 / 32 (31%)	3 / 12 (25%)
Undetectable	22 / 32 (69%)	9 / 12 (75%)

[1]n / N (%)

14. **Supplemental Table 8**: Proportion of samples with detectable values at pre-MTZ and week 8 visits for the 7 vaginal fluid analytes that were not quantifiable in > 35% of participants

**Analyte**	**LACTIN-V**, N = 31[1]	**Placebo**, N = 11[1]
ITAC		
Detectable	10 / 31 (32%)	4 / 11 (36%)
Undetectable	21 / 31 (68%)	7 / 11 (64%)
IFN-γ		
Detectable	7 / 31 (23%)	5 / 11 (45%)
Undetectable	24 / 31 (77%)	6 / 11 (55%)
MIP-3α		
Detectable	13 / 31 (42%)	5 / 11 (45%)
Undetectable	18 / 31 (58%)	6 / 11 (55%)
IL-21		
Detectable	6 / 31 (19%)	3 / 11 (27%)
Undetectable	25 / 31 (81%)	8 / 11 (73%)
IL-23		
Detectable	8 / 31 (26%)	1 / 11 (9.1%)
Undetectable	23 / 31 (74%)	10 / 11 (91%)
MIP-1α		
Detectable	8 / 31 (26%)	3 / 11 (27%)
Undetectable	23 / 31 (74%)	8 / 11 (73%)
TNFα		
Detectable	6 / 31 (19%)	1 / 11 (9.1%)
Undetectable	25 / 31 (81%)	10 / 11 (91%)

[1]n / N (%)

15. SUPPLEMENTAL METHODS

15.1. Complete list of eligibility criteria

Inclusion Criteria: Participants must have met all of the inclusion criteria in order to be eligible to participate in the study:

- FRESH study participant. To be eligible for participation in FRESH (which was a requirement for eligibility for this trial) participants had to be sexually active, without anemia, and without other barriers to regular study participation, such as serious chronic illness, enrollment in other studies, or conflicting family responsibilities.
- Capable of reading and writing English or isiZulu and voluntarily provide written informed consent to participate in the study and comply with all study procedures.
- HIV-negative.
- Nugent score 4-10 on vaginal Gram stain.
- Otherwise healthy women, 18-23 years of age on the day of enrollment.
- Regular predictable menstrual cycles or amenorrhoeic for at least 3 months due to use of a long-acting progestin.
- Willing to complete 7-day course of oral metronidazole.
- Willing to be asked questions about personal medical health and sexual history.
- Willing to apply study agent vaginally and comply with study examinations.
- Willing to self-administer Study Product on dosing days that do not coincide with regular FRESH study visits.
- Agree to try to abstain from sexual intercourse 12 hours prior to study visits that include a gynaecological exam (Randomization Visit 3, Follow-up Visit 11, Final Visit 19).

- Agree to try to abstain from sexual intercourse for 12 hours after study product administration to ensure that the product will remain inside the vagina.
- Agree to abstain from the use of any other vaginal product throughout the trial period from the time of enrollment through the end of the study. (*Note: Intravaginal products include contraceptive creams such as Gynol II, gels, foams, sponges, lubricants not approved by the study investigators, tampons and douches.)*
- Must be stable on a reliable method of long-acting birth control and agree to remain on, for the duration of the study (if of childbearing potential) or, of nonchildbearing potential (permanently sterile).

Exclusion Criteria: Participants meeting any of the following criteria when assessed at the Enrollment Visit, were excluded from the study:

- Urogenital infection (as tested during the FRESH Week 5 Study Visit, reported within 30 days of detection at the LACTIN-V Enrollment Visit). *Note: Urogenital infection includes urinary tract infection, Trichomonas (T.) vaginalis, Neisseria (N.) gonorrhoeae, Chlamydia (C.) trachomatis, Mycoplasma genitalium.*
- Diagnosis of two or more outbreaks of *N. gonorrhoeae, C. trachomatis, T. vaginalis, Mycoplasma genitalium*, or herpes simplex virus (herpes genitalis) within 6 months prior to enrollment.
- Subject is ineligible if menstrual cycle length is less than 21 days.
- Subject is ineligible if deep epithelial disruption is observed on genital examination noted on or before the Randomization Visit.

- Positive for HIV (as tested during the FRESH Week 5 Study Visit, within 30 days of the LACTIN-V Enrollment Visit).
- Current pregnancy or within 2 months of last pregnancy.
- Vaginal or systemic antibiotic or antifungal therapy within 21 days of enrollment.
- Use of disulfiram within past 2 weeks or other contraindication to use of metronidazole.
- Any condition requiring regular periodic use of systemic antibiotics during participation in the trial.
- Investigational drug use other than LACTIN-V within 30 days or 10 half- lives of the drug, whichever is longer, of Enrollment Visit.
- Other planned participation in an investigational drug study while participating in this study.
- IUD insertion or removal, pelvic surgery, cervical cryotherapy or cervical laser treatment within the last 2 months prior to enrollment.
- Use of vaginal ring (e.g, NuvaRing) within 3 days of enrollment or during the course of the study.
- Hysterectomy.
- Unwilling to complete 7 days of oral metronidazole (twice daily) with the last dose taken no later than 48 hours prior to randomization (minimum of 12 of 14 doses required).
- Use of new long-acting hormonal treatments. Participant may be enrolled if stable (at least 1 month) on existing therapy as determined by the principal investigator (PI).
- Known allergy to any component of LACTIN-V/placebo or metronidazole or to nitroimidazole derivatives or latex (condoms).

- Any social, medical, or psychiatric condition including history of drug or alcohol abuse that in the opinion of the investigator would make it difficult for the participant to comply with study procedures.
- Any serious or chronic illness, deemed incompatible with study participation by the study doctor, including immunosuppression due to cancer chemotherapy, systemic corticosteroids.

15.2. Product Administration Error

In December 2022, one of the trial pharmacists noticed that nine participants had recently received 1 or 2 applicators retrieved by new staff from incorrect non-assigned kits stored in the research pharmacy instead of from the assigned kits stored in the clinical examination room refrigerator. After unblinding of the database after the end of the trial, it was identified that 3 participant(s) in the LACTIN-V group received 1 or 2 applicators containing placebo, and 1 participant in the placebo group received a single applicator containing LACTIN-V. All 5 other participants had received dosing that was identical to their assigned group. After discussion among the investigators and data safety monitoring board, we decided to exclude the one participant in the placebo group who erroneously received LACTIN-V was excluded from the modified intention-to-treat (ITT) population used to determine the effect of LACTIN-V. To ascertain safety, all participants were included in the ITT population.

15.3. Laboratory Methods

Cervicovaginal Lavage Collection and Processing

Cervicovaginal lavage (CVL) samples were obtained by rinsing the endocervix with 5mL of sterile saline. Pooled samples were collected from the posterior fornix region into a 15mL conical. CVLs were transported on wet ice to the laboratory and were centrifuged at 1700 rpm for 10 min to fractionate the supernatant from the cellular components. 1mL aliquots of the supernatant were stored at -80C. 200uL of thawed CVL samples were aliquoted in 96-well 0.22um filtering microplates and centrifuged at 4ºC, 1000 xg for 20 min for use in the Luminex assays.

Luminex

Concentrations of 20 cytokines/chemokines were measured in undiluted cervicovaginal lavage fluid using a Custom Milliplex High Sensitivity 20-Plex Luminex Kit (EMD Millipore). The analyte panel included C-X-C motif chemokine 10 (CXCL10; also known as IP-10), interleukin (IL)-8, IL-6, MIG, ITAC, IL-1a, IL-1b, MIP-1a, MIP-1b, MIP-3a, TNFa, IL-21, IL-17, IFNg, IL-23, IL-12 (p70), IL-13, IL-10, IL-4, and IL-5. Samples were assayed on 96-well plates that contained two quality controls and eight concentration standards in duplicate. Two biological controls were assayed on all plates to monitor interplate variation. Equal volumes of sample, assay buffer, and 1:2 diluted magnetic bead mix were aliquoted onto the plate and incubated with agitation at 300 rpm for 16-18h at 4C in the dark. After the overnight incubation, the samples were washed three times with 1X Wash Buffer using the magnetic Bio-Plex Pro wash station to remove excess reagent and unbound sample. The samples were then labeled with 1:2 diluted detection antibodies and incubated for 1 hour at room temperature with agitation at 300 rpm in the dark.

Next, the samples were stained with 1:2 diluted Streptavidin PE and incubated at room temperature for 30 minutes with agitation at 300 rpm in the dark. The samples were again washed three times with 1X wash buffer using the magnetic Bio-Plex Pro wash station to remove excess staining reagents. 150uL of sheath fluid was added to all plates well after the final wash and agitated at 300 rpm for 2 minutes. All samples were run on the Bio-Plex-200 and cytokine concentration data was acquired using the Bio-Plex Manager software.

Cytokine Analysis

Bead counts were used to standardize the quality of the experimental runs as low bead counts are most often indicative of sample processing or instrumental complications not biological differences. If samples resulted in bead counts below 30 for analytes of interest, the samples were re-run on an additional plate. The highest lower limit of detection from the Bio-Plex Manager software across all sample plates was used for analysis. From the original panel of 20 cytokines/chemokines, seven analytes (MIP 1-b, IL-10, IL-4, IL-13, Il-12 (p70), IL-17, IL-5) were removed due to >= 80% of samples having concentration values outside the limits of detection. Mann-Whitney U tests were used to identify significant differences in log fold changes across treatment arms.

Total Nucleic Acid Extraction

Total nucleic acids (TNA) from cervicovaginal swabs were extracted using a phenol-chloroform extraction method, which includes a bead beating process to disrupt the bacteria [1,2] with a modification for 96-well plate. The "dry" swabs stored at -80C were transferred onto ice. A volume of 750uL of lysis buffer (sodium chloride (200mM), Tris-HCl (200mM), EDTA (20 mM) in

ultrapure H_2O) was added to the cryovial containing swab, vortexed vigorously, and left on ice. An aliquot of 200uL of the sample solution was transferred to cluster tubes containing sterile glass beads (200mg), phenol chloroform (200uL) and 20% SDS (80uL) and homogenized at 30Hz for 2 minutes on the TisssueLyserII (Eppendorf). Samples were then centrifuged at 2800xg for 15 minutes at 4C and the top aqueous phase (250-300uL) was transferred to a 96 well "S-block" on ice. Sodium acetate (approximately 0.08 vol of the aqueous phase) and -20C isopropanol (approximately 0.8 vol of the aqueous phase) were added to precipitate sample overnight. S-Block was centrifuged at 2800xg for 30 minutes at 4C. The supernatant was removed and the TNA pellet was washed with 300uL of 100% ethanol. After centrifuge at 2800xg for 15 minutes at 4C, ethanol was removed and the S-block left open to dry at room temperature. The TNA pellet was resuspended in 85uL of TE Buffer and aliquots were stored at -20C.

PCR amplification and sequencing of the 16S rRNA gene

Bacterial taxonomic compositions in the cervicovaginal samples were determined through sequencing the V4 region of 16S rRNA gene. This region was amplified using the primer set 515F/806R (with 806R barcoded for multiplexing) in a 25 ul reaction containing 1X Q5 reaction buffer (NEB), 0.2 mM of dNTPs, 0.2 uM of each primer, 0.5 unit of Q5 high-fidelity DNA polymerase (NEB), and 2 ul of TNA sample. PCR was performed in triplicate for each sample at 98°C for 30 s, followed by 30 cycles of 98°C for 10s, 60°C for 30s, and 72°C for 20s, with a final extension at 72°C for 2min. To monitor contamination, negative controls with water as template, in parallel to TNA samples in each barcode master mix, were also PCR amplified to confirm no amplification. Triplicate PCRs for each sample were combined and checked on an

agarose gel. PCR products of samples were pooled based on gel band strength, purified with QIAquick PCR purification kit, and sequenced on Illumina MiSeq with a 300-cycle kit. Negative controls for TNA extraction and PCR were included in the sequencing.

16S Data Processing

Denoising and removal of sequencing errors from the Illumina amplicon reads was conducted using DADA2. Taxonomic assignments were made with Genome Taxonomy Database (GTDB) version "X" and curated taxonomy from prior vaginal microbiome studies (CITE).

Cytobrush Collection and Processing

Cytobrushes were inserted into the endocervical canal and rotated 360 degrees. Samples were then placed in a 15mL conical containing 5mL of R10 collection media. The samples were transported on wet ice to the laboratory where the mucus was loosely dislodged by manual agitation and subsequently mixed with additional R10 media. Sample solutions were centrifuged at 4ºC at 300g for 10 minutes, and the supernatants were discarded and the pellets resuspended in R10.

Immunophenotyping of cervical cytobrush samples

Flow cytometry of cytobrush cells was acquired within the same day of collection. Samples were stained with fluorescently-labeled monoclonal antibodies for the following surface markers: HLA-DR, CD8, CD45, EpCAM, CD4, CCR5, CD14, CD19, CD38, CD11c, and CD66b. The following antibodies were used for cell staining and sorting: CD8 (BD Biosciences-560179), CD45 (BD Biosciences-560566), CD14 (BD Biosciences-561391), CD4 (BD Biosciences-563877), CCR5

(BD Biosciences-563379), Epcam (Biolegend-324208), CD19 (BD Biosciences-562440), CD66B (Biolegend-305106), CD3 (BD Biosciences-562280), Brilliant stain buffer (Fisher Scientific-563794), CD38 (BD Biosciences-335790), CD11c (BD Biosciences-551077) , HLADR (BD Biosciences-555811), BD CompBeads Negative Control (BD Biosciences-51-90-9001291) , BD CompBeads Positive Control (BD Biosciences-51-90-9001229) , Rainbow Beads (BD Biosciences-556298). Sample acquiring and sorting of cells were conducted on the BD Aria Fusion cell sorter (BD Biosciences, Woburn MA, USA) and FMO controls were used to define gates for activation markers. Sample data was analyzed using FlowJo Version 10.9.0 (FlowJo Enterprise).

Quantitative PCR

The qPCR assay targeting *L. crispatus* and *L.iners* bacteria were performed as described previously [3,4] Each reaction was performed in a final volume of 20 uL containing 10ul of TaqMan™ Fast Advanced Master Mix (Thermofisher Scientific) 5 µl of DNA template with the final reaction containing 0.8 µM of each forward and reverse primer, 150 nM of the TaqMan probe. The BactiQuant qPCR assay measuring the total bacterial abundance was performed as described previously.[5] Each reaction was performed in a final volume of 20 uL containing 10ul of TaqMan™ Fast Advanced Master Mix (Thermofisher Scientific) 5 µl of DNA template with the final reaction containing 1.8 µM of each forward and reverse primer, 225nM the TaqMan probe. Calibration curves were done using accurately quantified plasmid standards containing the 16 S rRNA gene DNA segment amplify in the qPCR reaction. The *L. iners* plasmid standard was used for the BactiQuant assay. The qPCR assay targeting *L. crispatus* CTV-05 strain was performed in a final volume of 20 uL containing 10ul of iTaq™ Universal SYBR® Green Supermix

(Qiagen) and 5 µl of DNA template, with the final reaction containing 0.5 µM of each forward and reverse primer. The *L. crispatus* CTV-05 strain primers sequences are for the forward primer GCTGTTGCAGCCAGACAGTT and for the reverse primer TCTCTGGGACATCCATAAGTTG.[6] Following initial denaturation at 95°C for 15 minutes, PCR cycling consisted of 40 cycles at 95°C for 30 seconds, 58°C for 60 seconds, and 72°C for 60 seconds. The calibration curve was created using genomic DNA extracted from the CTV-05 strain using the DNA easy UltraClean Microbial Kit (Quiagen). The QuantStudio™ 7 Flex System (Thermo Fisher Scientific) was used to perform all the qPCR reactions.

References

1. Anahtar MN, Bowman BA, Kwon DS. Efficient Nucleic Acid Extraction and 16S rRNA Gene Sequencing for Bacterial Community Characterization. *J Vis Exp* 2016; (110).
2. Bloom SM, Mafunda NA, Woolston BM, et al. Cysteine dependence in Lactobacillus iners constitutes a novel therapeutic target to modify the vaginal microbiota. *bioRxiv* 2021: 2021.06.12.448098.
3. Fredricks DN, Fiedler TL, Thomas KK, Mitchell CM, Marrazzo JM. Changes in Vaginal Bacterial Concentrations with Intravaginal Metronidazole Therapy for Bacterial Vaginosis as Assessed by Quantitative PCR. *Journal of clinical microbiology* 2009.
4. Srinivasan S, Liu C, Mitchell CM, et al. Temporal variability of human vaginal bacteria and relationship with bacterial vaginosis. *PLoS One* 2010; **5**(4): e10197.
5. Liu CM, Aziz M, Kachur S, et al. BactQuant: an enhanced broad-coverage bacterial quantitative real-time PCR assay. *BMC microbiology* 2012; **12**: 56.
6. Cohen CR, Wierzbicki MR, French AL, et al. Randomized Trial of Lactin-V to Prevent Recurrence of Bacterial Vaginosis. *The New England journal of medicine* 2020; **382**(20): 1906-15.

Phase 2 placebo-controlled randomized trial of LACTIN-V (*Lactobacillus crispatus* CTV-05) among women at high risk of HIV acquisition in Durban, South Africa

Funding Mechanism: NICHD Grant 1R01HD098978

Pharmaceutical Support Provided by: Osel, Inc.

Trial Sponsor: University of California, San Francisco (USA)

South Africa

Co- Principal Investigator: Vaneshree Govender, MBBCh

Co- Principal Investigator: Thumbi Ndung'u, BVM, PhD

International

Principal Investigator: Craig R. Cohen, MD, MPH

Co- Principal Investigator: Douglas S. Kwon, MD, PhD

Co- Principal Investigator: Krista Dong, MD

Protocol Number: LV-007

Version 4.3

03 September 2021

STATEMENT OF COMPLIANCE

The study will be carried out in accordance with Good Clinical Practice (GCP) as required by the following

- United States (US) Code of Federal Regulations (CFR) applicable to clinical studies (45 CFR Part 46; 21 CFR Part 50, 21 CFR Part 56, and 21 CFR Part 312)
- ICH E6; 62 Federal Register 25691 (1997)
- NIH Clinical Terms of Award
- Guidelines for Good Practice in the Conduct of Clinical Trials with Human Participants in South Africa. Department of Health, 2006.

All key personnel (all individuals responsible for the design and conduct of this study) have completed Human Subjects Protection Training.

SIGNATURE PAGE

The signature below constitutes the approval of this protocol and the attachments, and provides the necessary assurances that this trial will be conducted according to all stipulations of the protocol, including all statements regarding confidentiality, and according to local legal and regulatory requirements and applicable US federal regulations and ICH guidelines.

Co-Principal Investigator (SOUTH AFRICA):

Signed: Date: 03 September, 2021

Vaneshree Govender, MBBCh

Co-Principal Investigator (SOUTH AFRICA):

Signed: Date: 03 September, 2021

Thumbi Ndung'u, BVM, PhD

Principal Investigator (USA):

Signed: Date: 03 September, 2021

Craig R. Cohen, MD, MPH

Co-Principal Investigator (USA):

Signed: Date: 03 September, 2021

Doug S. Kwon, MD, Ph D

Co-Principal Investigator (USA):

Signed: Date: 03 September, 2021

Krista Dong, MD

TABLE OF CONTENTS

STATEMENT OF COMPLIANCE 2

SIGNATURE PAGE 3

TABLE OF CONTENTS 4

LIST OF ABBREVIATIONS 7

PROTOCOL SUMMARY 9

SCHEMATIC OF STUDY DESIGN: 10

1.0 KEY ROLES 11
Key Investigators 11
Others 13

2.0 BACKGROUND INFORMATION AND SCIENTIFIC RATIONALE 14
2.1 Background Information the vaginal microbiome and its influence on risk of HIV infection/transmission 14
2.2 Background Information on Bacterial Vaginosis 16
2.3 Preliminary Studies on increased HIV acquisition and mucosal inflammation in young South African women 16
2.4 Preliminary Studies on LACTIN-V for Bacterial Vaginosis 18
2.5 Rationale 21
2.6 Potential Risks and Benefits 23

3.0 HYPOTHESIS AND OBJECTIVES 27
3.1 Study Hypothesis 27
3.2 Summary of Methods 27
3.3 Study Objectives 27
3.4 Study Outcome Measures 27

4.0 STUDY DESIGN 30

5.0 STUDY ENROLMENT AND WITHDRAWAL 34
5.1 Subject Inclusion Criteria 34
5.2 Subject Exclusion Criteria 34
5.3 Treatment Assignment Procedures 36

6.0 STUDY PRODUCT 39
6.1 Study Product Description 39
6.2 Dosage, Preparation, and Administration of Study Product 41
6.3 Accountability Procedures for the Study Investigational Product 41
6.4 Assessment of Subject Compliance with Study Product 42
6.5 Concomitant Medications/Treatments 42

7.0 STUDY SCHEDULE 43
7.1 Enrolment Visit 1 43
7.2 Check-in Visit 2 (Day 4) 45

7.3 Randomization Visit 3 (Day 8) 45
7.4 Check-In Visits 4 – 10 47
7.5 Follow-up Visit 11 (Day 36) (Allowable window: Days 33- 43) 48
7.6 Check-In Visits 12 –18 50
7.7 Final Study Visit 19, Day 64 (Allowable window: Days 57 - 71) 50
7.8 Study Product Discontinuation/Early Termination Visit 52
7.9 Unscheduled Visit 53

8.0 STUDY PROCEDURES/EVALUATIONS 55
8.1 Clinical Evaluations 55
8.2 Laboratory Evaluations 55

9.0 ASSESSMENT OF SAFETY 57
9.1 Specification of Safety Parameters 57
9.1 Methods and Timing for Assessing, Recording, and Analyzing Safety Parameters 57
9.3 Reporting Procedures 59
9.4 Type and Duration of Follow-up of Subjects after Adverse Events 61
9.5 Halting Rules 61
9.6 Safety Oversight (DSMB) 61

10.0 MONITORING 63
10.1 Site Monitoring Plan 63

11.0 STATISTICAL CONSIDERATIONS 64
11.1 Introduction 64
11.2 Study Objectives and Outcome Measures 64
11.3 Sample Size Considerations 65
11.4 Safety Analysis 65
11.5 Statistical Analysis Plan 66

12.0 SOURCE DOCUMENTS AND ACCESS TO SOURCE DATA/ DOCUMENTS 68

13.0 QUALITY CONTROL AND QUALITY ASSURANCE 69

14.0 ETHICS/PROTECTION OF HUMAN SUBJECTS 70
14.1 Ethical Standard 70
14.2 Institutional Review Board 70
14.3 Informed Consent Process 70
14.4 Exclusion of Women, Minorities, and Children (Special Populations) 71
14.5 Subject Confidentiality 71
14.6 Study Discontinuation 71

15.0 DATA HANDLING AND RECORD KEEPING 72
15.1 Data Management Responsibilities 72
15.2 Data Capture Methods 72
15.3 Types of Data 72
15.4 Timing/Reports 73
15.5 Study Records Retention 73

15.6 Protocol Deviations 73

16.0 PUBLICATION POLICY 74

17.0 SPONSOR INDEMNIFICATION FOR SITES AND INVESTIGATORS 75

18.0 LITERATURE REFERENCES 76

19.0 APPENDICES 81

Appendix A Schedule of Events

Appendix B Division of AIDS Table for Grading the Severity of Adult and Pediatric Adverse Events, December 2004; Addendum 1: Female Genital Grading Table for Use in Microbicide Studies, November 2007

Appendix C DAIDS Toxicity Table for Grading the Severity of Adult and Pediatric Adverse Events, Version 2.1-July 2017

Appendix D Coordination between FRESH and LACTIN-V studies

LIST OF ABBREVIATIONS

AE	Adverse Event
ATP	According to Protocol
βHCG	Beta Human Chorionic Gonadotropin
BV	Bacterial Vaginosis
CAPRISA	Centre for the AIDS Programme of Research in South Africa
CAR	Clinical Agents Repository
CC	Complete Case Colony
cfu	Forming Units
CMH	Cochran-Mantel-Haenszel
CDC	Centers for Disease Control and Prevention
CI	Confidence Interval
CONRAD	Contraceptive Research and Development
CRF	Case Report Form
CROMS DAIDS	Clinical Research Operations and Management Support Division of AIDS, NIAID, NIH, DHHS
DHHS DMID	Department of Health and Human Services Division of Microbiology and Infectious Diseases, NIAID, NIH, DHHS
DSMB	Data and Safety Monitoring Board
ELISA	Enzyme-linked Immunosorbent Assay
FDA	Food and Drug Administration
GCP	Good Clinical Practice
GMP	Good Manufacturing Practice Informed
ICF	Consent Form
ICH	International Conference for Harmonisation International
ICMJE	Committee of Medical Journal Editors
IDES	Internet Data Entry System
IEC	Independent or Institutional Ethics Committee
IRB	Institutional Review Board
ISM	Independent Safety Monitor
ITT	Intent-to-treat
LACTIN-V	*Lactobacillus crispatus* CTV-05
LBP	Live Biotherapeutic Product
LOCF	Last Observation Carried Forward
MedDRA	Medical Dictionary for Regulatory Activities
Metronidazole	Metronidazole oral tablets
mITT	Modified Intent-to-Treat
MOP	Manual of Procedures
N	Number (typically refers to participants)
NIAID	National Institute of Allergy and Infectious Diseases, NIH, DHHS
NIH	National Institutes of Health
OCRA	Office of Clinical Research Affairs, DMID, NIAID, NIH, DHHS

OHRP	Office for Human Research Protections
ORA	Office of Regulatory Affairs, DMID, NIAID, NIH, DHHS
OSEL	Office of Science and Engineering Laboratories Potential
pH	Hydrogen
PI	Principal Investigator
PP	Per Protocol
qPCR	Quantitative Polymerase Chain Reaction
RR	Rate Ratio
rUTI	Recurrent Urinary Tract Infection
SAE	Serious Adverse Event
SAHPRA	South African Health Products Regulatory Authority
SDCC	Statistical and Data Coordinating Center
SMC	Safety Monitoring Committee
SOP	Standard Operating Procedure
STAR	Sexually Transmitted Infections Treatment and Research
STI	Sexually Transmitted Infection
STI-CTG	Sexually Transmitted Infections Clinical Trials Group
UCSF	University of California, San Francisco
UKZN	University of KwaZulu-Natal
US	United States
UTI	Urinary Tract Infection
WHO	World Health Organization

PROTOCOL SUMMARY

Title:	Phase 2 placebo-controlled randomized trial of LACTIN-V (*Lactobacillus crispatus* CTV-05) among women at high risk of HIV acquisition in Durban, South Africa
Phase:	2
Population:	Non-pregnant women age 18 – 23.<br>Enrolment will be continued until 60 women are randomized into the study
Number of Sites:	One site in Durban, South Africa:<br>FRESH Clinic: Umlazi, KZN
Study Duration:	90 weeks after enrolment of the first participant
Participant Participation: Duration:	10 weeks (64 days) per participant
Description of Agent:	Lactobacillus *(L.) crispatus* CTV-05 (LACTIN-V) contains a naturally occurring vaginal strain of *L. crispatus* CTV-05, preserved as a powder applied by a vaginal applicator.<br>LACTIN-V at 2×10^9 cfu/dose or matching placebo will be administered by vaginal applicator once daily for 5 consecutive days, followed by twice weekly for 3 consecutive additional weeks.
Primary Objectives:	1: Determine the effect of repeat dosing of LACTIN-V (2×10^9 cfu/dose) on genital tract inflammation in young South African women at risk of HIV.<br>2: Determine the ability of LACTIN-V to promote a *Lactobacillus*-dominant vaginal microbiota in young South African women at risk of HIV.<br>3: Determine the safety and acceptability of LACTIN-V in a population of young South African women at risk for HIV.
Description of Study Design:	See schematic
Time to Complete Enrolment:	65 weeks
Keywords:	Vaginal microbiome, lactobacilli, HIV risk, vaginal mucosa

SCHEMATIC OF STUDY DESIGN:

Enrolment Visit 1
Day 1

Check-In Visit 2
Day 4

Find out if you are eligible for the study:

- Be invited to participate if prior FRESH Lab results (Gram Stain, STI testing) confirm that you are eligible.
- Have physical exam, as well as medical, gynaecological and sexual history, and answer questionnaire.
- Start 7-day course of oral Metronidazole. Check-In Visit 2 on Day 4.

Randomization
Visit 3
Day 8

- Confirm completion of Metronidazole treatment.
- Have physical exam, medical, gynaecological and sexual history, gynaecological exam, vaginal swabs, urine test, pregnancy test.

Be assigned to receive LACTIN-V or placebo (the inactive substance) by randomization (chance)

- Administer first dose at clinic based on randomization. Take 2 doses home to be administered before bed the following two days (Study Day 9 and 10).

LACTIN-V | PLACEBO

Dosing Visits
Visit 4, Day 11
Visit 5, Day 15
Visit 6, Day 18
Visit 7, Day 22
Visit 8, Day 25
Visit 9, Day 29
Visit 10, Day 32

- Answer questions about symptoms, medications, and sexual history
- Administer study product at clinic
- At Visit 4, take 1 dose home to be administered before bed the next day (Study Day 12)

Follow-up Visits
Visit 11, Day 36

- Have physical exam, medical, gynaecological and sexual history, gynaecological exam, vaginal swabs, urine test, pregnancy test

Follow-up Visits
Visit 12, Day 39
Visit 13, Day 43
Visit 14, Day 46
Visit 15, Day 50
Visit 16, Day 53
Visit 17, Day 57
Visit 18, Day 60

- Answer questions about symptoms, medications, and sexual history

Final Visit
Visit 19, Day 64

- Have physical exam, gynaecological medical and sexual history, gynaecological exam, vaginal swabs, urine test, pregnancy test

1.0 KEY ROLES

Key Investigators

SOUTH AFRICA:	
Co-Principal Investigator	Vaneshree Govender, MBBCh<br>FRESH<br>Shop 109A, Umlazi Mega City,<br>50 Griffiths Mxenge Highway<br>Umlazi, 4066<br>South Africa<br>+27 (0) 31 003 5414<br>+27 (0) 84 759 6461 (mobile)<br>The Aurum Institute<br>Department: Research Management<br>Division: Clinical Research<br>29 Queens Road, Parktown,<br>Johannesburg<br>South Africa, 2193<br>+27 (0) 10 590 1300<br>VGovender@auruminstitute.org
Co-Principal Investigator	Thumbi Ndung'u, DVM, PhD<br>HIV Pathogenesis Programme (HPP)<br>Nelson R Mandela School of Medicine<br>University of KwaZulu-Natal<br>719 Umbilo Road<br>Durban<br>South Africa<br>+27 (0) 31 260 4618<br>Ndungu@ukzn.ac.za
Co-Investigator	Siphesihle Reward Ngcobo, MBChB<br>Ekasi Medical Centre<br>Umlazi Mega City,<br>50 Griffiths Mxenge Highway,<br>Umlazi, 4066<br>South Africa<br>+27 (0) 31 902 8808<br>+27 (0) 61 448 6836 (mobile)<br>sihler312@gmail.com
INTERNATIONAL:	
Principal Investigator	Craig Cohen, MD, MPH<br>550 16th Street (Mission Bay Campus)<br>San Francisco, CA 94158<br>(415) 476-5874<br>Craig.Cohen@ucsf.edu
Co- Principal Investigator	Douglas S. Kwon, MD, PhD<br>The Ragon Institute of MGH, MIT and Harvard<br>400 Technology Square 893<br>Cambridge, MA 02139, USA<br>+1 (857) 268-7079<br>dkwon@mgh.harvard.edu

Co-Principal Investigator	Krista Dong, MD<br>The Ragon Institute of MGH, MIT and Harvard / FRESH<br>Shop 109A, Umlazi Mega City,<br>50 Griffiths Mxenge Highway<br>Umlazi, 4066<br>South Africa<br>+27 (0) 31 906 0394<br>woodil.iteach@gmail.com<br>kdong@mgh.harvard.edu
Co- Investigator	Anke Hemmerling, MD, PhD, MPH 550<br>16th Street<br>(Mission Bay Campus, GHS Building)<br>San Francisco, CA 94158<br>+ 1 (415) 476-5878<br>Anke.Hemmerling@ucsf.edu 2
Co- Investigator	Caroline Mitchell, MD, MPH<br>Vincent Center for Reproductive Biology<br>Assistant Professor, Obstetrics, Gynecology & Reproductive Biology<br>caroline.mitchell@mgh.harvard.edu
Protocol Statistician	Musie Ghebremichael, PhD<br>The Ragon Institute of MGH, MIT and Harvard<br>400 Technology Square 870<br>Cambridge, MA 02139<br>+1 (857) 268-7079<br>Musie_Ghebrimichael@dfci.harvard.edu
Co- Investigator	Laurel Lagenaur, PhD<br>Osel, Inc.<br>320 Logue Avenue<br>Mountain View, CA 94043<br>+1 (240) 760-6649<br>llagenaur@oselinc.com
Co- Investigator	Thomas Parks, PhD<br>Osel, Inc.<br>320 Logue Avenue<br>Mountain View, CA 94043<br>+1 (650) 396-7626<br>tparks@oselinc.com

Others

Monitoring	TCD Global<br>Tracy Southwood MD<br>Design House, 121 Amkor Road,<br>Centurion 0157, Gauteng<br>PO Box 15775,<br>Lyttelton 0140, Gauteng, South Africa<br>TEL +27 (0) 31 764 1413<br>FAX +27 (0) 86 668 8657<br>Tracy.Southwood@Tcd-global.com
Regulatory Support	Wilberto Robles<br>Women Care Global<br>701 Palomar Airport Road<br>Suite 300<br>Carlsbad CA<br>92011<br>(858) 314-4025, ext. 300<br>wrobles@wcgcares.org
Data Management	DF Net Research Inc.<br>Darryl Pahl, CEO<br>140 Lakeside Ave, Suite 310<br>Seattle, WA 98122<br>USA<br>+1.206.322.5931 Phone<br>+1.206.322.5932 Fax<br>darryl@dfnetresearch.com<br>www.dfnetresearch.com
Industry Representatives:	
Executive Chairman	Peter Lee, MD Osel,<br>Inc.<br>320 Logue Avenue Mountain<br>View, CA 94043 (650) 964-<br>4679<br>plee@oselinc.com
Director of Product Development	Thomas Parks, PhD<br>Osel, Inc.<br>320 Logue Avenue Mountain<br>View, CA 94043 (650) 964-<br>4679<br>tparks@oselinc.com

2.0 BACKGROUND INFORMATION AND SCIENTIFIC RATIONALE

2.1 Background Information the vaginal microbiome and its influence on risk of HIV infection/transmission

HIV incidence in parts of sub-Saharan Africa (SSA), especially KwaZulu-Natal, South Africa remains very high due to biological, behavioral and unknown factors: SSA is the region most heavily affected by HIV worldwide with 67% of new infections among 15-24 year old women.[1] The glaring geographic and gender disparities in HIV risk have been attributed to multiple causes, including differences in the genital tract immune milieu;[2] hormonally-dependent differences in the genital epithelium;[3] developmental factors; structural factors; and sociocultural factors.[4, 5] The FRESH (Females Rising through Education, Support, and Health) cohort consists of 300 HIV uninfected young women age 18-23 living in Umlazi, South Africa. In this region, <1% of 14 year old girls are HIV positive but over 60% of 24 year old women are HIV infected.[6] The development, evaluation, and testing of safe and highly acceptable female-controlled HIV prevention methods that are effective against HIV and other STIs are an urgent priority. Significant challenges remain for the field, including the reduced efficacy of tenofovir vaginal gel in women with a non-*Lactobacillus*-dominant vaginal microbiota compared to women with a *Lactobacillus*-dominant vaginal microbiota.[7] Current approaches such as vaginal rings, gels and films for HIV prevention face adherence issues and significant obstacles to maintain and finance an uninterrupted large scale distribution chain.[8, 9, 10] A durable, self- renewing intervention such as vaginal live biotherapeutic product (LBP) *Lactobacillus* could avoid some of the persistent obstacles for female-controlled HIV prevention.

Antiretroviral-based prevention strategies demonstrate biologic effectiveness but face challenges with real world efficacy. Oral PrEP with tenofovir or Truvada has been shown to be effective in MSM and HIV sero-discordant couples, and its efficacy is not affected by the vaginal microbiota.[11, 12] Vaginal tenofovir pre-exposure prophylaxis has been shown to reduce the risk of HIV acquisition, among adherent women.[13] However, in cohorts of young women, neither oral nor topical PrEP has demonstrated consistent effectiveness–largely due to low rates of adherence to the intervention.[14, 15, 16] In addition, genital tract inflammation[17] and dysbiotic microbial communities[7] have been associated with attenuation of the protective effect. More recently, the world community has adopted the UNAIDS 90-90-90 platform, advocating for increased testing, treatment and viral suppression as the way to contain the HIV epidemic.[18] However, in a large scale treatment-as-prevention trial conducted in Kenya and Uganda in which 80% of the people living with HIV in the intervention arm were virally suppressed, HIV incidence decreased over three years but remained at 0.7% and was not statistically different in the control communities.[19] Thus, the development of a vaccine and other biomedical prevention products remain a high global priority to end the intractable HIV epidemic, especially among adolescent and young women in sub-Saharan Africa.[20]

The vaginal microbiome influences risk of HIV infection/transmission: Early in the epidemic, bacterial vaginosis (BV) was associated with prevalent HIV[21, 22] and subsequent studies showed associations with incident HIV in women.[23 - 27] BV was also associated with transmission of HIV to the fetus[28] and a four-fold increased risk to male sexual

partners.[29] In a case-control analysis of participants from several studies in eastern and southern Africa, the quantity of several BV-associated microbial taxa (measured by qPCR) was associated with a 2-4 fold increased odds for incident HIV.[30] Using next-generation sequencing techniques, we have demonstrated a 4-fold increased risk for HIV acquisition in women with a diverse, non-*Lactobacillus*-dominant vaginal microbiome (whether or not it meets criteria for BV) compared to a *L. crispatus* dominant cervicotype.[31] These results suggest that *L. crispatus* may significantly reduce the risk of HIV acquisition in women at high risk of infection.

Genital mucosal inflammation increases the risk for HIV acquisition: Among women enrolled in the CAPRISA 004 topical tenofovir PrEP trial, those with genital tract inflammation (defined as ≥3 of 9 cytokines with a concentration in the upper quartile found in vaginal fluid) demonstrated higher HIV incidence and lower efficacy for topical tenofovir (3%) than women without genital inflammation (57% efficacy).[17, 32] Women in the FRESH cohort with diverse vaginal microbial communities, who had the highest risk for HIV acquisition, also had the highest quantities of vaginal proinflammatory cytokines, and the highest numbers of activated cervical CD4+ T cells.[33, 31] These data suggest that the mechanism underlying the association between the female genital microbiota and HIV acquisition is mucosal inflammation; thus a microbial intervention to decrease the risk of female HIV acquisition should decrease mucosal inflammatory markers in the genital tract.

Vaginal colonization with *L. crispatus* reduces the risk of HIV acquisition through several potential mechanisms of action: Although vaginal colonization with H_2O_2-producing *Lactobacillus* species is associated with decreased incidence of HIV [24], not all L*actobacillus* species are equal in this regard. Women with a *L. crispatus* cervicotype had the lowest rates of HIV acquisition, as well as the lowest concentrations of proinflammatory cytokines in vaginal fluid and the lowest numbers of CD4+ HIV-target cells in the endocervix. [33, 31] Unfortunately, only a small fraction of the population had this microbial phenotype (8/94, 9%). The far more prevalent *L. iners* did not confer the same anti-inflammatory effect, nor protection against HIV infection. In the FRESH cohort, when participants transition from a higher CT (more diverse) to a lower CT (less diverse, more *Lactobacillus*-dominant) the concentrations of genital cytokines dropped.[33] Cervicovaginal lavage (CVL) from women with BV has lower SLPI, higher proinflammatory cytokines, and lower anti-HIV activity compared to CVL of women with *Lactobacillus* dominant microbiota.[34] In the presence of the proinflammatory stimulus of BV, colonization with H_2O_2-producing *Lactobacillus* species, like *L. crispatus*, is associated with lower quantities of vaginal cytokines.[35] Vaginal inoculation of gnotobiotic mice with *L. crispatus* is associated with significantly lower numbers

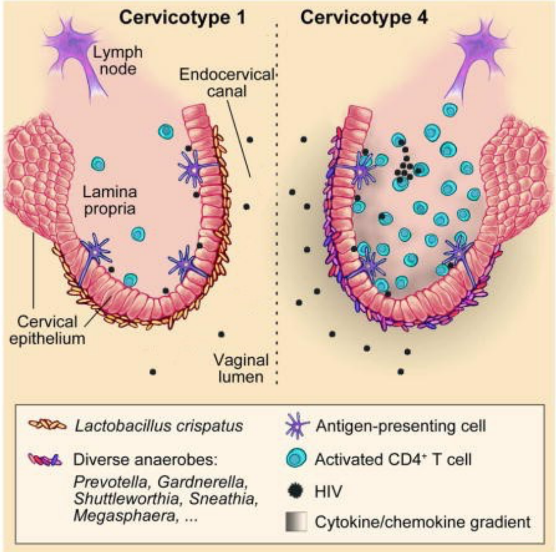

Figure 1: Graphical representation of our hypothesis that *L. crispatus* protects agains HIV acquisition through three complimentary pathways: 1) decreased inflammation in genital mucosa; **2)** reduced number of activated CD4+ T-cells in endocervix; and **3)** reduction of infectious viral particles in genital secretions.

of activated CD4+ T cells in the genital tract compared to *Prevotella bivia*.[33] *In vitro*, several *L. crispatus* strains demonstrated anti-viral effects on HIV, as well as inhibition of viral replication in tissue.[36] Cervicovaginal secretions from women with *L. crispatus* dominant microbiota demonstrate increased HIV-trapping activity compared to *L. iners*-dominated communities.[37] Acidic vaginal secretions characteristic of a *Lactobacillus*-dominant microbial community both trap and inhibit HIV.[38, 39] We will explore these three potential mechanisms of action (Figure 1) in the proposed LACTIN-V clinical trial.

2.2 Background Information on Bacterial Vaginosis

Bacterial vaginosis (BV), characterized by an imbalanced vaginal flora deficient in naturally occurring acid-producing lactobacilli, is one of the most frequent vaginal infections and affects about 15–50% of reproductive aged women globally.[40] Many women are unaware of their condition. BV has been associated with significant gynaecological and obstetric complications, such as pelvic inflammatory disease [41] , endometritis [42] and post-operative infections, including post-cesarean endometritis [43] and post-hysterectomy vaginal cuff cellulitis. [44] Strong associations have also been reported between BV and pre-term delivery, miscarriage [45], and amniotic fluid infections. [46] Studies have linked BV to both female HIV-1 acquisition, and female-to-male HIV transmission.[47-49] A recent study showed that the presence of lactobacilli decrease the odds for fetal inflammatory responses to placental colonization with pathogens. [50] Following standard antibiotic treatment of BV, 20–75% of women relapse within 1–3 months. [51-52] The high risk of recurrence and sequelae suggests that investigational studies of new agents like live biotherapeutic products [53] may be effective for the improved treatment and prevention of BV.

Reconstituting a normal, *Lactobacillus*-predominant microbiome has been promoted for many years as a microbial defense against pathogens. The vaginal live biotherapeutic product *Lactobacillus (L.) crispatus* CTV-05 (LACTIN-V) was developed by Osel, Inc. in Mountain View, California, and is designed to replenish the vaginal lactobacilli population following conventional antibiotic treatment with metronidazole. The product contains a naturally occurring vaginal strain of *L. crispatus* CTV-05, preserved as a powder applied by a vaginal applicator. Since the *Lactobacillus* strain used in LACTIN-V is a commensal organism normally present in the vagina associated and with vaginal health, the product has an excellent pre-clinical and clinical safety profile.

2.3 Preliminary Studies on increased HIV acquisition and mucosal inflammation in young South African women

Lactobacillus-deficient cervicovaginal bacterial communities are associated with increased HIV acquisition and mucosal inflammation in young South African women: We recently characterized cervicovaginal microbiota in a prospective cohort study of healthy HIV-uninfected South African women monitored with high frequency HIV testing. Sequencing of the bacterial 16S rRNA gene revealed the presence of four distinct cervicovaginal bacterial cervicotypes, or “cervicotypes” (CTs), two of which had low diversity and were dominated by either *L. crispatus* (CT1, 11% of participants) or *Lactobacillus iners* (CT2, 32%), and two high diversity CTs dominated by *Gardnerella vaginalis* (CT3, 28%) or a bacterial genus other than

Lactobacillus or *Gardnerella* (CT4, 28%) (Fig. 2A). [31] The most abundant taxa in CT4 were anaerobic genera including *Prevotella*, *Gardnerella*, *Sneathia* and *Megasphaera.* Individuals with CT4 communities were at over 4-fold higher risk of acquiring HIV compared to those with *L. crispatus* dominance (Fig. 2B).[7] They further had elevated genital cytokine levels[31] (Fig. 2C) and 17-fold increased numbers of cervical HIV target cells (Fig. 2D) [31], providing a plausible biological mechanism for the observed increase in infection. We identified specific bacterial taxa linked with HIV infection, showing that *Prevotella melaninogenica*,

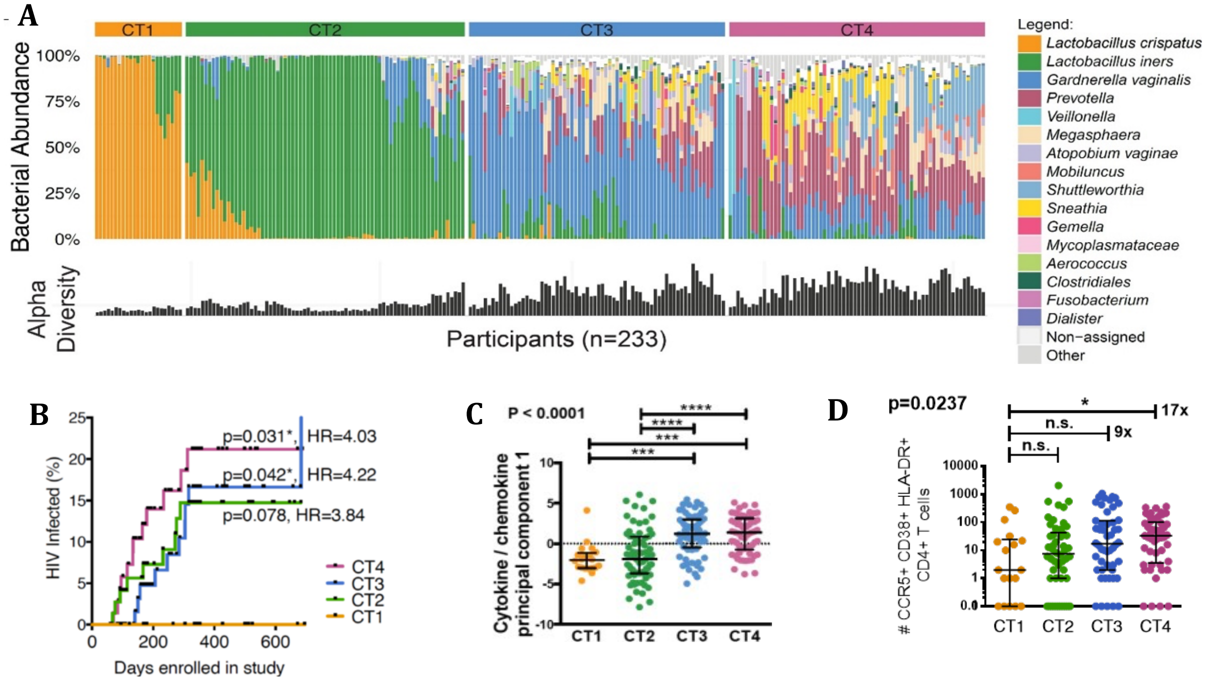

Figure 2: Cervicovaginal microbiome is associated with genital inflammation and increased HIV acquisition. A) Bacterial 16s rRNA gene sequencing reveals 4 cervicovaginal community types ("CTs") in women in FRESH. **B)** Kaplan-Meier curve showing HIV infections in each CT group over time. Acquisition curves for CT2 (n=65 individuals), CT3 (n=57) and CT4 (n=54) were compared with the acquisition curve for CT1 (n=23). Log-rank (Mantel-Cox) test-based p values and Mantel-Haenszel hazard ratios (HR) are displayed. **C)** Principal component 1 (PC1) of 28 measured cytokines shows increased soluble markers of genital inflammation in women with more diverse cervicovaginal bacterial communities. **D)** Flow cytometry analysis of HIV target cell numbers in in cytobrushes from 169 individuals, grouped by CT. Data are summarized as median and IQR. Kruskal-Wallis test with Dunn's post hoc analyses (*$p<0.05$).

Prevotella bivia and *Sneathia sanguinegens*, among other anaerobes, were significantly more abundant in individuals who subsequently acquired HIV. [31] This association was significant in women without co-infection with STIs, confirming a clear association between bacteria and both inflammation and HIV acquisition.

Using cytobrush samples from the FRESH cohort we demonstrated that women with a more diverse, non-*Lactobacillus* dominant microbiome (CT4) had significantly higher numbers of HIV target cells. In addition, these women had increased concentrations of Th17 cytokines, suggesting higher numbers of Th17+ T cells. [31] Women with the highest levels of vaginal proinflammatory cytokines had higher number of activated (CD38+ HLA- DR+) CCR5+CD4+ T cells vs. women with the lowest levels ($p = 0.0361$) (Fig. 3). Antigen presenting cells from women with CT4 had significant upregulation of multiple proinflammatory cytokine gene

such as *IL1A* (5.8-fold), *IL1B* (4.3-fold), *TNF* (6.5-fold), *IL10* (10.6-fold), *IFNB1* (23.4-fold), *IL23A* (7.3-fold), and *IL6* (8.9-fold) vs. CT1 or CT2. [33] None of these difference in subset numbers or transcription were seen when comparing immune cells from peripheral blood.

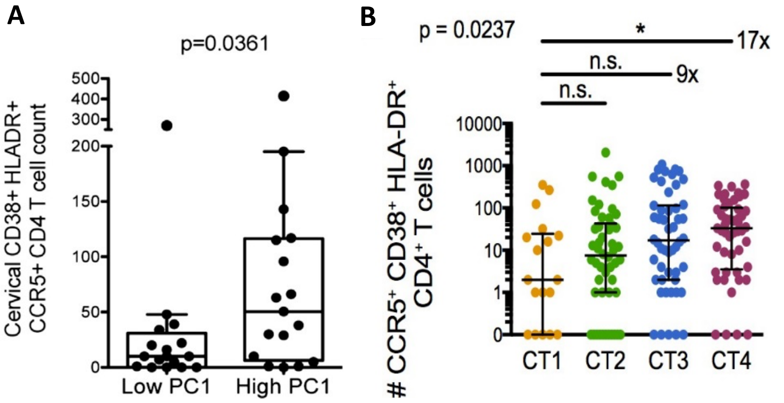

Figure 3. Women with less genital inflammation or with *L. crispatus* dominant microbiota have lower numbers of cervical HIV target cells. **A.** Women in FRESH cohort with the lowest quintile of vaginal cytokines had fewer activated cervical CD4+ T cells than women in the highest quintile of inflammation. **B.** Women with *L. crispatus*-dominant microbiota (CT1) had 17-fold lower number of activated CD4+ T cells vs. those with a diverse vaginal microbial community (CT4)

In the completed Centre for the AIDS Programme of Research in South Africa **(CAPRISA)-004 trial** that tested vaginally delivered 1% tenofovir gel for HIV-1 prevention [55], participants with recurrent symptoms of vaginal irritation and inflammation had a higher risk of HIV-1 acquisition even in the 1% tenofovir gel arm (Karim SA, personal communication). In addition, a strong link has been shown between high-diversity cervicovaginal microbiota with low *Lactobacillus* levels and genital inflammation in a study of South African women. This genital inflammation was accompanied by increased numbers of activated HIV-infectable CD4+ cells in the cervix, providing a potential cellular link to increased HIV acquisition risk. [56] Thus, normalization of vaginal flora through use of exogenous vaginal *Lactobacillus crispatus*, and subsequent reduction of inflammation in the genital tract, could potentially enhance HIV-1 prevention associated with tenofovir gel use and other topical pre-exposure prophylaxis therapies under development. [57]

2.4 Preliminary Studies on LACTIN-V for Bacterial Vaginosis

LACTIN-V contains *Lactobacillus crispatus* CTV-05, a strain of *L. crispatus*, a gram- positive rod isolated from the vagina of a healthy woman. *L.crispatus* is found naturally in the vaginas of healthy women and is commonly found as a component of the natural human intestinal flora. It is a facultative anaerobe, homofermentor of lactic acid, fastidious in its growth, and capable of H_2O_2 production. Unlike most commercially available strains of *Lactobacillus*, CTV-05 adheres well to vaginal epithelial cells and is capable of colonizing the vaginal epithelium.

Early studies of LACTIN-V administered the product in gelatin-coated capsules. LACTIN-V administered as a capsule has been tested in four trials (LV 001-004) with a dose level up to 5 x 10^8 cfu/capsule. After the completion of a Phase 1 safety trial testing low doses of 10^6 and 10^8 cfu/dose or placebo, a Phase 2 multisite placebo-controlled trial tested a dose of 5 x 10^8 cfu of LACTIN-V administered in a gelatin capsule in 149 women with BV following standard antibiotic treatment.

The product was dosed for 5 consecutive days followed by a weekly dose over 10 additional weeks. After 4 months, colonization efficiency in the LACTIN-V group reached 42% in the intent-to-treat (ITT) cohort and 59% in the according-to- protocol (ATP) cohort. Although not statistically significant, the time to first BV recurrence was longer in the LACTIN-V arm (118.7 days) compared to the placebo arm (98.7 days, p = 0.37). The proportion of women in the LACTIN-V treatment group successfully colonized with *L. crispatus* CTV-05 experienced fewer episodes of recurrent symptomatic BV (12.5%) than those who were not colonized (36%), or those in the placebo group (30%). The product had a good safety profile with mostly mild (Grade 1 severity) adverse events (AEs) including vaginal discharge, odor and pruritus, each affecting less than 40% of participants. AEs were evenly distributed between LACTIN-V and placebo arms (Osel, personal communication).

LACTIN-V has also been evaluated as a therapeutic agent to prevent recurrent urinary tract infections (rUTI). In a Phase 2 study in 100 women who received antibiotic treatment for cystitis, those who additionally received 10^8 cfu of LACTIN-V administered in a gelatin capsule over 5 consecutive days followed by a weekly dose over 10 additional weeks had a reduction in rUTI from 27% in the placebo arm to 15% in the LACTIN-V arm (response rate [RR] = 0.5, 95% confidence interval [CI] 0.2-1.2) in comparison to the placebo arm. Among women receiving LACTIN-V who had detected high levels of vaginal colonization with *L. crispatu*s, the reduction of rUTI reached statistical significance (RR=0.07, 95% CI 0.02-0.3). Interestingly, high levels of pre- existing endogenous *L. crispatus* strains in women receiving placebo did not provide protection against rUTI. [58]

Later studies of LACTIN-V administered the product in pre-filled vaginal applicators. Since the dose of 5 x 10^8 cfu/capsule resulted in colonization rates lower than desired, several changes to the study product were made. The higher dose of 2 x 10^9 cfu/dose delivered via vaginal applicator as dried powder directly into the upper vaginal vault, without the impediment of a gelatin capsule, which was found to dissolve slowly in the vaginal environment.

Under BB-IND 11363 (NCT00635622), the new dosage form was first studied in a **small Phase 1 (LV-005)** escalating dose trial at the University of California, San Francisco (UCSF) to assess safety, tolerability and acceptability of three doses [5 X 10^8 cfu/dose (150mg), 1 X 10^9 cfu/dose (300mg), 2 x 10^9 cfu/dose (600mg)] of LACTIN-V (IND No. 11363). Twelve healthy women were randomized 3:1 to use study product for 5 consecutive days, returned for follow-up on Days 7 and 14, and had phone interviews on Days 2 and 35. The DAIDS Toxicity Table Addendum for Vaginal Microbicide Studies (November 2007)[59] was used to grade severity of genitourinary (GU) abnormalities, and colposcopic findings were assessed following the 2004 WHO/CONRAD manual.[60] All 12 participants took 5 doses and completed study follow-up. Overall, 45 adverse events (AEs) occurred, 31 (69%) of which were GU AEs. GU AEs appeared evenly distributed between the 3 dose levels of LACTIN-V and the placebo arms. No grade 3 or 4 AEs or serious adverse events (SAEs) occurred. All three dose levels of LACTIN-V were safe and acceptable.[61]

This study was followed by a **Phase 2a trial (LV-006)** to assess colonization efficiency, safety, tolerability, and acceptability of *L. crispatus* CTV-05. [62]

Twenty-four participants (African American: 9; White: 12; Other: 3) diagnosed with BV were randomized 3:1 to use LACTIN-V (2×10^9 cfu/dose) vs. an inert placebo administered daily for 5 days, and once weekly for 2 weeks. Participants completed a 5-day treatment course with topical metronidazole (MetroGel®) and within 24-72 hours initiated a regimen of high-dose LACTIN-V or inert placebo, administered once daily for 5 consecutive days and then once weekly over 2 additional weeks. They returned for follow-up on Days 10 and 28.

Safety results: Of the 120 AEs that occurred, 108 (90%) were mild and only 12 (10%) were moderate in severity; no grade 3 or 4 AEs or SAEs occurred, and no deep epithelial disruption was seen during colposcopic evaluation. [62] Furthermore, AEs were evenly distributed between the LACTIN-V and placebo groups.

Efficiency of colonization: Sixty-one percent of the 18 women randomized to the LACTIN- V group were colonized with *L. crispatus* CTV-05 at Day 10 and/or Day 28. Among LACTIN-V users with complete adherence to the study regimen, 78% were colonized at Day 10 and/or Day 28. [62]

Effect of endogenous bacteria on vaginal colonization of exogenous *L. crispatus* CTV-05: The median vaginal concentrations of seven BV-associated bacteria declined in all participants between screening, when metronidazole treatment for BV was started, and enrolment. In participants who subsequently colonized with *L. crispatus* CTV-05, this trend was maintained at the Day 28 Visit when levels of six species were below limits of detection, with up to 7-$\log_{10}$ reductions in median values. In contrast, among participants who did not colonize with CTV-05, the concentrations of BV-associated bacterial DNA, especially those known to create a biofilm in the vagina [i.e., *A. vaginae* (p=0.04) and *G. vaginalis* (p=0.19)], resurged between enrolment and Day 28. Overall, this study provides evidence that vaginal colonization with a *L. crispatus* LBP can reduce colonization with diverse microbes, and potentially decrease inflammation and HIV risk. [63] We will evaluate the effect of LACTIN-V on the vaginal microbiome, expecting a shift to a *L. crispatus* dominant cervicotype.

Our **Phase 2b clinical trial** of LACTIN-V to prevent recurrent BV (NCT02766023) completed follow-up in February 2019. The trial was designed to provide a screening evaluation for the hypothesis that, following a 5-day treatment with MetroGel® (topical metronidazole) to treat BV, LACTIN-V administered at 2×10^9 cfu/dose using a vaginal applicator reduces the 12-week incidence of BV recurrence when compared to placebo. The primary objectives of this study were: 1) to estimate the efficacy of 5 daily doses in week 1 followed by twice weekly dosing in weeks 2-11 of LACTIN-V (2×10^9 cfu/dose) as compared to placebo in preventing BV recurrence by 12 weeks following treatment of BV with MetroGel; 2) to assess the safety of LACTIN-V over 24 weeks by comparing the incidence of AEs between individuals randomized to LACTIN-V or placebo. A total of 228 women underwent randomization: 152 to the Lactin-V group and 76 to the placebo group; of these participants, 88% in the Lactin-V group and 84% in the placebo group could be evaluated for the primary outcome. In the intention-to-treat population, recurrence of bacterial vaginosis by week 12 occurred in 46 participants (30%) in the Lactin-V group and in 34 participants (45%) in the placebo group (risk ratio after multiple imputation for missing responses, 0.66; 95%.

2.5 Rationale

The human vaginal microbiota has long been considered a factor impacting an individual's risk for acquiring sexually transmitted infections (STIs) such as HIV, but the extent of this contribution and the underlying mechanisms had not been well defined. We recently demonstrated that young South African women with vaginal microbial communities dominated by *Lactobacillus crispatus* had a 4-fold lower rate of HIV acquisition, reduced numbers of mucosal $CD4^+$ T cells and lower levels of genital tract proinflammatory cytokines compared with women with communities deficient in *Lactobacillus* species.[33, 31]

In longitudinal samples, genital tract proinflammatory cytokine concentrations increased when the cervical microbial community shifted from *Lactobacillus* dominance to a more diverse community dominated by other species [33], suggesting a cause/effect link between vaginal bacteria and genital tract inflammation. This link was further supported by the observation that germ-free mice intravaginally inoculated with high-risk bacteria had increased numbers of mucosal $CD4^+$ T cells in their female genital tract compared to mice inoculated with *L. crispatus*. [31]

The syndrome of vaginal dysbiosis known as bacterial vaginosis (BV) is characterized by a diverse, non-*Lactobacillus* dominant microbial community, similar to communities associated with an increased risk for HIV in our study. Standard treatment with antibiotics leads to a decrease in the presence of BV-associated microbes, but re-colonization with *Lactobacillus* species is often slow, and recurrence of BV is common.[64, 65] In Kenyan women, monthly treatment with antibiotics to reduce BV and decrease risk for STIs was successful in reducing recurrence of BV by 10%,[66] and was associated with an overall reduction in bacterial STI. [67] During the trial there was a 36% increase in the proportion of women with a *Lactobacillus*-dominant vaginal microbiota, [54] but this did not persist after stopping oral metronidazole. [68]

Given the apparent protection from infections afforded by a *Lactobacillus*-dominant vaginal microbiota and the limited efficacy of antibiotics in establishing such a community, a different intervention strategy may be necessary. *L. crispatus* CTV-05 (LACTIN-V) under development as a live biotherapeutic product (LBP) has shown excellent tolerability and close to 80% colonization in Phase 1 and 2a studies in the US. [61, 62]

We hypothesize that use of LACTIN-V by young women at high risk for HIV will decrease genital tract inflammation associated with increased HIV acquisition. We propose a randomized, placebo-controlled trial of this product in young South African women with a non-*Lactobacillus*-dominant microbiota to assess whether this intervention reduces proinflammatory cytokines and HIV target cells in the lower genital tract, leads to a persistent *Lactobacillus*-dominant vaginal microbial community, and is safe and acceptable in this population of young women at high risk for HIV.

We will conduct a randomized, placebo-controlled trial of the vaginal live biotherapeutic product LACTIN-V in a cohort of 60 young South African women. The trial will enrol women with a non-*Lactobacillus* dominant microbiota, will provide oral metronidazole 400 mg twice daily for seven days to all women, and will then randomize women 2:1 to LACTIN-V vs. placebo. Within 8-48 hours of taking the final metronidazole dose, women will receive the study product for five consecutive days, followed by twice weekly for three additional weeks. Women will be followed during the dosing (4 weeks) and post-dosing phase (4 weeks) for a total of 64 days.

The FRESH cohort consists of HIV-uninfected young women age 18-23 living in Umlazi, South Africa. In this region, <1% of 14 year old girls are HIV positive but over 60% of 24 year old women are HIV infected. [6] Since the study inception in 2012, over 1400 women have been enrolled. FRESH follows women for 9-months with twice weekly HIV viral load PCR testing. Paired blood and genital mucosal sampling occurs every three months with specific collection of cervical and vaginal swabs, cervical cytobrush and cervicovaginal lavage (CVL). Questionnaires are collected at each visit detailing symptomatic and sexual behavior data, diet, vaginal hygiene practices, contraceptive use, medical history and demographic factors. All participants undergo regular STI testing. Currently, over 6,000 CVL and cytobrushes, and 13,000 swabs have been stored from the study. We have also begun to culture, store, perform whole genome sequencing (WGS) on bacterial isolates to provide a detailed characterization of genomic and strain level variation within the cervicovaginal microbiome of these participants. Enrolment is ongoing with 300 new participants enrolled each year. Baseline characterization of cervicovaginal bacterial communities by 16s rRNA gene sequencing is performed on all participants.

Women who have a non-*Lactobacillus*-dominant vaginal microbiota community type and meet the inclusion and exclusion criteria will be offered enrolment in the LACTIN-V trial.

Funding for the core sample collection in FRESH has been secured from the Bill & Melinda Gates Foundation, Gilead Sciences Inc., and the Ragon Institute to cover the funding period of this grant proposal. Therefore, this study leverages an existing major site investment to enrol female participants, characterize their baseline vaginal microbiome and mucosal immune factors and perform detailed analysis on specimens collected as part of this cohort. There is no overlap between the work in this proposal and any other grants. The proposed Phase 2 trial of LACTIN-V within the FRESH cohort provides an unprecedented opportunity to study the effects of a *L. crispatus* on host-microbial interactions and genital tract inflammation.

LACTIN-V—if determined to be a safe and effective live biotherapeutic product (LBP)—has the potential to offer women a sustained, coitally independent, multi-purpose prevention product that promotes vaginal health and provides protection from HIV and potentially other STIs. By exploring the effects of LACTIN-V on genital tract mucosal immune markers associated with increased HIV susceptibility in young women, the ability of LACTIN-V to colonize the vagina and support a *L. crispatus*-dominant cervicotype in the female genital tract, and the safety of LACTIN-V in young South African women, we aim to provide critical data to aid in the continued development of vaginally administered LACTIN-V to prevent HIV-acquisition in women. The use of a safe LBP is an important paradigm shift in the development of HIV prevention technologies. LACTIN-V serves as an attractive alternative to the use of antiretroviral drug formulations currently under development. In addition, we will use molecular diagnostic technology to detect the effect of *L. crispatus C*TV-05 on the vaginal microbiota during follow-up.

2.6 Potential Risks and Benefits

2.6.1 Potential Risks

2.6.1.1 Biological Risks: LACTIN-V and Placebo

The study product contains a naturally occurring strain of *L. crispatus* CTV-05. To date, there has been considerable amount of experience using LACTIN-V at concentrations up to 2 x 10^9 cfu/dose without any apparent related adverse effects, with the exception of a short duration of asymptomatic vaginal discharge. No serious adverse events have been reported in Protocol No. LV-001, LV-002 and LV-004 using LACTIN-V at 5 x 10^6 and 5 x 10^8 cfu/dose, and no subject left the protocol due to an adverse event. No severe or severe-related adverse event has been experienced by more than one subject. In Protocol No. LV-003, using LACTIN-V at 5 x 10^8 cfu/dose, one subject in the placebo group experienced three simultaneous SAEs unrelated to LACTIN-V. Two subjects in the LACTIN-V group discontinued study treatment due to AEs; one subject became pregnant and the other reported nausea and diarrhea, which required treatment.

Safety results of the recently completed Phase 2a trial (LV-006) assessed colonization efficiency, safety, tolerability and acceptability of LACTIN-V at 2 x 10^9 cfu/ dose in 24 women with BV. No Grade 3 AEs or SAEs were observed during the clinical phase of the study. Based on the DAIDS Toxicity Table Addendum for Vaginal Microbicide Studies (November 2007) [69] designed to standardize AE assessment, a total of 120 total AEs were reported, 108 (90%) of which were Grade 1 and 12 (10.0%) were Grade 2 severity. The most common genitourinary AEs included vaginal discharge of study product (46%), abdominal pain (46%) and dysuria (21%). AEs were evenly distributed between LACTIN-V and placebo groups. All enrolled women (n=24) reported at least one AE. A single participant receiving placebo discontinued herself from study product due to a moderate AE (vaginal irritation).

Risks from the administration of LACTIN-V are low since colonization with this type of organism is strongly associated with improved vaginal health. No systemic risks are anticipated since this is an organism that is applied topically and not expected to be absorbed. These risks will be explained in the written informed consent form. No pregnant women, fetuses, prisoners, children, persons with an active STI or women undergoing in vitro fertilization are included in this study.

Adverse effects that may be associated with LACTIN-V include those seen with other vaginally administered products, and include:

Most likely:

- Vaginal, genital or menstrual symptoms: vaginal discharge

Less likely:

- Gastrointestinal symptoms: abdominal (stomach area) pain, constipation, diarrhea, nausea, vomiting
- Urinary symptoms: needing to urinate urgently, needing to urinate at night and pain with urination

- Vaginal, genital or menstrual symptoms: genital itching, bleeding between
- menstrual periods, delayed menstrual periods, vaginal odor, vaginal burning sensation, vaginal irritation, vaginal bleeding, vaginal dryness, genital swelling, rash, vaginal candidiasis (yeast infection)
- Other symptoms: lower back pain

Rare but potentially life-threatening:

- Allergic reaction (including anaphylaxis) to the study product

2.6.1.2 Metronidazole

Metronidazole is indicated in the treatment of bacterial vaginosis. It is contraindicated in patients with a prior history of hypersensitivity to metronidazole, parabens, other ingredients of the formulation, or other nitroimidazole derivatives.

Rare but serious:

- *Convulsive Seizures and Peripheral Neuropathy:* Convulsive seizures and peripheral neuropathy, the latter characterized mainly by numbness or paresthesia of an extremity, have been reported in patients treated with oral or intravenous metronidazole. The appearance of abnormal neurologic signs demands the prompt discontinuation of metronidazole vaginal gel therapy. Metronidazole vaginal gel should be administered with caution to patients with central nervous system diseases.
- *Psychotic Reactions:* Psychotic reactions have been reported in alcoholic patients who were using oral metronidazole and disulfiram concurrently. Metronidazole vaginal gel should not be administered to patients who have taken disulfiram within the last two weeks.

2.6.1.3 Biological Risk: Study Procedures

Participants may experience discomfort and slight vaginal bleeding when having pelvic exams for this study.

2.6.1.4 Biological Risk: Non-spermicidal lubricated condoms

Participants may be at a slightly increased risk of pregnancy, when using the study-provided non-spermicidal condoms, especially if the condom is not used correctly.

2.6.1.5 Social and Psychological Risks

Social Risks. (1) Participants may experience discord in their intimate relationships (boyfriend/sexual partner(s)) as a result of the request to abstain from sex for 12 hours following study product administration and for 24 hours prior to 3 separate scheduled pelvic examinations. (2) Participants may also experience tension between themselves and their co-enrollees in the FRESH cohort who were unable to join the LACTIN-V study for any

reason - e.g., not meeting the eligibility criteria or due to the limited study enrolment target.

Psychological Risks. (1) Participants may become embarrassed, worried, or anxious when receiving STI counseling. They may become worried or anxious while waiting for their STI test results. (2) Participants may experience anxiety from not knowing which Study Product they are receiving, LACTIN-V or the placebo drug. (3) Participants may experience shyness or embarrassment when sharing details of sexual practices and/or undergoing pelvic exams.

2.6.1.6 Legal and Financial Risks

There are no legal or financial risks related to study participation. All study costs are paid by the study sponsor.

2.6.1.7 Procedures for Minimizing Potential Risks

Biological risks. Participants will be under the care of a specialist physician and experienced professional nurses, who will closely monitor participant health and safety throughout the trial. Allergic reactions (including anaphylaxis): Participants with a known allergy to components of the study product will be excluded from enrolment. If an allergy or sensitivity occurs during the course of the study, the participant will be advised to immediately discontinue study product use and seek medical attention. All such events will be reported as an AE.

Psychological risks. Questions about sensitive information, such as sexual practices and STIs will be asked by counselors and nurses who have years of experience working with this demographic of young women and counsel in such a way to minimize stigma associated with sharing such intimate information. Adequate preparation and education of participants around sexual health and STIs will be provided. Similarly, careful education about the need for blinding, randomization and use of a placebo agent will be provided. Participants will receive counseling prior to STI testing. Counselors will prepare them for possible feelings of anxiety and the ramifications of a positive test result. All results will be kept confidential by the study physician/nurse and will be maintained in a limited access database.

Social risks. Study staff will provide counseling and guidance to participants around communication with intimate partners. The importance of abstaining from sex will be explained (to minimize factors that could affect efficacy of the study product) and a calendar outlining the specific dates/times that abstinence has been requested will be provided to each participant. Participants will participate in focused discussions and may engage in role-play exercises to practice communication with their intimate partners. To mitigate tension and jealousy with co-enrollees in the FRESH cohort, all FRESH participants will be provided information about the study, including the eligibility and exclusion criteria and the targeted enrolment for the LACTIN-V study.

2.6.2 Potential Benefits

2.6.2.1 Possible direct benefits to study participants

There may be no direct benefits to participants in this study.

2.6.2.2 Benefits related to clinical care

Participants will be screened for urinary tract infection, pregnancy, bacterial vaginosis and a number of STIs and referred for treatment, if clinically indicated. (Treatment for UTI will consider the urine test result, symptoms, and medical history.) Participants with a non-*Lactobacillus*-dominant microbiota will receive study-supplied oral Metronidazole treatment. As needed, women will be provided long acting injectable contraceptives during the study.

Participants will be under the care of a specialist physician and experienced professional nurses, who will closely monitor participant health and safety throughout the trial.

2.6.2.3 Benefits related to public health

Participants and others may benefit in the future from information learned from this study. Information learned in this study may lead to the development of a safe and effective live biotherapeutic product that can help normalize the vaginal microbiome to a *Lactobacillus*-dominant state, and potentially decrease the risk of HIV acquisition.

2.6.2.4 Financial benefits

Participants will not directly benefit financially from this study, but will be remunerated for their time and transportation cost. In addition, lunch and refreshments will be provided at all study visits.

2.6.2.5 Prospects of tested intervention being available to the study population if proven effective

Should LACTIN-V be proven effective for reducing the risk of HIV acquisition in women following a definitive Phase 3 efficacy trial, the study sponsor UCSF and the drug developer Osel, Inc. would plan to obtain a new drug approval from the South African Health Products Regulatory Authority (SAHPRA).

3.0 HYPOTHESIS AND OBJECTIVES

3.1 Study Hypothesis

We hypothesize that use of LACTIN-V by young women at high risk for HIV will decrease genital tract inflammation associated with increased HIV acquisition. We propose randomized, placebo-controlled trial of this product in young South African women with a non Lactobacillus-dominant microbiota to assess whether this intervention reduces proinflammatory cytokines and HIV target cells in the lower genital tract, leads to a persistent Lactobacillus-dominant vaginal microbial community, and is safe and acceptable in this population of young women at high risk for HIV.

3.2 Summary of Methods

We will conduct a randomized, placebo-controlled trial of the vaginal live biotherapeutic product LACTIN-V in 60 young South African women. The trial will enrol women with a non-*Lactobacillus* dominant microbiota, will provide oral metronidazole 400 mg twice daily for seven days to all women, and will then randomize women 2:1 to LACTIN-V vs. placebo. Within 8-48 hours of the final metronidazole dose, women will receive the study product for five consecutive days, followed by twice weekly for three additional weeks. Women will be followed during the dosing (4 weeks) and post-dosing phase (4 weeks) for a total of 64 days.

3.3 Study Objectives

3.3.1 Primary Objectives

1) Determine the effect of repeat dosing of LACTIN-V (2 x 10^9 cfu/dose) on genital tract inflammation in young South African women at risk of HIV.
2) Determine the ability of LACTIN-V to promote a *Lactobacillus*-dominant vaginal microbiota in young South African women at risk of HIV.
3) Determine the safety and acceptability of LACTIN-V in a population of young South African women at risk for HIV.

3.4 Study Outcome Measures

3.4.1 Primary/ Secondary Outcome Measures

Primary / Secondary Outcome Measures for Objective 1 (effect on genital tract proinflammatory cytokines and HIV target cells)

The proportion of participants with decreased genital tract proinflammatory cytokines and HIV target cells at 6 and 10 weeks of follow up, compared to levels at the baseline FRESH Study Visit, and Randomization Visit on Day 8 of the study (after treatment with Metronidazole is completed).

Our outcome will be a change after 4 weeks of treatment with LACTIN-V in comparison to

placebo, for the following:

i. a decrease of the concentrations of proinflammatory cytokines in genital fluid that have been associated with risk for HIV acquisition,
ii. a decrease in the number of activated HIV target cells (i.e. CD4+/CCR5+/HLA-DR+/CD38+ T cells) in the endocervix and
iii. an increase the anti-HIV effect of vaginal secretions relative to placebo.

*Primary / Secondary Efficacy Outcome Measures for Objective 2 (promoting Lactobacillus-*dominant microbiota)

1:
The proportion of participants with increased *Lactobacillus*-dominant vaginal microbiota in young South African women, compared to levels at randomization (Day 8) after completion of metronidazole. Measured will be overall *Lactobacillus* species, as well as *Lactobacillus crispatus, Lactobacillus jensenii, Lactobacillus gasseri, and Lactobacillus iners.*

Our primary outcome is the presence of *Lactobacillus*-dominant vaginal microbial cervicotypes after 4 weeks of treatment with LACTIN-V, and following the post- dosing phase at Day 64.

Secondary outcomes include comparison of *L. crispatus* vs. *L. iners* prevalence between treatment groups, persistence of colonization after stopping therapy, effect of sex and/or vaginal hygiene practices on colonization by lactobacilli, and stability of vaginal microbial communities over time in the treatment vs. placebo group.

2:
The proportion of participants with a positive BV diagnosis in each study arm by Day 64.
BV is defined Nugent score 7-10. Following FDA guidance all BV diagnoses during follow-up visits are considered incident, as they occur at least 22-30 days after the commencement of Metronidazole treatment and consequently are treatment failures or new infections.[70] For the purpose of this trial, treatment failure and new infection will both be considered recurrent BV.

3:
The proportion of participants experiencing successful colonization with *L. crispatus* CTV-05 following dose of study product through final visit (approximately Day 36 and 64) in the LACTIN-V arm.

4:
The proportion of subjects with cervicotypes I-IV compared by study arm.

Primary / Secondary Outcome Safety Measures for Objective 3 (safety and acceptability)

Safety of LACTIN-V and the applicator will be measured by:

The proportion of participants reporting product-related AEs and SAEs through the final visit that are likely related to use of study product compared by study arm, in particular Grade 3 AEs and SAEs.

Acceptability of LACTIN-V and the applicator will be measured by standardized questionnaire about acceptability of the study product and participants' stated willingness to use this type of product in the future, in each study arm.

4.0 STUDY DESIGN

This is a Phase 2 randomized double-blind placebo-controlled trial to assess the impact of the live biotherapeutic product LACTIN-V containing *Lactobacillus crispatus* CTV-05 on the vaginal microbiome of young women in South Africa at high risk of HIV acquisition.

The study will also assess the safety of LACTIN-V by comparing the incidence of AEs between women randomized to LACTIN-V or placebo.

We will conduct a randomized, placebo-controlled trial of LACTIN-V in a cohort of young South African women with a non-*Lactobacillus* dominant microbiota, who are sexually experienced, age 18 to 23 years. Enrolment will be continued until 60 women will be randomized into the study.

Eligible women will be treated with oral metronidazole 400 mg twice daily for seven days and will then be randomized 2:1 to LACTIN-V vs. placebo.

Within 8-48 hours of taking the final metronidazole dose, women will receive the study product for five consecutive days, followed by a twice weekly dose for three additional weeks.

After enrolment and metronidazole treatment, women will be followed throughout the 4 weeks of dosing and the 4 weeks of post-dosing phase for a total of 10 weeks (until approximately Day 64).

The primary outcome (**Objective 1)** focuses on the ability of the *L. crispatus* CTV-05 to acutely decrease genital tract inflammation (as a marker of HIV susceptibility). This will be assessed by collecting cervicovaginal fluid and endocervical immune cells at randomization (Day 8), after 4 weeks of treatment with the LACTIN-V vs. placebo (Day 36), and after 4 weeks of post-dosing phase (Day 64).

Direct anti-HIV activity will be assessed with *in vitro* HIV infection assays using vaginal fluid samples from randomization and after treatment: Randomization Visit (Day 8), Follow-up Visit (Day 36), and Final Visit at Day 64.

A secondary outcome (**Objective 2**) is to assess the ability of the *L. crispatus* CTV-05 to establish durable vaginal colonization as with the potential as a self-sustaining prevention strategy. This will be assessed by characterization of the vaginal microbiome at the baseline FRESH Study Visit, and the end of treatment (Day 36) and at the Final Visit (Day 64), after 4 additional weeks during the post-treatment phase.

Finally, while LACTIN-V has been shown to be safe and well tolerated in US women, we will assess the safety and tolerability **(Objective 3**) in this cohort of young South African women up to Week 9 (4 weeks post-dosing phase).

The Umlazi site of the FRESH study enrols approximately 300 women per year into the cohort. During the baseline Screening Visit of the FRESH enrolment process, new cohort members will learn about the parallel LACTIN-V clinical trial, including the findings from the FRESH study which demonstrated the association of a *L. crispatus* cervicotype with a reduced risk of HIV acquisition.

At the **FRESH Study Visit conducted in Week 5** of the FRESH protocol, a vaginal smear is collected sent to the site laboratory for Gram stain evaluation by Nugent score. For STI diagnostics, a vaginal swab for *Neisseria gonorrhoeae, Chlamydia trachomatis*, *Trichomonas* and *Mycoplasma genitalium* molecular testing will be collected, as well as a urine sample for a Point-of-care (POC) urine testing and a β- hCG-based pregnancy test. Test results for STIs as well as the Gram Stain are expected to arrive within one week.

Any women who enrolled in the FRESH study who express interest in the LACTIN-V trial and during the Week 5 FRESH Study Visit were diagnosed with a Nugent score of 4-10 on vaginal Gram Stain, and without any positive tests for the STIs listed in the exclusion criteria, will be contacted by study staff about their preliminary eligibility for the LACTIN-V trial, and will be offered concurrent enrolment in the LACTIN-V study.

In consideration of their menstrual cycle (they should not be bleeding at the time of enrolment and during the first five days of product administration), a LACTIN-V Enrolment Visit is scheduled at least 7 days out, not to exceed 30 days from the FRESH Week 5 Visit to ensure a high likelihood that the test results of that visit are still relevant.

Women will return to the study clinic for enrolment into the LACTIN-V study. During the informed consent procedure, they will learn details about the LACTIN-V study.

At the **Enrolment Visit 1 (Day 1)**, participants will complete a medical history and undergo a physical examination. Enrolled participants will receive oral Metronidazole 400mg tablets, and are instructed to immediately start the 7-day course (twice daily), to be completed within 8-48 hours of the Randomization Visit on Day 8. A **Check-In Visit 2 (Day 4)** is scheduled to provide additional guidance for the metronidazole administration.

At the **Randomization Visit 3 (Day 8),** a gynaecological exam will be conducted to collect baseline vaginal microbiome data, including cervical swabs, cervicovaginal lavage fluid and an endocervical cytobrush to assess the microbial composition, cytokines and mucosal HIV target cells. A gram stain and a vaginal swab for *L. crispatus* (including CTV-05) identification will be collected. Only participants who completed their 7-day course of oral Metronidazole will be randomized and receive study product.

The 11 doses of LACTIN-V at 2 x 10^9 cfu/dose or placebo are scheduled to be administered by vaginal applicator for 5 consecutive days and then twice weekly for 3 weeks, throughout Week 1-4 of the study. The first dose of study product is administered as part of the Randomization Visit on Day 8. Participants will be instructed to take the second and third dose on subsequent Days 9 and 10 at home before bedtime. All other doses will remain at the clinic site, where the women are attending regular twice-weekly visits as participants of the FRESH study. During these FRESH visits, they will be handed the remaining LACTIN-V applicators to administer on site according to schedule in a form of directly observed therapy.

During the study, participants will be counselled to use the provided lubricated or unlubricated non-spermicidal condoms during vaginal intercourse. Study staff will provide condoms that have been tested to not interfere with lactobacilli growth.[71] In addition, participants are counselled to avoid vaginal intercourse during the 12 hours before scheduled gynaecological exams, as this activity could lead to microabrasions notable at speculum exam, and could interfere with a correct determination of symptoms related to study product administration.

Further, participants will be counselled to avoid sexual intercourse for 12 hours after study product administration to ensure that the product will remain inside the vagina.

Brief Check-in Follow-up Visits 4 - 10 (Days 11, 15, 18, 22, 25, 29 and 32) without physical and gynaecological exams to assess adherence, menstrual cycle, sexual activity, condom use, concomitant medications, and AEs, and to administer subsequent doses of study product are scheduled to coincide with regular clinic visits as part of the FRESH schedule (twice weekly either Monday/Thursday or Tuesday/Friday).

Week 1: Day 8 (where the doses for Day 9 & 10 are handed to participants to take at home)
Day 11 (where the dose for Day 12 is handed to participants to take at home)
Week 2: Day 15 and 18 (Doses 6 and 7) administered in clinic
Week 3: Day 22 and 25. (Doses 8 and 9) administered in clinic
Week 4: Day 29 and 32. (Doses 10 and 11) administered in clinic

At the beginning of Week 5, at the end of the dosing phase, a **Follow-up Visit 11** is scheduled for **Day 36**. This visit will include a physical and gynaecological exam to collect vaginal microbiome data, including cervical swabs, cervicovaginal lavage fluid and an endocervical cytobrush to assess the microbial composition, cytokines and mucosal HIV target cells. A gram stain and a vaginal swab for L. crispatus (including CTV-05) identification will be collected.

Should symptoms or the sexual history suggest the acquisition of an STI, testing for STIs will be included. Acceptability will be assessed using a questionnaire.

During Week 5 – 8, study staff will continue to check in with participants twice weekly for brief **Check-in Follow-up Visits 12 – 18 (Days 39, 43, 46, 50, 53, 57 and 60)** without physical and gynaecological exams to assess menstrual cycle, sexual activity, condom use, concomitant medications, and AEs. These are scheduled to coincide with regular clinic visits as part of the FRESH schedule (twice weekly either Monday/Thursday or Tuesday/Friday).

Week 5: Day 39
Week 6: Day 43 and 46
Week 7: Day 50 and 53
Week 8: Day 57 and 60

The **Final Visit 19 (Day 64)** 4 weeks post dosing will include a physical and gynaecological exam to collect vaginal microbiome data, including cervical swabs, cervicovaginal lavage fluid and an endocervical cytobrush to assess the microbial composition, cytokines and mucosal HIV target cells. A gram stain and a vaginal swab for L. crispatus (including CTV-05) identification will be collected. Should symptoms or the sexual history suggest the acquisition of an STI, testing for STIs will be included.

Electronic reminders per cell phone will be sent to women prior to each scheduled visit. See complete listing of all study visits in the timeline below:

Wk	Monday (Tuesday)	Tuesday (Wednesday)	Wednesday (Thursday)	Thursday (Friday)	Friday (Saturday)	Saturday (Sunday)	Sunday (Monday)
0	Day 1<br>**Enrolment Visit 1**<br>Informed Consent<br>Metronidazole<br>1 & 2	2<br><br><br>Metronidazole<br>3 & 4	3<br><br><br>Metronidazole<br>5 & 6	4<br>**Visit 2**<br>Check-in & dispense<br>Metronidazole<br>7 & 8	5<br><br><br>Metronidazole<br>9 & 10	6<br><br><br>Metronidazole<br>11 & 12	7<br><br><br>Metronidazole<br>13 & 14
1	8<br>**Randomization**<br>**Visit 3**<br>Gynaecol. exam<br>Dose 1, on site	9<br><br><br><br>Dose 2, at home	10<br><br><br><br>Dose 3, at home	11<br>**Visit 4**<br>AE Check<br><br>Dose 4, on site	12<br><br><br><br>Dose 5, at home	13	14
2	15<br>**Visit 5**<br>AE Check<br>Dose 6, on site	16	17	18<br>**Visit 6**<br>AE Check<br>Dose 7, on site	19	20	21
3	22<br>**Visit 7**<br>AE Check<br>Dose 8, on site	23	24	25<br>**Visit 8**<br>AE Check<br>Dose 9, on site	26	27	28
4	29<br>**Visit 9**<br>AE Check<br>Dose 10, on site	30	31	32<br>**Visit 10**<br>AE Check<br>Dose 11, on site	33	34	35
5	36<br>**Visit 11**<br>Gynaecol. exam	37	38	39<br>**Visit 12**<br>AE Check	40	41	42
6	43<br>**Visit 13**<br>AE Check	44	45	46<br>**Visit 14**<br>AE Check	47	48	49
7	50<br>**Visit 15**<br>AE Check	51	52	53<br>**Visit 16**<br>AE Check	54	55	56
8	57<br>**Visit 17**<br>AE Check	58	59	60<br>**Visit 18**<br>AE Check	61	62	63
9	64<br>**Visit 19**<br>Gynaecol. exam						

5.0 STUDY ENROLMENT AND WITHDRAWAL

The study plans to enrol non-pregnant women, age 18 to 23 years, diagnosed with a non-*Lactobacillus* dominant microbiota (diagnosed by Nugent 4 – 10).

Enrolment will be continued until 60 women have been randomised into the study. Eligibility criteria for participation in the study are described in detail in sections 5.1 and 5.2.

The consent process will be completed during the Enrolment Visit 1 (Day 1) to determine eligibility. Pregnant women, men and children are excluded from study participation (see section 14.4).

5.1 Subject Inclusion Criteria

Participants must meet all of the inclusion criteria in order to be eligible to participate in the study:

1. FRESH study participant.
2. Capable of reading and writing English or isiZulu and voluntarily provide written informed consent to participate in the study and comply with all study procedures
3. HIV-negative
4. Nugent score 4-10 on vaginal Gram stain
5. Otherwise healthy women, 18–23 years of age on the day of enrolment
6. Regular predictable menstrual cycles or amenorrhoeic for at least 3 months due to use of a long-acting progestin.
7. Willing to complete 7-day course of oral metronidazole.
8. Willing to be asked questions about personal medical health and sexual history
9. Willing to apply study agent vaginally and comply with study examinations
10. Willing to self-administer Study Product on dosing days that do not coincide with regular FRESH study visits.
11. Agree to try to abstain from sexual intercourse 12 hours prior to study visits that include a gynaecological exam (Randomization Visit 3, Follow-up Visit 11, Final Visit 19).
12. Agree to try to abstain from sexual intercourse for 12 hours after study product administration to ensure that the product will remain inside the vagina.
13. Agree to abstain from the use of any other vaginal product throughout the trial period from the time of enrolment through the end of the study.

    Note: Intravaginal products include contraceptive creams such as Gynol II, gels, foams, sponges, lubricants not approved by the study investigators, tampons and douches.
14. Must be stable on a reliable method of long-acting birth control and agree to remain on, for the duration of the study (if of childbearing potential) or, of non-childbearing potential (permanently sterile).

5.2 Subject Exclusion Criteria

Participants meeting any of the following criteria when assessed at the Enrolment Visit, will be excluded from the study:

1. Urogenital infection (as tested during the FRESH Week 5 Study Visit, reported within 30 days of detection at the LACTIN-V Enrolment Visit).
   *<u>Note</u>**:** Urogenital infection includes urinary tract infection, Trichomonas (T.) vaginalis, Neisseria (N.) gonorrhoeae, Chlamydia (C.) trachomatis, Mycoplasma genitalium.*
2. Diagnosis of two or more outbreaks of *N. gonorrhoeae*, *C. trachomatis*, *T. vaginalis, Mycoplasma genitalium*, or herpes simplex virus (herpes genitalis) within 6 months prior to enrolment.
3. Subject is ineligible if menstrual cycle length is less than 21 days
4. Subject is ineligible if deep epithelial disruption is observed on genital examination noted on or before the Randomization Visit
5. Positive for HIV (as tested during the FRESH Week 5 Study Visit, within 30 days of the LACTIN-V Enrolment Visit).
6. Current pregnancy or within 2 months of last pregnancy
7. Vaginal or systemic antibiotic or antifungal therapy within 21 days of enrolment
8. Use of disulfiram within past 2 weeks or other contraindication to use of metronidazole
9. Any condition requiring regular periodic use of systemic antibiotics during participation in the trial
10. Investigational drug use other than LACTIN-V within 30 days or 10 half- lives of the drug, whichever is longer, of Enrolment Visit
11. Other planned participation in an investigational drug study while participating in this study
12. IUD insertion or removal, pelvic surgery, cervical cryotherapy or cervical laser treatment within the last 2 months prior to enrolment
13. Use of vaginal ring (e.g, NuvaRing) within 3 days of enrolment or during the course of the study
14. Hysterectomy
15. Unwilling to complete 7 days of oral metronidazole (twice daily) with the last dose taken no later than 48 hours prior to randomization (minimum of 12 of 14 doses required)
16. Use of new long-acting hormonal treatments. Participant may be enrolled if stable (at least 1 month) on existing therapy as determined by the principal investigator (PI)
17. Known allergy to any component of LACTIN-V/placebo or metronidazole or to nitroimidazole derivatives or latex (condoms)
18. Any social, medical, or psychiatric condition including history of drug or alcohol abuse that in the opinion of the investigator would make it difficult for the participant to comply with study procedures
19. Any serious or chronic illness, deemed incompatible with study participation by the study doctor, including immunosuppression due to cancer chemotherapy, systemic corticosteroids.

5.3 Treatment Assignment Procedures

5.3.1 Randomization Procedures

Women will be randomized at the Randomization Visit 3 (Day 8) upon completion of the initial standardized antibiotic treatment with 2 x 400 mg daily over 7 days of oral metronidazole.

Women will be randomly assigned to receive LACTIN-V at 2 x 10^9 cfu/dose or placebo in a 2:1 ratio. Enrolment will be continued until 60 women are randomized.

The list of randomized treatment assignments will be prepared by a statistician not involved with the study, and provided to the pharmacy, who will label the applicator pouches and cartons. Each carton will contain 11 study treatment applicators and be sequentially numbered according to the randomization scheme in a 2:1 (LACTIN-V to placebo) ratio.

Disenrolled participants before randomization will be replaced until 60 women are randomized into the study. Once randomized, participants will not be disenrolled.

Participants who test HIV-positive after randomization, will discontinue study product, but continue follow-up and attend all remaining study visits to monitor for adverse events. HIV-specific care, including the provision of antiretroviral treatment and ongoing management will be provided by the FRESH Study.

5.3.2 Blinding Procedures

At the time of randomization, the site pharmacist will select the next available box of study product applicators in sequential order and will distribute to masked study personnel with no labels that identify the product or applicators as LACTIN-V or placebo. No masking procedures are required for the metronidazole treatment.

The participants, study personnel who perform study assessments, data entry personnel at the sites, and laboratory personnel performing study assays will be blinded to treatment assignment. The Data and Safety Monitoring Board (DSMB) may receive data in aggregate and presented by treatment group, but without the treatment group identified. The DSMB may be unmasked to individual study treatment assignments, as needed, to adequately assess safety issues. Refer to the MOP for unmasking procedures, including emergency unblinding procedures.

5.3.3 Reasons for Withdrawal

5.3.3.1. Withdrawal after Enrolment

Participants may withdraw or be withdrawn for any of the reasons given below. The reason for withdrawal will be recorded in the data collection form.

At Randomization Visit 3:

- A diagnosis of a UTI, an active *herpes genitalis* lesion, or symptomatic vulvo-vaginal candidiasis

- Any diagnosis other than BV requiring antibiotics
- Failure to complete 7-day course of Metronidazole

Disenrolled participants before randomization will be replaced until 60 women are randomized into the study. Once randomized, participants will not be disenrolled.

At any Study Visit 3 – 19 after Enrolment Visit 1:

- Participant withdraws consent
- Pregnancy
- Adverse event which requires discontinuation of the treatment regimen or results in inability to comply with study procedures
- Discretionary decision by the site investigator
- At the discretion of the IRB at UKZN and UCSF, SAHPRA, NIH, or other government agencies as part of their duties to ensure that research participants are protected, or the industry supporter or its designee
- Study is terminated

5.3.4 Handling of Withdrawals

Disenrolled participants before randomization will be replaced until 60 women are randomized into the study. Once randomized, participants will not be disenrolled. All randomized participants who are discontinued from receiving further study product will continue to be followed through the Final Visit 19.

If withdrawal of consent or study product discontinuation occurs after study treatment is initiated, the participant will be asked to continue scheduled study procedures including safety evaluations, if possible, and be given appropriate care under medical supervision if symptoms of any AEs related to participation in the study are continuing. The participant will be followed until the AE is resolved or until the participant's condition becomes stable.

Pregnant women will discontinue study product and continue to be followed through the Final Visit 19 in Week 9. All samples will be collected except those directly collected at the cervix.

Participants who withdraw their consent for further participation in the study after their study treatment ends or discontinue study product early will be reminded of the importance of continuing in the study for safety evaluations. Participants will be encouraged to complete the Early Termination evaluations described in section 7.9 if they choose not to complete the remaining study visits.

Participants who enrol in the study but do not return for study visits after a minimum of three attempts to contact them over a 2-week period will be considered lost to follow-up.

5.3.5 Termination of Study

Study closure may occur due to DSMB review, or at the discretion of the Institutional Review Board (IRB) at the University of KwaZulu-Natal (UKZN) and UCSF, SAHPRA, NIH, and other government agencies as part of their duties to ensure that research participants are protected, or at the discretion of the industry supporter or its designee.

6.0 STUDY PRODUCT

6.1 Study Product Description

LACTIN-V *(Lactobacillus crispatus* CTV-05)

LACTIN-V contains a naturally occurring vaginal strain of *Lactobacillus (L.) crispatus* CTV-05 isolated from an African American woman in Seattle. LACTIN-V, preserved as a powder, is applied by a vaginal applicator at a potency of 2 x 10^9 cfu/dose. The LACTIN-V powder contains trehalose, xylitol, sodium ascorbate, colloidal silicon dioxide and maltodextrin. A matching placebo formulation without *L. crispatus* CTV-05 is supplied in an identical applicator containing placebo powder.

Metronidazole
Metronidazole is the oral dosage form of the synthetic antibacterial agent, metronidazole, at a concentration of 400mg per tablet. Metronidazole is a member of the nitro imidazole class of antibacterial agents and is classified therapeutically as an antiprotozoal and antibacterial agent. Chemically, metronidazole is a 2-methyl-5-nitro imidazole-1-ethanol.

At the Enrolment Visit 1, eligible participants will receive oral metronidazole for their non-Lactobacillus-dominant microbiota. Participants who receive metronidazole, but do not complete their full metronidazole treatment, do not return for the Randomization Visit 3, or are otherwise found ineligible to be randomized to study treatment will be not be randomized and will be withdrawn from the study.

6.1.1 Acquisition

LACTIN-V and placebo
Osel, Inc. will provide LACTIN-V and placebo. LACTIN-V and placebo will be transferred from List Biological Laboratories, Inc. to the following address:

The Aurum Institute
Department: Research Management
Division: Clinical Research
29 Queens Road, Parktown, Johannesburg, South Africa, 2193
Contact Number: +27 (0) 10 590 1300
Contact person: Jayajothi Moodley (Pharmacist)

Metronidazole
Metronidazole oral tablets will be purchased and supplied in South Africa.

6.1.2 Formulation, Packaging, and Labeling

LACTIN-V and placebo

Each applicator will contain a LACTIN-V powder formulation containing *L.crispatus*

CTV-05 at a potency of ~2 x 10^9 cfu/dose. LACTIN-V powder contains trehalose, xylitol, sodium ascorbate, colloidal silicon dioxide and maltodextrin. A matching placebo formulation without *L. crispatus* CTV-05 is supplied in an identical applicator containing placebo powder.

LACTIN-V or placebo pre-filled applicators will be individually packaged in heat-sealed foil pouches, each containing one desiccant packet. Each participant will receive one carton containing 11 applicators. Each foil pouch and box will be labeled with the study number and unique participant number.

The applicator pouches and cartons will be labeled by the research pharmacist at the Aurum Institute.

Each label will contain the study number. The label of each box of study product applicators will also include the unique participant number and the statements: "*Store in a cool, dry place out of reach of children (at room temperature).*"

Metronidazole oral tablets
Metronidazole oral tablets contain metronidazole at a concentration of 500 mg per tablet. Metronidazole is supplied in a box with 14 tablets. Each participant will receive one box of Metronidazole 400 mg sufficient for the 7-day course of treatment with oral tablets to be taken twice daily.

6.1.3 Product Storage and Stability

LACTIN-V and placebo

LACTIN-V and placebo applicators will be shipped in an appropriate container on frozen gel packs and immediately placed into temperature- controlled conditions (2–8°C) upon arrival. During transit, temperature will be recorded using a temperature logger.

Following the randomization code provided by an independent statistician, the research pharmacist at the Aurum Institute will label all applicators and package them in the cartons, each containing 11 applicators. The cartons will be stored at 2-8°C.

On the day of randomization (Randomization Visit 3), the study product will be transported from the research pharmacy to the site.

Cartons will be stored at the research site in a cool, dry place at room temperature until dispensed.

Participants will take home applicators for Days 2, 3 and 5, and will be instructed to store applicators in a cool, dry place at room temperature, and to avoid heat. Applicators should be kept out of reach of children.

Metronidazole oral tablets
Store at room temperature 15° to 30°C (59° to 86 °F). Protect from freezing, and

store out of reach of children.

6.2 Dosage, Preparation, and Administration of Study Product

LACTIN-V and placebo

At Randomization Visit 3 (Day 8), eligible participants will be instructed on how to insert the pre-filled applicator (LACTIN-V or the placebo) and will administer the first dose under direct supervision by the study clinician before leaving the clinic. Study personnel will verify that each carton contains the 10 remaining applicators, and this will be recorded on the appropriate Case Report Form (CRF).

Also, at the Randomization Visit 3, participants will be handed two applicators for the next two days (Day 9 and 10) and will be instructed to administer them at home before bedtime. They will return to the clinic on Day11 and receive one applicator for immediate administration, and the applicator for Day 12 to take home. The applicator cartons will be stored on site, and during Week 1-4, applicators will be dispensed twice weekly to study participants for immediate administration.

Metronidazole oral tablets

Participants will be instructed to administer one tablet twice daily (one in the morning and one in the evening) initiated depending on the day set for enrolment. The 7-day course needs to be planned according to the requirement of completing the course within 8-48 hours of the Randomization Visit 3 (see Section 7.0 for additional details).

6.3 Accountability Procedures for the Study Investigational Product

The study PI has ultimate responsibility for the storage, dispensing accountability, and destruction of study products. For this protocol, the responsibility for study product management is being delegated to the research pharmacist.

The research pharmacist is responsible for storage at the research pharmacy and for distribution of product to the site. After receipt of the study product at the site, the site Principal Investigator (PI) is responsible for distribution and disposition of the study product to study participants and has ultimate responsibility for drug accountability. Both, the research pharmacist (or designee) and the site PI must maintain study product records and document logs of receipt, accountability, and storage temperature conditions. These study product accountability and dispensing logs must be maintained in the study file. Upon completion of the study and after the final monitoring visit, unused applicators will be retained until monitored and released for disposition as per the Sponsor. For detailed information regarding final disposition of study product see the protocol-specific MOP.

6.4 Assessment of Subject Compliance with Study Product

Compliance with study product will be ensured by directly observed administration in the clinic and through participant self-reporting for doses taken at home (Day 9, 10 and 12).

6.5 Concomitant Medications/Treatments

The study requires documentation of all medications (name, dose, route, frequency of dosing, and reason for use) taken by participants 30 days prior to enrolment through the final visit (Day 64) or early termination, whichever occurs first.

The following medications are permitted throughout the study:

- Vitamins and other nutritional supplements
- Hormonal contraceptives
- Cough medicine
- Non-steroidal anti-inflammatory drugs
- Prescription and over-the counter medications for allergies and asthma
- Herbal, naturopathic, and traditional preparations (e.g., Chinese traditional medications)
- Externally applied topical medications (except genital area)
- Antibiotics prescribed during the trial, including Metronidazole for BV

The following medications are prohibited throughout the study, and participants will be instructed to not use them throughout the study up to the Final Visit 19 (Day 64):

- Immune suppressants
- Investigational drug preparations other than the study product
- Intravaginal medications/preparations and topical medications/preparations other than the study product applied to the external genitalia

Any other medication/treatment not listed as permitted or prohibited is subject to the judgment of the investigator.

7.0 STUDY SCHEDULE

7.1 Enrolment Visit 1

The purpose of the Enrolment Visit 1 is to complete the informed consent process and identify participants who satisfy all eligibility criteria.

Women who are part of the FRESH study cohort can be recruited for the LACTIN-V study. Potentially eligible participants will be approached during the regular Week 5 FRESH Study Visit that includes a pelvic exam and mucosal sampling. Women will receive information about the opportunity to co-enrol in LACTIN-V. Should they be interested in participating in the LACTIN-V study, they will be told that some of the test results routinely collected at the Week 5 FRESH Study Visit will be used to assess their eligibility for LACTIN-V, and that they would be contacted should they meet the following eligibility criteria:

1. non-*Lactobacillus* dominated vaginal microbiota as determined by Nugent score 4-10 on vaginal Gram stain
2. negative STI testing results
3. on stable long acting injectable contraception, either on Depot Medroxyprogesterone Acetate (DepoProvera) or norethisterone enanthate (NetEn), or an IUD or implant for at least 3 months.

Interested women who meet these eligibility criteria for LACTIN-V will be contacted after review of the laboratory results, usually within 7-10 days. In accordance with their menstrual cycle and expected onset of the next menses, they will be invited and scheduled for a LACTIN-V Enrolment Visit that will take place on a date at least 12 days before the next expected menses, and no later than 30 days after the Week 5 FRESH Study Visit.

Eligible women who agree to be enrolled in LACTIN-V will be asked to sign the informed consent form available in English and isiZulu that will describe the purpose of the study, the procedures to be followed, and the risks and benefits of the study, and will include a separate section for consent to store samples for future use. An Assessment of Understanding form, available in English and isiZulu, will be completed to ensure that study participants retained the most important aspects of the provided information.
A copy of the consent form will be given to the participant and this fact will be documented in the medical record.

Participants will be asked to provide contact information (i.e., address, email address, home/cell phone number, emergency contact), which will be recorded on the appropriate contact form, which will be kept away from other study forms in a locked cabinet.

At the Enrolment Visit (Visit 1) the woman's menstrual cycle history will be evaluated to ensure regular menstrual cycles (or amenorrhea due to long-acting contraceptives).

Participants who give informed consent will undergo the following procedures and assessments at the Enrolment Visit 1:

1. Assessment of eligibility
2. Detailed structured interview to obtain demographic information, medical, gynaecological and sexual history
3. Review of concomitant medications
4. Complete physical examination by a study physician or nurse that will include vital

signs, including temperature, height and weight, and examination of respiratory and cardiovascular systems

5. Clean catch urine dipstick. If test results suggest a UTI, the woman will be managed as clinically indicated and not randomized at this time.
6. Rapid urine βhCG pregnancy test. If the test is positive, the Enrolment Visit 1 will be terminated as the woman is not eligible.
7. All findings at the time of the evaluation will be recorded on the CRF.
8. Subjects who meet all eligibility criteria will be given the first 6 of a total of 14 oral tablets of 400 mg Metronidazole to take twice daily in the morning and evening for the next 3 days (total of 7 consecutive days). They will take the first dose in the clinic.
9. Participants will be invited to return for the Check-In Visit 3 on Day 4 (to coincide with their next regular FRESH visit) to receive the remaining 8 doses for the last 4 days of the 7-day Metronidazole treatment.
10. Participants will be invited to return for the Randomization Visit 3 within 8-48 hours after completion of Metronidazole treatment.
11. Non-spermicidal condoms will be dispensed.
12. Enrolment into the study database

All women in the FRESH cohort come to the clinic twice weekly for HIV testing and life skills training. Depending on a woman's placement within the FRESH cohort, she is either attending Monday/ Thursday sessions or Tuesday/ Friday sessions. Randomization Visits will take place either Monday or Tuesday. Consequently, the 7-day Metronidazole course will have to be timed to start on the day of the Enrolment Visit 1, exactly one week prior to the Randomization Visit 3 (see Table 7.1).

Table 7.1

FRESH COHORT with regular clinic visits MONDAYS & THURSDAYS

MON	TUE	WED	THU	FRI	SAT	SUN	MON	TUE	WED	THU	FRI	SAT	SUN	MON
7-day oral Metronidazole (400mg per tablet, twice daily)							RANDOMIZATION DOSE 1	DOSE 2	DOSE 3	CHECK-IN VISIT, DOSE 4	DOSE 5			CHECK- IN VISIT

FRESH COHORT with regular clinic visits TUESDAYS & FRIDAYS

TUE	WED	THU	FRI	SAT	SUN	MON	TUE	WED	THU	FRI	SAT	SUN	MON	TUE
7-day oral Metronidazole (400mg per tablet, twice daily)							RANDOMIZATION DOSE 1	DOSE 2	DOSE 3	CHECK-IN VISIT, DOSE 4	DOSE 5			CHECK- IN VISIT

7.1.1. Rescreening Visit

Because of the complex enrolment logistics into two parallel studies, FRESH and LACTIN-V, no rescreening will be offered to ineligible volunteers.

7.2 Check-in Visit 2 (Day 4)

Coinciding with the second regular weekly visit of the FRESH study, a brief Check-in Visit will be conducted, during which directly observed product administration of the fourth dose of oral Metronidazole will occur, and the remaining doses 5- 7 will be handed to the participants to take at home on subsequent days.

7.3 Randomization Visit 3 (Day 8)

Before commencing with the physical and gynaecological exam, eligibility for randomization should be confirmed by reviewing the medical history and all laboratory results.

Only women who had negative STI tests and a Nugent score 4-10 on the vaginal specimen collected at the Week 5 FRESH Study Visit, are enrolled in LACTIN-V and completed a full 7-day course of oral Metronidazole (at least 12 of 14 doses, last dose within 8-48 hours of Randomization Visit 3) will proceed to be randomized. Women who did not complete a full 7-day course of oral Metronidazole and took less than 12 of 14 doses, or took the last dose more than 48 hours before the Randomization Visit 3, will be withdrawn from the study.

Participants will be asked to verify previously reported contact information which will be recorded on the appropriate form.

Additionally, her menstrual cycle history will be re-evaluated to ensure regular menstrual cycles (or amenorrhea with long-acting contraceptives) and that menstruation will not be expected during the next 5 days when the use of the study product for 5 consecutive days (days 1-5) has to occur. If she is not currently having menstrual bleeding and her period is not to be expected within the next 5 days, she will proceed with the Randomization Visit 3.

Participants will undergo the following procedures at the Randomization Visit 3 (Day 8):

1. Review of concomitant medications
2. The pelvic examination will be conducted in the following order:
    a. Examination of the external genitalia
    b. Speculum insertion and examination of vagina, fornices and cervix with naked eye. Water will be used to ease speculum insertion. No lubricant should be used as it could contain chlorhexidine which may kill *Lactobacillus*.
    c. Vaginal pH
    d. Vaginal swab for Gram stain (Nugent scoring system)
    e. Two Vaginal swabs for complete mucosal sampling including identification of vaginal bacteria, lactobacilli species, cytokines and HIV target cells as well as multiomic (e.g. proteomics, metabolomics, etc.) testing
    f. Cervical (or vaginal) swabs for nucleic acid amplification of N. gonorrhoeae, and C. trachomatis.

g. A cervicovaginal lavage sample
h. Vaginal swabs for *L. crispatus* identification with qPCR
i. Vaginal swab for prostate specific antigen testing as a proxy for recent exposure to semen

Women with findings on pelvic exam at the Randomization Visit 3, including active genital herpes lesions, cervicitis, vulvovaginitis or for other reasons will be considered screening failures, will not be enrolled and will be referred for further evaluation.

3. Rapid urine βhCG pregnancy test. If the test is positive, the Randomization Visit 3 will be terminated as the woman is not eligible.
4. Clean catch urine dipstick. If test results and clinical screening suggest a UTI, the woman will be managed as clinically indicated and not randomized at this time.
5. All clinical and laboratory findings at the time of the evaluation will be recorded on the CRF. These findings will include observations present on the pelvic exam such as genital findings including location; presence or absence, and character of vaginal or cervical discharge or lesions; and results of any on site tests.
6. If the clinician suspects the participant developed an STI since the Week 5 FRESH Study Visit, appropriate tests will be conducted and if needed the woman will be referred to standard of care treatment in compliance with current Centers for Disease Control and Prevention (CDC) STI and South African Department of Health STI Treatment Guidelines.[72] The participant cannot be randomized at this time.
7. Randomization
8. The carton corresponding to the randomization code that contains study product (LACTIN-V or placebo) will be retrieved from the research pharmacy, and the first three applicators will be dispensed to participants. Participants will self-administer the first dose of LACTIN-V/placebo in the clinic during this visit. Participants will then be instructed to administer the second and the third dose of LACTIN-V/or placebo once a day at bedtime for the next two days (Day 9, and Day 10) and return to the clinic for a brief Check-In Visit on Day 11, when she will receive the fourth dose to take directly, and the fifth dose to take at home on Day 12. The remaining Doses 6-11 will be administered in the clinic twice weekly for 3 weeks.
9. Participants will be counselled to try to abstain from sexual intercourse during the first 5 days of study product administration. During the twice weekly visits, participants will be counselled to try to abstain from sexual intercourse 12 hours after administering the study product, as well as, 12 hours before each study visit that includes a gynaecological exam (Visit 11 on Day 36, and Visit 19 on Day 64). The twice weekly brief Check-In Visits 4 – 10 and 12 - 18 are visits without a gynaecological exam.
10. Participants will receive non-spermicidal lubricated condoms for their male sexual partners who will be encouraged to use condoms throughout the study if they engage in vaginal intercourse.
11. Participants will be counselled not to use tampons or any other vaginal products throughout the study.
12. Participants will be advised to return for the following brief Check-In Visits for a check of symptoms, AEs, sexual activity and menses as well as on site study product administration:
    a. Check-In Visit 4 (Day 11), Dose 4 (Dose 5 to take home for Day 12)

b. Check-In Visit 5 (Day 15), Dose 6
c. Check-In Visit 6 (Day 18), Dose 7
d. Check-In Visit 7 (Day 22), Dose 8
e. Check-In Visit 8 (Day 25), Dose 9
f. Check-In Visit 9 (Day 29), Dose 10
g. Check-In Visit 10 (Day 32), Dose 11

Further, they will be advised to return for the following visits after the completion of the dosing phase:

h. Follow-Up Visit 11 (Day 36), including gynaecological exam
i. Check-In Visit 12 (Day 39)
j. Check-In Visit 13 (Day 43)
k. Check-In Visit 14 (Day 46)
l. Check-In Visit 15 (Day 50)
m. Check-In Visit 16 (Day 53)
n. Check-In Visit 17 (Day 57)
o. Check-In Visit 18 (Day 60)
p. and the Final Visit (Day 64), including gynaecological exam

These visits are timed to coincide with the regular twice weekly visits of the FRESH study.

13. Depending on the time of the enrolment and the average length of the woman's menstrual cycle, careful attention should be paid to the approximate time of her next menstrual period when scheduling gynacological visits. Later adjustments of those dates may be necessary.

Participants diagnosed with an STI after randomization (when showing symptoms at subsequent visits that are confirmed with laboratory testing) will be referred for standard treatment in compliance with current CDC STI Treatment Guidelines[72], and continue to receive study product.

7.4 Check-In Visits 4 – 10

Coinciding with the regular twice weekly visits of the FRESH study, brief Check-in Visits will be conducted, during which directly observed product administration of the remaining doses will occur (Doses 4 – 11).

a. Check-In Visit 4 (Day 11), Dose 4 (Dose 5 to take home for Day 12)
b. Check-In Visit 5 (Day 15), Dose 6
c. Check-In Visit 6 (Day 18), Dose 7
d. Check-In Visit 7 (Day 22), Dose 8
e. Check-In Visit 8 (Day 25), Dose 9
f. Check-In Visit 9 (Day 29), Dose 10
g. Check-In Visit 10 (Day 32), Dose 11

The following short assessments will be performed at these Check-In Visits 4 – 10:

1. Staff will review symptoms, concomitant medications, last menses, sexual activity and any medical problems since the last visit, which will be recorded on the appropriate CRF.
2. The participant will be asked to provide exact dates and times of those study product administrations that took place at home (Dose 2,3 and 5 only).
3. Staff will hand the appropriate dose (4, 6- 11) to the participant from the carton stored on site, labeled with the participant's study ID.
4. In Week 1 only, staff will hand the fifth dose to the participant from the carton on site, to take home and self-administer the next day (Day 12)
5. Staff will document the study product administration on the appropriate CRF.
6. Participants will be reminded not to use tampons or any other vaginal products throughout the study.
7. Participants will be encouraged to use the provided non-spermicidal condoms if they engage in vaginal intercourse throughout the study.
8. Participants will be counselled to try to abstain from sexual intercourse for 12 hours after each study product application and for 12 hours before those study visits that include a gynaecological exam.
9. An appointment for the next Check-In, Follow-Up or Final Visit will be confirmed or rescheduled if necessary. Depending on the time of the enrolment and the average length of the participant's menstrual cycle, careful attention should be paid to the approximate time of the participant's next menstrual period when scheduling those visits. Later adjustments of those dates may be necessary.

If the participant fails to cancel the appointment and reschedule, and she is unreachable during the agreed upon time for the telephone interview, the study team will make three attempts to reach the participant to reschedule the telephone interview.

7.5 Follow-up Visit 11 (Day 36) (Allowable window: Days 33 - 43)

The following procedures and assessments will be performed at the Follow-Up Visit 11, Day 36:

1. Participants will return on Day 36 (Monday or Tuesday). With staff they will review symptoms, concomitant medications, and any medical problems since the last visit, which will be recorded on the appropriate CRF.
2. Focused medical, gynaecological and sexual history since the last visit to include assessment of STI risk.
3. Symptom-directed physical and pelvic examination by a study physician or nurse that will include vital signs, including temperature, as well as assessment of symptoms and adverse events.
4. The pelvic examination will be conducted in the following order:
    a. Examination of the external genitalia
    b. Speculum insertion and examination of vagina, fornices and cervix with naked eye. Water will be used to ease speculum insertion. No lubricant should be used as it could contain chlorhexidine which may kill *Lactobacillus.*
    c. Vaginal pH
    d. Vaginal swab for Gram stain (Nugent scoring system)

e. Two Vaginal swabs for complete mucosal sampling including identification of vaginal bacteria, lactobacilli species, cytokines and HIV target cells as well as multiomic (e.g. proteomics, metabolomics, etc.) testing
f. Vaginal swab for prostate specific antigen testing as a proxy for recent exposure to semen
g. Vaginal swab for STI testing if indicated by symptoms
h. A cervicovaginal lavage sample
i. Vaginal swabs for *L. crispatus* identification with qPCR
If indicated, cervical (or vaginal) swabs for nucleic acid amplification of *N. gonorrhoeae, and C. trachomatis*.

5. Dipstick on clean catch urine; if abnormal, urinalysis (UA) will be performed.
6. Rapid Urine βhCG pregnancy test
7. All clinical and laboratory findings at the time of the evaluation will be recorded on the CRF. These findings will include observations present on the pelvic exam such as genital findings including location; presence or absence, and character of vaginal or cervical discharge or lesions; and results of any on site tests.
8. Participants will be reminded not to use tampons or any other vaginal products throughout the study.
9. Participants will be counselled to try to abstain from sexual intercourse for 12 hours before the Final Visit 19 (Day 64).
10. Participants will receive non-spermicidal lubricated condoms for their male sexual partners. Participants will be encouraged to use condoms if they engage in vaginal intercourse throughout the study.
11. Participants will be asked to complete a detailed self-administered questionnaire assessing the acceptability of the study product and the applicator.

Women with vaginal discharge or vaginitis symptoms during the Follow-Up Visit 11 will be referred for standard treatment. If the test results at the follow-up visit suggest a urinary tract infection, cervicitis or vulvovaginitis, the participant will be referred for standard treatment.

If the clinician suspects the woman to have developed an STI since the last visit, appropriate tests will be conducted and if needed the woman will be referred to standard of care treatment in compliance with current CDC Treatment Guidelines.[72]

Women who develop *symptomatic* BV confirmed by Nugent 7-10 on Gram stain at least 4 weeks after the last Metronidazole treatment will be retreated with Metronidazole. This practice follows CDC treatment guidelines and FDA recommendations. The 7-day course of Metronidazole will begin as soon as possible. Women diagnosed with *asymptomatic* BV during the follow-up visit will not be retreated with Metronidazole.

If test results and clinical screening suggest a UTI, the woman will be managed as clinically indicated.

Participants who test HIV-positive after randomization, will discontinue study product, but continue follow-up and attend all remaining study visits to monitor for adverse events.

HIV-specific care, including the provision of antiretroviral treatment and ongoing management will be provided by the FRESH Study.

7.6 Check-In Visits 12 –18

Coinciding with the regular twice weekly visits of the FRESH study, brief Check-Visits will be conducted.

a. Check-In Visit 12 (Day 39)
b. Check-In Visit 13 (Day 43)
c. Check-In Visit 14 (Day 46)
d. Check-In Visit 15 (Day 50)
e. Check-In Visit 16 (Day 53)
f. Check-In Visit 17 (Day 57)
g. Check-In Visit 18 (Day 60)

The following short assessments will be performed at these Check-In Visits 12 – 18:

1. Staff will review symptoms, concomitant medications, last menses, sexual activity and any medical problems since the last visit, which will be recorded on the appropriate CRF.
2. Participants will be reminded not to use tampons or any other vaginal products throughout the study.
3. Participants will be encouraged to use the provided non-spermicidal condoms if they engage in vaginal intercourse throughout the study.
4. Participants will be counselled to try to abstain from sexual intercourse for 12 hours after each study product application and for 12 hours before those study visits that include a gynaecological exam.
5. An appointment for the next Check-In will be confirmed or rescheduled if necessary.
6. At Check-In Visit 18: The appointment for the Final Visit 19 (Monday or Tuesday) will be confirmed. Depending on the time of the enrolment and the average length of the participant's menstrual cycle, careful attention should be paid to the approximate time of the participant's next menstrual period when scheduling those visits. Later adjustments of those dates may be necessary.

If the participant fails to cancel the appointment and reschedule, and she is unreachable during the agreed upon time for the telephone interview, the study team will make three attempts to reach the participant to reschedule the telephone interview.

7.7 Final Study Visit 19, Day 64 (Allowable window: Days 57 - 71)

The following procedures and assessments will be performed at the Final Visit 19, Day 64:

1. Participants will return on Day 64 (Monday or Tuesday). Staff will review symptoms, concomitant medications and any medical problems since the last visit, which will be

recorded on the appropriate CRF.

2. Medical, gynaecological and sexual history to include assessment of STI risk.
3. Symptom-directed physical and pelvic examination by a study physician or nurse that will include vital signs, including temperature, and examination of respiratory and cardiovascular systems, as well as assessment of symptoms and adverse events.
4. The pelvic examination will be conducted in the following order:
   a. Examination of the external genitalia
   b. Speculum insertion and examination of vagina, fornices and cervix with naked eye. Water will be used to ease speculum insertion. No lubricant should be used as it could contain chlorhexidine which may kill *Lactobacillus.*
   c. Vaginal pH
   d. Vaginal swab for Gram stain (Nugent scoring system)
   e. Two Vaginal swabs for complete mucosal sampling including identification of vaginal bacteria, lactobacilli species, cytokines and HIV target cells as well as multiomic (e.g. proteomics, metabolomics, etc.) testing
   f. Vaginal swab for prostate specific antigen testing as a proxy for recent exposure to semen
   g. Vaginal swab for STI testing if indicated by symptoms
   h. A cervicovaginal lavage sample
   i. Vaginal swabs for *L. crispatus* identification with qPCR
   j. Vaginal swab for prostate specific antigen testing as a proxy for recent exposure to semen
5. Dipstick on clean catch urine; if abnormal, urinalysis (UA) will be performed.
6. Rapid Urine βhCG pregnancy test
7. All clinical and laboratory findings at the time of the evaluation will be recorded on the CRF. These findings will include observations present on the pelvic exam such as genital findings including location; presence or absence, and character of vaginal or cervical discharge or lesions; and results of any on site tests.

Women with vaginal discharge or vaginitis symptoms at the Final Visit 19 will be referred for standard treatment.

If the test results and clinical screening at the follow-up visit suggest a urinary tract infection, cervicitis or vulvovaginitis, the participant will be referred for standard treatment.

If the clinician suspects the woman to have developed an STI since the last visit, appropriate tests will be conducted and if needed the woman will be referred to standard of care treatment in compliance with current CDC STI Treatment Guidelines. [72] The participant should be treated in case of positive findings.

Women who develop *symptomatic* BV confirmed by Nugent Score 7- 10 on Gram stain at least 4 weeks after the last Metronidazole treatment will be retreated with Metronidazole. This practice follows CDC treatment guidelines and FDA recommendations. The 7-day course of Metronidazole will begin as soon as possible. Women diagnosed with

asymptomatic BV during the follow-up visit will not be retreated with Metronidazole.

If test results and clinical screening suggest a UTI, the woman will be managed as clinically indicated.

Participants who test HIV-positive after randomization, will discontinue study product, but continue follow-up and attend all remaining study visits to monitor for adverse events. HIV-specific care, including the provision of antiretroviral treatment and ongoing management will be provided by the FRESH Study.

7.8 Study Product Discontinuation/Early Termination Visit

Study product discontinuation and early termination evaluations will be performed as described below. Women who must discontinue taking the study product, for reasons described in section 5.3.3.1, before the end of the study-defined treatment period, will be asked to stay in the study to be followed on study/off treatment until study completion. Women who terminate their participation in the study early will be asked to have one final study visit but could return to be followed on study/off treatment until study completion if circumstances of the termination change.

1. Staff will review symptoms, concomitant medications and any medical problems since the last visit, which will be recorded on the appropriate CRF. The participant will be asked to provide exact dates and times of the study product administration
2. Medical, gynaecological and sexual history to include assessment of STI risk
3. Symptom-directed physical and pelvic examination by a study physician or nurse that will include vital signs, including temperature, and examination of respiratory and cardiovascular systems, as well as assessment of symptoms and adverse events
4. The pelvic examination will be conducted in the following order:
    a. Examination of the external genitalia
    b. Speculum insertion and examination of vagina, fornices and cervix with naked eye. Water will be used to ease speculum insertion. No lubricant should be used as it could contain chlorhexidine which may kill *Lactobacillus.*
    c. Vaginal pH
    d. Vaginal swab for Gram stain (Nugent scoring system)
    e. Two Vaginal swabs for complete mucosal sampling including identification of vaginal bacteria, lactobacilli species, cytokines and HIV target cells as well as multiomic (e.g. proteomics, metabolomics, etc.) testing
    f. Vaginal swab for prostate specific antigen testing as a proxy for recent exposure to semen
    g. Vaginal swab for STI testing if indicated by symptoms
    h. A cervicovaginal lavage sample
    i. Vaginal swabs for *L. crispatus* identification with qPCR
    j. Vaginal swab for prostate specific antigen testing as a proxy for recent exposure to semen
5. Dipstick on clean catch urine; if abnormal, urinalysis (UA) will be performed.

6. Rapid Urine βhCG pregnancy test
7. All clinical and laboratory findings at the time of the evaluation will be recorded on the CRF. These findings will include observations present on the pelvic exam such as genital findings including location; presence or absence, and character of vaginal or cervical discharge or lesions; and results of any on site tests.
8. Administer acceptability questionnaire
9. If participants remain in follow up, they will receive non-spermicidal lubricated condoms for their male sexual partners. Participants will be encouraged to use condoms if they engage in vaginal intercourse throughout the study.
10. If the clinician suspects the participant developed an STI since the last visit, appropriate tests will be conducted and if needed the woman will be referred to standard of care treatment in compliance with current CDC STI Treatment Guidelines.

7.9 Unscheduled Visit

An unscheduled visit following a participant's request or the site investigator's recommendation should be recorded on the appropriate CRF. The following procedures and assessments may be performed at the unscheduled visit:

1. Staff will review symptoms, concomitant medications and any medical problems since the last visit, which will be recorded on the appropriate CRF. Additionally, the participant will be asked to provide exact dates and times of the study product administration.
2. Medical, gynaecological and sexual history to include assessment of STI risk
3. Symptom-directed physical and pelvic examination by a study physician or nurse that will include vital signs, including temperature, and examination of respiratory and cardiovascular systems, as well as assessment of symptoms and adverse events
4. The pelvic examination will be conducted in the following order:
    a. Examination of the external genitalia
    b. Speculum insertion and examination of vagina, fornices and cervix with naked eye. Water will be used to ease speculum insertion. No lubricant should be used as it could contain chlorhexidine which may kill *Lactobacillus.*
    c. Vaginal pH
    d. Vaginal swab for Gram stain (Nugent scoring system)
    e. Two Vaginal swabs for complete mucosal sampling including identification of vaginal bacteria, lactobacilli species, cytokines and HIV target cells as well as multiomic (e.g. proteomics, metabolomics, etc.) testing
    f. Vaginal swab for prostate specific antigen testing as a proxy for recent exposure to semen
    g. Vaginal swab for STI testing if indicated by symptoms
    h. Cervical (or vaginal) swabs for nucleic acid amplification of N. gonorrhoeae, and C. trachomatis.
    i. A cervico-vaginal lavage sample
    j. Vaginal swabs for *L. crispatus* identification with qPCR

5. Dipstick on clean catch urine; if abnormal, urinalysis (UA) will be performed.
6. Rapid Urine βhCG pregnancy test
7. All clinical and laboratory findings at the time of the evaluation will be recorded on the CRF. These findings will include observations present on the pelvic exam such as genital findings including location; presence or absence, and character of vaginal or cervical discharge or lesions; and results of any on site tests.
8. An additional unscheduled visit as deemed necessary by the site investigator to ensure short-term surveillance will be planned.
9. Participants will be reminded not to use tampons or any other vaginal products throughout the study.
10. Participants will be counselled to try to abstain from sexual intercourse for 12 hours after each study product application and 12 hours before the remaining study visits.
11. Participants will receive non-spermicidal lubricated condoms for their male sexual partners. Participants will be encouraged to use condoms if they engage in vaginal intercourse throughout the study.
12. An appointment for the next regular follow-up visit will be scheduled. Depending on the time of the enrolment and the average length of the participant's menstrual cycle, careful attention should be paid to the approximate time of the participant's next menstrual period when scheduling those visits. Later adjustments of those dates may be necessary.

Women with vaginal discharge or vaginitis symptoms at the unscheduled visit will be referred for standard treatment and continue to receive study product.

If the test results at the unscheduled visit suggest a urinary tract infection, cervicitis or vulvovaginitis, the participant will be referred for standard treatment and continue to receive study product.

If the clinician suspects the woman to have developed an STI since the last visit, appropriate tests will be conducted and if needed the woman will be referred to standard of care treatment in compliance with current CDC STI Treatment Guidelines.[72] The participant should be treated in case of positive findings and continue to use study product.

Women who develop *symptomatic* BV confirmed by Nugent 7-10 on Gram stain at least 4 weeks after the last Metronidazole treatment will be retreated with Metronidazole. This practice follows CDC treatment guidelines and FDA recommendations. The 7-day course of Metronidazole will begin as soon as possible.

Women diagnosed with *asymptomatic* BV during the follow-up visit will not be retreated with Metronidazole.

If test results and clinical screening suggest a UTI, the woman will be managed as clinically indicated.

Participants who test HIV-positive after randomization, will discontinue study product, but continue follow-up and attend all remaining study visits to monitor for adverse events. HIV-specific care, including the provision of antiretroviral treatment and ongoing management will be

provided by the FRESH Study.

8.0 STUDY PROCEDURES/EVALUATIONS

8.1 Clinical Evaluations

The following assessments and procedures will be performed:

- Sociodemographic and participant contact information
- Medical, gynaecological and sexual history
- Review of concomitant medications
- Physical exam to include vital signs
- Pelvic exam
- Acceptability questionnaire

8.2 Laboratory Evaluations

8.2.1 Clinical Laboratory Evaluations

At the clinical site, the following tests will be performed using approved standard in-house tests:

- Pregnancy will be tested by standard rapid urine βhCG assays.
- Urine will be tested by urine dipstick for evidence of urinary tract infection, with follow-up urinalysis (UA), if abnormal.
- HIV testing will be conducted using standardized algorithms.*
- Cervical (or vaginal) swabs will be tested for *N. gonorrhoeae*, *T. vaginalis*, *Mycoplasma genitalium* and *C. trachomatis* by nucleic acid amplification.
- Gram-stained vaginal smear by Nugent's scoring system for diagnosis of BV

8.2.2 Special Assays or Procedures

- Vaginal specimens will be analyzed for vaginal colonization with *L. crispatus* using qPCR assays and 16S rRNA gene/shotgun sequencing, which will be performed by a central laboratory at the Ragon Institute of MGH, MIT and Harvard
- Cervicovaginal lavage (CVL) specimens will be analyzed for cytokines and chemokines using a luminex array
- Cervical cytobrush samples will be used to assess presence of HIV target cells by flow cytometry.
- Vaginal specimens will be analyzed using multiomic (e.g. proteomics, metabolomics, etc.) testing.
- Vaginal samples will be used for culturing of live bacterial isolates for quenching and functional analysis
- CVL will also be used for in vitro anti-HIV activity assays

8.2.3. Specimen Preparation, Handling, and Shipping

- Instructions for Specimen Preparation, Handling, and Storage will be described in further detail in the MOP.
- Many of the collected samples will be analyzed in laboratories in Durban and at UKZN.
- But some of your collected vaginal swabs will be shipped to a laboratory located in Boston, USA, headed by Dr. Douglas Kwon of the Ragon Institute at MGH.
- Mucosal sampling swabs, and cytobrush cells will all be initially processed and stored at UKZN. Further analysis will be performed at both UKZN and in the U.S. at the Ragon Institute of MGH, MIT and Harvard. All work performed in the U.S. will be done in close collaboration with South African LACTIN-V investigators.
- Instructions for specimen shipment will be described in further detail in the MOP.

9.0 ASSESSMENT OF SAFETY

9.1 Specification of Safety Parameters

All AEs and SAEs will be collected through the Final Visit 19 (Day 64).

9.2 Methods and Timing for Assessing, Recording, and Analyzing Safety Parameters

9.2.1 Adverse Events

ICH (International Conference on Harmonisation) E6 [73] defines an AE as any untoward medical occurrence in a patient or clinical investigation subject administered a pharmaceutical product regardless of its causal relationship to the study treatment. An AE can therefore be any unfavorable and unintended sign (including an abnormal laboratory finding), symptom, or disease temporally associated with the use of medicinal (investigational) product. The occurrence of an AE may come to the attention of study personnel during study visits and interviews of a study participant presenting for medical care, or upon review by a study monitor.

All AEs including local and systemic reactions not meeting the criteria for "serious adverse events" should be captured on the appropriate CRF and will include:

- Vaginal bleeding other than menstruation
- Abnormal vaginal discharge
- Abnormal vaginal odor
- Genital itching
- Genital burning
- External genital irritation
- External genital swelling
- Nausea
- Vomiting
- Abdominal pain/cramps
- Diarrhea
- Constipation
- Genital rash
- Pain/burning with urination
- Frequent urination
- Blood in urine
- Headache

Information to be collected includes event description, time of onset, clinician's assessment of severity, relationship to study product (assessed only by those with the training and authority to make a diagnosis), and time of resolution/stabilization of the event. All AEs occurring while on study must be documented appropriately regardless of relationship. All AEs will be followed to adequate resolution.

Any medical condition that is present at the time that the participant is screened should be considered as baseline and not reported as an AE. However, if it deteriorates at any time during the study, it should be recorded as an AE. All AEs must be graded for severity and relationship to study product. The FDA defines an AE as any untoward medical occurrence associated with the use of a drug in humans, whether or not considered drug related.

Severity of Event: All AEs will be assessed by the clinician using a protocol-defined grading system. For events not included in the protocol-defined grading system, the following guidelines will be used to quantify intensity.

- Mild (Grade 1): Events require minimal or no treatment and do not interfere with the participant's daily activities.
- Moderate (Grade 2): Events result in a low level of inconvenience or concern with the therapeutic measures. Moderate events may cause some interference with functioning.
- Severe (Grade 3): Events interrupt a participant's usual daily activity and may require systemic drug therapy or other treatment. Severe events are usually incapacitating.
- Life-threatening (Grade 4): Life-threatening consequences; urgent intervention indicated.

Changes in the severity of an AE should be documented to allow an assessment of the duration of the event at each level of intensity to be performed. AEs characterized as intermittent require documentation of onset and duration of each episode.

Relationship to Study Products: The clinician's assessment of an AE's relationship to test article is part of the documentation process, but it is not a factor in determining what is or is not reported in the study. If there is any doubt as to whether a clinical observation is an AE, the event should be reported. All AEs must have their relationship to study product assessed using the terms: related or not related. In a clinical trial, the study product must always be suspect. To help assess, the following guidelines are used.

- Related: There is a reasonable possibility that the study product caused the AE. Reasonable possibility means that there is evidence to suggest a causal relationship between the study product and the AE.
- Not Related: There is not a reasonable possibility that the administration of the study product caused the event.

9.2.2 Serious Adverse Events

An AE or SAE reaction is considered "serious" if, in the view of either the investigator or sponsor, it results in any of the following outcomes:

- Death
- A life-threatening adverse event*

- Inpatient hospitalization or prolongation of existing hospitalization
- A persistent or significant incapacity or substantial disruption of the ability to conduct normal life functions
- A congenital anomaly/birth defect.
- Important medical events that may not result in death, be life-threatening, or require hospitalizations may be considered serious when, based upon appropriate medical judgment they may jeopardize the participant and may require medical or surgical intervention to prevent one of the outcomes listed in this definition. Examples of such medical events include allergic bronchospasm requiring intensive treatment in an emergency room or at home, blood dyscrasias or convulsions that do not result in inpatient hospitalization, or the development of drug dependency or drug abuse.
- *Life-threatening adverse event. An AE is considered "life-threatening" if, in the view of either the investigator or sponsor, its occurrence places the participant at immediate risk of death. It does not include an AE that, had it occurred in a more severe form, might have caused death.

All SAEs will be:

- Recorded on the appropriate SAE form and CRF
- Followed through resolution by a study physician
- Reviewed and evaluated by the study chairs, relevant IRBs, and the DSMB

9.2.3 Procedures to be followed in the Event of Abnormal Laboratory Test Values or Abnormal Clinical Findings

The site principal investigator or appropriate sub-investigator is responsible for reporting all AE/SAEs that are observed or reported during the study, regardless of their relationship to study product. AE/SAEs, abnormal laboratory values, or abnormal clinical findings will be documented, reported, and followed appropriately.

For grading abnormal gynaecological events, refer to Appendix B, Division of AIDS Table for Grading the Severity of Adult and Pediatric Adverse Events, December 2004; Addendum 1: Female Genital Grading Table for Use in Microbicide Studies, November 2007.

For grading abnormal laboratory and clinical events, refer to Appendix C, DAIDS Toxicity Table for Grading the Severity of Adult and Pediatric Adverse Events, Version 2.1 July 2017.

9.3 Reporting Procedures

9.3.1 Serious Adverse Events

AEs will be followed until resolution even if this extends beyond the study- reporting period. Resolution of an AE is defined as the return to pretreatment status or stabilization of the condition with the expectation that it will remain chronic.

Any AE that meets a protocol-defined serious criterion must be submitted immediately (within 24 hours of site awareness) on an SAE form to the following address:

BIOMEDICAL RESEARCH ETHICS COMMITTEE (BREC)
University of KwaZulu-Natal
(UKZN) Research Office, Westville
Campus Govan Mbeki Building
Private Bag X 54001
Durban 4000
KZN, SOUTH AFRICA
Tel: 27 31 2604769
Email: BREC@ukzn.ac.za

Any AE that meets a protocol-defined serious criterion must also be submitted within 7 days of site awareness) on an SAE form to the following address:

Chief Executive Officer
South African Health Products Regulatory
Authority Clinical Trials Unit
Private Bag X828
Pretoria
ctcsaes@sahpra.org.za

Other supporting documentation of the event may be requested by the study sponsor or the DSMB and should be provided as soon as possible.

The medical monitor and clinical protocol manager will be notified of the SAE by the site. The medical monitor will review and assess the SAE for regulatory reporting and potential impact on study subject safety and protocol conduct.

At any time during the study, if the investigator becomes aware of an SAE that is suspected to be related to study product, the investigator will report the event to the IRB at UKZN, UCSF and the Ragon Institute of MGH, MIT and Harvard.

9.3.2 Regulatory Reporting

Following notification from the site principal investigator or appropriate sub-investigator, the study sponsor, will report any suspected adverse reaction that is both serious and unexpected. UCSF will report an AE as a suspected adverse reaction only if there is evidence to suggest a causal relationship between the drug and the AE. UCSF will notify SAHPRA in a safety report of potential serious risks from clinical trials or any other source, as soon as possible, but in no case later than 15 calendar days after the sponsor determines that the information qualifies for reporting as specified in 21 CFR Part 312.32. UCSF will also notify SAHPRA of any unexpected fatal or life- threatening suspected adverse reaction as soon as possible, but in no case later than 7 calendar days after the sponsor's initial receipt of the information. Relevant follow-up information to a safety report will be submitted as

soon as the information is available. Upon request from SAHPRA, UCSF will submit to SAHPRA any additional data or information that the agency deems necessary, as soon as possible, but in no case later than 15 calendar days after receiving the request.

9.3.3 Reporting of Pregnancy

Pregnancies occurring in study participants will be reported on the Pregnancy Report form. Efforts will be made to follow all pregnancies reported during the course of the study to pregnancy outcome pending the participant's permission.

9.4 Type and Duration of Follow-up of Subjects after Adverse Events

AEs and SAEs will be followed from the time of study treatment through resolution even if this extends beyond the study-reporting period. Resolution of an AE/SAE is defined as the return to pretreatment status or stabilization of the condition with the expectation that it will remain chronic.

Follow-up procedures, evaluations, and outcomes will be recorded on the appropriate data collection form.

9.5 Halting Rules

Study enrolment and dosing will be halted and an ad hoc DSMB review will be performed if any of the following occur at any time during the study:

1) One or more participants experience a treatment-related SAE.
2) Two or more participants experience treatment-related vulvar and/or vaginal ulceration, abscess, or necrosis.
3) Two or more participants experience a treatment-related severe (Grade 3) or life-threatening (Grade 4) systemic adverse event.
4) An overall pattern of symptomatic, clinical, or laboratory events that the monitor or DSMB consider associated with study product and that may collectively represent a serious potential concern for safety.

A decision to reinitiate the study and proceed with study treatments will be made based on the recommendation of the DSMB.

9.6 Safety Oversight (DSMB)

Safety oversight will be under the direction of a DSMB, consisting of 3 voting members. The DSMB will meet once - prior to first enrollment, once - after half of the 60 participants complete the Follow-Up Study Visit 11(Day 36), and then, finally – once, after study conclusion and database lock, to assess safety and efficacy data in each arm of the study.

The DSMB will review aggregate safety data for increased rate of occurrence of serious suspected adverse reactions. If halting rules are initiated, more frequent meetings may be held. The DMSB will operate under the rules of an approved charter that will be written at the organizational meeting of the DSMB. At this time, each data element that the DSMB needs to assess will be clearly defined. The DSMB will advise UKZN, UCSF and the Ragon Institute at MGH of its findings.

10.0 MONITORING

10.1 Site Monitoring Plan

Site monitoring is conducted to ensure that the human subject protections, study and laboratory procedures, study intervention administration, and data collection processes are of high quality and meet sponsor, ICH/GCP guidelines and applicable regulations, and that the study is conducted in accordance with the protocol, protocol-specific MOP and applicable sponsor standard operating procedures. The sponsoring agency, or its designee will conduct site-monitoring visits as detailed in the clinical monitoring plan.

Site visits will be made at standard intervals as defined by the sponsor and may be made more frequently as directed by the sponsor. Monitoring visits will include, but are not limited to, review of regulatory files, accountability records, CRFs, informed consent forms, medical and laboratory reports, and protocol and GCP compliance. Site monitors will have access to the study site, study personnel, and all study documentation according to the sponsor-approved site monitoring plan. Study monitors will meet with site principal investigators to discuss any problems and actions to be taken and document visit findings and discussions.

\

11.0 STATISTICAL CONSIDERATIONS

11.1 Introduction

This is a Phase 2 randomized double-blind placebo-controlled trial to assess the impact of the live biotherapeutic product LACTIN-V containing *Lactobacillus crispatus* CTV-05 on the non-l*actobacillus* dominant microbiome of young women in South Africa at high risk of HIV acquisition.

The study plans to enrol and randomize 60 sexually experienced women age 18 to 23 years, who are using a reliable method of long- acting injectable birth control or are of non-childbearing potential (permanently sterile). After enrolment, women will start a standard 7 -day course of oral Metronidazole tablets. After completing the 7-day course of Metronidazole, subjects will be randomized to receive either placebo or LACTIN-V in a 1:2 ratio. The participants will receive protocol treatment for four weeks and will have four weeks of additional off treatment follow-up time.

11.2 Study Objectives and Outcome Measures

As described in section 3.2 and 3.3, the primary study objectives are to compare LACTIN-V with placebo following a 7-day treatment course with Metronidazole with respect to safety and impact on the vaginal microbiome, specifically *lactobacillus* growth and decrease in pro-inflammatory cytokines and HIV target cells.

In Objective 1, the outcomes of interest will be:
(1) a decrease in genital tract inflammation from baseline (the FRESH Study Visit before enrollment) to Day 36 Follow-Up Visit, as defined by a ≥ 1 Log_{10} decrease in the concentration of cytokines that have been associated with an increased risk for HIV infection.[17] , and
(2) the change in the concentration of cytokines at the Final Visit on Day 64 (4 weeks after dosing phase completed) compared to baseline, number of endocervical $CD4^+$ HIV target cells at Day 36 Follow-Up and Day 64 Final Visits compared to baseline, and in vitro anti-HIV activity of cervicovaginal lavage at Day 36 Follow-Up and Day 64 Final Visits. We will compare each outcome by study arm.

In Objective 2, the outcomes of interest will be:
(1) the presence of *Lactobacillus*-dominant vaginal microbial cervicotypes at the Day 36 Follow-Up Visit, and following the post-dosing phase at the Day 64 Final Visit in the LACTIN-V arm; and
(2) the comparison of *L. crispatus* vs. *L. iners* prevalence between treatment groups, quantity of vaginal *L. crispatus*, presence of the specific CTV-05 *L. crispatus* strain, persistence of colonization after stopping therapy, effect of sex and/or vaginal hygiene practices on colonization by lactobacilli, and stability of vaginal microbial communities over time in the treatment vs. placebo arms.

In Objective 3, the outcomes of interest will be:
(1) the proportion of treatment related adverse events study arm, in particular Grade 3 AEs; and
(2) the proportion of participants finding the product acceptable and are willing to use it

\

should it become commercially available, as measured in a standardized acceptability questionnaire.

11.3 Sample Size Considerations

The study will accrue a total of 60 randomized subjects (n=20 for the placebo arm A and n=40 for the LACTIN-V arm B). The accrual for this study is expected to be completed within 75 weeks (18 months) with projected accrual rate of 3 subjects per month. Each subject will be followed up for 64 days.

The primary endpoint upon which the sample size calculation was based is the proportion of subjects with a ≥1 Log_{10} decrease in at least 3 of 9 proinflammatory cytokines in CVL specimens on the Day 36 Visit, i.e. at the completion of the LACTIN-V/Placebo dosing phase. The proportion of women with decreased inflammation will be compared between the two study arms. This endpoint is adapted from the CAPRISA 004 study which demonstrated that women with the highest levels of at least 3 of 9 proinflammatory cytokines in CVL specimens had an increased risk of HIV acquisition.[25] Our adaptation of the CAPRISA 004 analysis is that we will define "decreased inflammation" as a ≥1 Log_{10} decrease in the concentration of at least 3 of the same 9 proinflammatory cytokines. Based on our prior data,[2, 42] we assume that no more than 10% of placebo-treated women will achieve a decrease in ≥3 of the 9 proinflammatory genital tract cytokines. Moreover, we hypothesize that at least half of the participants in the treatment arm will achieve a decrease ≥1 log_{10} in ≥3 of the 9 proinflammatory genital tract cytokines. With the targeted sample size of 60 randomized women (1/3 in placebo arm & 2/3 in the LACTIN-V arm; there will be more than 88% power to detect a 40% absolute difference (10% vs. 50%) in the proportion of women with decreased inflammation between the two treatment arms. This power calculation was based on simulation study using 10,000 Monte Carlo samples and two-sided Fisher's exact test with type 1 error rate of 5%.

11.4 Safety Analysis

This study will be monitored for safety and to determine if any of the safety halting rules described in detail in Section 9.5 are met (one or more treatment-related SAE, two or more treatment-related vaginal ulceration, two or more treatment-related severe Grade 3 systemic adverse events). Thus, all patients will be evaluated for toxicity. The DSMB will review interim analyses comparing product-related AEs between the two treatment arms will be performed after half of the 60 participants complete the Follow-Up Study Visit 11, and then annually. Reports of these analyses will be sent to the Principal Investigator or Senior Investigators at the participating institutions.

The table below gives the probability of observing at least one incidence of AE together with the corresponding true AE rate for a total of 60 participants.

True AE rate:	0.5%	1%	2%	5%
P [AE>1]:	0.26	0.45	0.70	0.95

For example, the probability of observing at least one incidence of AE with true rate of 5% is 0.95.

\

11.5. Statistical Analysis Plan

The primary analyses in Objective 1 is to compare the levels of i) pro-inflammatory genital tract cytokines levels & proportion of subjects with a significant decrease (≥1 $\log_{10}$) in cytokines levels, ii) HIV target cells (i.e., CD4+/CCR5+/HLA-DR+/CD38+ T cells) and iii) anti-HIV effect of CVL, as assessed by HIV p24 levels, between the two treatment arms.

Graphical and descriptive measures (such as frequency, percent mean, median, standard deviation and IQR) will be used to summarize data. Spearman rank correlation will be used to examine associations among the aforementioned outcomes. Statistical tests (such as the two sample T and Wilcoxon rank sum tests) will be used to compare levels of cytokines, HIV p24 levels and HIV target cells between the two treatment groups.

For within group comparison, statistical tests (such as paired T and Wilcoxon signed rank tests) will be used. Longitudinal data analyses using nonlinear mixed models or generalized estimating equations will be performed to examine and compare the overtime changes in cytokines levels and HIV target cells. Fisher's exact test will be used to compare the proportion of subjects with a significant decrease (≥1 $\log_{10}$) in cytokines levels.

Data classification and dimension reduction techniques (e.g., principal component analysis, and cluster analysis) will be used to group a set of cytokines or HIV target cells to uncorrelated summary variables or to identify study subjects with similar cytokine or HIV target cell profiles. Adjustment will be made for multiple comparisons using less conservative multiple testing correction procedures (such as the Holm's and Benjamini-Hochberg methods) where appropriate.

The primary analysis in Objective 2 is to compare the presence and abundance of *Lactobacillus spp*. (including *L. crispatus* CTV-05) between the two study groups. As in Objective 1, graphical and descriptive measures will be used to summarize data. Fisher's exact test will be used to compare presence of specific strains of vaginal lactobacilli and cervicotypes (CT).[1] Empirical Bayes method will be used for identification of microbes that show differential abundance between study arms. Empirical Bayes method is more efficient due to its ability of pooling information across microbes.

Sparse clustering, where clustering and feature selection are integrated, will be used to identify study subjects with similar microbial profiles. Sparse clustering has a number of advantages. If the underlying groups differ only in terms of some of the features, then it might result in more accurate identification of these groups than standard clustering. It also yields interpretable results, since one can determine precisely which features are responsible for the observed differences between the groups or clusters. Similarly, Sparse Principal Component analysis will be used to group a set of highly correlated microbial reads to uncorrelated summary variables. As in Objective 1, adjustment for multiple testing will be made using less conservative multiple testing correction methods (e.g., Benjamini-Hochberg).

Solicited AEs will be analyzed by taking the most severe response over the Day 64 follow-up period. The analyses of AEs and SAEs is mainly descriptive. Descriptive measures (frequency and percent) will be used to summarize the proportion of participants with product-related AEs (Grade 0, Grade 1, Grade 2, and Grade 3) in each treatment arm. Confidence intervals for AEs rates will be estimated using methods for exact binomial confidence intervals. Fisher's exact test will be used to compare rates of AEs between the

\

two treatment arms. Multivariate analyses using logistic regression models will be used to compare AEs rates between the two arms while adjusting for the effect of other covariates.

Unsolicited AEs will be coded by MedDRA® for preferred term and system organ class. The proportion of participants and exact 95% confidence intervals of AEs in aggregate, as well as by MedDRA® categories, will be computed. The number of SAEs will be reported by a detailed listing showing the type, MedDRA® coding, relevant dates (treatment dosing dates and AE onset and resolution dates), severity, relatedness, and outcome for each event.

12.0 SOURCE DOCUMENTS AND ACCESS TO SOURCE DATA / DOCUMENTS

Each participating site will maintain appropriate medical and research records for this trial, in compliance with ICH E6, Section 4.9 and regulatory and institutional requirements for the protection of confidentiality of participants. Each site will permit authorized representatives of UKZN BREC, its designees, and appropriate regulatory agencies such as SAHPRA to examine (and when required by applicable law, to copy) clinical records for the purposes of quality assurance reviews, audits, and evaluation of the study safety and progress. These representatives will be permitted access to all source data, which include, but are not limited to, hospital records, clinical and office charts, laboratory notes, memoranda, evaluation checklists, pharmacy dispensing records, recorded data from automated instruments, copies or transcriptions certified after verification as being accurate and complete, microfiches, photographic negatives, microfilm or magnetic media, x-rays, and participant files and records kept at the pharmacy, at the laboratories, and medico-technical departments involved in the clinical trial. Data collection forms will be derived from the CRFs and be provided by the UCSF study team in collaboration with the site and DF Net Data Management.

13.0 QUALITY CONTROL AND QUALITY ASSURANCE

The data management company DF Net Research, Inc. will implement quality control procedures beginning with the data entry system and generate data quality control checks that will be run on the database. Any missing data or data anomalies will be communicated to the site(s) for prompt clarification and resolution.

The investigational site is responsible for conducting routine quality control (QC) and quality assurance (QA) activities to internally monitor study progress and protocol compliance. A Clinical Quality Management Plan (CQMP) is in place for ensuring compliance with the protocol, applicable federal regulations, and Good Clinical Practice guidelines.

The Principal Investigator will provide direct access to the trial-related site, source data/documents, and reports for the purpose of monitoring and auditing by the sponsor, and inspection by local and regulatory authorities.

The Principal Investigator will ensure all study personnel are appropriately trained and applicable documentations are maintained on site.

14.0 ETHICS/PROTECTION OF HUMAN SUBJECTS

14.1 Ethical Standard

The investigator will ensure that this study is conducted in full conformity with the principles set forth in the Belmont Report: Ethical Principles and Guidelines for the Protection of Human Subjects of Research of the US National Commission for the Protection of Human Subjects of Biomedical and Behavioral Research (April 18, 1979) and codified in 45 CFR Part 46 and/or the ICH E6; 62 Federal Regulations 25691 (1997).

14.2 Institutional Review Board

Each participating institution will provide for the review and approval of this protocol and the associated informed consent documents and recruitment material by an appropriate independent ethics committee (IEC) or IRB registered with the OHRP. Any amendments to the protocol or consent materials will also be approved before they are placed into use.

14.3 Informed Consent Process

Informed consent is a process that is initiated prior to the participant's agreeing to participate in the study and continuing throughout the participant's study participation. Extensive discussion of risks and possible benefits of this therapy will be provided to the participant and their families.

The study coordinator will approach individual FRESH study participants about joining the parallel LACTIN-V study. The FRESH cohort has included sub-studies before (additional blood draws, high-frequency genital mucosal sampling), thus we anticipate that the overall idea will be acceptable and not stigmatizing to women enrolled in the FRESH cohort. Probiotics for gut health are relatively common over-the-counter products in South Africa. Thus, while less information is available about using 'probiotic-like' products in the vagina, we do not anticipate significant challenges with recruitment of FRESH participants into the LACTIN-V study.[74]

The consent form describing in detail the study interventions, products, study procedures, and risks are given to the participant and written documentation of informed consent is required prior to starting intervention or administering study product. The consent form will be IRB-approved (at UKZN BREC, UCSF CHR and Ragon Institute IRB) and the participant will be asked to read and review the document. Upon reviewing the document, the investigator will explain the research study to the participant and answer any questions that may arise. The participants will sign the informed consent document available in English and isiZulu prior to any procedures being done specifically for the study. The participants should have the opportunity to discuss the study with their surrogates or think about it prior to agreeing to participate. The participants may withdraw consent at any time throughout the course of the trial. An Assessment of Understanding form, available in English and isiZulu, will be completed to ensure that study participants retained the most important aspects of the provided information.
A copy of the informed consent document will be given to the participants for their records.

The rights and welfare of the participants will be protected by emphasizing to them that the quality of their medical care will not be adversely affected if they decline to participate in this study.

14.4 Exclusion of Women, Minorities, and Children (Special Populations)

This trial will enrol adult women who meet the participant inclusion criteria regardless of religion or ethnic background.

The following populations will be excluded from study participation:

Pregnant women
Pregnant women are not eligible for this study because there are no current recommendations for the use of LACTIN-V during pregnancy.

Men
Men are not eligible for this study as the study evaluates the uterine cervix and vagina for changes associated with the use of LACTIN-V.

Children
Children under the age of 18 are not eligible for this study, which requires participants to be sexually experienced.

14.5 Subject Confidentiality

Participant confidentiality is strictly held in trust by the participating investigators, their staff, and the sponsor(s) and their agents. This confidentiality is extended to cover testing of biological samples and genetic tests in addition to the clinical information relating to participating participants.

The study protocol, documentation, data, and all other information generated will be held in strict confidence. No information concerning the study, or the data will be released to any unauthorized third party without prior written approval of the sponsor.

The study monitor or other authorized representatives of the sponsor may inspect all documents and records required to be maintained by the investigator, including but not limited to, medical records (office, clinic, or hospital) and pharmacy records for the participants in this study. The clinical study site will permit access to such records.

14.6 Study Discontinuation

If the trial is discontinued, participants who sign the informed consent form, and are randomized and treated will continue to be followed for safety assessments. No further study product will be administered.

15.0 DATA HANDLING AND RECORD KEEPING

The investigator is responsible for ensuring the accuracy, completeness, legibility, and timeliness of the data reported.

Data collection forms will be derived from the CRFs to record and maintain data for each participant enrolled in the study. All source documents should be completed in a neat, legible manner to ensure accurate interpretation of data. Permanent ink is required to ensure clarity of reproduced copies. When making a change or correction, the original entry should be crossed out with a single line, and the change should be initialed and dated. Do not erase, overwrite, or use correction fluid or tape on the original.

Data reported in the CRF should be consistent with the data collection form/source documents or the discrepancies should be documented.

The sponsor and/or its designee will provide guidance to investigators on making corrections to the data collection forms and CRFs.

15.1 Data Management Responsibilities

All source documents and laboratory reports must be reviewed by the clinical team and data entry staff, who will ensure that they are accurate and complete. Adverse events must be graded, assessed for severity and causality, and reviewed by the site principal investigator (PI) or designee.

Data collection is the responsibility of the clinical trial staff at the site under the supervision of the site PI. During the study, the investigator must maintain complete and accurate documentation for the study.

DF Net Research Inc. will serve as the Statistical and Data Coordinating Center for this study and will be responsible for data management, quality review, analysis, and reporting of the study data.

15.2 Data Capture Methods

Clinical data (including, but not limited to, AE/SAEs, concomitant medications, and reactogenicity data) and clinical laboratory data will be entered into a 21 CFR Part 11-compliant Internet Data Entry System, provided by DF Net Research Inc. The data system includes password protection and internal quality checks, such as automatic range checks, to identify data that appear inconsistent, incomplete, or inaccurate.

Clinical data will be entered directly from the source documents.

15.3 Types of Data

Data for this study will include clinical, efficacy, safety, laboratory and outcome measures.

15.4 Timing/Reports

A final report will be prepared following the availability of all the safety and laboratory data. Interim statistical reports may be generated as deemed necessary and appropriate by the sponsor. Safety and laboratory data summary reports may be generated for the DSMB.

15.5 Study Records Retention

Study documents should be retained for a minimum of 2 years after the last approval of a marketing application in an ICH region and until there are no pending or contemplated marketing applications in an ICH region or at least 2 years have elapsed since the formal discontinuation of clinical development of the investigational product. These documents should be retained for a longer period, however, if required by local regulations. No records will be destroyed without the written consent of the sponsor, if applicable. It is the responsibility of the sponsor to inform the investigator when these documents no longer need to be retained.

15.6 Protocol Deviations

A protocol deviation is any noncompliance with the clinical trial protocol, Good Clinical Practice (GCP), or MOP requirements. The noncompliance may be either on the part of the participant, the investigator, or the study site staff. As a result of deviations, corrective actions are to be developed by the site and implemented promptly.

These practices are consistent with ICH E6:

4.5 Compliance with Protocol, sections 4.5.1, 4.5.2, and 4.5.3

5.1 Quality Assurance and Quality Control, section 5.1.1

5.20 Noncompliance, sections 5.20.1, and 5.20.2. I

It is the responsibility of the site to use continuous vigilance to identify and report deviations within 5 working days of identification of the protocol deviation, or within 5 working days of the scheduled protocol-required activity. All deviations must be promptly reported to the sponsor.

All deviations from the protocol must be addressed in study subject data collection forms. A completed copy of the Protocol Deviation Form must be maintained in the regulatory file, as well as in the participant's source document. Protocol deviations must be sent to the local IRB/IEC per their guidelines. The site PI/study staff is responsible for knowing and adhering to their IRB/IEC requirements

16.0 PUBLICATION POLICY

Following completion of the study, the Investigator is expected to publish the results of this research in a scientific journal. Publication of the results of this study will be governed by sponsor policies and the International Committee of Medical Journal Editors (ICMJE) member journals, which have adopted a trials-registration policy as a condition for publication.

This policy requires that all clinical trials be registered in a public trials registry such as ClinicalTrials.gov which is sponsored by the National Library of Medicine as well as the South African National Clinical Trials Registry. Other biomedical journals are considering adopting similar policies. It is the responsibility of the sponsor to register this trial in an acceptable registry. Any clinical trial starting enrolment after 01 July 2005 must be registered on or before patient enrolment. For trials that began enrolment prior to this date, the ICMJE member journals will require registration by 13 September 2005, before considering the results of the trial for publication.

The ICMJE defines a clinical trial as any research project that prospectively assigns human participants to intervention or comparison groups to study the cause-and-effect relationship between a medical intervention and a health outcome. Studies designed for other purposes, such as to study pharmacokinetics or major toxicity (e.g., Phase 1 trials), would be exempt from this policy.

Any presentation, abstract, or manuscript will be made available by the Investigator to the sponsor, and Osel Inc. for review prior to submission.

17.0 SPONSOR INDEMNIFICATION FOR SITES AND INVESTIGATORS

In consideration of the sites, the Aurum Institute - Pharmacy and Females Rising Through Education, Support and Health, participation in the study, we shall indemnify and hold harmless the Aurum Institute Pharmacy and Females Rising Through Education, Support and Health and its employees from any legal liability for costs or damages for death or personal injury which may result from the administration of LACTIN-V (*Lactobacillus crispatus CTV-05*) pursuant to the said study. This indemnity does not apply to the extent that such death or personal injury arises out of any negligent act, default or omission of the Aurum Pharmacy and Females Rising Through Education, Support and Health participation in the study, we shall indemnify and hold harmless the Aurum Institute or its employees. Furthermore, this indemnity is subject to the condition that the study is carried out in accordance with the Protocol approved by us in writing, that University of California, San Francisco (UCSF) is notified immediately on receipt of any claim, that University of California, San Francisco (UCSF shall have full control of the management and defence of any such claim and that no offer to compromise or settle any claim is made without the written agreement of University of California, San Francisco (UCSF).

18.0 LITERATURE REFERENCES

1. UNAIDS Press Release. 2017. UNAIDS announces nearly 21 million people living with HIV now on treatment. http://www.unaids.org/en/resources/presscentre/pressreleaseandstatementarchive/2017/november/20171121_righttohealth_report. Accessed 09-06-2018.
2. Cohen CR, Duerr A, Pruithithada N, Rugpao S, Hillier S, Garcia P, Nelson K. Bacterial vaginosis and HIV seroprevalence among female commercial sex workers in Chiang Mai, Thailand. Aids.1995;9(9):1093-1097. PMID: 8527084
3. Wira CR, Fahey JV. A new strategy to understand how HIV infects women: identification of a window of vulnerability during the menstrual cycle. AIDS. 2008 Oct 1;22(15):1909-17. PMCID: PMC2647143
4. Bearinger LH, Sieving RE, Ferguson J, Sharma V. Global perspectives on the sexual and reproductive health of adolescents: patterns, prevention, and potential. Lancet. 2007;369(9568):1220-1231. PMID: 17416266
5. Pettifor AE, Measham DM, Rees HV, Padian NS. Sexual power and HIV risk, South Africa. Emerg Infect Dis. 2004;10(11):1996-2004. PMCID: PMC3328992.
6. Kharsany AB1, Frohlich JA, Yende-Zuma N, Mahlase G, Samsunder N, Dellar RC, Zuma-Mkhonza M, Abdool Karim SS, Abdool Karim Q. Trends in HIV Prevalence in Pregnant Women in Rural South Africa. J Acquir Immune Defic Syndr. 2015 Nov 1;70(3):289-95. doi: 10.1097/QAI.0000000000000761. PMID: 26186507
7. Klatt NR, Cheu R, Birse K, Zevin AS, Perner M, Noël-Romas L, Grobler A, Westmacott G, Xie IY, Butler J, Mansoor L, McKinnon LR, Passmore JS, Abdool Karim Q, Abdool Karim SS, Burgener AD. Vaginal bacteria modify HIV tenofovir microbicide efficacy in African women. Science. 2017 Jun 2;356(6341): 938-945. PMID: 28572388
8. Ramjee G. Microbicide research: current and future directions. Curr Opin HIV AIDS. 2010;5(4):316-321. PMID: 20543607
9. Romano J, Malcolm RK, Garg S, Rohan LC, Kaptur PE. Microbicide delivery: formulation technologies and strategies. Curr Opin HIV AIDS. 2008;3(5):558-566. PMID: 19373022
10. Vail JG, Cohen JA, Kelly KL. Improving topical microbicide applicators for use in resource-poor settings. Am J Public Health. 2004;94(7):1089-1092. PMCID: PMC1448402
11. Baeten JM, Donnell D, Ndase P, Mugo NR, Campbell JD, Wangisi J et al. Antiretroviral prophylaxis for HIV prevention in heterosexual men and women. New England Journal of Medicine 2012 Aug; 367(5): 399-410. doi: 10.1056/NEJMoa1108524. PMID: 22784037
12. Heffron R, McClelland RS, Baljus JE, Celum C, Cohen CR, Mugo N et al. Efficacy of oral Pre-exposure prophylaxis (PrEP) for HIV among women with abnormal vaginal microbiota: a post-hoc analysis of the randomised placebo-controlled partners PrEP study. doi: 10.1016/S2352-3018(17)30110-8. PMID: 28732773
13. Abdool Karim Q, Abdool Karim SS, Frohlich JA, et al. Effectiveness and safety of tenofovir gel, an antiretroviral microbicide, for the prevention of HIV infection in women. Science. 2010;329:1168–1174. PMID: 20643915
14. Marrazzo JM, Ramjee G, Richardson BA, Gomez K, Mgodi N, Nair G et al. Tenofovir-based preexposure prophylaxis for HIV infection among African women. New England Journal of Medicine 2015 Feb;372(6) 509-18. doi: 10.1056/NEJMoa1402269. PMID 25651245
15. Van Damme L, Corneli A, Ahmed K, Agot K, Lombaard J, Kapiga S, et al. Preexposure prophylaxis for HIV infection among African women. New England Journal of Medicine 2012 Aug ;367(5): 411-22. doi: 10.1056/NEJMoa1202614. PMID: 22784040
16. Dai JY, Hendrix CW, Richardson BA, Kelly C, Marzinke M, Chirenje ZM et al. Pharmacological measures of treatment adherence and risk of HIV infection in theVOICE

study. Journal of Infectious Disease 2016 Feb; 213(3): 335-42. doi: 10.1093/infdis/jiv333. PMID: 26123563
17. McKinnon LR, Liebenberg LJ, Yende-Zuma N, Archary D, Ngcapu S, Sivro A, Nagelkerke N, Garcia Lerma JG, Kashuba AD, Masson L, Mansoor LE, Karim QA, Karim SSA, Passmore JS. Genital inflammation undermines the effectiveness of tenofovir gel in preventing HIV acquisition in women. Nat Med. 2018 May;24(4):491-496. doi: 10.1038/nm.4506. Epub 2018 Feb 26.
18. UNAIDS. Ending AIDS: progress towards the 90–90–90 targets. http://www.unaids.org/en/resources/documents/2017/20170720_Global_AIDS_update_201 7. Accessed 09-06-2018.
19. D. Havlir; E. Charlebois; L. Balzer; T.; C. Cohen; E. Bukusi; M. Petersen; M. Kamya and SEARCH Collaboration. SEARCH community cluster randomized study of HIV “test and treat” using multi- disease approach and streamlined care in rural Uganda and Kenya. Abstract WEAX0106LB. JIAS 2018 supplement, page 168. https://onlinelibrary.wiley.com/doi/epdf/10.1002/jia2.25148. Accessed 09-06-2018.
20. PEPFAR. Adolescent Girls & Women. https://www.pepfar.gov/priorities/girlswomen/index.htm. Accessed 09-06-2018.
21. Sewankambo N, Gray RH, Wawer MJ, PaxtonL, McNaim D Wabwire-Mangen F et al. HIV- 1 infection associated with abnormal vaginal flora morphology and bacterial vaginosis. Lancet 1997 Aug; 350(9077): 546-50. PMID 9284776
22. Cohen C, Duerr A, Pruithithada N, Rugpao S, Sungwal G, Patricia N et al. Bacterial vaginosis and HIV seroprevalence among female commercial sex workers in Chiang Mai, Thailand. AIDS 1995 Sept;9(9). PMID 9764791.
23. Taha TE, Hoover DR, Dallabetta GA, Kumwenda NI, Mtimavalye LA, Yang LP, Liomba GN, Broadhead RL, Chiphangwi JD, Miotti PG. Bacterial vaginosis and disturbances of vaginal flora: association with increased acquisition of HIV. AIDS. 1998 Sep 10;12(13):1699-706. PMID:9764791
24. Martin HL, Richardson BA, Nyange PM, Lavreys L, Hillier SL, Chohan B, et al. Vaginal lactobacilli, microbial flora, and risk of human immunodeficiency virus type 1 and sexually transmitted disease acquisition. Journal of Infectious Disease 1999 Dec; 180(6): 1863-8. PMID: 10558942 DOI: 10.1086/315127
25. Haddad LB, Wall KM, Kilembe W, Vwalika B, Khu NH, Brill I et al. Bacterial vaginosis modifies the association between hormonal contraception and HIV acquisition. AIDS 2018 Mar; 32(5): 595-604. doi: 10.1097/QAD.0000000000001741. PMID: 29334545
26. Kinuthia J, Drake AL, Matemo D, Richardson BA, Zeh C, Osborn L et al. HIV acquisition during pregnancy and post-partum is associated with genital infections and partnership characteristics. AIDS 2015 Sep 24; 29(15): 2025-33. doi: 10.1097/QAD.0000000000000793. PMID: 26352880
27. Van de Wijgert JH, Morrison CS, Cornelissee PG, Munjoma M, MoncadaJ, Awio P et al. Bacterial vaginosis and vaginal yeast, but not vaginal cleansing increase HIV-1 acquisition in African women. Journal of Acquired Immune Deficiency Syndrome 2008 Jun; 48(2): 203-10. doi: 10.1097/QAI.0b013e3181743936. PMID18520679
28. Frank DN, Manigart O, Leroy V, Meda N, Valea D, Zhang W et al. Altered vaginal microbiota are associated with perinatal mother-to child transmission of HIV in African women from Burkina Faso. Journal of Acquired Immune Deficiency Syndrome 2012 Jul; 60(3): 299-306. doi: 10.1097/QAI.0b013e31824e4bdb. PMID: 22343176
29. Cohen CR, Lingappa JR, Baeten JM, Ngayo MO, Spiegel CA, Hong T et al. Bacterial vaginosis associated with increased risk of female-to-male HIV-1 transmission: a prospective cohort analysis among African couples. PLOS Medicine 2012 Jun; 9(6):e1001251. doi: 10.1371/journal.pmed.1001251. PMID: e22745608

30. McClelland RS, Lingappa JR, Srinivasan S, Kinuthia J, John-Stewart GC, Jaoko W, Richardson BA, Yuhas K, Fiedler TL, Mandaliya KN, Munch MM, Mugo NR, Cohen CR, Baeten JM, Celum C, Overbaugh J, Fredricks DN. Evaluation of the association between the concentrations of key vaginal bacteria and the increased risk of HIV acquisition in African women from five cohorts: a nested case-control study. Lancet Infect Dis. 2018 May;18(5):554-564. doi: 10.1016/S1473-3099(18)30058-6. Epub 2018 Jan 26. PMID: 29396006.
31. Gosmann C, Anahtar MN, Handley SA, Farcasanu M, Abu-Ali G, Bowman BA et al. Lactobacillus-Deficient cervicovaginal bacterial communities are associated with increased HIV acquisistion in young South African Women. Immunity 2017 Jan; 46(1): 29-37. doi: 10.1016/j.immuni.2016.12.013. PMID: 28087240
32. Masson L, Passmore JA, Liebenberg LJ, Werner L, Baxter C, Arnold KB et al. Genital inflammation and the risk of HIV acquisition in women. Clinical Infectious Disease Jul; 61(2). doi: 10.1093/cid/civ298. PMID: 25900168
33. Anahtar MN, Byrne EH, Doherty KE, Bowman BA, Yamamoto HS, Soumillon M, et al. Cervicovaginal bacteria are a major modulator of host inflammatory responses in the female genital tract. Immunity 2015 May 19;42(5):965-76. doi: 10.1016/j.immuni.2015.04.019. PMID: 25992865
34. Thurman A, Kimble T, Herold B, Mesquita P, Fichorova R, Dawood H et al. Bacterial Vaginosis and subclinical markers of genital tract inflammation and mucosal immunity. ARHR 2015 Nov; 31(11): 1139-1152. doi: 10.1089/aid.2015.0006 PMID: 26204200
35. Mitchell C, Fredricks D, Agnew K, Hitti J. Hydrogen peroxide-producing Lactobacilli are associated with lower levels of vaginal interleukin-1B, independent of bacterial vaginosis. Sexually transmitted disease 2015 Jul; 42(7): 358-63. doi: 10.1097/OLQ.0000000000000298 PMID: 26222747
36. Nahui Palomino RA, Zicari S, Vanpouille C, Vitali B, Margolis L. Vaginal Lactobacillus inhibits HIV-1 replication in human tissues ex vivo. Front Microbiology 2017 May; 906. doi: 10.3389/fmicb.2017.00906 PMID: 28579980
37. Nunn KL, Wang YY, Harit D, Humphrys MS, Ma B, Cone R, Ravel J, Lai SK. Enhanced Trapping of HIV-1 by Human Cervicovaginal Mucus Is Associated with Lactobacillus crispatus-Dominant Microbiota. MBio. 2015 Oct 6;6(5):e01084-15. doi: 10.1128/mBio.01084-15. PMID: 26443453
38. Lai SK, Hida K, Shukair S, Wang YY, Figueiredo A, Cone R. Human immunodeficiency virus type 1 is trapped by acidic but not by neutralized human cervicovaginal mucus. Journal of Virology Nov; 83(21): 11196-200. doi: 10.1128/JVI.01899-08 PMID:19692470
39. Tyssen D, Wang YY, Hayward JA, Agius PA, DeLong K, Aldunate M et al. Anti-HIV-1 activity of lactic acid in human cervicovaginal fluid. mSphere 2018 Jul; 3(4). doi: 10.1128/mSphere.00055-18. PMID: 29976641
40. Allsworth JE and Peipert JF. Prevalence of bacterial vaginosis: 2001-2004 National Health and Nutrition Examination Survey data. Obstet Gynecol 2007;109(1):114-20.
41. Eschenbach DA, Hillier S, Critchlow C, et al. Diagnosis and clinical manifestations of bacterial vaginosis. Am J Obstet Gynecol 1988;158(4):819-28.
42. Hillier SL, Kiviat NB, Hawes SE, et al. Role of bacterial vaginosis- associated microorganisms in endometritis. Am J Obstet Gynecol 1996;175(2):435-41.
43. Watts DH, Krohn MA, Hillier SL, et al. Bacterial vaginosis as a risk factor for post- cesarean endometritis. Obstet Gynecol 1990;75(1):52-8.
44. Soper DE. Bacterial vaginosis and postoperative infections. Am J Obstet Gynecol. 1993 Aug;169(2 Pt 2):467-9.
45. Kurki T, Sivonen A, Renkonen OV, et al. Bacterial vaginosis in early pregnancy and pregnancy outcome. Obstet Gynecol 1992;80(2):173-7.
46. Hillier SL, Martius J, Krohn M, et al. A case-control study of chorioamnionic infection and

histologic chorioamnionitis in prematurity. N Engl J Med 1988;319(15):972-8.
47. Taha TE, Hoover DR, Dallabetta GA, et al. Bacterial vaginosis and disturbances of vaginal flora: association with increased acquisition of HIV. AIDS 1998;12(13):1699-706.
48. Myer L, Denny L, Telerant R, et al. Bacterial vaginosis and susceptibility to HIV infection in South African women: a nested case-control study. J Infect Dis 2005;192(8):1372-80.
49. Cohen CR, Lingappa JR, Baeten JM, et al. Bacterial vaginosis associated with increased risk of female-to-male HIV-1 transmission: a prospective cohort analysis among African couples. PLoS Med. 2012;9(6):e1001251. doi: 10.1371/journal.pmed.1001251. Epub 2012 Jun 26.
50. Fichorova RN, Onderdonk AB, Yamamoto H, et al. Maternal microbe-specific modulation of inflammatory response in extremely low-gestational-age newborns. MBio 2011;2(1).
51. Bradshaw CS, Morton AN, Hocking J, et al. High recurrence rates of bacterial vaginosis over the course of 12 months after oral metronidazole therapy and factors associated with recurrence. J Infect Dis 2006;193(11):1478-86.
52. Bukusi EA, Thomas KK, Nguti R, Cohen CR, et al. Topical Penile Microbicide Use by Men to Prevent Recurrent Bacterial Vaginosis in Sex Partners: A Randomized Clinical Trial. Sex Transm Dis. 2011 Jun;38(6):483-9.
53. Bolton M van der Straten A and Cohen CR. Probiotics: potential to prevent HIV and sexually transmitted infections in women. Sex Transm Dis 2008;35(3):214- 25.
54. Balkus JE, Richardson BA, Mandaliya K, Kiarie J, Jaoko W, Ndinya-Achola JO, et al. Establishing and sustaining a healthy vaginal environment: analysis of data from a randomized trial of periodic presumptive treatment for vaginal infections. The Journal of infectious diseases 2011 Jul 15;204(2):323-6. doi: 10.1093/infdis/jir241. PMID: 21673045
55. Abdool Karim Q, Abdool Karim SS, Frohlich JA, et al. Effectiveness and safety of tenofovir gel, an antiretroviral microbicide, for the prevention of HIV infection in women. Science. 2010;329(5996):1168-1174.
56. Anahtar MN, Byrne EH, Koherty KE, et al. Cervicovaginal bacteria are a major modulator of host inflammatory responses in the female genital tract. Immunity 2015;42(5):965-76.
57. Cohen CR, Moscicki AB, Scott ME, et al. Increased levels of immune activation in the genital tract of healthy young women from sub-Saharan Africa. AIDS 2010;24(13):2069- 74.
58. Stapleton AE, Au-Yeung M, Hooton TM, et al. Randomized, Placebo-Controlled Phase 2 Trial of a Lactobacillus crispatus Probiotic Given Intravaginally for Prevention of Recurrent Urinary Tract Infection. Clin Infect Dis 2011;52(10):1212-7.
59. DIVISION OF AIDS Table For Grading The Severity Of Adult And Pediatric Adverse Events. December 2004. Addendum 1. Female Genital Grading Table For Use In Microbicide Studies. https://mtnstopshiv.org/sites/default/files/attachments/Addendum_1_Female_Genital_Grading_Table_v1_Nov_2007_0.pdf. Accessed: 09-06-2018
60. WHO/ CONRAD. Manual For The Standardization Of Colposcopy For The Evaluation Of Vaginal Products. Update 2004. http://www.conrad.org/assets/attachments/Revised_Manual.PDF. Accessed 09-06-2018.
61. Hemmerling A, Harrison W, Schroeder A, Park J, Korn A, Shiboski S, Cohen CR. Phase 1 dose-ranging safety trial of Lactobacillus crispatus CTV-05 for the prevention of bacterial vaginosis. Sex Transm Dis. 2009 Sep;36(9):564-9. doi: 10.1097/OLQ.0b013e3181a74924. PMID:19543144

62. Hemmerling A, Harrison W, Schroeder A, Park J, Korn A, Shiboski S, et al. Phase 2a study assessing colonization efficiency, safety, and acceptability of Lactobacillus crispatus CTV- 05 in women with bacterial vaginosis. Sexually transmitted diseases 2010 Dec;37(12):745- 50. doi: 10.1097/OLQ.0b013e3181e50026. PMID: 20644497
63. Ngugi BM, Hemmerling A, Bukusi EA, Kikuvi G, Gikunju J, Shiboski S, Fredricks DN, Cohen CR. Effects of bacterial vaginosis-associated bacteria and sexual intercourse on vaginal colonization with the probiotic Lactobacillus crispatus CTV-05. Sex Transm Dis. 2011 Nov;38(11):1020-7. doi: 10.1097/OLQ.0b013e3182267ac4. PMID: 21992977
64. Bradshaw CS, Morton AN, Hocking J, Garland SM, Morris MB, Moss LM, et al. High recurrence rates of bacterial vaginosis over the course of 12 months after oral metronidazole therapy and factors associated with recurrence. The Journal of infectious diseases 2006 Jun 1;193(11):1478-86. PMID: 16652274
65. Mitchell C, Manhart LE, Thomas K, Fiedler T, Fredricks DN, Marrazzo J. Behavioral predictors of colonization with Lactobacillus crispatus or Lactobacillus jensenii after treatment for bacterial vaginosis: a cohort study. Infectious diseases in obstetrics and gynecology 2012;2012:706540. doi: 10.1155/2012/706540. PMID: 22693410
66. McClelland RS, Richardson BA, Hassan WM, Chohan V, Lavreys L, Mandaliya K, et al. Improvement of vaginal health for Kenyan women at risk for acquisition of human immunodeficiency virus type 1: results of a randomized trial. The Journal of infectious diseases 2008 May 15;197(10):1361-8. doi: 10.1086/587490. PMID: 18444793
67. Balkus JE, Manhart LE, Lee J, Anzala O, Kimani J, Schwebke J, et al. Periodic Presumptive Treatment for Vaginal Infections May Reduce the Incidence of Sexually Transmitted Bacterial Infections. The Journal of infectious diseases 2016 Jun 15;213(12):1932-7. doi: 10.1093/infdis/jiw622. PMID: 28007924
68. Balkus JE, Jaoko W, Mandaliya K, Richardson BA, Masese L, Gitau R, et al. The posttrial effect of oral periodic presumptive treatment for vaginal infections on the incidence of bacterial vaginosis and Lactobacillus colonization. Sexually transmitted diseases 2012 May;39(5):361-5. doi: 10.1097/OLQ.0b013e31824790d7. PMID: 22504600
69. Division of AIDS Table for Grading the Severity of Adult and Pediatric Adverse Events, December 2004; Addendum 1: Female Genital Grading Table for Use in Microbicide Studies, November 2007
70. FDA Draft Guidance for the Use of Metronidazole Gel. http://www.fda.gov/downloads/drugs/guidancecomplianceregulatoryinformation/guidances /u cm345807.pdf
71. Swedek I, Hemmerling A, Cohen CR, Lee PP, Lagenaur LA, Parks TP, Marcobal A. Safe sex for vaginal microbiota? Lactobacillus inhibition by lubricants and lubricated condoms. Poster at International Human Microbiome Conference (IHMC). 2016
72. CDC Sexually Transmitted Treatment Guidelines. http://www.cdc.gov/std/tg2015/tg- 2015-print.pdf
73. International Conference on Harmonisation (ICH) E9. http://www.ich.org/fileadmin/Public_Web_Site/ICH_Products/Guidelines/Efficacy/E9/Step 4/ E9_Guideline.pdf
74. Happel AU, Jaumdally SZ, Pidwell T, Cornelius T, Jaspan HB, Froissart R et al. Probiotics for vaginal health in South Africa: what is on the retarilers' shelves? BMC Womens Health 2017 Jan; 17(1):7 doi: 10.1186/s12905-017-0362-6. PMID: 28103868

19.0 APPENDICES

Appendix A	Schedule of Events
Appendix B	Division of AIDS Table for Grading the Severity of Adult and Pediatric Adverse Events, December 2004; Addendum 1: Female Genital Grading Table for Use in Microbicide Studies, November 2007
Appendix C	DAIDS Toxicity Table for Grading the Severity of Adult and Pediatric Adverse Events, Version 2.1 – July 2017
Appendix D	Coordination between FRESH and LACTIN-V studies

Appendix A Schedule of Events

Evaluation		Enrolment Visit 1 Day 1	Study Treatment		Study Follow-Up					
			Randomization Visit 3 (Day 8)	Check-In and Dosing Visits 4 - 10	Follow-Up Visit 11 Day 36	Check-In Visits 12 - 18	Final Study Visit 19 Day 64	Unscheduled Visit	Study Product Discontinuation	Early Termination Visit
Visit window (Study Days)					33- 43		57-71			
Signed consent form		X								
Assessment of eligibility criteria		X	X							
Demographics		X								
Randomization			X							
Detailed medical, gynaecological and sexual history		X								
Dispense Metronidazole		X			(X)		(X)			
Dispense LACTIN-V/placebo and applicators			X	X						
Dispense condoms		X	X	X	X	X		X	X	
Brief medical, gynaecological and sexual history			X		X		X	X	X	X
Review of concomitant medications		X	X		X		X	X	X	X
Study intervention			X	X						
Physical Examination	Complete	X								
	Symptom-directed		X		X		X	X	X	X
	Vital signs	X	X		X		X	X	X	X
	Pelvic examination		X		X		X	X	X	X
Review symptoms, AEs, menses, sexual activity				X	X	X	X	X	X	X
Counsel to abstain from sexual intercourse			X	X	X	X		X		

			Study Treatment		Study Follow-Up					
Evaluation		Enrolment Visit 1 Day 1	Randomization Visit 3 (Day 8)	Check-In and Dosing Visits 4 - 10	Follow-Up Visit 11 Day 36	Check-In Visits 12 - 18	Final Study Visit 19 Day 64	Unscheduled Visit	Study Product Discontinuation	Early Termination Visit
Reminder not to use vaginal products			X	X	X	X		X		
Assessment of adverse events				X	X	X	X	X	X	X
Clinical Labor	Clean catch urine dipstick	X	X		X		X	X	X	X
	Urinalysis	(X)	(X)		(X)		(X)	(X)	(X)	(X)
	Rapid urine βhCG pregnancy test	X	X		X		X	X	X	X
	Vaginal swab for *N. gonorrhoeae, C. trachomatis, T. vaginalis and M. genitalium*		(X)		(X)		(X)	(X)	(X)	(X)
Research Laboratory	Vaginal swab for pH		X		X		X	X	X	X
	PSA test for sperm exposure as POCT		X		X		X	X	X	X
	Vaginal swab for Gram stain		X		X		X	X	X	X
	Vaginal swab for cytokines, HIV Target cells, vaginal bacteria, lactobacilli species		X		X		X	X	X	X
	Vaginal swab for qPCR (*L. crispatus* identification)		X		X		X	X	X	X
	Cervicovaginal Lavage		X		X		X	X	X	X
	Future Use vaginal swabs		X		X		X	X	X	X
	Acceptability questionnaire	X			X				X	X

(X) if indicated

DIVISION OF AIDS TABLE FOR GRADING THE SEVERITY OF ADULT AND PEDIATRIC ADVERSE EVENTS
PUBLISH DATE: DECEMBER 2004
Addendum 1
Female Genital Grading Table for Use in Microbicide Studies

APPENDIX B

INDIVIDUAL SIGNS/SYMPTOMS					
**PARAMETER**	**GRADE 0 NORMAL**	**GRADE 1 MILD**	**GRADE 2 MODERATE**	**GRADE 3 SEVERE**	**GRADE 4 POTENTIALLY LIFE-THREATENING**
**GENERAL**					
Odor	No complaint	Mild-moderate unpleasant odor	Severe unpleasant odor	NA	NA
**PAIN AND TENDERNESS (Specify Area: Vulvar/Perineum, Vagina, Cervix (including cervical motion tenderness), Uterus, Adnexae, Pelvic/Lower Abdominal, or Ovulatory)** ***Note – if both pain and tenderness are present, only report the one with the most severe grade**					
Pain* [1]	None	Pain causing no or minimal interference with usual social & functional activities	Pain causing greater than minimal interference with usual social & functional activities or the need for non-narcotic medication	Pain causing inability to perform usual social & functional activities or the need for narcotic medication	Disabling pain causing inability to perform basic self-care functions OR hospitalization (other than emergency room visit) indicated
Tenderness* [1]	None	Mild tenderness	Moderate tenderness	Severe tenderness	NA
Dyspareunia (pain with sexual activity)	None	Pain causing no or minimal interference with sexual function	Pain causing greater than minimal interference with sexual function	NA	NA
Dysmenorrhea/cramping with menses	None	Pain causing no or minimal interference with usual social & functional activities	Pain causing greater than minimal interference with usual social & functional activities or the need for non-narcotic medication	Pain causing inability to perform usual social or functional activities or the need for narcotic medication	NA

[1] If pain or tenderness is included in the grading of another category (e.g., PID), it should not be graded again in the pain or tenderness category.

NOTE: For protocols utilizing this Addendum, when the same parameter appears in both this Female Genital Grading Table and the main DAIDS AE Grading Table, use the grading scheme in this table.

DIVISION OF AIDS TABLE FOR GRADING THE SEVERITY OF ADULT AND PEDIATRIC ADVERSE EVENTS
PUBLISH DATE: DECEMBER 2004
Addendum 1
Female Genital Grading Table for Use in Microbicide Studies

INDIVIDUAL SIGNS/SYMPTOMS					
**PARAMETER**	**GRADE 0 NORMAL**	**GRADE 1 MILD**	**GRADE 2 MODERATE**	**GRADE 3 SEVERE**	**GRADE 4 POTENTIALLY LIFE-THREATENING**
**GENITOURINARY SIGNS/SYMPTOMS – VULVA**					
Vulvar/vaginal itching	None	Itching causing no, mild, or moderate interference with usual social & functional activities	Itching causing inability to perform usual social & functional activities; may require intervention such as antihistamine or bathing to provide relief	NA	NA
Vulvar edema	None	Mild, non-pitting edema	Moderate, 1-2+ pitting edema	3+ pitting edema, severe enough to require urinary drainage, or weeping edema ± skin breakdown	NA
Vulvar erythema	None	Erythema covering < 50% of vulvar surface	Erythema covering ≥ 50% of vulvar surface	NA	NA
Vulvar lesions (findings seen only by colposcopy should not be included here)	Normal variants including skin tags, moles, scars, etc.	Blisters, ulcerations, or pustules - no treatment indicated	Blisters, ulcerations or pustules, with treatment indicated	Severe epithelial disruption with hospitalization indicated	NA
Vulvar rash	None	Rash covering < 50% of vulvar surface	Rash covering ≥ 50% of vulvar surface	Severe epithelial disruption with hospitalization indicated	NA
Bartholin's or Skene's gland	No findings	Cyst with no inflammation	Cyst or abscess with outpatient intervention indicated	Cyst or abscess with hospitalization indicated	Necrotizing fasciitis from Bartholin's abscess
**GENITOURINARY SIGNS/SYMPTOMS – VAGINA** **** Note – if vaginal discharge is present both by history and on examination, only report the one with the most severe grade**					
Vaginal edema	None	Mild-moderate engorgement	Loss of ruggae and friability	NA	NA
Vaginal erythema	None	Erythema covering < 50% of vaginal surface	Erythema covering ≥ 50% of vaginal surface	NA	NA

NOTE: For protocols utilizing this Addendum, when the same parameter appears in both this Female Genital Grading Table and the main DAIDS AE Grading Table, use the grading scheme in this table.

DIVISION OF AIDS TABLE FOR GRADING THE SEVERITY OF ADULT AND PEDIATRIC ADVERSE EVENTS
PUBLISH DATE: DECEMBER 2004
Addendum 1
Female Genital Grading Table for Use in Microbicide Studies

INDIVIDUAL SIGNS/SYMPTOMS					
**PARAMETER**	**GRADE 0 NORMAL**	**GRADE 1 MILD**	**GRADE 2 MODERATE**	**GRADE 3 SEVERE**	**GRADE 4 POTENTIALLY LIFE-THREATENING**
Vaginal dryness	No complaint	Dryness causing no or minimal interference with usual sexual, social, & functional activities	Dryness causing greater than minimal interference with usual sexual, social, & functional activities	NA	NA
Vaginal discharge by participant report **	Participant's usual amount of discharge, regardless of color or quantity	Mild-moderate increase in amount above participant baseline - no sanitary protection required	Profuse increase in discharge requiring pad use or other hygienic intervention	NA	NA
Vaginal discharge as observed by clinician ** (red or brown discharge should be reported under bleeding, not discharge)	Slight amount of discharge, any color	Mild-moderate increase in amount	Significant increase in amount with pooling in vagina on examination	NA	NA
Vaginal abrasions or lacerations (including probable applicator injuries)	None	Superficial disruptions and disruptions extending through the mucosa with minimal impact on life	Large disruptions extending through the mucosa or large superficial disruptions, hospitalization not indicated	Large disruptions extending through the mucosa or large superficial disruptions, hospitalization indicated	Lacerations extending into the peritoneal cavity, bladder, or rectum
Vaginal lesions (findings seen only by colposcopy should not be included here)	Normal variants including skin tags, moles, scars, etc.	Blisters, ulcerations, or pustules, no treatment indicated	Blisters, ulcerations, or pustules with treatment indicated	Severe epithelial disruption requiring hospitalization	NA
Vaginal and Cervical masses (polyps, myomas, or possible malignancy)	None or normal variants such as Nabothian cyst or Gartner duct cyst	Polyp or myoma or undiagnosed mass without symptoms	Polyp, myoma, or undiagnosed mass causing mild symptoms, e.g., bleeding/pain not requiring more than mild analgesia	Polyp, myoma, or undiagnosed mass causing severe symptoms, e.g., bleeding/pain affecting bladder and bowel function	Visible cervical cancer
**GENITOURINARY SIGNS/SYMPTOMS – CERVIX**					
Cervical edema and friability	None	Edema without friability	Friable cervix	NA	NA

NOTE: For protocols utilizing this Addendum, when the same parameter appears in both this Female Genital Grading Table and the main DAIDS AE Grading Table, use the grading scheme in this table.

DIVISION OF AIDS TABLE FOR GRADING THE SEVERITY OF ADULT AND PEDIATRIC ADVERSE EVENTS
PUBLISH DATE: DECEMBER 2004
Addendum 1
Female Genital Grading Table for Use in Microbicide Studies

INDIVIDUAL SIGNS/SYMPTOMS					
**PARAMETER**	**GRADE 0 NORMAL**	**GRADE 1 MILD**	**GRADE 2 MODERATE**	**GRADE 3 SEVERE**	**GRADE 4 POTENTIALLY LIFE-THREATENING**
Cervical erythema	None	Erythema covering < 50% of cervix	Erythema covering ≥ 50% of cervix	NA	NA
Cervical discharge	White or clear discharge	Small amount of purulent discharge at os	Purulent discharge extending onto cervix or vagina	NA	NA
Visible cervical lesions (findings seen only by colposcopy should not be included here)	Normal variants including skin tags, moles, scars, etc.	Blisters, ulcerations, or pustules, no treatment indicated	Blisters, ulcerations, or pustules with treatment indicated	NA	NA
**GENITOURINARY SIGNS/SYMPTOMS – UTERUS**					
Uterine masses/enlargement based on bimanual examination	Normal to 8 week size, no palpable myomas	Enlarged uterus and mild symptoms, e.g., bleeding/pain requiring mild analgesics	Enlarged uterus/myoma with moderate pain or symptoms, e.g., bleeding	Mass causing severe bleeding/pain or with impact on bowel/bladder function	Uterine mass that requires transfusion or surgery
Polyp, submucosal fibroid, or thickened endometrium detected by transvaginal ultrasound (new or increasing in size from prior exam)	None or unchanged/reduced in size from prior exam	New myomas < 6 cm diameter (single or multiple) or diameter increased < 6 cm since prior exam	New myomas ≥ 6 cm diameter (single or multiple) or diameter increased ≥ 6 cm since prior exam	Hospitalization and/or surgery indicated	NA
**GENITOURINARY SIGNS/SYMPTOMS – ADNEXA**					
Not pregnancy- or infection-related adnexal masses based on bimanual exam (use if no ultrasound done; if ultrasound done, use ultrasound categories below)	None, ≤ 4 cm, normal size ovary	> 4 cm with minimal or no symptoms	> 4 cm with severe symptoms, e.g., pain, but hospitalization not indicated (see footnote #1)	> 4 cm with severe symptoms, e.g., pain and hospitalization indicated (see footnote #1)	NA
Hydrosalpinx based on ultrasound	None	Asymptomatic, suspected hydrosalpinx	Hydrosalpinx with pain, but without evidence of infection or ectopic pregnancy	Signs/symptoms of infection with hospitalization and/or surgery indicated	NA
Adnexal mass based on ultrasound	None	Simple cyst, asymptomatic	Simple cyst, symptomatic	Mass suspicious for malignancy	Malignant mass

NOTE: For protocols utilizing this Addendum, when the same parameter appears in both this Female Genital Grading Table and the main DAIDS AE Grading Table, use the grading scheme in this table.

DIVISION OF AIDS TABLE FOR GRADING THE SEVERITY OF ADULT AND PEDIATRIC ADVERSE EVENTS
PUBLISH DATE: DECEMBER 2004
Addendum 1
Female Genital Grading Table for Use in Microbicide Studies

INDIVIDUAL SIGNS/SYMPTOMS					
**PARAMETER**	**GRADE 0 NORMAL**	**GRADE 1 MILD**	**GRADE 2 MODERATE**	**GRADE 3 SEVERE**	**GRADE 4 POTENTIALLY LIFE-THREATENING**
**GENITOURINARY SIGNS/SYMPTOMS – ABDOMEN**					
Abdominal mass not palpable on pelvic exam of unknown diagnosis	None or known (pre-existing) mass unchanged in size	New mass or increased size of known mass requiring mild analgesia with minimal impact	New mass or increased size of known mass with moderate symptoms	Mass causing severe bleeding/ pain with impact on bladder/bowel function or with hospitalization indicated	Malignancy
**GENITOURINARY SIGNS/SYMPTOMS – URINARY TRACT**					
Urinary frequency	None	Up to 2 times participant's normal frequency	> 2 times participant's normal frequency	NA	NA
Dysuria	None	Superficial only	Deep ± superficial	Inability to void due to pain	NA
Hematuria	None	Microscopic, no intervention indicated (beyond evaluation for infection)	Gross blood in urine or medical intervention/ evaluation indicated (beyond evaluation for infection)	Persistent bleeding with transfusion, hospitalization or intervention indicated to obtain hemostasis (endoscopy, interventional radiology, or operative)	Profuse hemorrhage with shock or orthostatic dizziness

NOTE: For protocols utilizing this Addendum, when the same parameter appears in both this Female Genital Grading Table and the main DAIDS AE Grading Table, use the grading scheme in this table.

DIVISION OF AIDS TABLE FOR GRADING THE SEVERITY OF ADULT AND PEDIATRIC ADVERSE EVENTS
PUBLISH DATE: DECEMBER 2004
Addendum 1
Female Genital Grading Table for Use in Microbicide Studies

COMPOSITE SIGNS/SYMPTOMS (Use instead of individual categories if 2 or more signs/symptoms are present)					
**PARAMETER**	**GRADE 0 NORMAL**	**GRADE 1 MILD** (Use if all signs/ symptoms would individually be Grade 0 or 1)	**GRADE 2 MODERATE** (Use if one or more signs/symptoms would individually be Grade 2 and all others Grade 0 or 1)	**GRADE 3 SEVERE** (Use if one or more signs/symptoms would individually be Grade 3)	**GRADE 4 POTENTIALLY LIFE-THREATENING**
**NO ORGANISM IDENTIFIED BUT INADEQUATE TESTING PERFORMED**					
Vulvovaginitis (combinations of pain, itching, erythema, edema, rash, tenderness, or discharge)	None	Mild signs/ symptoms	Moderate signs/ symptoms	Severe signs/ symptoms	NA
Cervicitis (combinations of dyspareunia, erythema, edema, tenderness, and discharge)	None	Mild signs/ symptoms	Moderate signs/ symptoms	Severe signs/ symptoms	NA
PID (if Gonorrhea or Chlamydia identified use that category)	None	NA	Cervicitis with mild uterine tenderness, ± mild cervical motion tenderness, no signs of peritoneal irritation	More diffuse tenderness, any signs of peritoneal irritation, or indications for hospitalization	Tubo-ovarian abscess or surgery required for resolution
**NO ORGANISM IDENTIFIED AFTER APPROPRIATE TESTING PERFORMED**					
Vulvovaginitis (combinations of pain, itching, erythema, edema, rash, tenderness, or discharge)	None	Mild signs/ symptoms	Moderate signs/ symptoms	Severe signs/ symptoms	NA
Cervicitis (combinations of dyspareunia, erythema, edema, tenderness, and discharge)	None	Mild signs/ symptoms	Moderate signs/ symptoms	Severe signs/ symptoms	NA
PID (if Gonorrhea or Chlamydia identified use that category)	None	NA	Cervicitis with mild uterine tenderness, ± mild cervical motion tenderness, no signs of peritoneal irritation	More diffuse tenderness, any signs of peritoneal irritation, or indications for hospitalization	Tubo-ovarian abscess or surgery required for resolution

NOTE: For protocols utilizing this Addendum, when the same parameter appears in both this Female Genital Grading Table and the main DAIDS AE Grading Table, use the grading scheme in this table.

DIVISION OF AIDS TABLE FOR GRADING THE SEVERITY OF ADULT AND PEDIATRIC ADVERSE EVENTS
PUBLISH DATE: DECEMBER 2004
Addendum 1
Female Genital Grading Table for Use in Microbicide Studies

INFECTIONS AND DYSPLASIA					
**PARAMETER**	**GRADE 0 NORMAL**	**GRADE 1 MILD**	**GRADE 2 MODERATE**	**GRADE 3 SEVERE**	**GRADE 4 POTENTIALLY LIFE-THREATENING**
**GENITOURINARY INFECTIONS**					
Genital herpes	No lesions	Characteristic ulcerative or vesicular lesions confirmed by culture, PCR, Tzanck prep or other diagnostic test of lesion or previous type-specific serology, covering < 25% of vulva, vagina, or cervix	Same criteria as mild but covering 25-50% of vulvar, vaginal, or cervical surface	Same criteria as mild but covering > 50% of vulvar, vaginal, or cervical surface	Symptoms of significant systemic involvement, e.g., encephalitis, hepatitis
Candida	Absence of symptoms regardless of candida test results	Positive culture, wet mount, or other laboratory test for yeast, with mild symptoms	Positive culture, wet mount, or other laboratory test for yeast, with moderate to severe symptoms	NA	NA
Trichomonas	Negative	NA	Positive wet mount, culture, PCR or other licensed test, excluding pap smear, showing T. vaginalis, regardless of symptoms	NA	NA
Bacterial Vaginosis (BV)	Negative	Asymptomatic BV diagnosed by Amsel criteria, wet mount, Gram stain, or licensed diagnostic test	Symptomatic confirmed by wet mount, Gram stain, or any licensed diagnostic test	NA	NA

NOTE: For protocols utilizing this Addendum, when the same parameter appears in both this Female Genital Grading Table and the main DAIDS AE Grading Table, use the grading scheme in this table.

DIVISION OF AIDS TABLE FOR GRADING THE SEVERITY OF ADULT AND PEDIATRIC ADVERSE EVENTS
PUBLISH DATE: DECEMBER 2004
Addendum 1
Female Genital Grading Table for Use in Microbicide Studies

INFECTIONS AND DYSPLASIA					
**PARAMETER**	**GRADE 0 NORMAL**	**GRADE 1 MILD**	**GRADE 2 MODERATE**	**GRADE 3 SEVERE**	**GRADE 4 POTENTIALLY LIFE-THREATENING**
Chlamydia	Negative	NA	Positive culture or other diagnostic test for Chlamydia, asymptomatic or with mild uterine or cervical motion tenderness (no signs of peritoneal irritation)	Positive test for Chlamydia with abdominal or uterine or adnexal tenderness on examination, with or without adnexal mass, diffuse tenderness, any signs of peritoneal irritation, or indications for hospitalization	Tubo-ovarian abscess or surgery required for resolution
Gonorrhea	Negative	NA	Positive culture or other diagnostic test for Gonorrhea, asymptomatic or with mild uterine or cervical motion tenderness (no signs of peritoneal irritation)	Positive test for Gonorrhea with abdominal or uterine or adnexal tenderness on examination, with or without adnexal mass, diffuse tenderness, any signs of peritoneal irritation, or indications for hospitalization	Tubo-ovarian abscess or surgery required for resolution or disseminated gonococcal infection
Urinary tract infection (by urinalysis and urine culture)	Negative	5-10 WBC/hpf on urinalysis with a negative culture per protocol definition (with or without symptoms)	> 10 WBC/hpf on urinalysis OR a positive culture per protocol definition (with or without symptoms)	Pyelonephritis	Sepsis (septicemia) due to urinary tract infection

NOTE: For protocols utilizing this Addendum, when the same parameter appears in both this Female Genital Grading Table and the main DAIDS AE Grading Table, use the grading scheme in this table.

DIVISION OF AIDS TABLE FOR GRADING THE SEVERITY OF ADULT AND PEDIATRIC ADVERSE EVENTS
PUBLISH DATE: DECEMBER 2004
Addendum 1
Female Genital Grading Table for Use in Microbicide Studies

INFECTIONS AND DYSPLASIA					
**PARAMETER**	**GRADE 0 NORMAL**	**GRADE 1 MILD**	**GRADE 2 MODERATE**	**GRADE 3 SEVERE**	**GRADE 4 POTENTIALLY LIFE-THREATENING**
Syphilis	Negative treponemal or non-treponemal test or both positive with known treatment and stable titers (< 4 fold increase)	NA	Syphilis diagnosed by a positive treponemal test along with a positive non- treponemal test and no previous treatment or a four-fold rise in titer on the non- treponemal test after previous treatment regardless of symptoms or non-oral lesions positive by darkfield exam for treponemes	Criteria for Grade 2 Syphilis in the presence of neurologic symptoms or a positive CSF VDRL or FTA-ABS	NA
**GENITAL DYSPLASIA**					
Condyloma (specify site: cervical, vaginal, vulvar, perianal)	None	Condylomata causing no or mild interference with daily function	Condylomata causing moderate interference with daily function	Condylomata causing severe interference with daily function, secondary infection, or hospitalization indicated	NA
Intraepithelial Neoplasia by biopsy (VIN, CIN, VAIN)	None	Intraepithelial Neoplasia 1 (IN1)	Intraepithelial Neoplasia 2 (IN2)	Carcinoma in situ (CIS)	Invasive carcinoma
Pap (use this category only if treatment performed without diagnostic testing, otherwise use biopsy category above)	nl PAP	ASCUS or LSIL	HSIL	Carcinoma in situ or Carcinoma	NA

NOTE: For protocols utilizing this Addendum, when the same parameter appears in both this Female Genital Grading Table and the main DAIDS AE Grading Table, use the grading scheme in this table.

DIVISION OF AIDS TABLE FOR GRADING THE SEVERITY OF ADULT AND PEDIATRIC ADVERSE EVENTS
PUBLISH DATE: DECEMBER 2004
Addendum 1
Female Genital Grading Table for Use in Microbicide Studies

UTERINE BLEEDING AND PREGNANCY COMPLICATIONS					
**PARAMETER**	**GRADE 0 NORMAL**	**GRADE 1 MILD**	**GRADE 2 MODERATE**	**GRADE 3 SEVERE**	**GRADE 4 POTENTIALLY LIFE-THREATENING**
**ABNORMAL UTERINE BLEEDING UNRELATED TO PREGNANCY**					
Menorrhagia [2] (prolonged and/or heavy menstrual bleeding)	Participant report of normal bleeding relative to her baseline	Increase from usual with no or minimal interference with usual social & functional activities (including sexual functioning)	Increase from usual with moderate interference with usual social & functional activities (including sexual)	Incapacitating or severe interference with usual social & functional activities (including sexual functioning), transfusion indicated	Life threatening hemorrhage with or without shock
Metrorrhagia [2] (intermenstrual or frequent bleeding)	None or any expected nonmenstrual bleeding	Increase from usual with no or minimal interference with usual social & functional activities (including sexual functioning)	Increase from usual with moderate interference with usual social & functional activities (including sexual)	Incapacitating or severe interference with usual social & functional activities (including sexual functioning), transfusion indicated	Life threatening hemorrhage with or without shock
Unexplained infrequent bleeding (excludes expected absence of menses due to hormonal contraception or pregnancy/postpartum)	Participant report of normal or expected bleeding frequency	No menses for 1-3 months (missed menses)	No menses for > 3 months (oligomenorrhea/ amenorrhea)	NA	NA
Postcoital bleeding	None	Occasional (< 25% of coital acts) OR Increase from usual with no or minimal interference with usual social functioning (including sexual functioning)	Frequent (25-75% of coital acts) OR Increase from usual with moderate interference with usual social functioning (including sexual)	Consistent (> 75% of coital acts) OR Incapacitating or severe interference with usual social functioning (including sexual functioning), transfusion indicated	Life threatening hemorrhage with or without shock

[2] If both Menorrhagia and Metrorrhagia are present, a single adverse event should be reported as "Menometrorrhagia" and graded per the Menorrhagia grading scale.

NOTE: For protocols utilizing this Addendum, when the same parameter appears in both this Female Grading Table and the main DAIDS AE Grading Table, use the grading scheme in this table.

APPENDIX B (Cont'd)
DIVISION OF AIDS TABLE FOR GRADING THE SEVERITY OF ADULT AND PEDIATRIC ADVERSE EVENTS PUBLISH DATE: DECEMBER 2004
Addendum 1
Female Genital Grading Table for Use in Microbicide Studies

UTERINE BLEEDING AND PREGNANCY COMPLICATIONS					
**PARAMETER**	**GRADE 0 NORMAL**	**GRADE 1 MILD**	**GRADE 2 MODERATE**	**GRADE 3 SEVERE**	**GRADE 4 POTENTIALLY LIFE-THREATENING**
**COMPLICATIONS OF PREGNANCY**					
First trimester bleeding	None	Spotting or bleeding less than menses with continuation of pregnancy	Bleeding like menses or heavier with continuation of pregnancy	Spontaneous abortion, or profuse bleeding with dizziness or orthostatic hypotension, transfusion indicated	Spontaneous abortion with profuse bleeding and/or shock
Postabortal endometritis/salpingitis	None	Low grade fever and uterine tenderness, resolved with oral antibiotics	Moderate symptoms, requiring ≤ 3 days of parenteral antibiotics	Severe symptoms requiring > 3 days of IV antibiotics or development of tubo-ovarian abscess	Ruptured TOA or diffuse peritonitis or severe uterine infection for which operative intervention indicated
Postpartum hemorrhage	EBL < 500 cc for vaginal delivery or < 1000 cc after CS or reported as normal	EBL 500-1000 for vaginal delivery or 1000-1500 for CS or reported as slightly increased	EBL > 1000 for vaginal delivery or > 1500 for CS, with or without mild dizziness, no transfusion required	Hemorrhage at a level for which transfusion of 1-2 units of packed cells, but no other blood products indicated	Hemorrhage with shock or coagulopathy, for which transfusion of > 2 units of packed cells or any amount of other blood components is indicated
Postpartum endometritis	None	Low grade fever and uterine tenderness, resolved with oral antibiotics	Moderate symptoms, treated by ≤ 3 days of parenteral antibiotics	Severe symptoms treated with > 3 days of IV antibiotics or addition of heparin	Severe infection or infection for which operative intervention is indicated

Chorioamnionitis	None	Fever (38°C – 38.4°C or 100.4°F – 100.9°F) with two or more: FHR > 160 BPM, maternal HR > 120, uterine tenderness between contractions or purulent AF or preterm labor	Same as Grade 1 plus fever 38.5°C – 40°C or 101°F – 104°F	Criteria for Grade 2 plus fetal distress or fever > 40°C or 104°F	Criteria for Grade 3 plus either fetal demise or maternal symptoms of shock

APPENDIX B (Cont'd)
DIVISION OF AIDS TABLE FOR GRADING THE SEVERITY OF ADULT AND PEDIATRIC ADVERSE EVENTS PUBLISH DATE: DECEMBER 2004
Addendum 1
Female Genital Grading Table for Use in Microbicide Studies

UTERINE BLEEDING AND PREGNANCY COMPLICATIONS					
**PARAMETER**	**GRADE 0 NORMAL**	**GRADE 1 MILD**	**GRADE 2 MODERATE**	**GRADE 3 SEVERE**	**GRADE 4 POTENTIALLY LIFE-THREATENING**
Episiotomy infection	None	Mild erythema, edema, and tenderness of wound	Fever > 38°C or 100.4°F with erythema, edema, and tenderness of wound	Fever with wound dehiscence or debridement required	Fever with signs of wound infection and shock or necrotizing fasciitis
Second/third trimester bleeding	None	Bleeding less than menses	Bleeding like menses or greater, but not requiring intervention	Bleeding requiring delivery or other intervention, e.g., transfusion	Bleeding with fetal demise or coagulopathy
Preterm rupture of membranes	None	NA	Preterm rupture with hospitalization but not resulting in delivery at less than 37 weeks' gestation	Delivery at 33-36 weeks' gestation or 1501-2500 grams birth weight	Delivery < 33 weeks' gestation or ≤ 1500 grams birth weight
Preterm contractions	None	Preterm contractions which resolve without medical intervention	Preterm contractions with cervical change which result in medical intervention but not resulting in preterm delivery	Delivery at 33-36 weeks' gestation or 1501-2500 grams birth weight	Delivery < 33 weeks' gestation or ≤ 1500 grams birth weight
Poor fetal growth	At or above 10th percentile	Fetal growth < 10th percentile but ≥ 3rd percentile for gestational age by ultrasound or newborn exam	NA	Fetal growth < 3rd percentile for gestational age by ultrasound or newborn exam	NA

APPENDIX B (Cont'd)
DIVISION OF AIDS TABLE FOR GRADING THE SEVERITY OF ADULT AND PEDIATRIC ADVERSE EVENTS PUBLISH DATE: DECEMBER 2004
Addendum 1
Female Genital Grading Table for Use in Microbicide Studies

APPENDIX C
DIVISION OF AIDS TABLE FOR
GRADING THE SEVERITY OF ADULT
AND PEDIATRIC ADVERSE EVENTS

VERSION 2.1, JULY 2017

The Division of AIDS (DAIDS) oversees more than 300 clinical trials domestically and internationally, which evaluate the safety and efficacy of therapeutic products, vaccines, and other preventive modalities. Adverse event (AE) data collected during these clinical trials form the basis for subsequent safety and efficacy analyses of pharmaceutical products and medical devices. Incorrect and inconsistent AE severity grading can lead to inaccurate data analyses and interpretation, which in turn can impact the safety and well-being of clinical trial participants and future patients using pharmaceutical products.

Over the years, DAIDS scientific knowledge and experience have expanded, necessitating revisions of the DAIDS grading table which serves as a guide for assessing the severity of AEs (including clinical and laboratory abnormalities) in participants enrolled in DAIDS-sponsored and -supported clinical trials. The *Division of AIDS (DAIDS) Table for Grading the Severity of Adult and Pediatric Adverse Events, Corrected Version 2.1 (July 2017)* updates and replaces version 2.1 (March 2017).

DAIDS is grateful to the DAIDS Grading Table Working Group, numerous government and non-government affiliated medical subject matter experts and reviewers who were instrumental in the revision of the DAIDS grading table.

PARAMETER	GRADE 1 MILD	GRADE 2 MODERATE	GRADE 3 SEVERE	GRADE 4 POTENTIALLY LIFE-THREATENING
**Clinical** adverse event **NOT** identified elsewhere in the grading table	Mild symptoms causing no or minimal interference with usual social & functional activities with intervention not indicated	Moderate symptoms causing greater than minimal interference with usual social & functional activities with intervention indicated	Severe symptoms causing inability to perform usual social & functional activities with intervention or hospitalization indicated	Potentially life-threatening symptoms causing inability to perform basic self-care functions with intervention indicated to prevent permanent impairment, persistent disability, or death

PARAMETER	GRADE 1 MILD	GRADE 2 MODERATE	GRADE 3 SEVERE	GRADE 4 POTENTIALLY LIFE-THREATENING
**Arrhythmia** (by ECG or physical examination) *Specify type, if applicable*	No symptoms AND No intervention indicated	No symptoms AND Non-urgent intervention indicated	Non-life-threatening symptoms AND Non-urgent intervention indicated	Life-threatening arrhythmia OR Urgent intervention indicated
**Blood Pressure Abnormalities** [1]				
Hypertension *(with the lowest reading taken after repeat testing during a visit)* *≥ 18 years of age*	140 to < 160 mmHg systolic OR 90 to < 100 mmHg diastolic	≥ 160 to < 180 mmHg systolic OR ≥ 100 to < 110 mmHg diastolic	≥ 180 mmHg systolic OR ≥ 110 mmHg diastolic	Life-threatening consequences in a participant not previously diagnosed with hypertension (e.g., malignant hypertension) OR Hospitalization indicated
< 18 years of age	> 120/80 mmHg	≥ 95th to < 99th percentile + 5 mmHg adjusted for age, height, and gender (systolic and/or diastolic)	≥ 99th percentile + 5 mmHg adjusted for age, height, and gender (systolic and/or diastolic)	Life-threatening consequences in a participant not previously diagnosed with hypertension (e.g., malignant hypertension) OR Hospitalization indicated
Hypotension	No symptoms	Symptoms corrected with oral fluid replacement	Symptoms AND IV fluids indicated	Shock requiring use of vasopressors or mechanical assistance to maintain blood pressure
**Cardiac Ischemia or Infarction** *Report only one*	NA	NA	New symptoms with ischemia (stable angina) OR New testing consistent with ischemia	Unstable angina OR Acute myocardial infarction
**Heart Failure**	No symptoms AND Laboratory or cardiac imaging abnormalities	Symptoms with mild to moderate activity or exertion	Symptoms at rest or with minimal activity or exertion (e.g., hypoxemia) OR Intervention indicated (e.g., oxygen)	Life-threatening consequences OR Urgent intervention indicated (e.g., vasoactive medications, ventricular assist device, heart transplant)

[1] Blood pressure norms for children < 18 years of age can be found in: Expert Panel on Integrated Guidelines for Cardiovascular Health and Risk Reduction in Children and Adolescents. *Pediatrics* 2011;128;S213; originally published online November 14, 2011; DOI: 10.1542/peds.2009-2107C.

Cardiovascular

PARAMETER	GRADE 1 MILD	GRADE 2 MODERATE	GRADE 3 SEVERE	GRADE 4 POTENTIALLY LIFE-THREATENING
**Hemorrhage** (with significant acute blood loss)	NA	Symptoms AND No transfusion indicated	Symptoms AND Transfusion of ≤ 2 units packed RBCs indicated	Life-threatening hypotension OR Transfusion of > 2 units packed RBCs (for children, packed RBCs > 10 cc/kg) indicated
**Prolonged PR Interval or AV Block** *Report only one* *> 16 years of age*	PR interval 0.21 to < 0.25 seconds	PR interval ≥ 0.25 seconds OR Type I 2nd degree AV block	Type II 2nd degree AV block OR Ventricular pause ≥ 3.0 seconds	Complete AV block
≤ 16 years of age	1st degree AV block (PR interval > normal for age and rate)	Type I 2nd degree AV block	Type II 2nd degree AV block OR Ventricular pause ≥ 3.0 seconds	Complete AV block
**Prolonged QTc Interval**[2]	0.45 to 0.47 seconds	> 0.47 to 0.50 seconds	> 0.50 seconds OR ≥ 0.06 seconds above baseline	Life-threatening consequences (e.g., Torsade de pointes, other associated serious ventricular dysrhythmia)
**Thrombosis or Embolism** *Report only one*	NA	Symptoms AND No intervention indicated	Symptoms AND Intervention indicated	Life-threatening embolic event (e.g., pulmonary embolism, thrombus)

[2] As per Bazett's formula.

Dermatologic

PARAMETER	GRADE 1 MILD	GRADE 2 MODERATE	GRADE 3 SEVERE	GRADE 4 POTENTIALLY LIFE-THREATENING
**Alopecia** (scalp only)	Detectable by study participant, caregiver, or physician AND Causing no or minimal interference with usual social & functional activities	Obvious on visual inspection AND Causing greater than minimal interference with usual social & functional activities	NA	NA
**Bruising**	Localized to one area	Localized to more than one area	Generalized	NA
**Cellulitis**	NA	Non-parenteral treatment indicated (e.g., oral antibiotics, antifungals, antivirals)	IV treatment indicated (e.g., IV antibiotics, antifungals, antivirals)	Life-threatening consequences (e.g., sepsis, tissue necrosis)
**Hyperpigmentation**	Slight or localized causing no or minimal interference with usual social & functional activities	Marked or generalized causing greater than minimal interference with usual social & functional activities	NA	NA
**Hypopigmentation**	Slight or localized causing no or minimal interference with usual social & functional activities	Marked or generalized causing greater than minimal interference with usual social & functional activities	NA	NA
**Petechiae**	Localized to one area	Localized to more than one area	Generalized	NA
**Pruritus** [3] (without skin lesions)	Itching causing no or minimal interference with usual social & functional activities	Itching causing greater than minimal interference with usual social & functional activities	Itching causing inability to perform usual social & functional activities	NA

Dermatologic

**Rash**<br>*Specify type, if applicable*	Localized rash	Diffuse rash OR Target lesions	Diffuse rash AND Vesicles or limited number of bullae or superficial ulcerations of mucous membrane limited to one site	Extensive or generalized bullous lesions OR Ulceration of mucous membrane involving two or more distinct mucosal sites OR Stevens-Johnson syndrome OR Toxic epidermal necrolysis

Endocrine and Metabolic

PARAMETER	GRADE 1 MILD	GRADE 2 MODERATE	GRADE 3 SEVERE	GRADE 4 POTENTIALLY LIFE-THREATENING
**Diabetes Mellitus**	Controlled without medication	Controlled with medication OR Modification of current medication regimen	Uncontrolled despite treatment modification OR Hospitalization for immediate glucose control indicated	Life-threatening consequences (e.g., ketoacidosis, hyperosmolar non-ketotic coma, end organ failure)
**Gynecomastia**	Detectable by study participant, caregiver, or physician AND Causing no or minimal interference with usual social & functional activities	Obvious on visual inspection AND Causing pain with greater than minimal interference with usual social & functional activities	Disfiguring changes AND Symptoms requiring intervention or causing inability to perform usual social & functional activities	NA
**Hyperthyroidism**	No symptoms AND Abnormal laboratory value	Symptoms causing greater than minimal interference with usual social & functional activities OR Thyroid suppression therapy indicated	Symptoms causing inability to perform usual social & functional activities OR Uncontrolled despite treatment modification	Life-threatening consequences (e.g., thyroid storm)
**Hypothyroidism**	No symptoms AND Abnormal laboratory value	Symptoms causing greater than minimal interference with usual social & functional activities OR Thyroid replacement therapy indicated	Symptoms causing inability to perform usual social & functional activities OR Uncontrolled despite treatment modification	Life-threatening consequences (e.g., myxedema coma)
**Lipoatrophy**[4]	Detectable by study participant, caregiver, or physician AND Causing no or minimal interference with usual social & functional activities	Obvious on visual inspection AND Causing greater than minimal interference with usual social & functional activities	Disfiguring changes	NA

[4] Definition: A disorder characterized by fat loss in the face, extremities, and buttocks.

Endocrine and Metabolic

PARAMETER	GRADE 1 MILD	GRADE 2 MODERATE	GRADE 3 SEVERE	GRADE 4 POTENTIALLY LIFE-THREATENING
**Lipohypertrophy**[5]	Detectable by study participant, caregiver, or physician AND Causing no or minimal interference with usual social & functional activities	Obvious on visual inspection AND Causing greater than minimal interference with usual social & functional activities	Disfiguring changes	NA

[5] Definition: A disorder characterized by abnormal fat accumulation on the back of the neck, breasts, and abdomen.

Gastrointestinal

PARAMETER	GRADE 1 MILD	GRADE 2 MODERATE	GRADE 3 SEVERE	GRADE 4 POTENTIALLY LIFE-THREATENING
**Anorexia**	Loss of appetite without decreased oral intake	Loss of appetite associated with decreased oral intake without significant weight loss	Loss of appetite associated with significant weight loss	Life-threatening consequences OR Aggressive intervention indicated (e.g., tube feeding, total parenteral nutrition)
**Ascites**	No symptoms	Symptoms AND Intervention indicated (e.g., diuretics, therapeutic paracentesis)	Symptoms recur or persist despite intervention	Life-threatening consequences
**Bloating or Distension** *Report only one*	Symptoms causing no or minimal interference with usual social & functional activities	Symptoms causing greater than minimal interference with usual social & functional activities	Symptoms causing inability to perform usual social & functional activities	NA
**Cholecystitis**	NA	Symptoms AND Medical intervention indicated	Radiologic, endoscopic, or operative intervention indicated	Life-threatening consequences (e.g., sepsis, perforation)
**Constipation**	NA	Persistent constipation requiring regular use of dietary modifications, laxatives, or enemas	Obstipation with manual evacuation indicated	Life-threatening consequences (e.g., obstruction)
**Diarrhea** *≥ 1 year of age*	Transient or intermittent episodes of unformed stools OR Increase of ≤ 3 stools over baseline per 24-hour period	Persistent episodes of unformed to watery stools OR Increase of 4 to 6 stools over baseline per 24-hour period	Increase of ≥ 7 stools per 24-hour period OR IV fluid replacement indicated	Life-threatening consequences (e.g., hypotensive shock)
< 1 year of age	Liquid stools (more unformed than usual) but usual number of stools	Liquid stools with increased number of stools OR Mild dehydration	Liquid stools with moderate dehydration	Life-threatening consequences (e.g., liquid stools resulting in severe dehydration, hypotensive shock)
**Dysphagia or Odynophagia** *Report only one and specify location*	Symptoms but able to eat usual diet	Symptoms causing altered dietary intake with no intervention indicated	Symptoms causing severely altered dietary intake with intervention indicated	Life-threatening reduction in oral intake

Gastrointestinal

**Gastrointestinal Bleeding**	Not requiring intervention other than iron supplement	Endoscopic intervention indicated	Transfusion indicated	Life-threatening consequences (e.g., hypotensive shock)

Gastrointestinal

PARAMETER	GRADE 1 MILD	GRADE 2 MODERATE	GRADE 3 SEVERE	GRADE 4 POTENTIALLY LIFE-THREATENING
**Mucositis or Stomatitis** *Report only one and specify location*	Mucosal erythema	Patchy pseudomembranes or ulcerations	Confluent pseudomembranes or ulcerations OR Mucosal bleeding with minor trauma	Life-threatening consequences (e.g., aspiration, choking) OR Tissue necrosis OR Diffuse spontaneous mucosal bleeding
**Nausea**	Transient (< 24 hours) or intermittent AND No or minimal interference with oral intake	Persistent nausea resulting in decreased oral intake for 24 to 48 hours	Persistent nausea resulting in minimal oral intake for > 48 hours OR Rehydration indicated (e.g., IV fluids)	Life-threatening consequences (e.g., hypotensive shock)
**Pancreatitis**	NA	Symptoms with hospitalization not indicated	Symptoms with hospitalization indicated	Life-threatening consequences (e.g., circulatory failure, hemorrhage, sepsis)
**Perforation** (colon or rectum)	NA	NA	Interventio n indicated	Life-threatening consequences
**Proctitis**	Rectal discomfort with no intervention indicated	Symptoms causing greater than minimal interference with usual social & functional activities OR Medical intervention indicated	Symptoms causing inability to perform usual social & functional activities OR Operative intervention indicated	Life-threatening consequences (e.g., perforation)
**Rectal Discharge**	Visible discharge	Discharge requiring the use of pads	NA	NA
**Vomiting**	Transient or intermittent AND No or minimal interference with oral intake	Frequent episodes with no or mild dehydration	Persistent vomiting resulting in orthostatic hypotension OR Aggressive rehydration indicated (e.g., IV fluids)	Life-threatening consequences (e.g., hypotensive shock)

Musculoskeletal

PARAMETER	GRADE 1 MILD	GRADE 2 MODERATE	GRADE 3 SEVERE	GRADE 4 POTENTIALLY LIFE-THREATENING
**Arthralgia**	Joint pain causing no or minimal interference with usual social & functional activities	Joint pain causing greater than minimal interference with usual social & functional activities	Joint pain causing inability to perform usual social & functional activities	Disabling joint pain causing inability to perform basic self-care functions
**Arthritis**	Stiffness or joint swelling causing no or minimal interference with usual social & functional activities	Stiffness or joint swelling causing greater than minimal interference with usual social & functional activities	Stiffness or joint swelling causing inability to perform usual social & functional activities	Disabling joint stiffness or swelling causing inability to perform basic self-care functions
**Myalgia** (generalized)	Muscle pain causing no or minimal interference with usual social & functional activities	Muscle pain causing greater than minimal interference with usual social & functional activities	Muscle pain causing inability to perform usual social & functional activities	Disabling muscle pain causing inability to perform basic self-care functions
**Osteonecrosis**	NA	No symptoms but with radiographic findings AND No operative intervention indicated	Bone pain with radiographic findings OR Operative intervention indicated	Disabling bone pain with radiographic findings causing inability to perform basic self-care functions
**Osteopenia**[6] *≥ 30 years of age*	BMD t-score -2.5 to -1	NA	NA	NA
< 30 years of age	BMD z-score -2 to -1	NA	NA	NA
**Osteoporosis**[6] *≥ 30 years of age*	NA	BMD t-score < -2.5	Pathologic fracture (e.g., compression fracture causing loss of vertebral height)	Pathologic fracture causing life-threatening consequences
< 30 years of age	NA	BMD z-score < -2	Pathologic fracture (e.g., compression fracture causing loss of vertebral height)	Pathologic fracture causing life-threatening consequences

[6] BMD t and z scores can be found in: Kanis JA on behalf of the World Health Organization Scientific Group (2007). Assessment of osteoporosis at the primary health-care level. Technical Report. World Health Organization Collaborating Centre for
Metabolic Bone Diseases, University of Sheffield, UK. 2007: Printed by the University of Sheffield.

Neurologic

PARAMETER	GRADE 1 MILD	GRADE 2 MODERATE	GRADE 3 SEVERE	GRADE 4 POTENTIALLY LIFE-THREATENING
**Acute CNS Ischemia**	NA	NA	Transient ischemic attack	Cerebral vascular accident (e.g., stroke with neurological deficit)
**Altered Mental Status** (for Dementia, see *Cognitive, Behavioral, or Attentional Disturbance* below)	Changes causing no or minimal interference with usual social & functional activities	Mild lethargy or somnolence causing greater than minimal interference with usual social & functional activities	Confusion, memory impairment, lethargy, or somnolence causing inability to perform usual social & functional activities	Delirium OR Obtundation OR Coma
**Ataxia**	Symptoms causing no or minimal interference with usual social & functional activities OR No symptoms with ataxia detected on examination	Symptoms causing greater than minimal interference with usual social & functional activities	Symptoms causing inability to perform usual social & functional activities	Disabling symptoms causing inability to perform basic self-care functions
**Cognitive, Behavioral, or Attentional Disturbance** (includes dementia and attention deficit disorder) *Specify type, if applicable*	Disability causing no or minimal interference with usual social & functional activities OR Specialized resources not indicated	Disability causing greater than minimal interference with usual social & functional activities OR Specialized resources on part-time basis indicated	Disability causing inability to perform usual social & functional activities OR Specialized resources on a full-time basis indicated	Disability causing inability to perform basic self-care functions OR Institutionalization indicated
**Developmental Delay** *< 18 years of age* *Specify type, if applicable*	Mild developmental delay, either motor or cognitive, as determined by comparison with a developmental screening tool appropriate for the setting	Moderate developmental delay, either motor or cognitive, as determined by comparison with a developmental screening tool appropriate for the setting	Severe developmental delay, either motor or cognitive, as determined by comparison with a developmental screening tool appropriate for the setting	Developmental regression, either motor or cognitive, as determined by comparison with a developmental screening tool appropriate for the setting
**Headache**	Symptoms causing no or minimal interference with usual social & functional activities	Symptoms causing greater than minimal interference with usual social & functional activities	Symptoms causing inability to perform usual social & functional activities	Symptoms causing inability to perform basic self-care functions OR Hospitalization indicated OR Headache with significant impairment of alertness or other neurologic function

Neurologic

PARAMETER	GRADE 1 MILD	GRADE 2 MODERATE	GRADE 3 SEVERE	GRADE 4 POTENTIALLY LIFE-THREATENING
**Neuromuscular Weakness** (includes myopathy and neuropathy) *Specify type, if applicable*	Minimal muscle weakness causing no or minimal interference with usual social & functional activities OR No symptoms with decreased strength on examination	Muscle weakness causing greater than minimal interference with usual social & functional activities	Muscle weakness causing inability to perform usual social & functional activities	Disabling muscle weakness causing inability to perform basic self-care functions OR Respiratory muscle weakness impairing ventilation
**Neurosensory Alteration** (includes paresthesia and painful neuropathy) *Specify type, if applicable*	Minimal paresthesia causing no or minimal interference with usual social & functional activities OR No symptoms with sensory alteration on examination	Sensory alteration or paresthesia causing greater than minimal interference with usual social & functional activities	Sensory alteration or paresthesia causing inability to perform usual social & functional activities	Disabling sensory alteration or paresthesia causing inability to perform basic self-care functions
**Seizures** ***New Onset Seizure*** *≥ 18 years of age*	NA	NA	1 to 3 seizures	Prolonged and repetitive seizures (e.g., status epilepticus) OR Difficult to control (e.g., refractory epilepsy)
< 18 years of age (includes new or pre- existing febrile seizures)	Seizure lasting < 5 minutes with < 24 hours postictal state	Seizure lasting 5 to < 20 minutes with < 24 hours postictal state	Seizure lasting ≥ 20 minutes OR > 24 hours postictal state	Prolonged and repetitive seizures (e.g., status epilepticus) OR Difficult to control (e.g., refractory epilepsy)
Pre-existing Seizure	NA	Increased frequency from previous level of control without change in seizure character	Change in seizure character either in duration or quality (e.g., severity or focality)	Prolonged and repetitive seizures (e.g., status epilepticus) OR Difficult to control (e.g., refractory epilepsy)
**Syncope**	Near syncope without loss of consciousness (e.g., pre-syncope)	Loss of consciousness with no intervention indicated	Loss of consciousness AND Hospitalization or intervention required	NA

Pregnancy, Puerperium, and Perinatal

PARAMETER	GRADE 1 MILD	GRADE 2 MODERATE	GRADE 3 SEVERE	GRADE 4 POTENTIALLY LIFE-THREATENING
**Stillbirth** (report using mother's participant ID)<br>*Report only one*	NA	NA	Fetal death occurring at ≥ 20 weeks gestation	NA
**Preterm Birth** (report using mother's participant ID)	Live birth at 34 to < 37 weeks gestational age	Live birth at 28 to < 34 weeks gestational age	Live birth at 24 to < 28 weeks gestational age	Live birth at < 24 weeks gestational age
**Spontaneous Abortion or Miscarriage**[7] (report using mother's participant ID)<br>*Report only one*	Chemical pregnanc y	Uncomplicated spontaneous abortion or miscarriage	Complicated spontaneous abortion or miscarriage	NA

[7] Definition: A pregnancy loss occurring at < 20 weeks gestational age.

Psychiatric

PARAMETER	GRADE 1 MILD	GRADE 2 MODERATE	GRADE 3 SEVERE	GRADE 4 POTENTIALLY LIFE-THREATENING
**Insomnia**	Mild difficulty falling asleep, staying asleep, or waking up early causing no or minimal interference with usual social & functional activities	Moderate difficulty falling asleep, staying asleep, or waking up early causing more than minimal interference with usual social & functional activities	Severe difficulty falling asleep, staying asleep, or waking up early causing inability to perform usual social & functional activities requiring intervention or hospitalization	NA
**Psychiatric Disorders** (includes anxiety, depression, mania, and psychosis)<br>*Specify disorder*	Symptoms with intervention not indicated OR Behavior causing no or minimal interference with usual social & functional activities	Symptoms with intervention indicated OR Behavior causing greater than minimal interference with usual social & functional activities	Symptoms with hospitalization indicated OR Behavior causing inability to perform usual social & functional activities	Threatens harm to self or others OR Acute psychosis OR Behavior causing inability to perform basic self-care functions
**Suicidal Ideation or Attempt**<br>*Report only one*	Preoccupied with thoughts of death AND No wish to kill oneself	Preoccupied with thoughts of death AND Wish to kill oneself with no specific plan or intent	Thoughts of killing oneself with partial or complete plans but no attempt to do so OR Hospitalization indicated	Suicide attempted

Respiratory

PARAMETER	GRADE 1 MILD	GRADE 2 MODERATE	GRADE 3 SEVERE	GRADE 4 POTENTIALLY LIFE-THREATENING
**Acute Bronchospasm**	Forced expiratory volume in 1 second or peak flow reduced to ≥ 70 to < 80% OR Mild symptoms with intervention not indicated	Forced expiratory volume in 1 second or peak flow 50 to < 70% OR Symptoms with intervention indicated OR Symptoms causing greater than minimal interference with usual social & functional activities	Forced expiratory volume in 1 second or peak flow 25 to < 50% OR Symptoms causing inability to perform usual social & functional activities	Forced expiratory volume in 1 second or peak flow < 25% OR Life-threatening respiratory or hemodynamic compromise OR Intubation
**Dyspnea or Respiratory Distress** *Report only one*	Dyspnea on exertion with no or minimal interference with usual social & functional activities OR Wheezing OR Minimal increase in respiratory rate for age	Dyspnea on exertion causing greater than minimal interference with usual social & functional activities OR Nasal flaring OR Intercostal retractions OR Pulse oximetry 90 to < 95%	Dyspnea at rest causing inability to perform usual social & functional activities OR Pulse oximetry < 90%	Respiratory failure with ventilator support indicated (e.g., CPAP, BPAP, intubation)

Sensory

PARAMETER	GRADE 1 MILD	GRADE 2 MODERATE	GRADE 3 SEVERE	GRADE 4 POTENTIALLY LIFE-THREATENING
**Hearing Loss**<br>*≥ 12 years of age*	NA	Hearing aid or intervention not indicated	Hearing aid or intervention indicated	Profound bilateral hearing loss (> 80 dB at 2 kHz and above) OR Non-serviceable hearing (i.e., >50 dB audiogram and <50% speech discrimination)
< 12 years of age (based on a 1, 2, 3, 4, 6 and 8 kHz audiogram)	> 20 dB hearing loss at ≤ 4 kHz	> 20 dB hearing loss at > 4 kHz	> 20 dB hearing loss at ≥ 3 kHz in one ear with additional speech language related services indicated (where available) OR Hearing loss sufficient to indicate therapeutic intervention, including hearing aids	Audiologic indication for cochlear implant and additional speech-language related services indicated (where available)
**Tinnitus**	Symptoms causing no or minimal interference with usual social & functional activities with intervention not indicated	Symptoms causing greater than minimal interference with usual social & functional activities with intervention indicated	Symptoms causing inability to perform usual social & functional activities	NA
**Uveitis**	No symptoms AND Detectable on examination	Anterior uveitis with symptoms OR Medical intervention indicated	Posterior or pan-uveitis OR Operative intervention indicated	Disabling visual loss in affected eye(s)
**Vertigo**	Vertigo causing no or minimal interference with usual social & functional activities	Vertigo causing greater than minimal interference with usual social & functional activities	Vertigo causing inability to perform usual social & functional activities	Disabling vertigo causing inability to perform basic self-care functions
**Visual Changes** (assessed from baseline)	Visual changes causing no or minimal interference with usual social & functional activities	Visual changes causing greater than minimal interference with usual social & functional activities	Visual changes causing inability to perform usual social & functional activities	Disabling visual loss in affected eye(s)

Systemic

PARAMETER	GRADE 1 MILD	GRADE 2 MODERATE	GRADE 3 SEVERE	GRADE 4 POTENTIALLY LIFE-THREATENING
**Acute Allergic Reaction**	Localized urticaria (wheals) with no medical intervention indicated	Localized urticaria with intervention indicated OR Mild angioedema with no intervention indicated	Generalized urticaria OR Angioedema with intervention indicated OR Symptoms of mild bronchospasm	Acute anaphylaxis OR Life-threatening bronchospasm OR Laryngeal edema
**Chills**	Symptoms causing no or minimal interference with usual social & functional activities	Symptoms causing greater than minimal interference with usual social & functional activities	Symptoms causing inability to perform usual social & functional activities	NA
**Cytokine Release Syndrome**[8]	Mild signs and symptoms AND Therapy (i.e., antibody infusion) interruption not indicated	Therapy (i.e., antibody infusion) interruption indicated AND Responds promptly to symptomatic treatment OR Prophylactic medications indicated for ≤ 24 hours	Prolonged severe signs and symptoms OR Recurrence of symptoms following initial improvement	Life-threatening consequences (e.g., requiring pressor or ventilator support)
**Fatigue or Malaise** *Report only one*	Symptoms causing no or minimal interference with usual social & functional activities	Symptoms causing greater than minimal interference with usual social & functional activities	Symptoms causing inability to perform usual social & functional activities	Incapacitating symptoms of fatigue or malaise causing inability to perform basic self-care functions
**Fever** (non-axillary temperatures only)	38.0 to < 38.6°C or 100.4 to < 101.5°F	≥ 38.6 to < 39.3°C or ≥ 101.5 to < 102.7°F	≥ 39.3 to < 40.0°C or ≥ 102.7 to < 104.0°F	≥ 40.0°C or ≥ 104.0°F
**Pain**[9] (not associated with study agent injections and not specified elsewhere) *Specify location*	Pain causing no or minimal interference with usual social & functional activities	Pain causing greater than minimal interference with usual social & functional activities	Pain causing inability to perform usual social & functional activities	Disabling pain causing inability to perform basic self-care functions OR Hospitalization indicated

[8] Definition: A disorder characterized by nausea, headache, tachycardia, hypotension, rash, and/or shortness of breath.
[9] For pain associated with injections or infusions, see the *Site Reactions to Injections and Infusions* section (page 23).

Systemic

**Serum Sickness**[10]	Mild signs and symptoms	Moderate signs and symptoms AND Intervention indicated (e.g., antihistamines)	Severe signs and symptoms AND Higher level intervention indicated (e.g., steroids or IV fluids)	Life-threatening consequences (e.g., requiring pressor or ventilator support)
**Underweight**[11] *> 5 to 19 years of age*	WHO BMI z-score < -1 to -2	WHO BMI z-score < -2 to -3	WHO BMI z-score < -3	WHO BMI z-score < -3 with life-threatening consequences
2 to 5 years of age	WHO Weight-for-height z-score < -1 to -2	WHO Weight-for-height z-score < -2 to -3	WHO Weight-for-height z-score < -3	WHO Weight-for-height z-score < -3 with life-threatening consequences
< 2 years of age	WHO Weight-for-length z-score < -1 to -2	WHO Weight-for-length z-score < -2 to -3	WHO Weight-for-length z-score < -3	WHO Weight-for-length z-score < -3 with life-threatening consequences
**Unintentional Weight Loss** (excludes postpartum weight loss)	NA	5 to < 9% loss in body weight from baseline	≥ 9 to < 20% loss in body weight from baseline	≥ 20% loss in body weight from baseline OR Aggressive intervention indicated (e.g., tube feeding, total parenteral nutrition)

[10] Definition: A disorder characterized by fever, arthralgia, myalgia, skin eruptions, lymphadenopathy, marked discomfort, and/or dyspnea.
[11] WHO reference tables may be accessed by clicking the desired age range or by accessing the following URLs: http://www.who.int/growthref/who2007_bmi_for_age/en/ for participants > 5 to 19 years of age and http://www.who.int/childgrowth/standards/chart_catalogue/en/ for those ≤ 5 years of age.

Urinary

PARAMETER	GRADE 1 MILD	GRADE 2 MODERATE	GRADE 3 SEVERE	GRADE 4 POTENTIALLY LIFE-THREATENING
**Urinary Tract Obstruction**	NA	Signs or symptoms of urinary tract obstruction without hydronephrosis or renal dysfunction	Signs or symptoms of urinary tract obstruction with hydronephrosis or renal dysfunction	Obstruction causing life-threatening consequences

Site Reactions to Injections and Infusions

PARAMETER	GRADE 1 MILD	GRADE 2 MODERATE	GRADE 3 SEVERE	GRADE 4 POTENTIALLY LIFE-THREATENING
**Injection Site Pain or Tenderness** *Report only one*	Pain or tenderness causing no or minimal limitation of use of limb	Pain or tenderness causing greater than minimal limitation of use of limb	Pain or tenderness causing inability to perform usual social & functional activities	Pain or tenderness causing inability to perform basic self-care function OR Hospitalization indicated
**Injection Site Erythema or Redness** [12] *Report only one* *> 15 years of age*	2.5 to < 5 cm in diameter OR 6.25 to < 25 cm^2 surface area AND Symptoms causing no or minimal interference with usual social & functional activities	≥ 5 to < 10 cm in diameter OR ≥ 25 to < 100 cm^2 surface area OR Symptoms causing greater than minimal interference with usual social & functional activities	≥ 10 cm in diameter OR ≥ 100 cm^2 surface area OR Ulceration OR Secondary infection OR Phlebitis OR Sterile abscess OR Drainage OR Symptoms causing inability to perform usual social & functional activities	Potentially life-threatening consequences (e.g., abscess, exfoliative dermatitis, necrosis involving dermis or deeper tissue)
≤ 15 years of age	≤ 2.5 cm in diameter	> 2.5 cm in diameter with < 50% surface area of the extremity segment involved (e.g., upper arm or thigh)	≥ 50% surface area of the extremity segment involved (e.g., upper arm or thigh) OR Ulceration OR Secondary infection OR Phlebitis OR Sterile abscess OR Drainage	Potentially life-threatening consequences (e.g., abscess, exfoliative dermatitis, necrosis involving dermis or deeper tissue)
**Injection Site Induration or Swelling** *Report only one* *> 15 years of age*	Same as for **Injection Site Erythema or Redness,** *> 15 years of age*	Same as for **Injection Site Erythema or Redness,** *> 15 years of age*	Same as for **Injection Site Erythema or Redness,** *> 15 years of age*	Same as for **Injection Site Erythema or Redness,** *> 15 years of age*
≤ 15 years of age	Same as for **Injection Site Erythema or Redness,** *≤ 15 years of age*	Same as for **Injection Site Erythema or Redness,** *≤ 15 years of age*	Same as for **Injection Site Erythema or Redness,** *≤ 15 years of age*	Same as for **Injection Site Erythema or Redness,** *≤ 15 years of age*
**Injection Site Pruritus**	Itching localized to the injection site that is relieved spontaneously or in < 48 hours of treatment	Itching beyond the injection site that is not generalized OR Itching localized to the injection site requiring ≥ 48 hours treatment	Generalized itching causing inability to perform usual social & functional activities	NA

[12] Injection Site Erythema or Redness should be evaluated and graded using the greatest single diameter or measured surface area.

*Laboratory Values**

Chemistries

PARAMETER	GRADE 1<br>MILD	GRADE 2<br>MODERATE	GRADE 3<br>SEVERE	GRADE 4<br>POTENTIALLY LIFE-THREATENING
**Acidosis**	NA	pH ≥ 7.3 to < LLN	pH < 7.3 without life-threatening consequences	pH < 7.3 with life-threatening consequences
**Albumin, Low**<br>(g/dL; *g/L*)	3.0 to < LLN<br>*30 to < LLN*	≥ 2.0 to < 3.0<br>*≥ 20 to < 30*	< 2.0<br>*< 20*	NA
**Alkaline Phosphatase, High**	1.25 to < 2.5 x ULN	2.5 to < 5.0 x ULN	5.0 to < 10.0 x ULN	≥ 10.0 x ULN
**Alkalosis**	NA	pH > ULN to ≤ 7.5	pH > 7.5 without life-threatening consequences	pH > 7.5 with life-threatening consequences
**ALT or SGPT, High**<br>*Report only one*	1.25 to < 2.5 x ULN	2.5 to < 5.0 x ULN	5.0 to < 10.0 x ULN	≥ 10.0 x ULN
**Amylase (Pancreatic) or Amylase (Total), High**<br>*Report only one*	1.1 to < 1.5 x ULN	1.5 to < 3.0 x ULN	3.0 to < 5.0 x ULN	≥ 5.0 x ULN
**AST or SGOT, High**<br>*Report only one*	1.25 to < 2.5 x ULN	2.5 to < 5.0 x ULN	5.0 to < 10.0 x ULN	≥ 10.0 x ULN
**Bicarbonate, Low**<br>(mEq/L; *mmol/L*)	16.0 to < LLN<br>*16.0 to < LLN*	11.0 to < 16.0<br>*11.0 to < 16.0*	8.0 to < 11.0<br>*8.0 to < 11.0*	< 8.0<br>*< 8.0*
**Bilirubin**<br>***Direct Bilirubin[13], High***<br>*> 28 days of age*	NA	NA	> ULN with other signs and symptoms of hepatotoxicity.	> ULN with life-threatening consequences (e.g., signs and symptoms of liver failure)
≤ 28 days of age	ULN to ≤ 1 mg/dL	> 1 to ≤ 1.5 mg/dL	> 1.5 to ≤ 2 mg/dL	> 2 mg/dL
Total Bilirubin, High<br>*> 28 days of age*	1.1 to < 1.6 x ULN	1.6 to < 2.6 x ULN	2.6 to < 5.0 x ULN	≥ 5.0 x ULN
≤ 28 days of age	See Appendix A. Total Bilirubin for Term and Preterm Neonates	See Appendix A. Total Bilirubin for Term and Preterm Neonates	See Appendix A. Total Bilirubin for Term and Preterm Neonates	See Appendix A. Total Bilirubin for Term and Preterm Neonates

*Reminder: An asymptomatic abnormal laboratory finding without an accompanying AE should not be reported to DAIDS in an expedited time frame unless it meets protocol-specific reporting requirements.

[13] Direct bilirubin > 1.5 mg/dL in a participant < 28 days of age should be graded as grade 2, if < 10% of the total bilirubin.

Chemistries

PARAMETER	GRADE 1 MILD	GRADE 2 MODERATE	GRADE 3 SEVERE	GRADE 4 POTENTIALLY LIFE-THREATENING
**Calcium, High** (mg/dL; *mmol/L*) *≥ 7 days of age*	10.6 to < 11.5<br>*2.65 to < 2.88*	11.5 to < 12.5<br>*2.88 to < 3.13*	12.5 to < 13.5<br>*3.13 to < 3.38*	≥ 13.5<br>*≥ 3.38*
< 7 days of age	11.5 to < 12.4<br>*2.88 to < 3.10*	12.4 to < 12.9<br>*3.10 to < 3.23*	12.9 to < 13.5<br>*3.23 to < 3.38*	≥ 13.5<br>*≥ 3.38*
**Calcium (Ionized), High** (mg/dL; *mmol/L*)	> ULN to < 6.0<br>*> ULN to < 1.5*	6.0 to < 6.4<br>*1.5 to < 1.6*	6.4 to < 7.2<br>*1.6 to < 1.8*	≥ 7.2<br>*≥ 1.8*
**Calcium, Low** (mg/dL; *mmol/L*) *≥ 7 days of age*	7.8 to < 8.4<br>*1.95 to < 2.10*	7.0 to < 7.8<br>*1.75 to < 1.95*	6.1 to < 7.0<br>*1.53 to < 1.75*	< 6.1<br>*< 1.53*
< 7 days of age	6.5 to < 7.5<br>*1.63 to < 1.88*	6.0 to < 6.5<br>*1.50 to < 1.63*	5.50 to < 6.0<br>*1.38 to < 1.50*	< 5.50<br>*< 1.38*
**Calcium (Ionized), Low** (mg/dL; *mmol/L*)	< LLN to 4.0<br>*< LLN to 1.0*	3.6 to < 4.0<br>*0.9 to < 1.0*	3.2 to < 3.6<br>*0.8 to < 0.9*	< 3.2<br>*< 0.8*
**Cardiac Troponin I, High**	NA	NA	NA	Levels consistent with myocardial infarction or unstable angina as defined by the local laboratory
**Creatine Kinase, High**	3 to < 6 x ULN	6 to < 10x ULN	10 to < 20 x ULN	≥ 20 x ULN
**Creatinine, High** **Report only one*	1.1 to 1.3 x ULN	> 1.3 to 1.8 x ULN OR Increase to 1.3 to < 1.5 x participant's baseline	> 1.8 to < 3.5 x ULN OR Increase to 1.5 to < 2.0 x participant's baseline	≥ 3.5 x ULN OR Increase of ≥ 2.0 x participant's baseline
**Creatinine Clearance [14] or eGFR, Low** **Report only one*	NA	< 90 to 60 ml/min or ml/min/1.73 m^2 OR 10 to < 30% decrease from participant's baseline	< 60 to 30 ml/min or ml/min/1.73 m^2 OR 30 to < 50% decrease from participant's baseline	< 30 ml/min or ml/min/1.73 m^2 OR ≥ 50% decrease from participant's baseline or dialysis needed
**Glucose** (mg/dL; *mmol/L*) ***Fasting, High***	110 to 125<br>*6.11 to < 6.95*	> 125 to 250<br>*6.95 to < 13.89*	> 250 to 500<br>*13.89 to < 27.75*	≥ 500<br>*≥ 27.75*
Nonfasting, High	116 to 160<br>*6.44 to < 8.89*	> 160 to 250<br>*8.89 to < 13.89*	> 250 to 500<br>*13.89 to < 27.75*	≥ 500<br>*≥ 27.75*

[14] Use the applicable formula (i.e., Cockcroft-Gault in mL/min or Schwartz, MDRD, CKD-Epi in mL/min/1.73m2). Sites should choose the method defined in their study and when not specified, use the method most relevant to the study population.
*Reminder: Choose the method that selects for the higher grade.

Chemistries

PARAMETER	GRADE 1 MILD	GRADE 2 MODERATE	GRADE 3 SEVERE	GRADE 4 POTENTIALLY LIFE-THREATENING
**Glucose, Low** (mg/dL; *mmol/L*) *≥ 1 month of age*	55 to 64 *3.05 to <3.55*	40 to < 55 *2.22 to < 3.05*	30 to < 40 *1.67 to < 2.22*	< 30 *< 1.67*
< 1 month of age	50 to 54 *2.78 to < 3.00*	40 to < 50 *2.22 to < 2.78*	30 to < 40 *1.67 to < 2.22*	< 30 *< 1.67*
**Lactate, High**	ULN to < 2.0 x ULN without acidosis	≥ 2.0 x ULN without acidosis	Increased lactate with pH < 7.3 without life-threatening consequences	Increased lactate with pH < 7.3 with life- threatening consequences
**Lipase, High**	1.1 to < 1.5 x ULN	1.5 to < 3.0 x ULN	3.0 to < 5.0 x ULN	≥ 5.0 x ULN
**Lipid Disorders** (mg/dL; *mmol/L*) ***Cholesterol, Fasting, High*** *≥ 18 years of age*	200 to < 240 *5.18 to < 6.19*	240 to < 300 *6.19 to < 7.77*	≥ 300 *≥ 7.77*	NA
< 18 years of age	170 to < 200 *4.40 to < 5.15*	200 to < 300 *5.15 to < 7.77*	≥ 300 *≥ 7.77*	NA
LDL, Fasting, High *≥ 18 years of age*	130 to < 160 *3.37 to < 4.12*	160 to < 190 *4.12 to < 4.90*	≥ 190 *≥ 4.90*	NA
> 2 to < 18 years of age	110 to < 130 *2.85 to < 3.34*	130 to < 190 *3.34 to < 4.90*	≥ 190 *≥ 4.90*	NA
Triglycerides, Fasting, High	150 to 300 *1.71 to 3.42*	>300 to 500 *>3.42 to 5.7*	>500 to < 1,000 *>5.7 to 11.4*	> 1,000 *> 11.4*
**Magnesium[15], Low** (mEq/L; *mmol/L*)	1.2 to < 1.4 *0.60 to < 0.70*	0.9 to < 1.2 *0.45 to < 0.60*	0.6 to < 0.9 *0.30 to < 0.45*	< 0.6 *< 0.30*
**Phosphate, Low** (mg/dL; *mmol/L*) *> 14 years of age*	2.0 to < LLN *0.65 to < LLN*	1.4 to < 2.0 *0.45 to < 0.65*	1.0 to < 1.4 *0.32 to < 0.45*	< 1.0 *< 0.32*
1 to 14 years of age	3.0 to < 3.5 *0.97 to < 1.13*	2.5 to < 3.0 *0.81 to < 0.97*	1.5 to < 2.5 *0.48 to < 0.81*	< 1.5 *< 0.48*
< 1 year of age	3.5 to < 4.5 *1.13 to < 1.45*	2.5 to < 3.5 *0.81 to < 1.13*	1.5 to < 2.5 *0.48 to < 0.81*	< 1.5 *< 0.48*
**Potassium, High** (mEq/L; *mmol/L*)	5.6 to < 6.0 *5.6 to < 6.0*	6.0 to < 6.5 *6.0 to < 6.5*	6.5 to < 7.0 *6.5 to < 7.0*	≥ 7.0 *≥ 7.0*
**Potassium, Low** (mEq/L; *mmol/L*)	3.0 to < 3.4 *3.0 to < 3.4*	2.5 to < 3.0 *2.5 to < 3.0*	2.0 to < 2.5 *2.0 to < 2.5*	< 2.0 *< 2.0*

[15] To convert a magnesium value from mg/dL to mmol/L, laboratories should multiply by 0.4114.

Chemistries

PARAMETER	GRADE 1 MILD	GRADE 2 MODERATE	GRADE 3 SEVERE	GRADE 4 POTENTIALLY LIFE-THREATENING
**Sodium, High** (mEq/L; *mmol/L*)	146 to < 150<br>*146 to < 150*	150 to < 154<br>*150 to < 154*	154 to < 160<br>*154 to < 160*	≥ 160<br>*≥ 160*
**Sodium, Low** (mEq/L; *mmol/L*)	130 to < 135<br>*130 to < 135*	125 to < 130<br>*125 to < 130*	121 to < 125<br>*121 to < 125*	≤ 120<br>*≤ 120*
**Uric Acid, High** (mg/dL; *mmol/L*)	7.5 to < 10.0<br>*0.45 to < 0.59*	10.0 to < 12.0<br>*0.59 to < 0.71*	12.0 to < 15.0<br>*0.71 to < 0.89*	≥ 15.0<br>*≥ 0.89*

Hematology

PARAMETER	GRADE 1<br>MILD	GRADE 2<br>MODERATE	GRADE 3<br>SEVERE	GRADE 4<br>POTENTIALLY LIFE-THREATENING
**Absolute CD4+ Count, Low**<br>(cell/mm^3; *cells/L*)<br>*> 5 years of age (not HIV infected)*	300 to < 400<br>*300 to < 400*	200 to < 300<br>*200 to < 300*	100 to < 200<br>*100 to < 200*	< 100<br>*< 100*
**Absolute Lymphocyte Count, Low**<br>(cell/mm^3; *cells/L*)<br>*> 5 years of age (not HIV infected)*	600 to < 650<br>*0.600 x 10^9 to < 0.650 x 10^9*	500 to < 600<br>*0.500 x 10^9 to < 0.600 x 10^9*	350 to < 500<br>*0.350 x 10^9 to < 0.500 x 10^9*	< 350<br>*< 0.350 x 10^9*
**Absolute Neutrophil Count (ANC), Low**<br>(cells/mm^3; *cells/L*)<br>*> 7 days of age*	800 to 1,000<br>*0.800 x 10^9 to 1.000 x 10^9*	600 to 799<br>*0.600 x 10^9 to 0.799 x 10^9*	400 to 599<br>*0.400 x 10^9 to 0.599 x 10^9*	< 400<br>*< 0.400 x 10^9*
2 to 7 days of age	1,250 to 1,500<br>*1.250 x 10^9 to 1.500 x 10^9*	1,000 to 1,249<br>*1.000 x 10^9 to 1.249 x 10^9*	750 to 999<br>*0.750 x 10^9 to 0.999 x 10^9*	< 750<br>*< 0.750 x 10^9*
≤ 1 day of age	4,000 to 5,000<br>*4.000 x 10^9 to 5.000 x 10^9*	3,000 to 3,999<br>*3.000 x 10^9 to 3.999 x 10^9*	1,500 to 2,999<br>*1.500 x 10^9 to 2.999 x 10^9*	< 1,500<br>*< 1.500 x 10^9*
**Fibrinogen, Decreased**<br>(mg/dL; *g/L*)	100 to < 200<br>*1.00 to < 2.00*<br>OR<br>0.75 to < 1.00 x LLN	75 to < 100<br>*0.75 to < 1.00*<br>OR<br>≥ 0.50 to < 0.75 x LLN	50 to < 75<br>*0.50 to < 0.75*<br>OR<br>0.25 to < 0.50 x LLN	< 50<br>*< 0.50*<br>OR<br>< 0.25 x LLN<br>OR Associated with gross bleeding
**Hemoglobin[16], Low**<br>(g/dL; *mmol/L*)[17]<br>*≥ 13 years of age (male only)*	10.0 to 10.9<br>*6.19 to 6.76*	9.0 to < 10.0<br>*5.57 to < 6.19*	7.0 to < 9.0<br>*4.34 to < 5.57*	< 7.0<br>*< 4.34*
≥ 13 years of age (female only)	9.5 to 10.4<br>*5.88 to 6.48*	8.5 to < 9.5<br>*5.25 to < 5.88*	6.5 to < 8.5<br>*4.03 to < 5.25*	< 6.5<br>*< 4.03*

[16] Male and female sex are defined as sex at birth. For transgender participants ≥13 years of age who have been on hormone therapy for more than 6 consecutive months, grade hemoglobin based on the gender with which they identify (i.e., a transgender female should be graded using the female sex at birth hemoglobin laboratory values).

[17] The most commonly used conversion factor to convert g/dL to mmol/L is 0.6206. For grading hemoglobin results obtained by an analytic method with a conversion factor other than 0.6206, the result must be converted to g/dL using appropriate conversion factor for the particular laboratory.

Hematology

PARAMETER	GRADE 1<br>MILD	GRADE 2<br>MODERATE	GRADE 3<br>SEVERE	GRADE 4<br>POTENTIALLY LIFE-THREATENING
57 days of age to < 13 years of age (male and female)	9.5 to 10.4<br>*5.88 to 6.48*	8.5 to < 9.5<br>*5.25 to < 5.88*	6.5 to < 8.5<br>*4.03 to < 5.25*	< 6.5<br>*< 4.03*
36 to 56 days of age (male and female)	8.5 to 9.6<br>*5.26 to 5.99*	7.0 to < 8.5<br>*4.32 to < 5.26*	6.0 to < 7.0<br>*3.72 to < 4.32*	< 6.0<br>*< 3.72*
22 to 35 days of age (male and female)	9.5 to 11.0<br>*5.88 to 6.86*	8.0 to < 9.5<br>*4.94 to < 5.88*	6.7 to < 8.0<br>*4.15 to < 4.94*	< 6.7<br>*< 4.15*
8 to ≤ 21 days of age (male and female)	11.0 to 13.0<br>*6.81 to 8.10*	9.0 to < 11.0<br>*5.57 to < 6.81*	8.0 to < 9.0<br>*4.96 to < 5.57*	< 8.0<br>*< 4.96*
≤ 7 days of age (male and female)	13.0 to 14.0<br>*8.05 to 8.72*	10.0 to < 13.0<br>*6.19 to < 8.05*	9.0 to < 10.0<br>*5.59 to < 6.19*	< 9.0<br>*< 5.59*
**INR, High**<br>(not on anticoagulation therapy)	1.1 to < 1.5 x ULN	1.5 to < 2.0 x ULN	2.0 to < 3.0 x ULN	≥ 3.0 x ULN
**Methemoglobin**<br>(% hemoglobin)	5.0 to < 10.0%	10.0 to < 15.0%	15.0 to < 20.0%	≥ 20.0%
**PTT, High**<br>(not on anticoagulation therapy)	1.1 to < 1.66 x ULN	1.66 to < 2.33 x ULN	2.33 to < 3.00 x ULN	≥ 3.00 x ULN
**Platelets, Decreased**<br>(cells/mm^3; *cells/L*)	100,000 to < 125,000<br>*100.000 x 10^9 to < 125.000 x 10^9*	50,000 to < 100,000<br>*50.000 x 10^9 to < 100.000 x 10^9*	25,000 to < 50,000<br>*25.000 x 10^9 to < 50.000 x 10^9*	< 25,000<br>*< 25.000 x 10^9*
**PT, High**<br>(not on anticoagulation therapy	1.1 to < 1.25 x ULN	1.25 to < 1.50 x ULN	1.50 to < 3.00 x ULN	≥ 3.00 x ULN
**WBC, Decreased**<br>(cells/mm^3; *cells/L*)<br>*> 7 days of age*	2,000 to 2,499<br>*2.000 x 10^9 to 2.499 x 10^9*	1,500 to 1,999<br>*1.500 x 10^9 to 1.999 x 10^9*	1,000 to 1,499<br>*1.000 x 10^9 to 1.499 x 10^9*	< 1,000<br>*< 1.000 x 10^9*
≤ 7 days of age	5,500 to 6,999<br>*5.500 x 10^9 to 6.999 x 10^9*	4,000 to 5,499<br>*4.000 x 10^9 to 5.499 x 10^9*	2,500 to 3,999<br>*2.500 x 10^9 to 3.999 x 10^9*	< 2,500<br>*< 2.500 x 10^9*

Urinalysis

PARAMETER	GRADE 1 MILD	GRADE 2 MODERATE	GRADE 3 SEVERE	GRADE 4 POTENTIALLY LIFE-THREATENING
**Glycosuria (**random collection tested by dipstick)	Trace to 1+ or ≤ 250 mg	2+ or > 250 to ≤ 500 mg	> 2+ or > 500 mg	NA
**Hematuria** (not to be reported based on dipstick findings or on blood believed to be of menstrual origin)	6 to < 10 RBCs per high power field	≥ 10 RBCs per high power field	Gross, with or without clots OR With RBC casts OR Intervention indicated	Life-threatening consequences
**Proteinuria** (random collection tested by dipstick)	1+	2+	3+ or higher	NA

Appendix D

Coordination between FRESH and LACTIN-V studies

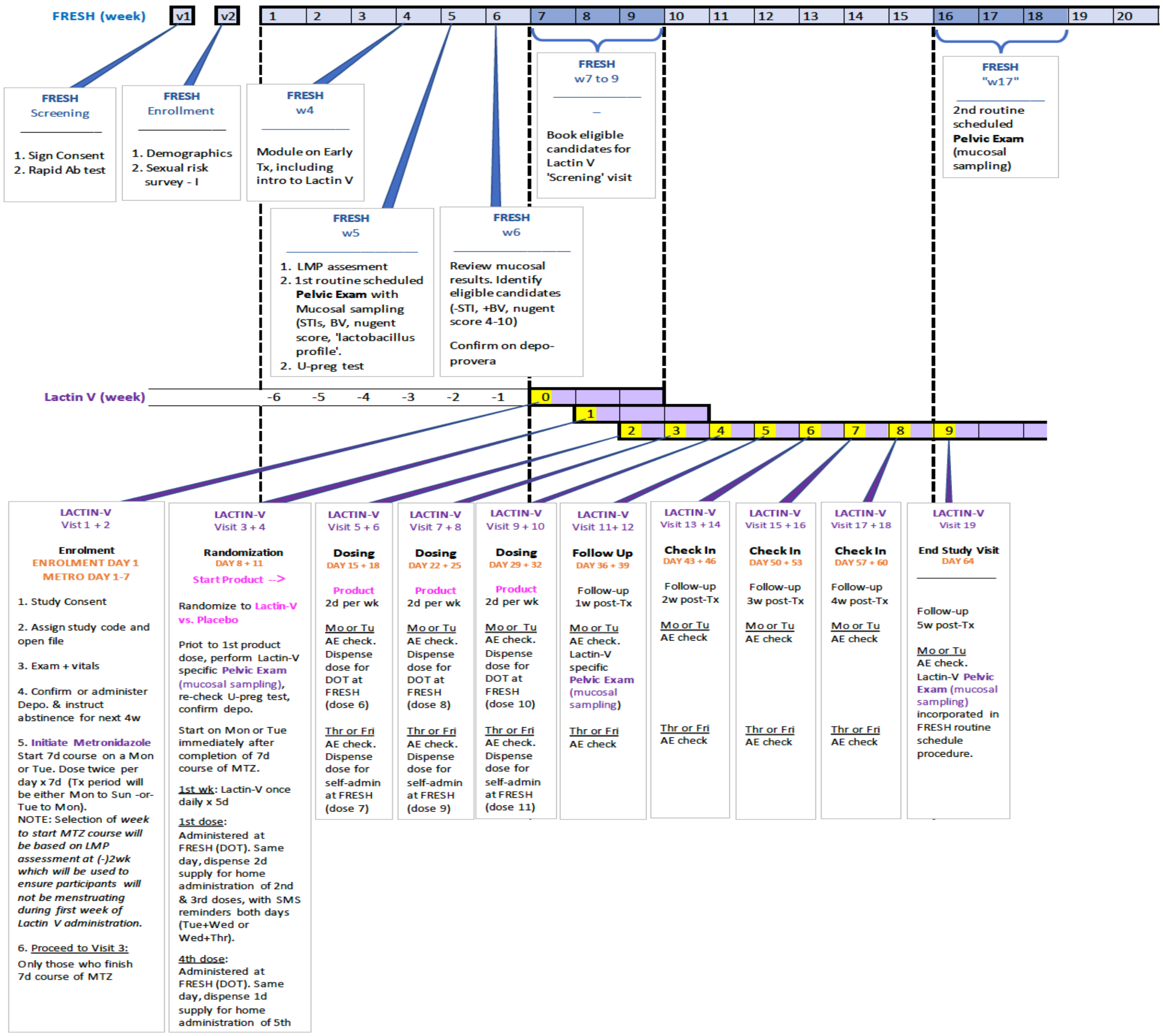

DocuSign Envelope ID: E3A8D8FC-11A1-44C7-AE49-DBDADC80E86F

STATISTICAL ANALYSIS PLAN for

Phase 2 placebo-controlled randomized trial of LACTIN-V *(Lactobacillus crispatus CTV-05)* among women at high risk of HIV acquisition in Durban, South Africa

Version 1.0

DATE: 12-MAY-2023

THIS COMMUNICATION IS PRIVILEGED AND CONFIDENTIAL

**Study Title**	Phase 2 placebo-controlled randomized trial of LACTIN-V (*Lactobacillus crispatus* CTV-05) among women at high risk of HIV acquisition in Durban, South Africa
**Development Phase:**	Phase 2b
**Products:**	LACTIN-V
**Form/Route:**	Intravaginally
**Indication Studied:**	Bacterial Vaginosis
**Sponsor:**	National Institutes of Health<br>NICHD<br>University of California, San Francisco<br>UCSF
**Clinical Trial Initiation Date:**	MAY 2021
**Clinical Trial Completion Date:**	APR 2023
**Date of the Statistical Analysis Plan:**	12-MAY-2023
**Version Number:**	1.0

Statistical Analysis Plan (SAP) Approval Form

Sponsor Reviewer Signature
This signature indicates approval for this SAP.

Typed Name/Title	Signature	Date
Anke Hemmerling, MD PhD MPH<br>Associate Adjunct Professor,<br>UCSF	DocuSigned by:<br>Anke Hemmerling<br>F1C05D752C534CC...	

Typed Name/Title	Signature	Date
Craig R Cohen, MD MPH<br>Professor,<br>UCSF	DocuSigned by:<br>Craig Cohen<br>05249986608D499...	

Author Signature
This signature indicates that appropriate and accurate information has been included in this SAP.

Typed Name/Title	Signature	Date
Dianne Weatherall,<br>Statistical Programmer,<br>DF/Net Research, Inc.	DocuSigned by:<br>Dianne Weatherall<br>3D0F52F16D0E4D6...	

DocuSign Envelope ID: E3A8D8FC-11A1-44C7-AE49-DBDADC80E86F

Revision History

Version	Date	Summary of Revision
1.0	12-MAY-2023	Initial version

TABLE OF CONTENTS

1. INTRODUCTION 9
2. STUDY OBJECTIVES 9
3. STUDY DESIGN 10
4. ANALYSIS POPULATIONS 11
5. SCHEDULE OF STUDY PROCEDURES 12
6. TABLE SHELLS 14
Table 14.1.1: Summary of Enrollment and Randomization (Screened Participants) 15
Table 14.1.2: Summary of Enrollment and Randomization Failures (Screened Participants) 16
Table 14.1.3: Analysis Populations by Treatment Group (Randomized Participants) 17
Table 14.1.4: Summary of Halting Rules by Treatment Group (Safety Population) 19
Table 14.1.5: Participant Disposition by Treatment Group (Enrolled Participants) 20
Table 14.1.6: Summary of Demographics by Treatment Group (ITT Population) 21
Table 14.1.7: Summary of Education and Employment Status by Treatment Group (ITT Population) 22
Table 14.1.8: Protocol Deviations by Treatment Group (ITT Population) 24
Table 14.1.9: Summary of Baseline Gynaecological History by Treatment Group (ITT Population) 25
Table 14.1.10: Summary of Long-Acting Reversible Contraception by Treatment Group (ITT Population) 30
Table 14.1.11: Treatment Compliance by Treatment Group and Analysis Population 31
Table 14.1.12: Product Administration by Week and Dose Number by Treatment Group (Safety Population) 32
Table 14.2.1: Bacterial Vaginosis Diagnosis by Visit 11 (Day 36) by Analysis Population and Treatment Group 34
Table 14.2.2: Bacterial Vaginosis Diagnosis by Visit 19 (Day 64) by Analysis Population and Treatment Group 35
Table 14.3.1.1: Number and Percentage of Participants Experiencing Solicited Adverse Events with 95% Confidence Intervals by Symptom and Treatment Group (Safety Population) .. 36
Table 14.3.1.2.1: Number and Percentage of Participants Experiencing Solicited Local Adverse Events with 95% Confidence Intervals by Symptom, Maximum Severity and Treatment Group (Safety Population) 37
Table 14.3.1.2.2: Number and Percentage of Participants Experiencing Solicited Local Adverse Events of Vaginal Bleeding Other Than Menstruation or Vaginal Discharge with 95% Confidence Intervals by Symptom, Maximum Severity and Treatment Group (Safety Population) 38
Table 14.3.1.2.3: Number and Percentage of Participants Experiencing a Solicited Local Adverse Event of Vaginal Bleeding Other Than Menstruation with 95% Confidence Intervals after Study Product Start (Day 8-64) Among Participants With and Without Irregular Bleeding in 3

DocuSign Envelope ID: E3A8D8FC-11A1-44C7-AE49-DBDADC80E86F

Months Prior to Start of Study Product by Treatment Group (Safety Population) 40

Table 14.3.1.3: Number and Percentage of Participants Experiencing Solicited Systemic Adverse Events with 95% Confidence Intervals by Symptom, Maximum Severity and Treatment Group (Safety Population) 42

Table 14.3.1.4: Proportion of Participants Reporting Product Related Unsolicited Adverse Events Following the First Dose of Study Product Through Visit 19 (Day 64) by Treatment Group (Safety Population) 43

Table 14.3.1.5: Number and Percentage of Participants Experiencing Unsolicited Adverse Events with 95% Confidence Intervals by MedDRA System Organ Class and Preferred Term by Treatment Group (Safety Population) 44

Table 14.3.1.6: Number and Percentage of Participants Experiencing Unsolicited Adverse Events by MedDRA System Organ Class and Preferred Term, Maximum Severity and Relationship by Treatment Group (Safety Population) 45

Table 14.3.1.7: Participants Reporting Adverse Events Occurring in 5% of Participants in Any Treatment Group by MedDRA System Organ Class and Preferred Term, and Treatment Group (Safety Population) 47

Table 14.3.4.1: Summary of Urine Pregnancy Test Results (Safety Population) 48

Table 14.3.4.2: Summary of Urinalysis Clinic Results (Safety Population) 49

Table 14.3.5.1: Gynaecological Review by Treatment Group (ITT Population) 50

Table 14.3.5.2: Acceptability Questionnaire Responses by Treatment Group (Safety Population). 53

7. FIGURE SHELLS 57

Figure 14.1.1: CONSORT Flow Diagram 58

Figure 14.2.1.1: HIV Target Cells by Group and Visit 59

Figure 14.2.1.2: Immune Cells by Group and Visit (Representative Cell Types Shown Here) 60

Figure 14.2.1.3: Inflammation Index by Treatment Group and Visit 61

Figure 14.2.1.4: Individual Cytokines by Treatment Group and Visit 62

Figure 14.2.1.5: *L. Crispatus* Relative Abundance by Treatment Group and Visit 63

Figure 14.2.1.6: Community Type (CT) Percentages by Treatment Group and Visit 64

Figure 14.2.1.7: CTV-05 Abundance Measured by qPCR by Treatment Group and Visit 65

Figure 14.2.1.8: *L. Iners* Abundance Measured by qPCR by Treatment Group and Visit 66

Figure 14.2.1.9: Total Bacterial Abundance Measured by qPCR by Treatment Group and Visit ... 67

Figure 14.3.1.1: Maximum Severity of Solicited Local Adverse Events by Symptom and Treatment Group (Safety Population) 68

Figure 14.3.1.2: Maximum Severity of Solicited Systemic Adverse Events by Symptom and Treatment Group (Safety Population) 69

Figure 14.3.1.3: Maximum Severity of Solicited Local Adverse Events by Study Week and Treatment Group (Safety Population) 70

Figure 14.3.1.4: Maximum Severity of Solicited Systemic Adverse Events by Study Week and Treatment Group (Safety Population) 71

Figure 14.3.1.5: Frequency of Unsolicited Adverse Events by MedDRA System Organ Class, Severity

DocuSign Envelope ID: E3A8D8FC-11A1-44C7-AE49-DBDADC80E86F

and Treatment Group (Safety Population) ... 72
Figure 14.3.1.6: Incidence of Unsolicited Adverse Events by MedDRA System Organ Class, Maximum Severity and Treatment Group (Safety Population) ... 73
Figure 14.3.1.7: Frequency of Unsolicited Adverse Events by MedDRA System Organ Class, Relationship to Treatment and Treatment Group (Safety Population) ... 74
Figure 14.3.1.8: Incidence of Unsolicited Adverse Events by MedDRA System Organ Class, Relationship to Treatment and Treatment Group (Safety Population) ... 75
8. LISTING SHELLS ... 76
Listing 16.2.1.1: Participant Disposition (Screened Participants) ... 77
Listing 16.2.1.2: Early Terminations or Discontinued Participants (Enrolled Participants) ... 80
Listing 16.2.2.1: Participant-Specific Protocol Deviations (Enrolled Participants) ... 81
Listing 16.2.2.2: Non-Participant-Specific Protocol Deviations ... 82
Listing 16.2.2.3: Participants Whose Assigned Treatment Group Does Not Match Their Actual Treatment Received ... 83
Listing 16.2.3: Participants Excluded from Analysis Populations (Enrolled Participants) ... 84
Listing 16.2.4.1: Demographic Data (Enrolled Participants) ... 85
Listing 16.2.4.2: Education and Employment Status (Enrolled Participants) ... 86
Listing 16.2.4.3: Urogenital History (Enrolled Participants) ... 87
Listing 16.2.4.4: Pre-Existing Medical Conditions (Enrolled Participants) ... 88
Listing 16.2.4.5: Other History (Enrolled Participants) ... 89
Listing 16.2.4.6: Smoking History (Enrolled Participants) ... 90
Listing 16.2.4.7: Gynaecological History (Enrolled Participants) ... 91
Listing 16.2.4.8: Pregnancy History (Enrolled Participants) ... 92
Listing 16.2.4.9: Recent Sexual History (Enrolled Participants) ... 93
Listing 16.2.4.10: Baseline Acceptability Questionnaire (Enrolled Participants) ... 94
Listing 16.2.4.11: Prior and Concomitant Medications (Enrolled Participants) ... 95
Listing 16.2.4.12: Birth Control (Enrolled Participants) ... 96
Listing 16.2.5.1: Treatment Compliance Data (Treated Participants) ... 97
Listing 16.2.5.2: Missed Dose Data (Treated Participants) ... 98
Listing 16.2.5.3: Metronidazole Administration (Enrolled Participants) ... 99
Listing 16.2.6.1: Bacterial Vaginosis Diagnosis (ITT Population) ... 100
Listing 16.2.6.2: Clinical Laboratory Results – Vaginal pH (ITT Population) ... 101
Listing 16.2.6.3: External Laboratory Results – Vaginal Gram Stain Results (ITT Population) ... 102
Listing 16.2.7.1: Solicited Local Adverse Events (Safety Population) ... 103
Listing 16.2.7.2: Solicited Systemic Adverse Events (Safety Population) ... 104
Listing 16.2.7.3: Unsolicited Adverse Events (Safety Population) ... 105
Listing 16.2.7.4: Non-Serious, Unsolicited, Moderate or Severe Adverse Events (Safety Population)

DocuSign Envelope ID: E3A8D8FC-11A1-44C7-AE49-DBDADC80E86F

.... 106
Listing 16.2.7.5: Serious Adverse Events (Safety Population).. 107
Listing 16.2.7.6: Medical Review (Safety Population) 108
Listing 16.2.7.7: Pregnancy Notification and Outcome (Safety Population)................................ 109
Listing 16.2.8.1: Clinical Laboratory Results – Urine Pregnancy Test (Safety Population).......... 110
Listing 16.2.8.2: Clinical Laboratory Results – Urinalysis (Safety Population)........................... 111
Listing 16.2.8.3: External Laboratory Results – Urinalysis (Safety Population).......................... 112
Listing 16.2.8.4: External Laboratory Results – Serology and Sexually Transmitted Infection Testing (Safety Population).. 113
Listing 16.2.8.5: Sexually Transmitted Infections (Safety Population).. 114
Listing 16.2.8.6: Vital Signs (Safety Population)... 115
Listing 16.2.8.7: Physical Examination Findings (Safety Population) 116
Listing 16.2.8.8: Speculum Examination Findings (Safety Population)....................................... 117
Listing 16.2.8.9: Abnormal Discharge (Safety Population) 118
Listing 16.2.8.10: Cervical Mucus (Safety Population) 119
Listing 16.2.8.11: External Genital Examination Findings (Safety Population)........................... 120
Listing 16.2.8.12: Gynaecological Review (Safety Population)... 121
Listing 16.2.8.13: Follow-Up Sexual History (Safety Population)... 122
Listing 16.2.8.14: Follow-Up Acceptability Questionnaire (Enrolled Participants)..................... 123

DocuSign Envelope ID: E3A8D8FC-11A1-44C7-AE49-DBDADC80E86F

1. INTRODUCTION

This Statistical Analysis Plan provides more detail for the statistical considerations identified in the LV-007 Protocol (Version 4.3, 03-SEP-2021) and includes table, listing and figure shells.

2. STUDY OBJECTIVES

The primary objectives of this study are:

- Determine the effect of repeat dosing of LACTIN-V (2 x 10^9 cfu/dose) on genital tract inflammation in young South African women at risk of HIV.
- Determine the ability of LACTIN-V to promote a Lactobacillus-dominant vaginal microbiota in young South African women at risk of HIV.
- Determine the safety and acceptability of LACTIN-V in a population of young South African women at risk for HIV.

3. STUDY DESIGN

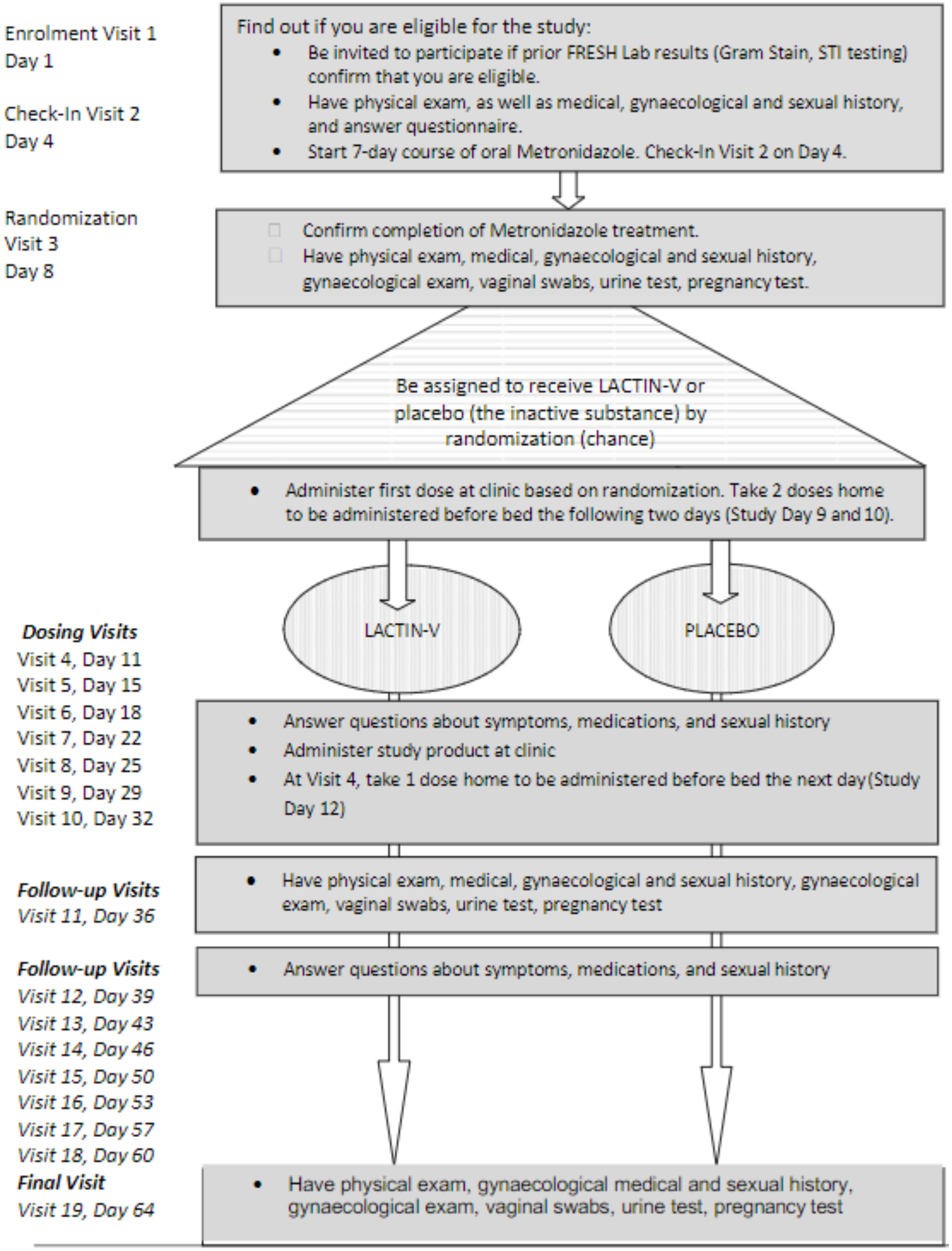

4. ANALYSIS POPULATIONS

Intent-to-Treat (ITT) Population: consists of all randomized participants.

Safety Population: consists of all randomized participants who received at least one dose of study product after randomization.

Modified Intent-to-Treat (mITT) Population: consists of all randomized participants, excluding those participants who were randomized to Placebo but erroneously received LACTIN-V.

Per-Protocol (PP) Population: consists of participants in the mITT Population who received at least 9 correct doses of the 11 assigned doses of study product.

DocuSign Envelope ID: E3A8D8FC-11A1-44C7-AE49-DBDADC80E86F

5. SCHEDULE OF STUDY PROCEDURES

			Study Treatment		Study Follow-Up					
Evaluation		Enrolment Visit 1 Day 1	Randomization Visit 3 (Day 8)	Check-In and Dosing Visits 4 - 10	Follow-Up Visit 11 Day 36	Check-In Visits 12 - 18	Final Study Visit 19 Day 64	Unscheduled Visit	Study Product Discontinuation	Early Termination Visit
Visit window (Study Days)					33- 43		57-71			
Signed consent form		X								
Assessment of eligibility criteria		X	X							
Demographics		X								
Randomization			X							
Detailed medical, gynaecological and sexual history		X								
Dispense Metronidazole		X			(X)		(X)			
Dispense LACTIN-V/placebo and applicators			X	X						
Dispense condoms		X	X	X	X	X		X	X	
Brief medical, gynaecological and sexual history			X		X		X	X	X	X
Review of concomitant medications		X	X		X		X	X	X	X
Study intervention			X	X						
Physical Examination	Complete	X								
	Symptom-directed		X		X		X	X	X	X
	Vital signs	X	X		X		X	X	X	X
	Pelvic examination		X		X		X	X	X	X
Review symptoms, AEs, menses, sexual activity				X	X	X	X	X	X	X
Counsel to abstain from sexual intercourse			X	X	X	X		X		

			Study Treatment		Study Follow-Up					
Evaluation		Enrolment Visit 1 Day 1	Randomization Visit 3 (Day 8)	Check-In and Dosing Visits 4 - 10	Follow-Up Visit 11 Day 36	Check-In Visits 12 - 18	Final Study Visit 19 Day 64	Unscheduled Visit	Study Product Discontinuation	Early Termination Visit
Reminder not to use vaginal products			X	X	X	X		X		
Assessment of adverse events				X	X	X	X	X	X	X
Clinical Labor	Clean catch urine dipstick	X	X		X		X	X	X	X
	Urinalysis	(X)	(X)		(X)		(X)	(X)	(X)	(X)
	Rapid urine βhCG pregnancy test	X	X		X		X	X	X	X
	Vaginal swab for *N. gonorrhoeae, C. trachomatis, T. vaginalis and M. genitalium*		(X)		(X)		(X)	(X)	(X)	(X)
Research Laboratory	Vaginal swab for pH		X		X		X	X	X	X
	PSA test for sperm exposure as POCT		X		X		X	X	X	X
	Vaginal swab for Gram stain		X		X		X	X	X	X
	Vaginal swab for cytokines, HIV Target cells, vaginal bacteria, lactobacilli species		X		X		X	X	X	X
	Vaginal swab for qPCR (*L. crispatus* identification)		X		X		X	X	X	X
	Cervicovaginal Lavage		X		X		X	X	X	X
	Future Use vaginal swabs		X		X		X	X	X	X
	Acceptability questionnaire	X			X				X	X

(X) if indicated

DocuSign Envelope ID: E3A8D8FC-11A1-44C7-AE49-DBDADC80E86F

6. TABLE SHELLS

Table 14.1.1: Summary of Enrollment and Randomization (Screened Participants)

Table 14.1.1: Summary of Enrollment and Randomization
Screened Participants

Category	All Participants (N=XX) n (%)
Participants screened	xx (xx.x%)
Enrollment failure	xx (xx.x%)
Participants enrolled	xx (xx.x%)
Randomization failure	xx (xx.x%)
Participants randomized	xx (xx.x%)

Notes:
n (%) = the number (percentage) of participants in each category.
The denominator for percentages is based on the number of participants screened (N).

DocuSign Envelope ID: E3A8D8FC-11A1-44C7-AE49-DBDADC80E86F

Table 14.1.2: Summary of Enrollment and Randomization Failures (Screened Participants)

Table 14.1.2: Summary of Enrollment and Randomization Failures
(Screened Participants)

Category	Inclusion / Exclusion / Other Criterion	All Participants (N=XX) n (%)
Enrollment Failures[1]	Any eligibility criterion	xx (xx.x%)
	Any inclusion criterion	xx (xx.x%)
	Xxx	xx (xx.x%)
	Xxx	xx (xx.x%)
	Any exclusion criterion	xx (xx.x%)
	Xxx	xx (xx.x%)
	Xxx	xx (xx.x%)
	Any other criterion	xx (xx.x%)
	Xxx	xx (xx.x%)
	Xxx	xx (xx.x%)
Randomization Failures[2]	Any eligibility criterion	xx (xx.x%)
	Any inclusion criterion	xx (xx.x%)
	Xxx	xx (xx.x%)
	Xxx	xx (xx.x%)
	Any exclusion criterion	xx (xx.x%)
	Xxx	xx (xx.x%)
	Xxx	xx (xx.x%)
	Any other criterion	xx (xx.x%)
	Xxx	xx (xx.x%)
	Xxx	xx (xx.x%)

Notes:
N = the number of screened participants.
n (%) = the number (percentage) of participants in each category.
1: The denominator for percentages is based on the number of participants who failed enrollment.
2: The denominator for percentages is based on the number of participants who failed randomization.
More than one criterion can be marked per participant.

Table 14.1.3: Analysis Populations by Treatment Group (Randomized Participants)

Table 14.1.3: Analysis Populations by Treatment Group
(Randomized Participants)

Analysis Population	Eligibility Category	Reason Participant(s) Excluded	LACTIN-V (N=XX) n (%)	Placebo (N=XX) n (%)	All Participants (N=XX) n (%)
ITT Analysis Population	Eligible for ITT		xx (xx.x%)	xx (xx.x%)	xx (xx.x%)
	Excluded from ITT	Any Reason	xx (xx.x%)	xx (xx.x%)	xx (xx.x%)
		Xxxxx	xx (xx.x%)	xx (xx.x%)	xx (xx.x%)
		Xxxxx	xx (xx.x%)	xx (xx.x%)	xx (xx.x%)
		Xxxxx	xx (xx.x%)	xx (xx.x%)	xx (xx.x%)
mITT Analysis Population	Eligible for mITT		xx (xx.x%)	xx (xx.x%)	xx (xx.x%)
	Excluded from mITT	Any Reason	xx (xx.x%)	xx (xx.x%)	xx (xx.x%)
		Xxxxx	xx (xx.x%)	xx (xx.x%)	xx (xx.x%)
		Xxxxx	xx (xx.x%)	xx (xx.x%)	xx (xx.x%)
		Xxxxx	xx (xx.x%)	xx (xx.x%)	xx (xx.x%)
Safety Analysis Population	Eligible for Safety		xx (xx.x%)	xx (xx.x%)	xx (xx.x%)
	Excluded from Safety	Any Reason	xx (xx.x%)	xx (xx.x%)	xx (xx.x%)
		Xxxxx	xx (xx.x%)	xx (xx.x%)	xx (xx.x%)
		Xxxxx	xx (xx.x%)	xx (xx.x%)	xx (xx.x%)
		Xxxxx	xx (xx.x%)	xx (xx.x%)	xx (xx.x%)
PP Analysis Population	Eligible for PP		xx (xx.x%)	xx (xx.x%)	xx (xx.x%)
	Excluded from PP	Any Reason	xx (xx.x%)	xx (xx.x%)	xx (xx.x%)
		Xxxxx	xx (xx.x%)	xx (xx.x%)	xx (xx.x%)
		Xxxxx	xx (xx.x%)	xx (xx.x%)	xx (xx.x%)
		Xxxxx	xx (xx.x%)	xx (xx.x%)	xx (xx.x%)

DocuSign Envelope ID: E3A8D8FC-11A1-44C7-AE49-DBDADC80E86F

Notes:
ITT = Intent-to-Treat. mITT = modified Intent-to-Treat. PP = Intent-to-Treat.
n (%) = the number (percentage) of participants in each category.
The denominator for percentages is based on the number of participants randomized (N).

DocuSign Envelope ID: E3A8D8FC-11A1-44C7-AE49-DBDADC80E86F

Table 14.1.4: Summary of Halting Rules by Treatment Group (Safety Population)

Table 14.1.4: Summary of Halting Rules by Treatment Group
(Safety Population)

Halting Rule	LACTIN-V (N=XX) n (%)	Placebo (N=XX) n (%)	All Participants (N=XX) n (%)
One or more participants experience a treatment related SAE	xx (xx.x%)	xx (xx.x%)	xx (xx.x%)
Two or more participants experience a treatment related vulvar and/or vaginal ulceration, abscess, or necrosis	xx (xx.x%)	xx (xx.x%)	xx (xx.x%)
Two or more participants experience a treatment related severe (Grade 3) or life-threatening (Grade 4) systemic adverse event	xx (xx.x%)	xx (xx.x%)	xx (xx.x%)
An overall pattern of symptomatic, clinical, or laboratory events that the monitor or DSMB consider associated with study product and that may collectively represent a serious potential concern for safety	xx (xx.x%)	xx (xx.x%)	xx (xx.x%)

Notes:
SAE = Serious Adverse Event.
DSMB = Data and Safety and Monitoring Board.
n (%) = the number (percentage) of participants contributing to the halting rule.
The denominator for percentages is based on the number of participants in the Safety population (N).

Table 14.1.5: Participant Disposition by Treatment Group (Enrolled Participants)

Table 14.1.5: Participant Disposition by Treatment Group
(Enrolled Participants)

Category	LACTIN-V (N=XX) n (%)	Placebo (N=XX) n (%)	All Participants (N=XX) n (%)
Participants enrolled			xx (100%)
Randomization failure			xx (xx.x%)
Participants randomized	xx (xx.x%)	xx (xx.x%)	xx (xx.x%)
Did not receive LACTIN-V or Placebo	xx (xx.x%)	xx (xx.x%)	xx (xx.x%)
Received at least one dose of LACTIN-V or Placebo	xx (xx.x%)	xx (xx.x%)	xx (xx.x%)
Complied with treatment	xx (xx.x%)	xx (xx.x%)	xx (xx.x%)
Received at least 9 correct doses of the 11 doses	xx (xx.x%)	xx (xx.x%)	xx (xx.x%)
Received at least 1 dose of LACTIN-V but assigned to Placebo		xx (xx.x%)	xx (xx.x%)
Received at least 1 dose of Placebo but assigned to LACTIN-V	xx (xx.x%)		xx (xx.x%)
Completed Visit 11 (Day 36)	xx (xx.x%)	xx (xx.x%)	xx (xx.x%)
Completed Visit 19 (Day 64)	xx (xx.x%)	xx (xx.x%)	xx (xx.x%)
Completed the study	xx (xx.x%)	xx (xx.x%)	xx (xx.x%)
Early terminated the study	xx (xx.x%)	xx (xx.x%)	xx (xx.x%)
Investigator termination	xx (xx.x%)	xx (xx.x%)	xx (xx.x%)
Voluntary withdrawal	xx (xx.x%)	xx (xx.x%)	xx (xx.x%)
Etc.	xx (xx.x%)	xx (xx.x%)	xx (xx.x%)

Notes:
Complied with treatment = received all 11 assigned doses of LACTIN-V or Placebo.
N (%) = the number (percentage) of participants in each category.
The denominator for percentages is based on the number of participants enrolled (N).

DocuSign Envelope ID: E3A8D8FC-11A1-44C7-AE49-DBDADC80E86F

Table 14.1.6: Summary of Demographics by Treatment Group (ITT Population)

Table 14.1.6: Summary of Demographics by Treatment Group
(ITT Population)

Category	Value / Statistic	LACTIN-V (N=XX)	Placebo (N=XX)	All Participants (N=XX)
Sex (n (%))	Female	xx (xx.x%)	xx (xx.x%)	xx (xx.x%)
Race (n (%))	American Indian or Alaska Native	xx (xx.x%)	xx (xx.x%)	xx (xx.x%)
	Asian	xx (xx.x%)	xx (xx.x%)	xx (xx.x%)
	Native Hawaiian or Other Pacific Islander	xx (xx.x%)	xx (xx.x%)	xx (xx.x%)
	Black or African	xx (xx.x%)	xx (xx.x%)	xx (xx.x%)
	White	xx (xx.x%)	xx (xx.x%)	xx (xx.x%)
	Multi-Racial	xx (xx.x%)	xx (xx.x%)	xx (xx.x%)
	Unknown	xx (xx.x%)	xx (xx.x%)	xx (xx.x%)
Age (years)	n	xx	xx	xx
	Mean	xx.x	xx.x	xx.x
	Standard Deviation	xx.xx	xx.xx	xx.xx
	Median	xx.x	xx.x	xx.x
	Minimum	xx	xx	xx
	Maximum	xx	xx	xx

Notes:
ITT = Intent-to-Treat.
N (%) = the number (percentage) of participants in each category.
N = the number of participants with data available.
The denominator for percentages is based on the number of participants in the ITT population (N) with data available.

Table 14.1.7: Summary of Education and Employment Status by Treatment Group (ITT Population)

Table 14.1.7: Summary of Education and Employment Status by Treatment Group
(ITT Population)

Category	Value / Statistic	LACTIN-V (N=XX)	Placebo (N=XX)	All Participants (N=XX)
Highest grade / level of school completed (n (%))	Grade 10	xx (xx.x%)	xx (xx.x%)	xx (xx.x%)
	Grade 11	xx (xx.x%)	xx (xx.x%)	xx (xx.x%)
	Grade 12	xx (xx.x%)	xx (xx.x%)	xx (xx.x%)
Completed high school (matric) (n (%))	Yes	xx (xx.x%)	xx (xx.x%)	xx (xx.x%)
	No	xx (xx.x%)	xx (xx.x%)	xx (xx.x%)
Tertiary education (n (%))	Yes	xx (xx.x%)	xx (xx.x%)	xx (xx.x%)
	No	xx (xx.x%)	xx (xx.x%)	xx (xx.x%)
Tertiary education (years completed)	n	xx	xx	xx
	Mean	xx.x	xx.x	xx.x
	Standard Deviation	xx.xx	xx.xx	xx.xx
	Median	xx.x	xx.x	xx.x
	Minimum	xx	xx	xx
	Maximum	xx	xx	xx
Employed (n (%))	Yes	xx (xx.x%)	xx (xx.x%)	xx (xx.x%)
	No	xx (xx.x%)	xx (xx.x%)	xx (xx.x%)
Employed (Part-time / Full-time) (n (%))	Part-time	xx (xx.x%)	xx (xx.x%)	xx (xx.x%)
	Full-time	xx (xx.x%)	xx (xx.x%)	xx (xx.x%)
Currently in school (n (%))	Yes	xx (xx.x%)	xx (xx.x%)	xx (xx.x%)
	No	xx (xx.x%)	xx (xx.x%)	xx (xx.x%)
Currently in school (Part-time / Full-time) (n (%))	Part-time	xx (xx.x%)	xx (xx.x%)	xx (xx.x%)
	Full-time	xx (xx.x%)	xx (xx.x%)	xx (xx.x%)

Notes:
ITT = Intent-to-Treat.
N (%) = the number (percentage) of participants in each category.
N = the number of participants with data available.
The denominator for percentages is based on the number of participants in the ITT population (N) with data available.

DocuSign Envelope ID: E3A8D8FC-11A1-44C7-AE49-DBDADC80E86F

Table 14.1.8: Protocol Deviations by Treatment Group (ITT Population)

Table 14.1.8: Protocol Deviations by Treatment Group
(ITT Population)

**Category**	**LACTIN-V (N=XX) n (%) E**	**Placebo (N=XX) n (%) E**	**All Participants (N=XX) n (%) E**
Any deviation	xx (xx.x%) xx	xx (xx.x%) xx	xx (xx.x%) xx
Eligibility criterion	xx (xx.x%) xx	xx (xx.x%) xx	xx (xx.x%) xx
Enrollment procedures	xx (xx.x%) xx	xx (xx.x%) xx	xx (xx.x%) xx
Informed consent	xx (xx.x%) xx	xx (xx.x%) xx	xx (xx.x%) xx
Out of visit window	xx (xx.x%) xx	xx (xx.x%) xx	xx (xx.x%) xx
Procedure done incorrectly	xx (xx.x%) xx	xx (xx.x%) xx	xx (xx.x%) xx
Procedure not conducted	xx (xx.x%) xx	xx (xx.x%) xx	xx (xx.x%) xx
Prohibited / restricted vaginal products	xx (xx.x%) xx	xx (xx.x%) xx	xx (xx.x%) xx
Specimen not obtained	xx (xx.x%) xx	xx (xx.x%) xx	xx (xx.x%) xx
Specimen result not obtained	xx (xx.x%) xx	xx (xx.x%) xx	xx (xx.x%) xx
Study product adherence	xx (xx.x%) xx	xx (xx.x%) xx	xx (xx.x%) xx
Study product administration error	xx (xx.x%) xx	xx (xx.x%) xx	xx (xx.x%) xx
Received at least 1 dose of LACTIN-V but assigned to Placebo		xx (xx.x%) xx	xx (xx.x%) xx
Received at least 1 dose of Placebo but assigned to LACTIN-V	xx (xx.x%) xx		xx (xx.x%) xx
Study product unblinded	xx (xx.x%) xx	xx (xx.x%) xx	xx (xx.x%) xx
Other	xx (xx.x%) xx	xx (xx.x%) xx	xx (xx.x%) xx

Notes:
ITT = Intent-to-Treat.
N (%) = the number (percentage) of participants in each category.
E = the number of deviations in each category.
The denominator for percentages is based on the number of participants in the ITT population (N).

Table 14.1.9: Summary of Baseline Gynaecological History by Treatment Group (ITT Population)

Table 14.1.9: Summary of Baseline Gynaecological History by Treatment Group
(ITT Population)

Interview Question	Value / Statistic	LACTIN-V (N=XX)	Placebo (N=XX)	All Participants (N=XX)
Participant had hysterectomy (n (%))	Yes	xx (xx.x%)	xx (xx.x%)	xx (xx.x%)
Have you been on long-acting hormonal contraception (depo or noristerate injection, implanon) for at least 3 months? (n (%))	Yes	xx (xx.x%)	xx (xx.x%)	xx (xx.x%)
	No	xx (xx.x%)	xx (xx.x%)	xx (xx.x%)
Have you had any menstrual bleeding in the past 3 months (n (%))	Yes	xx (xx.x%)	xx (xx.x%)	xx (xx.x%)
	No	xx (xx.x%)	xx (xx.x%)	xx (xx.x%)
How many days does your period last?	N	xx	xx	xx
	Mean (Standard Deviation)	xx.x (xx.xx)	xx.x (xx.xx)	xx.x (xx.xx)
	Median	xx.x	xx.x	xx.x
	Minimum, Maximum	xx, xx	xx, xx	xx, xx
How many days is your average menstrual cycle?	N	xx	xx	xx
	Mean (Standard Deviation)	xx.x (xx.xx)	xx.x (xx.xx)	xx.x (xx.xx)
	Median	xx.x	xx.x	xx.x
	Minimum, Maximum	xx, xx	xx, xx	xx, xx
In the past 3 months, have you had any abnormal menstrual cycles? (n (%))	Yes	xx (xx.x%)	xx (xx.x%)	xx (xx.x%)
	No	xx (xx.x%)	xx (xx.x%)	xx (xx.x%)
Cycle length less than 21 days (n (%))	Yes	xx (xx.x%)	xx (xx.x%)	xx (xx.x%)
	No	xx (xx.x%)	xx (xx.x%)	xx (xx.x%)
Cycle length more than 35 days (n (%))	Yes	xx (xx.x%)	xx (xx.x%)	xx (xx.x%)
	No	xx (xx.x%)	xx (xx.x%)	xx (xx.x%)

DocuSign Envelope ID: E3A8D8FC-11A1-44C7-AE49-DBDADC80E86F

Intermenstrual bleeding or spotting (n (%))	Yes	xx (xx.x%)	xx (xx.x%)	xx (xx.x%)
	No	xx (xx.x%)	xx (xx.x%)	xx (xx.x%)
What sanitary products do you use during your period? (n (%))	Pads	xx (xx.x%)	xx (xx.x%)	xx (xx.x%)
	Cups	xx (xx.x%)	xx (xx.x%)	xx (xx.x%)
	Tampons	xx (xx.x%)	xx (xx.x%)	xx (xx.x%)
	Other	xx (xx.x%)	xx (xx.x%)	xx (xx.x%)
Have you ever douched or used vaginal preparations, drying agents, sexual stimulants, or other vaginal products? (n (%))	Yes	xx (xx.x%)	xx (xx.x%)	xx (xx.x%)
	No	xx (xx.x%)	xx (xx.x%)	xx (xx.x%)
In the last 30 days, how many times have you douched, used vaginal preparations, drying agents, sexual stimulants, or other vaginal products?	N	xx	xx	xx
	Mean (Standard Deviation)	xx.x (xx.xx)	xx.x (xx.xx)	xx.x (xx.xx)
	Median	xx.x	xx.x	xx.x
	Minimum, Maximum	xx, xx	xx, xx	xx, xx
PREGNANCY HISTORY:				
How many times have you been pregnant?	N	xx	xx	xx
	Mean (Standard Deviation)	xx.x (xx.xx)	xx.x (xx.xx)	xx.x (xx.xx)
	Median	xx.x	xx.x	xx.x
	Minimum, Maximum	xx, xx	xx, xx	xx, xx
How many pregnancies resulted in a live birth?	N	xx	xx	xx
	Mean (Standard Deviation)	xx.x (xx.xx)	xx.x (xx.xx)	xx.x (xx.xx)
	Median	xx.x	xx.x	xx.x
	Minimum, Maximum	xx, xx	xx, xx	xx, xx
How many pregnancies resulted in a stillborn birth?	N	xx	xx	xx
	Mean (Standard Deviation)	xx.x (xx.xx)	xx.x (xx.xx)	xx.x (xx.xx)
	Median	xx.x	xx.x	xx.x
	Minimum, Maximum	xx, xx	xx, xx	xx, xx

How many pregnancies resulted in a spontaneous abortion?	N	xx	xx	xx
	Mean (Standard Deviation)	xx.x (xx.xx)	xx.x (xx.xx)	xx.x (xx.xx)
	Median	xx.x	xx.x	xx.x
	Minimum, Maximum	xx, xx	xx, xx	xx, xx
How many pregnancies resulted in a termination of pregnancy?	N	xx	xx	xx
	Mean (Standard Deviation)	xx.x (xx.xx)	xx.x (xx.xx)	xx.x (xx.xx)
	Median	xx.x	xx.x	xx.x
	Minimum, Maximum	xx, xx	xx, xx	xx, xx
How many pregnancies resulted in an ectopic pregnancy?	N	xx	xx	xx
	Mean (Standard Deviation)	xx.x (xx.xx)	xx.x (xx.xx)	xx.x (xx.xx)
	Median	xx.x	xx.x	xx.x
	Minimum, Maximum	xx, xx	xx, xx	xx, xx
Do you have any children born with birth defects? (n (%))	Yes	xx (xx.x%)	xx (xx.x%)	xx (xx.x%)
	No	xx (xx.x%)	xx (xx.x%)	xx (xx.x%)
RELATIONSHIP STATUS				
What is your current relationship status? (n (%))	Married	xx (xx.x%)	xx (xx.x%)	xx (xx.x%)
	Divorced, separated	xx (xx.x%)	xx (xx.x%)	xx (xx.x%)
	Single (no current partner)	xx (xx.x%)	xx (xx.x%)	xx (xx.x%)
	Widowed	xx (xx.x%)	xx (xx.x%)	xx (xx.x%)
	Steady partner, cohabitating	xx (xx.x%)	xx (xx.x%)	xx (xx.x%)
	Steady partner, not cohabitating	xx (xx.x%)	xx (xx.x%)	xx (xx.x%)
	Casual partner	xx (xx.x%)	xx (xx.x%)	xx (xx.x%)
RECENT SEXUAL HISTORY				
How old were you when you first had sexual intercourse?	N	xx	xx	xx
	Mean (Standard Deviation)	xx.x (xx.xx)	xx.x (xx.xx)	xx.x (xx.xx)
	Median	xx.x	xx.x	xx.x
	Minimum, Maximum	xx, xx	xx, xx	xx, xx

DocuSign Envelope ID: E3A8D8FC-11A1-44C7-AE49-DBDADC80E86F

How many sexual partners have you had in your life?	N	xx	xx	xx
	Mean (Standard Deviation)	xx.x (xx.xx)	xx.x (xx.xx)	xx.x (xx.xx)
	Median	xx.x	xx.x	xx.x
	Minimum, Maximum	xx, xx	xx, xx	xx, xx
Have you ever had anal sex? (n (%))	Yes	xx (xx.x%)	xx (xx.x%)	xx (xx.x%)
	No	xx (xx.x%)	xx (xx.x%)	xx (xx.x%)
How many male sexual partners have you had in the past 6 months?	N	xx	xx	xx
	Mean (Standard Deviation)	xx.x (xx.xx)	xx.x (xx.xx)	xx.x (xx.xx)
	Median	xx.x	xx.x	xx.x
	Minimum, Maximum	xx, xx	xx, xx	xx, xx
How many female sexual partners have you had in the past 6 months?	N	xx	xx	xx
	Mean (Standard Deviation)	xx.x (xx.xx)	xx.x (xx.xx)	xx.x (xx.xx)
	Median	xx.x	xx.x	xx.x
	Minimum, Maximum	xx, xx	xx, xx	xx, xx
In the past 30 days, did you have vaginal sex? (n (%))	Yes	xx (xx.x%)	xx (xx.x%)	xx (xx.x%)
	No	xx (xx.x%)	xx (xx.x%)	xx (xx.x%)
How many times did you have vaginal sex (in the last 30 days)?	N	xx	xx	xx
	Mean (Standard Deviation)	xx.x (xx.xx)	xx.x (xx.xx)	xx.x (xx.xx)
	Median	xx.x	xx.x	xx.x
	Minimum, Maximum	xx, xx	xx, xx	xx, xx
When you had vaginal sex, how many times did you use a condom?	N	xx	xx	xx
	Mean (Standard Deviation)	xx.x (xx.xx)	xx.x (xx.xx)	xx.x (xx.xx)
	Median	xx.x	xx.x	xx.x
	Minimum, Maximum	xx, xx	xx, xx	xx, xx

How many days since you had vaginal sex?	N	xx	xx	xx
	Mean (Standard Deviation)	xx.x (xx.xx)	xx.x (xx.xx)	xx.x (xx.xx)
	Median	xx.x	xx.x	xx.x
	Minimum, Maximum	xx, xx	xx, xx	xx, xx

Notes:
Baseline is defined as Visit 1 Day 1.
ITT = Intent-to-Treat.
N (%) = the number (percentage) of participants in each category.
N = the number of participants with data available.
The denominator for percentages is based on the number of participants in the ITT population (N) with data available.

Table 14.1.10: Summary of Long-Acting Reversible Contraception by Treatment Group (ITT Population)

Table 14.1.10: Summary of Prior Use of Long-Acting Reversible Contraception by Treatment Group
(ITT Population)

**Category**	**LACTIN-V (N=XX) n (%)**	**Placebo (N=XX) n (%)**	**All Participants (N=XX) n (%)**
1-3 Months of LARC Prior to Enrollment	xx (xx.x%)	xx (xx.x%)	xx (xx.x%)
>3 Months of LARC Prior to Enrollment	xx (xx.x%)	xx (xx.x%)	xx (xx.x%)
Birth Control Other Than LARC Prior to Enrollment	xx (xx.x%)	xx (xx.x%)	xx (xx.x%)

Notes:
ITT = Intent-to-Treat.
LARC = Long-Acting Reversible Contraception.
N (%) = the number (percentage) of participants in each category.
The denominator for percentages is based on the number of participants in the ITT population (N).

DocuSign Envelope ID: E3A8D8FC-11A1-44C7-AE49-DBDADC80E86F

Table 14.1.11: Treatment Compliance by Treatment Group and Analysis Population

Table 14.1.11: Treatment Compliance by Treatment Group and Analysis Population

Analysis Population	Treatment Group	Number of Participants Administered 11 Doses	Number of Participants Administered 11 Correct Doses	Number of Participants Compliant with Dose Regimen n	Number of Participants N	Proportion (95% CI[1]) of Participants Compliant with Dose Regimen	Difference in Proportion (95% CI[2]) of Participants Compliant with Dose Regimen between LACTIN-V Group and Placebo Group	p-value[3]
ITT	LACTIN-V	xx	xx	xx	xx	0.xxx (0.xxx-0.xxx)		0.xxx
	Placebo	xx	xx	xx	xx	0.xxx (0.xxx-0.xxx)		0.xxx
	All Participants	xx	xx	xx	xx	0.xxx (0.xxx-0.xxx)	0.xxx (0.xxx-0.xxx)	0.xxx
mITT	LACTIN-V	xx	xx	xx	xx	0.xxx (0.xxx-0.xxx)		0.xxx
	Placebo	xx	xx	xx	xx	0.xxx (0.xxx-0.xxx)		0.xxx
	All Participants	xx	xx	xx	xx	0.xxx (0.xxx-0.xxx)	0.xxx (0.xxx-0.xxx)	0.xxx
Safety	LACTIN-V	xx	xx	xx	xx	0.xxx (0.xxx-0.xxx)		0.xxx
	Placebo	xx	xx	xx	xx	0.xxx (0.xxx-0.xxx)		0.xxx
	All Participants	xx	xx	xx	xx	0.xxx (0.xxx-0.xxx)	0.xxx (0.xxx-0.xxx)	0.xxx
PP	LACTIN-V	xx	xx	xx	xx	0.xxx (0.xxx-0.xxx)		0.xxx
	Placebo	xx	xx	xx	xx	0.xxx (0.xxx-0.xxx)		0.xxx
	All Participants	xx	xx	xx	xx	0.xxx (0.xxx-0.xxx)	0.xxx (0.xxx-0.xxx)	0.xxx

Notes:
ITT = Intent-to-Treat.
PP = Per-Protocol.
CI = Confidence Interval.
Dose regimen compliance is defined as participants who received at least 9 correct doses out of 11 doses.
The denominator for proportions is based on the number of participants in the treatment group and analysis population (N).
1: 95%CI = 95% Wilson CI.
2: Difference in 95% CI = Asymptotic 95% CI from a Chi-Square test.
3: p-value calculated from the Chi-Square Test.

Table 14.1.12: Product Administration by Week and Dose Number by Treatment Group (Safety Population)

Table 14.1.12: Product Administration by Week and Dose Number by Treatment Group
(Safety Population)

	LACTIN-V (N=XX)				Placebo (N=XX)			
Week<br>Dose	Correct Dose Taken<br>n (%)	Incorrect Dose Taken<br>n (%)	Any Dose Taken<br>n (%)	Dose Not Taken<br>n (%)	Correct Dose Taken<br>n (%)	Incorrect Dose Taken<br>n (%)	Any Dose Taken<br>n (%)	Dose Not Taken<br>n (%)
Week 1								
1	xx (xx.x%)	xx (xx.x%)	xx (xx.x%)	xx (xx.x%)	xx (xx.x%)	xx (xx.x%)	xx (xx.x%)	xx (xx.x%)
2	xx (xx.x%)	xx (xx.x%)	xx (xx.x%)	xx (xx.x%)	xx (xx.x%)	xx (xx.x%)	xx (xx.x%)	xx (xx.x%)
3	xx (xx.x%)	xx (xx.x%)	xx (xx.x%)	xx (xx.x%)	xx (xx.x%)	xx (xx.x%)	xx (xx.x%)	xx (xx.x%)
4	xx (xx.x%)	xx (xx.x%)	xx (xx.x%)	xx (xx.x%)	xx (xx.x%)	xx (xx.x%)	xx (xx.x%)	xx (xx.x%)
5	xx (xx.x%)	xx (xx.x%)	xx (xx.x%)	xx (xx.x%)	xx (xx.x%)	xx (xx.x%)	xx (xx.x%)	xx (xx.x%)
Week 2								
1	xx (xx.x%)	xx (xx.x%)	xx (xx.x%)	xx (xx.x%)	xx (xx.x%)	xx (xx.x%)	xx (xx.x%)	xx (xx.x%)
2	xx (xx.x%)	xx (xx.x%)	xx (xx.x%)	xx (xx.x%)	xx (xx.x%)	xx (xx.x%)	xx (xx.x%)	xx (xx.x%)
Week 3								
1	xx (xx.x%)	xx (xx.x%)	xx (xx.x%)	xx (xx.x%)	xx (xx.x%)	xx (xx.x%)	xx (xx.x%)	xx (xx.x%)
2	xx (xx.x%)	xx (xx.x%)	xx (xx.x%)	xx (xx.x%)	xx (xx.x%)	xx (xx.x%)	xx (xx.x%)	xx (xx.x%)
Week 4								
1	xx (xx.x%)	xx (xx.x%)	xx (xx.x%)	xx (xx.x%)	xx (xx.x%)	xx (xx.x%)	xx (xx.x%)	xx (xx.x%)
2	xx (xx.x%)	xx (xx.x%)	xx (xx.x%)	xx (xx.x%)	xx (xx.x%)	xx (xx.x%)	xx (xx.x%)	xx (xx.x%)

Notes:
The denominator for percentages is based on the number of participants in the Safety population (N).

DocuSign Envelope ID: E3A8D8FC-11A1-44C7-AE49-DBDADC80E86F

Table 14.1.12: Product Administration by Week and Dose Number by Treatment Group
(Safety Population)

	All Participants (N=XX)			
**Week**<br>**Dose**	**Correct Dose Taken n (%)**	**Incorrect Dose Taken n (%)**	**Any Dose Taken n (%)**	**Dose Not Taken n (%)**
Week 1				
1	xx (xx.x%)	xx (xx.x%)	xx (xx.x%)	xx (xx.x%)
2	xx (xx.x%)	xx (xx.x%)	xx (xx.x%)	xx (xx.x%)
3	xx (xx.x%)	xx (xx.x%)	xx (xx.x%)	xx (xx.x%)
4	xx (xx.x%)	xx (xx.x%)	xx (xx.x%)	xx (xx.x%)
5	xx (xx.x%)	xx (xx.x%)	xx (xx.x%)	xx (xx.x%)
Week 2				
1	xx (xx.x%)	xx (xx.x%)	xx (xx.x%)	xx (xx.x%)
2	xx (xx.x%)	xx (xx.x%)	xx (xx.x%)	xx (xx.x%)
Week 3				
1	xx (xx.x%)	xx (xx.x%)	xx (xx.x%)	xx (xx.x%)
2	xx (xx.x%)	xx (xx.x%)	xx (xx.x%)	xx (xx.x%)
Week 4				
1	xx (xx.x%)	xx (xx.x%)	xx (xx.x%)	xx (xx.x%)
2	xx (xx.x%)	xx (xx.x%)	xx (xx.x%)	xx (xx.x%)

Notes:
The denominator for percentages is based on the number of participants in the Safety population (N).

DocuSign Envelope ID: E3A8D8FC-11A1-44C7-AE49-DBDADC80E86F

Table 14.2.1: Bacterial Vaginosis Diagnosis by Visit 11 (Day 36) by Analysis Population and Treatment Group

Table 14.2.1: Bacterial Vaginosis Diagnosis by Visit 11 (Day 36) by Analysis Population and Treatment Group

Analysis Population	Treatment Group	Number of Participants with BV Diagnosis n	Number of Participants N	Proportion of Participants Compliant with BV Diagnosis n/N	BV Diagnosis 95% CI[1]
ITT	LACTIN-V	xx	xx	0.xxx	0.xxx-0.xxx
	Placebo	xx	xx	0.xxx	0.xxx-0.xxx
	All Participants	xx	xx	0.xxx	0.xxx-0.xxx
mITT	LACTIN-V	xx	xx	0.xxx	0.xxx-0.xxx
	Placebo	xx	xx	0.xxx	0.xxx-0.xxx
	All Participants	xx	xx	0.xxx	0.xxx-0.xxx
PP	LACTIN-V	xx	xx	0.xxx	0.xxx-0.xxx
	Placebo	xx	xx	0.xxx	0.xxx-0.xxx
	All Participants	xx	xx	0.xxx	0.xxx-0.xxx

Notes:
ITT = Intent-to-Treat.
PP = Per-Protocol.
BV = Bacterial Vaginosis.
CI = Confidence Interval.
The denominator for proportions is based on the number of participants in the treatment group and analysis population (N).
1: 95%CI = 95% Wilson CI.

DocuSign Envelope ID: E3A8D8FC-11A1-44C7-AE49-DBDADC80E86F

Table 14.2.2: Bacterial Vaginosis Diagnosis by Visit 19 (Day 64) by Analysis Population and Treatment Group

<Table 14.2.2 has the same shell as Table 14.2.1, but with Visit 11 (Day 36) replaced with Visit 19 (Day 64)>

DocuSign Envelope ID: E3A8D8FC-11A1-44C7-AE49-DBDADC80E86F

Table 14.3.1.1: Number and Percentage of Participants Experiencing Solicited Adverse Events with 95% Confidence Intervals by Symptom and Treatment Group (Safety Population)

Table 14.3.1.1: Number and Percentage of Participants Experiencing Solicited Adverse Events with 95% Confidence Intervals by Symptom and Treatment Group
(Safety Population)

Category	Solicited Adverse Event	LACTIN-V (N=XX) n (%) [95% CI]	Placebo (N=XX) n (%) [95% CI]	All Participants (N=XX) n (%) [95% CI]
Solicited Adverse Events	Any Solicited Adverse Event	xx (xx.x%) [xx.x-xx.x]	xx (xx.x%) [xx.x-xx.x]	xx (xx.x%) [xx.x-xx.x]
Solicited Local Adverse Events	Any Local Adverse Event	xx (xx.x%) [xx.x-xx.x]	xx (xx.x%) [xx.x-xx.x]	xx (xx.x%) [xx.x-xx.x]
	Vaginal Bleeding Other Than Menstruation	xx (xx.x%) [xx.x-xx.x]	xx (xx.x%) [xx.x-xx.x]	xx (xx.x%) [xx.x-xx.x]
	Abnormal Vaginal Discharge	xx (xx.x%) [xx.x-xx.x]	xx (xx.x%) [xx.x-xx.x]	xx (xx.x%) [xx.x-xx.x]
	Abnormal Vaginal Odor	xx (xx.x%) [xx.x-xx.x]	xx (xx.x%) [xx.x-xx.x]	xx (xx.x%) [xx.x-xx.x]
	Genital Itching or Burning	xx (xx.x%) [xx.x-xx.x]	xx (xx.x%) [xx.x-xx.x]	xx (xx.x%) [xx.x-xx.x]
	External Genital Irritation	xx (xx.x%) [xx.x-xx.x]	xx (xx.x%) [xx.x-xx.x]	xx (xx.x%) [xx.x-xx.x]
	External Genital Swelling	xx (xx.x%) [xx.x-xx.x]	xx (xx.x%) [xx.x-xx.x]	xx (xx.x%) [xx.x-xx.x]
	Genital Rash	xx (xx.x%) [xx.x-xx.x]	xx (xx.x%) [xx.x-xx.x]	xx (xx.x%) [xx.x-xx.x]
Solicited Systemic Adverse Events	Any Systemic Adverse Event	xx (xx.x%) [xx.x-xx.x]	xx (xx.x%) [xx.x-xx.x]	xx (xx.x%) [xx.x-xx.x]
	Nausea	xx (xx.x%) [xx.x-xx.x]	xx (xx.x%) [xx.x-xx.x]	xx (xx.x%) [xx.x-xx.x]
	Vomiting	xx (xx.x%) [xx.x-xx.x]	xx (xx.x%) [xx.x-xx.x]	xx (xx.x%) [xx.x-xx.x]
	Abdominal Pain/Cramps	xx (xx.x%) [xx.x-xx.x]	xx (xx.x%) [xx.x-xx.x]	xx (xx.x%) [xx.x-xx.x]
	Diarrhea	xx (xx.x%) [xx.x-xx.x]	xx (xx.x%) [xx.x-xx.x]	xx (xx.x%) [xx.x-xx.x]
	Constipation	xx (xx.x%) [xx.x-xx.x]	xx (xx.x%) [xx.x-xx.x]	xx (xx.x%) [xx.x-xx.x]
	Pain/Burning with Urination	xx (xx.x%) [xx.x-xx.x]	xx (xx.x%) [xx.x-xx.x]	xx (xx.x%) [xx.x-xx.x]
	Frequent Urination	xx (xx.x%) [xx.x-xx.x]	xx (xx.x%) [xx.x-xx.x]	xx (xx.x%) [xx.x-xx.x]
	Blood in Urine	xx (xx.x%) [xx.x-xx.x]	xx (xx.x%) [xx.x-xx.x]	xx (xx.x%) [xx.x-xx.x]
	Headache	xx (xx.x%) [xx.x-xx.x]	xx (xx.x%) [xx.x-xx.x]	xx (xx.x%) [xx.x-xx.x]

Notes:
CI = Confidence Interval.
n (%) = the number (percentage) of participants in each solicited adverse event.
The denominator for percentages is based on the number of participants in the Safety population (N).
A participant is only counted once within each solicited adverse event.
95% CI = 95% Blaker Confidence Interval.

DocuSign Envelope ID: E3A8D8FC-11A1-44C7-AE49-DBDADC80E86F

Table 14.3.1.2.1: Number and Percentage of Participants Experiencing Solicited Local Adverse Events with 95% Confidence Intervals by Symptom, Maximum Severity and Treatment Group (Safety Population)

Table 14.3.1.2.1: Number and Percentage of Participants Experiencing Solicited Local Adverse Events with 95% Confidence Intervals by Symptom, Maximum Severity and Treatment Group
(Safety Population)

Solicited Adverse Event	Severity[1]	LACTIN-V (N=XX) n (%) [95% CI]	Placebo (N=XX) n (%) [95% CI]	All Participants (N=XX) n (%) [95% CI]
Any Solicited Local Adverse Event	None	xx (xx.x%) [xx.x-xx.x]	xx (xx.x%) [xx.x-xx.x]	xx (xx.x%) [xx.x-xx.x]
	Mild	xx (xx.x%) [xx.x-xx.x]	xx (xx.x%) [xx.x-xx.x]	xx (xx.x%) [xx.x-xx.x]
	Moderate	xx (xx.x%) [xx.x-xx.x]	xx (xx.x%) [xx.x-xx.x]	xx (xx.x%) [xx.x-xx.x]
	Severe	xx (xx.x%) [xx.x-xx.x]	xx (xx.x%) [xx.x-xx.x]	xx (xx.x%) [xx.x-xx.x]
Vaginal Bleeding Other Than Menstruation	None	xx (xx.x%) [xx.x-xx.x]	xx (xx.x%) [xx.x-xx.x]	xx (xx.x%) [xx.x-xx.x]
	Mild	xx (xx.x%) [xx.x-xx.x]	xx (xx.x%) [xx.x-xx.x]	xx (xx.x%) [xx.x-xx.x]
	Moderate	xx (xx.x%) [xx.x-xx.x]	xx (xx.x%) [xx.x-xx.x]	xx (xx.x%) [xx.x-xx.x]
	Severe	xx (xx.x%) [xx.x-xx.x]	xx (xx.x%) [xx.x-xx.x]	xx (xx.x%) [xx.x-xx.x]
Abnormal Vaginal Discharge	None	xx (xx.x%) [xx.x-xx.x]	xx (xx.x%) [xx.x-xx.x]	xx (xx.x%) [xx.x-xx.x]
	Mild	xx (xx.x%) [xx.x-xx.x]	xx (xx.x%) [xx.x-xx.x]	xx (xx.x%) [xx.x-xx.x]
	Moderate	xx (xx.x%) [xx.x-xx.x]	xx (xx.x%) [xx.x-xx.x]	xx (xx.x%) [xx.x-xx.x]
	Severe	xx (xx.x%) [xx.x-xx.x]	xx (xx.x%) [xx.x-xx.x]	xx (xx.x%) [xx.x-xx.x]
Etc.				

Notes:
CI = Confidence Interval.
n (%) = the number (percentage) of participants in each solicited adverse event and severity.
The denominator for percentages is based on the number of participants in the Safety population (N) with solicited adverse event data available after the first dose of study product.
A participant is only counted once within each solicited adverse event and severity.
95% CI = 95% Blaker Confidence Interval.
1: Each participant's maximum severity is reported for each solicited adverse event across all doses.

DocuSign Envelope ID: E3A8D8FC-11A1-44C7-AE49-DBDADC80E86F

Table 14.3.1.2.2: Number and Percentage of Participants Experiencing Solicited Local Adverse Events of Vaginal Bleeding Other Than Menstruation or Vaginal Discharge with 95% Confidence Intervals by Symptom, Maximum Severity and Treatment Group (Safety Population)

Table 14.3.1.2.2: Number and Percentage of Participants Experiencing Solicited Local Adverse Events of Vaginal Bleeding Other Than Menstruation or Vaginal Discharge with 95% Confidence Intervals by Symptom, Maximum Severity and Treatment Group
(Safety Population)

Solicited Adverse Event	Severity[1]	LACTIN-V (N=XX) n (%) [95% CI]	Placebo (N=XX) n (%) [95% CI]	All Participants (N=XX) n (%) [95% CI]
Vaginal Bleeding Other Than Menstruation				
ALL PARTICIPANTS	None	xx (xx.x%) [xx.x-xx.x]	xx (xx.x%) [xx.x-xx.x]	xx (xx.x%) [xx.x-xx.x]
	Mild	xx (xx.x%) [xx.x-xx.x]	xx (xx.x%) [xx.x-xx.x]	xx (xx.x%) [xx.x-xx.x]
	Moderate	xx (xx.x%) [xx.x-xx.x]	xx (xx.x%) [xx.x-xx.x]	xx (xx.x%) [xx.x-xx.x]
	Severe	xx (xx.x%) [xx.x-xx.x]	xx (xx.x%) [xx.x-xx.x]	xx (xx.x%) [xx.x-xx.x]
PARTICIPANTS ON BC OTHER THAN LARC	None	xx (xx.x%) [xx.x-xx.x]	xx (xx.x%) [xx.x-xx.x]	xx (xx.x%) [xx.x-xx.x]
	Mild	xx (xx.x%) [xx.x-xx.x]	xx (xx.x%) [xx.x-xx.x]	xx (xx.x%) [xx.x-xx.x]
	Moderate	xx (xx.x%) [xx.x-xx.x]	xx (xx.x%) [xx.x-xx.x]	xx (xx.x%) [xx.x-xx.x]
	Severe	xx (xx.x%) [xx.x-xx.x]	xx (xx.x%) [xx.x-xx.x]	xx (xx.x%) [xx.x-xx.x]
PARTICPANTS ON LARC 1-3 MONTHS	None	xx (xx.x%) [xx.x-xx.x]	xx (xx.x%) [xx.x-xx.x]	xx (xx.x%) [xx.x-xx.x]
	Mild	xx (xx.x%) [xx.x-xx.x]	xx (xx.x%) [xx.x-xx.x]	xx (xx.x%) [xx.x-xx.x]
	Moderate	xx (xx.x%) [xx.x-xx.x]	xx (xx.x%) [xx.x-xx.x]	xx (xx.x%) [xx.x-xx.x]
	Severe	xx (xx.x%) [xx.x-xx.x]	xx (xx.x%) [xx.x-xx.x]	xx (xx.x%) [xx.x-xx.x]
PARTICPANTS ON LARC >3 MONTHS	None	xx (xx.x%) [xx.x-xx.x]	xx (xx.x%) [xx.x-xx.x]	xx (xx.x%) [xx.x-xx.x]
	Mild	xx (xx.x%) [xx.x-xx.x]	xx (xx.x%) [xx.x-xx.x]	xx (xx.x%) [xx.x-xx.x]
	Moderate	xx (xx.x%) [xx.x-xx.x]	xx (xx.x%) [xx.x-xx.x]	xx (xx.x%) [xx.x-xx.x]
	Severe	xx (xx.x%) [xx.x-xx.x]	xx (xx.x%) [xx.x-xx.x]	xx (xx.x%) [xx.x-xx.x]
<Repeat for Vaginal Discharge>				

DocuSign Envelope ID: E3A8D8FC-11A1-44C7-AE49-DBDADC80E86F

Notes:
LARC = Long-Acting Reversible Contraception. BC = Birth Control. CI = Confidence Interval.
n (%) = the number (percentage) of participants in each solicited adverse event and severity.
The denominator for percentages is based on the number of participants in the Safety population (N) with solicited adverse event data available after the first dose of study product.
A participant is only counted once within each solicited adverse event and severity.
95% CI = 95% Blaker Confidence Interval.
1: Each participant’s maximum severity is reported for each solicited adverse event across all doses.

Table 14.3.1.2.3: Number and Percentage of Participants Experiencing a Solicited Local Adverse Event of Vaginal Bleeding Other Than Menstruation with 95% Confidence Intervals after Study Product Start (Day 8-64) Among Participants With and Without Irregular Bleeding in 3 Months Prior to Start of Study Product by Treatment Group (Safety Population)

Table 14.3.1.2.3: Number and Percentage of Participants Experiencing a Solicited Local Adverse Event of Vaginal Bleeding Other Than Menstruation with 95% Confidence Intervals after Study Product Start (Day 8-64) Among Participants With and Without Irregular Bleeding in 3 Months Prior to Start of Study Product by Treatment Group
(Safety Population)

Solicited Adverse Event	Category	LACTIN-V (N=XX) n (%) [95% CI]	Placebo (N=XX) n (%) [95% CI]	All Participants (N=XX) n (%) [95% CI]
Vaginal Bleeding Other Than Menstruation				
ALL PARTICIPANTS	All	xx (xx.x%) [xx.x-xx.x]	xx (xx.x%) [xx.x-xx.x]	xx (xx.x%) [xx.x-xx.x]
	With Irregular Bleeding Prior to Study Product	xx (xx.x%) [xx.x-xx.x]	xx (xx.x%) [xx.x-xx.x]	xx (xx.x%) [xx.x-xx.x]
	Without Irregular Bleeding Prior to Study Product	xx (xx.x%) [xx.x-xx.x]	xx (xx.x%) [xx.x-xx.x]	xx (xx.x%) [xx.x-xx.x]
PARTICIPANTS ON BC OTHER THAN LARC	All	xx (xx.x%) [xx.x-xx.x]	xx (xx.x%) [xx.x-xx.x]	xx (xx.x%) [xx.x-xx.x]
	With Irregular Bleeding Prior to Study Product	xx (xx.x%) [xx.x-xx.x]	xx (xx.x%) [xx.x-xx.x]	xx (xx.x%) [xx.x-xx.x]
	Without Irregular Bleeding Prior to Study Product	xx (xx.x%) [xx.x-xx.x]	xx (xx.x%) [xx.x-xx.x]	xx (xx.x%) [xx.x-xx.x]
PARTICPANTS ON LARC 1-3 MONTHS	All	xx (xx.x%) [xx.x-xx.x]	xx (xx.x%) [xx.x-xx.x]	xx (xx.x%) [xx.x-xx.x]
	With Irregular Bleeding Prior to Study Product	xx (xx.x%) [xx.x-xx.x]	xx (xx.x%) [xx.x-xx.x]	xx (xx.x%) [xx.x-xx.x]
	Without Irregular Bleeding Prior to Study Product	xx (xx.x%) [xx.x-xx.x]	xx (xx.x%) [xx.x-xx.x]	xx (xx.x%) [xx.x-xx.x]

PARTICPANTS ON LARC >3 MONTHS	All	xx (xx.x%) [xx.x-xx.x]	xx (xx.x%) [xx.x-xx.x]	xx (xx.x%) [xx.x-xx.x]
	With Irregular Bleeding Prior to Study Product	xx (xx.x%) [xx.x-xx.x]	xx (xx.x%) [xx.x-xx.x]	xx (xx.x%) [xx.x-xx.x]
	Without Irregular Bleeding Prior to Study Product	xx (xx.x%) [xx.x-xx.x]	xx (xx.x%) [xx.x-xx.x]	xx (xx.x%) [xx.x-xx.x]

Notes:
LARC = Long-Acting Reversible Contraception. BC = Birth Control. CI = Confidence Interval.
n (%) = the number (percentage) of participants in each solicited adverse event and severity.
The denominator for percentages is based on the number of participants in the Safety population (N) with solicited adverse event data available after the first dose of study product.
A participant is only counted once within each solicited adverse event and severity.
95% CI = 95% Blaker Confidence Interval.
1: Each participant’s maximum severity is reported for each solicited adverse event across all doses.

DocuSign Envelope ID: E3A8D8FC-11A1-44C7-AE49-DBDADC80E86F

Table 14.3.1.3: Number and Percentage of Participants Experiencing Solicited Systemic Adverse Events with 95% Confidence Intervals by Symptom, Maximum Severity and Treatment Group (Safety Population)

<Table 14.3.1.3 has the same shell as Table 14.3.1.2.1, but with local adverse events replaced with systemic adverse events>

DocuSign Envelope ID: E3A8D8FC-11A1-44C7-AE49-DBDADC80E86F

Table 14.3.1.4: Proportion of Participants Reporting Product Related Unsolicited Adverse Events Following the First Dose of Study Product Through Visit 19 (Day 64) by Treatment Group (Safety Population)

Table 14.3.1.4: Proportion of Participants Reporting Product Related Unsolicited Adverse Events Following the First Dose of Study Product Through Visit 19 (Day 64) by Treatment Group
(Safety Population)

**Treatment Group**	**Number of Participants with Product Related Adverse Events n**	**Number of Participants N**	**Proportion of Participants with Product Related Adverse Events n/N**	**Proportion of Participants with Product Related Adverse Events 95% CI**
LACTIN-V	xx	xx	0.xxx	0.xxx-0.xxx
Placebo	xx	xx	0.xxx	0.xxx-0.xxx
All Participants	xx	xx	0.xxx	0.xxx-0.xxx

Notes:
CI = Confidence Interval.
The denominator for proportions is based on the number of participants in the Safety population (N) and treatment group.
95% CI = 95% Blaker Confidence Interval.

Table 14.3.1.5: Number and Percentage of Participants Experiencing Unsolicited Adverse Events with 95% Confidence Intervals by MedDRA System Organ Class and Preferred Term by Treatment Group (Safety Population)

Table 14.3.1.5: Number and Percentage of Participants Experiencing Unsolicited Adverse Events with 95% Confidence Intervals by MedDRA System Organ Class and Preferred Term by Treatment Group
(Safety Population)

MedDRA System Organ Class<br>MedDRA Preferred Term	LACTIN-V<br>(N=XX)<br>n (%) E [95% CI]	Placebo<br>(N=XX)<br>n (%) E [95% CI]	All Participants<br>(N=XX)<br>n (%) E [95% CI]
Any unsolicited adverse event	xx (xx.x%) xx [xx.x-xx.x]	xx (xx.x%) xx [xx.x-xx.x]	xx (xx.x%) xx [xx.x-xx.x]
System Organ Class 1	xx (xx.x%) xx [xx.x-xx.x]	xx (xx.x%) xx [xx.x-xx.x]	xx (xx.x%) xx [xx.x-xx.x]
Preferred Term 1	xx (xx.x%) xx [xx.x-xx.x]	xx (xx.x%) xx [xx.x-xx.x]	xx (xx.x%) xx [xx.x-xx.x]
Preferred Term 2	xx (xx.x%) xx [xx.x-xx.x]	xx (xx.x%) xx [xx.x-xx.x]	xx (xx.x%) xx [xx.x-xx.x]
…	xx (xx.x%) xx [xx.x-xx.x]	xx (xx.x%) xx [xx.x-xx.x]	xx (xx.x%) xx [xx.x-xx.x]
System Organ Class 2	xx (xx.x%) xx [xx.x-xx.x]	xx (xx.x%) xx [xx.x-xx.x]	xx (xx.x%) xx [xx.x-xx.x]
Preferred Term 1	xx (xx.x%) xx [xx.x-xx.x]	xx (xx.x%) xx [xx.x-xx.x]	xx (xx.x%) xx [xx.x-xx.x]
Preferred Term 2	xx (xx.x%) xx [xx.x-xx.x]	xx (xx.x%) xx [xx.x-xx.x]	xx (xx.x%) xx [xx.x-xx.x]
…	xx (xx.x%) xx [xx.x-xx.x]	xx (xx.x%) xx [xx.x-xx.x]	xx (xx.x%) xx [xx.x-xx.x]
Etc.	xx (xx.x%) xx [xx.x-xx.x]	xx (xx.x%) xx [xx.x-xx.x]	xx (xx.x%) xx [xx.x-xx.x]

Notes:
CI = Confidence Interval.
n (%) = the number (percentage) of participants in each MedDRA system organ class or preferred term.
E = the number of adverse events in each MedDRA system organ class or preferred term.
The denominator for percentages is based on the number of participants in the Safety population (N).
A participant is only counted once within each MedDRA system organ class or preferred term.
95% CI = 95% Blaker Confidence Interval.

DocuSign Envelope ID: E3A8D8FC-11A1-44C7-AE49-DBDADC80E86F

Table 14.3.1.6: Number and Percentage of Participants Experiencing Unsolicited Adverse Events by MedDRA System Organ Class and Preferred Term, Maximum Severity and Relationship by Treatment Group (Safety Population)

Table 14.3.1.6: Number and Percentage of Participants Experiencing Unsolicited Adverse Events by MedDRA System Organ Class and Preferred Term, Maximum Severity and Relationship by Treatment Group
(Safety Population)

		LACTIN-V (N=XX)			Placebo (N=XX)		
**MedDRA System Organ Class MedDRA Preferred Term**	**Severity**	**Related n (%) E**	**Not Related n (%) E**	**Total n (%) E**	**Related n (%) E**	**Not Related n (%) E**	**Total n (%) E**
Any unsolicited adverse event	Any Severity	xx (xx.x%) xx	xx (xx.x%) xx	xx (xx.x%) xx	xx (xx.x%) xx	xx (xx.x%) xx	xx (xx.x%) xx
	Mild	xx (xx.x%)	xx (xx.x%)	xx (xx.x%)	xx (xx.x%)	xx (xx.x%)	xx (xx.x%)
	Moderate	xx (xx.x%)	xx (xx.x%)	xx (xx.x%)	xx (xx.x%)	xx (xx.x%)	xx (xx.x%)
	Severe	xx (xx.x%)	xx (xx.x%)	xx (xx.x%)	xx (xx.x%)	xx (xx.x%)	xx (xx.x%)
System Organ Class 1	Any Severity	xx (xx.x%) xx	xx (xx.x%) xx	xx (xx.x%) xx	xx (xx.x%) xx	xx (xx.x%) xx	xx (xx.x%) xx
	Mild	xx (xx.x%)	xx (xx.x%)	xx (xx.x%)	xx (xx.x%)	xx (xx.x%)	xx (xx.x%)
	Moderate	xx (xx.x%)	xx (xx.x%)	xx (xx.x%)	xx (xx.x%)	xx (xx.x%)	xx (xx.x%)
	Severe	xx (xx.x%)	xx (xx.x%)	xx (xx.x%)	xx (xx.x%)	xx (xx.x%)	xx (xx.x%)
Preferred Term 1	Any Severity	xx (xx.x%) xx	xx (xx.x%) xx	xx (xx.x%) xx	xx (xx.x%) xx	xx (xx.x%) xx	xx (xx.x%) xx
	Mild	xx (xx.x%)	xx (xx.x%)	xx (xx.x%)	xx (xx.x%)	xx (xx.x%)	xx (xx.x%)
	Moderate	xx (xx.x%)	xx (xx.x%)	xx (xx.x%)	xx (xx.x%)	xx (xx.x%)	xx (xx.x%)
	Severe	xx (xx.x%)	xx (xx.x%)	xx (xx.x%)	xx (xx.x%)	xx (xx.x%)	xx (xx.x%)
Etc.							

Notes:
n (%) = the number (percentage) of participants in each MedDRA system organ class or preferred term.
E = the number of adverse events in each MedDRA system organ class or preferred term.
The denominator for percentages is based on the number of participants in the Safety population (N).
For severity, a participant is counted once per MedDRA system organ class or preferred term and is summarized according to their highest severity.

DocuSign Envelope ID: E3A8D8FC-11A1-44C7-AE49-DBDADC80E86F

Table 14.3.1.6: Number and Percentage of Participants Experiencing Unsolicited Adverse Events by MedDRA System Organ Class and Preferred Term, Maximum Severity and Relationship by Treatment Group
(Safety Population)

		All Participants (N=XX)		
**MedDRA System Organ Class<br>MedDRA Preferred Term**	**Severity**	**Related<br>n (%) E**	**Not Related<br>n (%) E**	**Total<br>n (%) E**
Any unsolicited adverse event	Any Severity	xx (xx.x%) xx	xx (xx.x%) xx	xx (xx.x%) xx
	Mild	xx (xx.x%)	xx (xx.x%)	xx (xx.x%)
	Moderate	xx (xx.x%)	xx (xx.x%)	xx (xx.x%)
	Severe	xx (xx.x%)	xx (xx.x%)	xx (xx.x%)
System Organ Class 1	Any Severity	xx (xx.x%) xx	xx (xx.x%) xx	xx (xx.x%) xx
	Mild	xx (xx.x%)	xx (xx.x%)	xx (xx.x%)
	Moderate	xx (xx.x%)	xx (xx.x%)	xx (xx.x%)
	Severe	xx (xx.x%)	xx (xx.x%)	xx (xx.x%)
Preferred Term 1	Any Severity	xx (xx.x%) xx	xx (xx.x%) xx	xx (xx.x%) xx
	Mild	xx (xx.x%)	xx (xx.x%)	xx (xx.x%)
	Moderate	xx (xx.x%)	xx (xx.x%)	xx (xx.x%)
	Severe	xx (xx.x%)	xx (xx.x%)	xx (xx.x%)
Etc.				

Notes:
n (%) = the number (percentage) of participants in each MedDRA system organ class or preferred term.
E = the number of adverse events in each MedDRA system organ class or preferred term.
The denominator for percentages is based on the number of participants in the Safety population (N).
For severity, a participant is counted once per MedDRA system organ class or preferred term and is summarized according to their highest severity.

DocuSign Envelope ID: E3A8D8FC-11A1-44C7-AE49-DBDADC80E86F

Table 14.3.1.7: Participants Reporting Adverse Events Occurring in 5% of Participants in Any Treatment Group by MedDRA System Organ Class and Preferred Term, and Treatment Group (Safety Population)

Table 14.3.1.7: Participants Reporting Adverse Events Occurring in 5% of Participants in Any Treatment Group by MedDRA System Organ Class and Preferred Term, and Treatment Group
(Safety Population)

MedDRA Preferred Term	MedDRA System Organ Class	MedDRA Version	LACTIN-V (N=XX) n [E]	Placebo (N=XX) n [E]	All Participants (N=XX) n [E]
Xxxxx	Xxxxx	xx.x	xx [xx]	xx [xx]	xx [xx]

Notes:
N = the number of participants in the Safety population.
n = the number of participants reporting the adverse event.
E = the number of adverse events.

Table 14.3.4.1: Summary of Urine Pregnancy Test Results (Safety Population)

Table 14.3.4.1: Summary of Urine Pregnancy Test Results
(Safety Population)

Parameter	Visit	Value	LACTIN-V (N=XX) n (%)	Placebo (N=XX) n (%)	All Participants (N=XX) n (%)
Urine pregnancy test (n (%))	Visit 1/Day 1	Negative	xx (xx.x%)	xx (xx.x%)	xx (xx.x%)
		Positive	xx (xx.x%)	xx (xx.x%)	xx (xx.x%)
		N/A (hysterectomy)	xx (xx.x%)	xx (xx.x%)	xx (xx.x%)
	Visit 3/Day 8	Negative	xx (xx.x%)	xx (xx.x%)	xx (xx.x%)
		Positive	xx (xx.x%)	xx (xx.x%)	xx (xx.x%)
		N/A (hysterectomy)	xx (xx.x%)	xx (xx.x%)	xx (xx.x%)
	Etc.				

Notes:
n (%) = the number (percentage) of participants in each result category.
The denominator for percentages is based on the number of participants in the Safety population (N) with data available.

DocuSign Envelope ID: E3A8D8FC-11A1-44C7-AE49-DBDADC80E86F

Table 14.3.4.2: Summary of Urinalysis Clinic Results (Safety Population)

Table 14.3.4.2.1: Summary of Urinalysis Clinic Results
(Safety Population)

Parameter	Visit	Value	LACTIN-V (N=XX) n (%)	Placebo (N=XX) n (%)	All Participants (N=XX) n (%)
Protein (n (%))	Visit 1/Day 1	Negative	xx (xx.x%)	xx (xx.x%)	xx (xx.x%)
		Trace	xx (xx.x%)	xx (xx.x%)	xx (xx.x%)
		1+	xx (xx.x%)	xx (xx.x%)	xx (xx.x%)
		2+	xx (xx.x%)	xx (xx.x%)	xx (xx.x%)
		3+	xx (xx.x%)	xx (xx.x%)	xx (xx.x%)
	Visit 3/Day 8	Negative	xx (xx.x%)	xx (xx.x%)	xx (xx.x%)
		Trace	xx (xx.x%)	xx (xx.x%)	xx (xx.x%)
		1+	xx (xx.x%)	xx (xx.x%)	xx (xx.x%)
		2+	xx (xx.x%)	xx (xx.x%)	xx (xx.x%)
		3+	xx (xx.x%)	xx (xx.x%)	xx (xx.x%)
	Etc.				
Blood (n (%))		…			
Nitrite (n (%))		…			
Leukocyte Esterase (n (%))		…			

Notes:
n (%) = the number (percentage) of participants in each result category.
The denominator for percentages is based on the number of participants in the Safety population (N) with data available.

DocuSign Envelope ID: E3A8D8FC-11A1-44C7-AE49-DBDADC80E86F

Table 14.3.5.1: Gynaecological Review by Treatment Group (ITT Population)

Table 14.3.5.1: Gynaecological Review by Treatment Group
(ITT Population)

Interview Question	Visit	Value / Statistic	LACTIN-V (N=XX)	Placebo (N=XX)	All Participants (N=XX)
Participant had hysterectomy (n (%))	Visit 3/Day 8	Yes	xx (xx.x%)	xx (xx.x%)	xx (xx.x%)
	Visit 11/Day 36	Yes	xx (xx.x%)	xx (xx.x%)	xx (xx.x%)
	Visit 19/Day 64	Yes	xx (xx.x%)	xx (xx.x%)	xx (xx.x%)
Participant did not menstruate or have any other bleeding since the last visit (n (%))	Visit 3/Day 8	Yes	xx (xx.x%)	xx (xx.x%)	xx (xx.x%)
	Visit 11/Day 36	Yes	xx (xx.x%)	xx (xx.x%)	xx (xx.x%)
	Visit 19/Day 64	Yes	xx (xx.x%)	xx (xx.x%)	xx (xx.x%)
Since your last visit, have you experienced any spotting or bleeding, other than menstrual bleeding? (n (%))	Visit 3/Day 8	Yes	xx (xx.x%)	xx (xx.x%)	xx (xx.x%)
		No	xx (xx.x%)	xx (xx.x%)	xx (xx.x%)
	Visit 11/Day 36	Yes	xx (xx.x%)	xx (xx.x%)	xx (xx.x%)
		No	xx (xx.x%)	xx (xx.x%)	xx (xx.x%)
	Visit 19/Day 64	Yes	xx (xx.x%)	xx (xx.x%)	xx (xx.x%)
		No	xx (xx.x%)	xx (xx.x%)	xx (xx.x%)
Since your last visit, have you douched or vaginally inserted anything other than the study applicator (vaginal drying agents, sexual stimulants, etc.)? (n (%))	Visit 3/Day 8	Yes	xx (xx.x%)	xx (xx.x%)	xx (xx.x%)
		No	xx (xx.x%)	xx (xx.x%)	xx (xx.x%)
	Visit 11/Day 36	Yes	xx (xx.x%)	xx (xx.x%)	xx (xx.x%)
		No	xx (xx.x%)	xx (xx.x%)	xx (xx.x%)
	Visit 19/Day 64	Yes	xx (xx.x%)	xx (xx.x%)	xx (xx.x%)
		No	xx (xx.x%)	xx (xx.x%)	xx (xx.x%)

Since your last visit, what sanitary products have you used when bleeding/spotting? (n (%))	Visit …	Pads	xx (xx.x%)	xx (xx.x%)	xx (xx.x%)
		Cups	xx (xx.x%)	xx (xx.x%)	xx (xx.x%)
		Tampons	xx (xx.x%)	xx (xx.x%)	xx (xx.x%)
		Other	xx (xx.x%)	xx (xx.x%)	xx (xx.x%)
How long ago did you douche or vaginally insert anything other than the applicator?	Visit …	n	xx	xx	xx
		Mean (Standard Deviation)	xx.x (xx.xx)	xx.x (xx.xx)	xx.x (xx.xx)
		Median	xx.x	xx.x	xx.x
		Minimum, Maximum	xx, xx	xx, xx	xx, xx
Since your last visit, how many times have you douched or vaginally inserted anything other than the applicator?		n	xx	xx	xx
		Mean (Standard Deviation)	xx.x (xx.xx)	xx.x (xx.xx)	xx.x (xx.xx)
		Median	xx.x	xx.x	xx.x
		Minimum, Maximum	xx, xx	xx, xx	xx, xx
SEXUAL HISTORY					
Since your last visit, how many times did you have vaginal sex?		n	xx	xx	xx
		Mean (Standard Deviation)	xx.x (xx.xx)	xx.x (xx.xx)	xx.x (xx.xx)
		Median	xx.x	xx.x	xx.x
		Minimum, Maximum	xx, xx	xx, xx	xx, xx
Since your last visit, have you had any new boyfriends or partners that you had vaginal sex with? (n (%))		Yes	xx (xx.x%)	xx (xx.x%)	xx (xx.x%)
		No	xx (xx.x%)	xx (xx.x%)	xx (xx.x%)
How many new partners?		n	xx	xx	xx
		Mean (Standard Deviation)	xx.x (xx.xx)	xx.x (xx.xx)	xx.x (xx.xx)
		Median	xx.x	xx.x	xx.x
		Minimum, Maximum	xx, xx	xx, xx	xx, xx

How many days ago did you last have vaginal sex?	n	xx	xx	xx
	Mean (Standard Deviation)	xx.x (xx.xx)	xx.x (xx.xx)	xx.x (xx.xx)
	Median	xx.x	xx.x	xx.x
	Minimum, Maximum	xx, xx	xx, xx	xx, xx
If you had sex with a man, did your partner use condoms each time?	Yes	xx (xx.x%)	xx (xx.x%)	xx (xx.x%)
	No	xx (xx.x%)	xx (xx.x%)	xx (xx.x%)
Since your last visit, how many times did you have anal sex?	n	xx	xx	xx
	Mean (Standard Deviation)	xx.x (xx.xx)	xx.x (xx.xx)	xx.x (xx.xx)
	Median	xx.x	xx.x	xx.x
	Minimum, Maximum	xx, xx	xx, xx	xx, xx

Notes:
ITT = Intent-to-Treat.
n (%) = the number (percentage) of participants in each category.
n = the number of participants with data available.
The denominator for percentages is based on the number of participants in the ITT population (N) with data available.

DocuSign Envelope ID: E3A8D8FC-11A1-44C7-AE49-DBDADC80E86F

Table 14.3.5.2: Acceptability Questionnaire Responses by Treatment Group (Safety Population)

Table 14.3.5.2: Acceptability Questionnaire Responses by Treatment Group
(Safety Population)

**Questionnaire Item**	**Response / Statistic**	**LACTIN-V (N=XX)**	**Placebo (N=XX)**	**All Participants (N=XX)**
I was satisfied with the applicator (n (%))	Strongly agree	xx (xx.x%)	xx (xx.x%)	xx (xx.x%)
	Agree	xx (xx.x%)	xx (xx.x%)	xx (xx.x%)
	Neutral	xx (xx.x%)	xx (xx.x%)	xx (xx.x%)
	Disagree	xx (xx.x%)	xx (xx.x%)	xx (xx.x%)
	Strongly disagree	xx (xx.x%)	xx (xx.x%)	xx (xx.x%)
The applicator was comfortable when inserted (n (%))	Strongly agree	xx (xx.x%)	xx (xx.x%)	xx (xx.x%)
	Agree	xx (xx.x%)	xx (xx.x%)	xx (xx.x%)
	Neutral	xx (xx.x%)	xx (xx.x%)	xx (xx.x%)
	Disagree	xx (xx.x%)	xx (xx.x%)	xx (xx.x%)
	Strongly disagree	xx (xx.x%)	xx (xx.x%)	xx (xx.x%)
The applicator was easy to insert and use (n (%))	Strongly agree	xx (xx.x%)	xx (xx.x%)	xx (xx.x%)
	Agree	xx (xx.x%)	xx (xx.x%)	xx (xx.x%)
	Neutral	xx (xx.x%)	xx (xx.x%)	xx (xx.x%)
	Disagree	xx (xx.x%)	xx (xx.x%)	xx (xx.x%)
	Strongly disagree	xx (xx.x%)	xx (xx.x%)	xx (xx.x%)
It is important to me that I could use the product without my partner knowing (n (%))	Strongly agree	xx (xx.x%)	xx (xx.x%)	xx (xx.x%)
	Agree	xx (xx.x%)	xx (xx.x%)	xx (xx.x%)
	Neutral	xx (xx.x%)	xx (xx.x%)	xx (xx.x%)
	Disagree	xx (xx.x%)	xx (xx.x%)	xx (xx.x%)
	Strongly disagree	xx (xx.x%)	xx (xx.x%)	xx (xx.x%)

It is important to me that my partner/boyfriend approves of the product (n (%))	Strongly agree	xx (xx.x%)	xx (xx.x%)	xx (xx.x%)
	Agree	xx (xx.x%)	xx (xx.x%)	xx (xx.x%)
	Neutral	xx (xx.x%)	xx (xx.x%)	xx (xx.x%)
	Disagree	xx (xx.x%)	xx (xx.x%)	xx (xx.x%)
	Strongly disagree	xx (xx.x%)	xx (xx.x%)	xx (xx.x%)
My current partner's reaction to the product was: (n (%))	I currently don't have a partner	xx (xx.x%)	xx (xx.x%)	xx (xx.x%)
	Positive	xx (xx.x%)	xx (xx.x%)	xx (xx.x%)
	Neutral	xx (xx.x%)	xx (xx.x%)	xx (xx.x%)
	Negative	xx (xx.x%)	xx (xx.x%)	xx (xx.x%)
	Unknown	xx (xx.x%)	xx (xx.x%)	xx (xx.x%)
	My partner was unaware I was using this product	xx (xx.x%)	xx (xx.x%)	xx (xx.x%)
My current partner's reaction to the product affected my use of the product (n (%))	Yes, and I was more likely to use it	xx (xx.x%)	xx (xx.x%)	xx (xx.x%)
	Yes, and I was less likely to use it	xx (xx.x%)	xx (xx.x%)	xx (xx.x%)
	No	xx (xx.x%)	xx (xx.x%)	xx (xx.x%)
	Unknown	xx (xx.x%)	xx (xx.x%)	xx (xx.x%)
Did you experience any side effects using the product? (n (%))	Yes	xx (xx.x%)	xx (xx.x%)	xx (xx.x%)
	No	xx (xx.x%)	xx (xx.x%)	xx (xx.x%)
If yes, would these side effects make you less likely to use the product again? (n (%))	Strongly agree	xx (xx.x%)	xx (xx.x%)	xx (xx.x%)
	Agree	xx (xx.x%)	xx (xx.x%)	xx (xx.x%)
	Neutral	xx (xx.x%)	xx (xx.x%)	xx (xx.x%)
	Disagree	xx (xx.x%)	xx (xx.x%)	xx (xx.x%)
	Strongly disagree	xx (xx.x%)	xx (xx.x%)	xx (xx.x%)

On a scale of 0 to 10, with 0 being "not at all" to 10 being "extremely", I found the product to be:				
Effective	n	xx	xx	xx
	Mean (Standard Deviation)	xx.x (xx.xx)	xx.x (xx.xx)	xx.x (xx.xx)
	Median	xx.x	xx.x	xx.x
	Minimum, Maximum	xx, xx	xx, xx	xx, xx
Comfortable	n	xx	xx	xx
	Mean (Standard Deviation)	xx.x (xx.xx)	xx.x (xx.xx)	xx.x (xx.xx)
	Median	xx.x	xx.x	xx.x
	Minimum, Maximum	xx, xx	xx, xx	xx, xx
Easy to use	n	xx	xx	xx
	Mean (Standard Deviation)	xx.x (xx.xx)	xx.x (xx.xx)	xx.x (xx.xx)
	Median	xx.x	xx.x	xx.x
	Minimum, Maximum	xx, xx	xx, xx	xx, xx
On a scale of 0 to 10, with 0 being "not at all" to 10 being "extremely", the following things made the product hard for you to use:				
Frequency or timing of using the product	n	xx	xx	xx
	Mean (Standard Deviation)	xx.x (xx.xx)	xx.x (xx.xx)	xx.x (xx.xx)
	Median	xx.x	xx.x	xx.x
	Minimum, Maximum	xx, xx	xx, xx	xx, xx
Vaginal dryness	n	xx	xx	xx
	Mean (Standard Deviation)	xx.x (xx.xx)	xx.x (xx.xx)	xx.x (xx.xx)
	Median	xx.x	xx.x	xx.x
	Minimum, Maximum	xx, xx	xx, xx	xx, xx
"Messiness" or leakage / discharge of the product	n	xx	xx	xx
	Mean (Standard Deviation)	xx.x (xx.xx)	xx.x (xx.xx)	xx.x (xx.xx)
	Median	xx.x	xx.x	xx.x
	Minimum, Maximum	xx, xx	xx, xx	xx, xx

Partner's disapproval	n	xx	xx	xx
	Mean (Standard Deviation)	xx.x (xx.xx)	xx.x (xx.xx)	xx.x (xx.xx)
	Median	xx.x	xx.x	xx.x
	Minimum, Maximum	xx, xx	xx, xx	xx, xx
Other	No other issues mentioned	xx (xx.x%)	xx (xx.x%)	xx (xx.x%)
	n	xx	xx	xx
	Mean (Standard Deviation)	xx.x (xx.xx)	xx.x (xx.xx)	xx.x (xx.xx)
	Median	xx.x	xx.x	xx.x
	Minimum, Maximum	xx, xx	xx, xx	xx, xx
I would use the product again (n (%))	Strongly agree	xx (xx.x%)	xx (xx.x%)	xx (xx.x%)
	Agree	xx (xx.x%)	xx (xx.x%)	xx (xx.x%)
	Neutral	xx (xx.x%)	xx (xx.x%)	xx (xx.x%)
	Disagree	xx (xx.x%)	xx (xx.x%)	xx (xx.x%)
	Strongly disagree	xx (xx.x%)	xx (xx.x%)	xx (xx.x%)

Notes:
n (%) = the number (percentage) of participants in each response.
n = the number of participants with data available.
The denominator for percentages is based on the number of participants in the Safety population (N) with data available.

DocuSign Envelope ID: E3A8D8FC-11A1-44C7-AE49-DBDADC80E86F

7. FIGURE SHELLS

DocuSign Envelope ID: E3A8D8FC-11A1-44C7-AE49-DBDADC80E86F

Figure 14.1.1: CONSORT Flow Diagram

Figure 14.1.1: CONSORT Flow Diagram

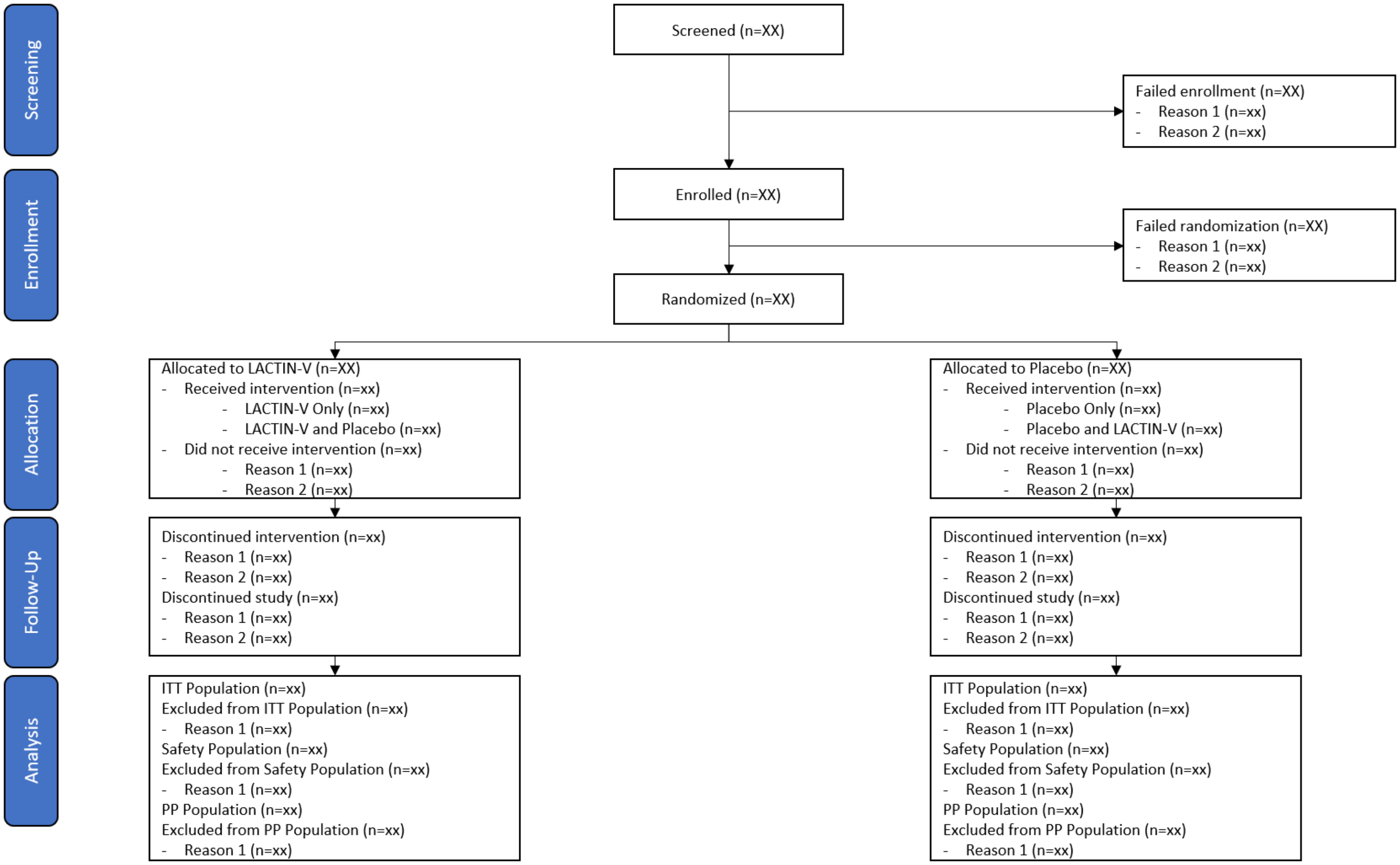

Figure 14.2.1.1: HIV Target Cells by Group and Visit

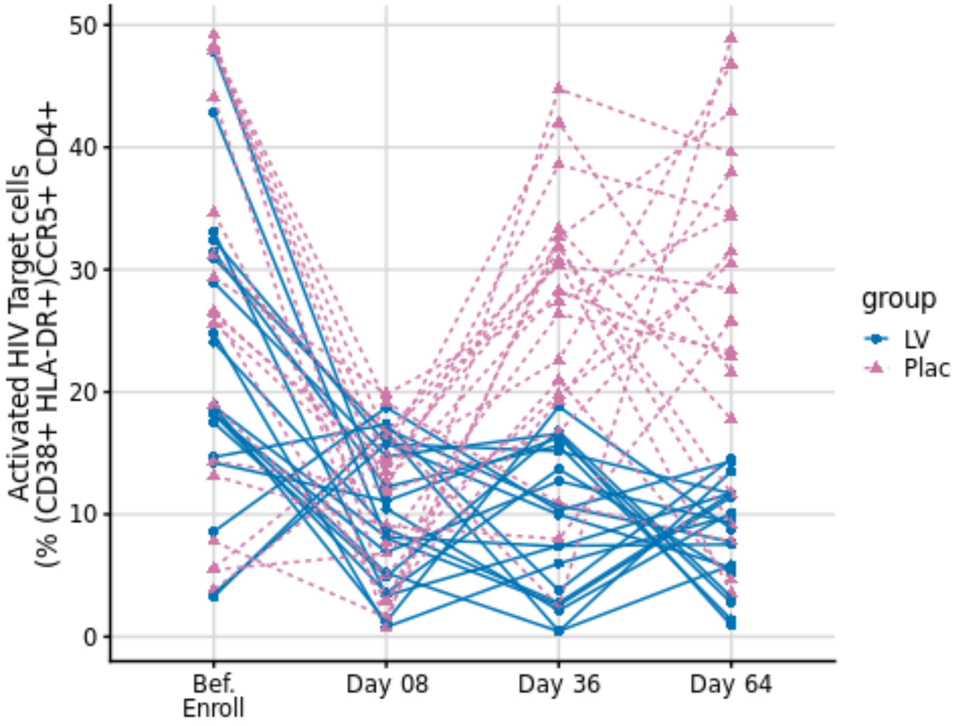

Figure 14.2.1.2: Immune Cells by Group and Visit (Representative Cell Types Shown Here)

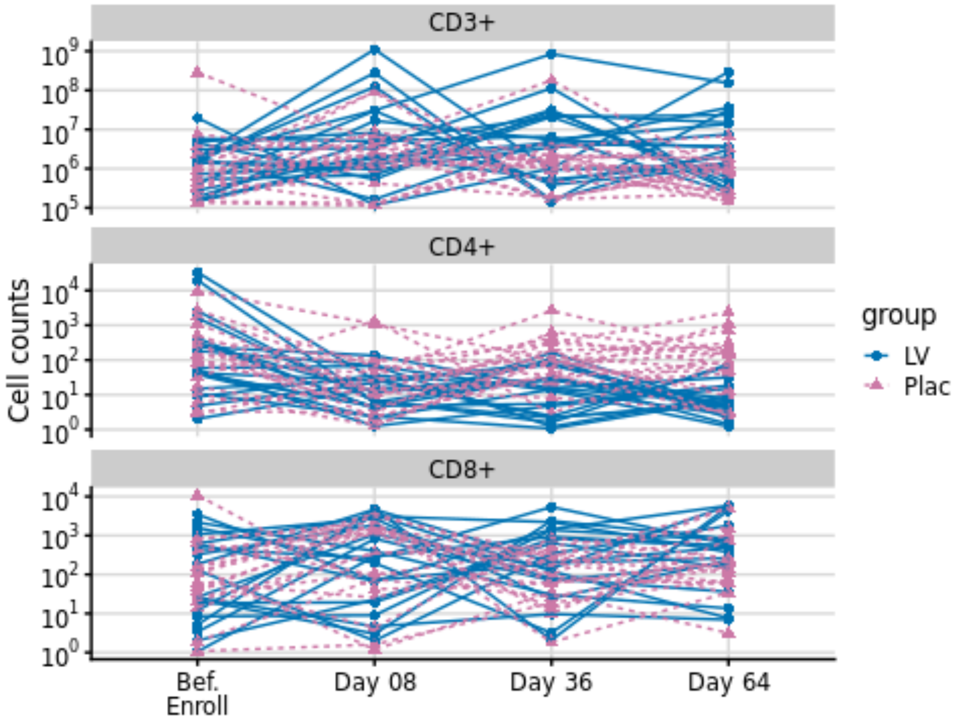

Figure 14.2.1.3: Inflammation Index by Treatment Group and Visit

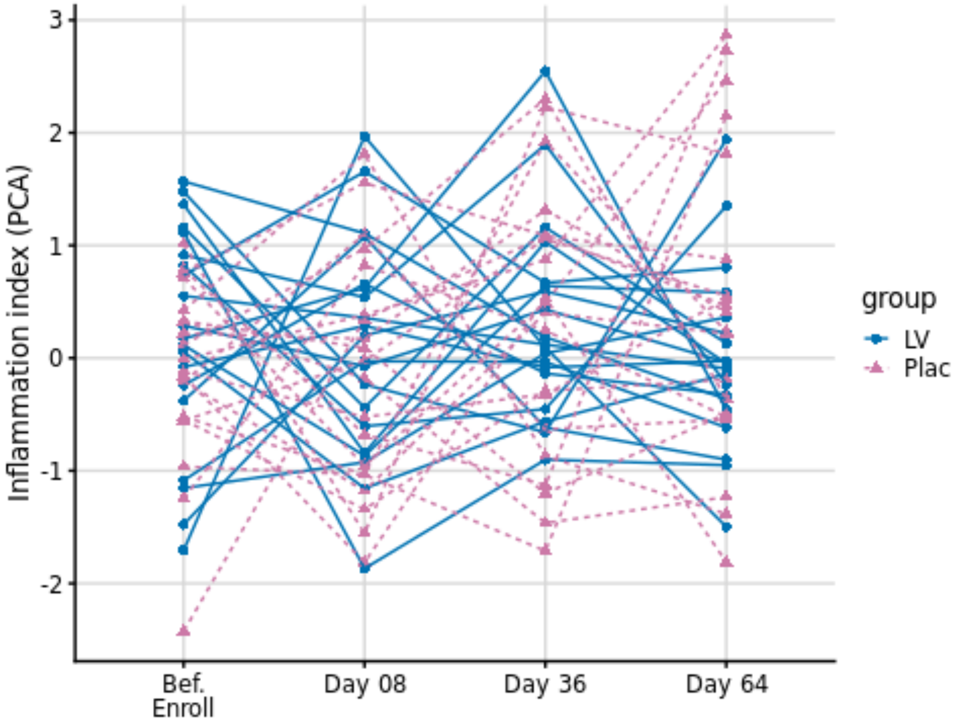

DocuSign Envelope ID: E3A8D8FC-11A1-44C7-AE49-DBDADC80E86F

Figure 14.2.1.4: Individual Cytokines by Treatment Group and Visit

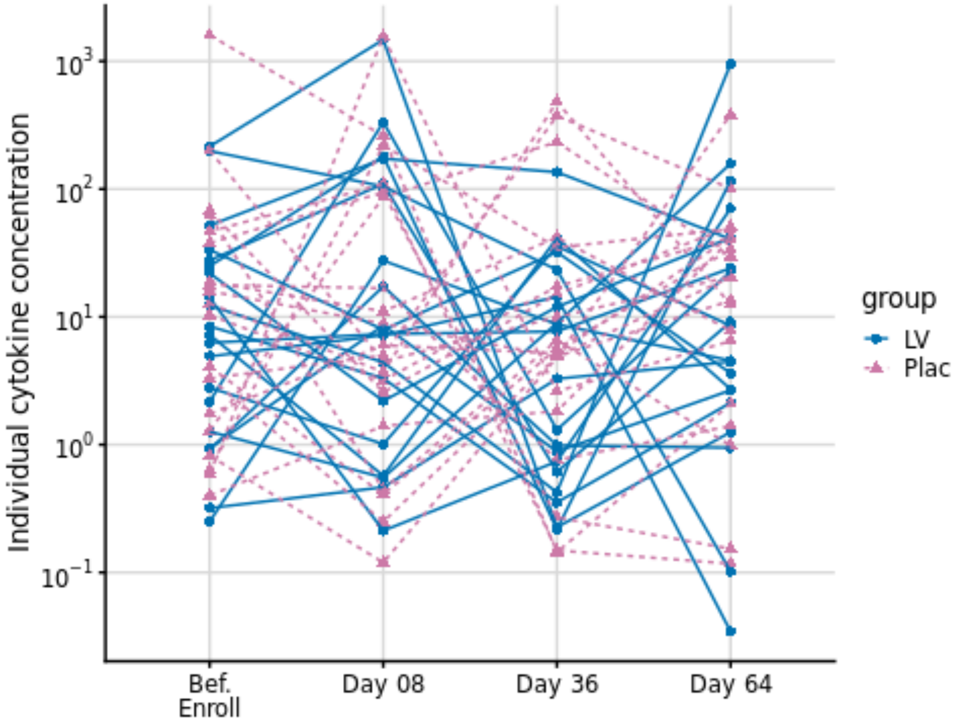

DocuSign Envelope ID: E3A8D8FC-11A1-44C7-AE49-DBDADC80E86F

Figure 14.2.1.5: *L. Crispatus* Relative Abundance by Treatment Group and Visit

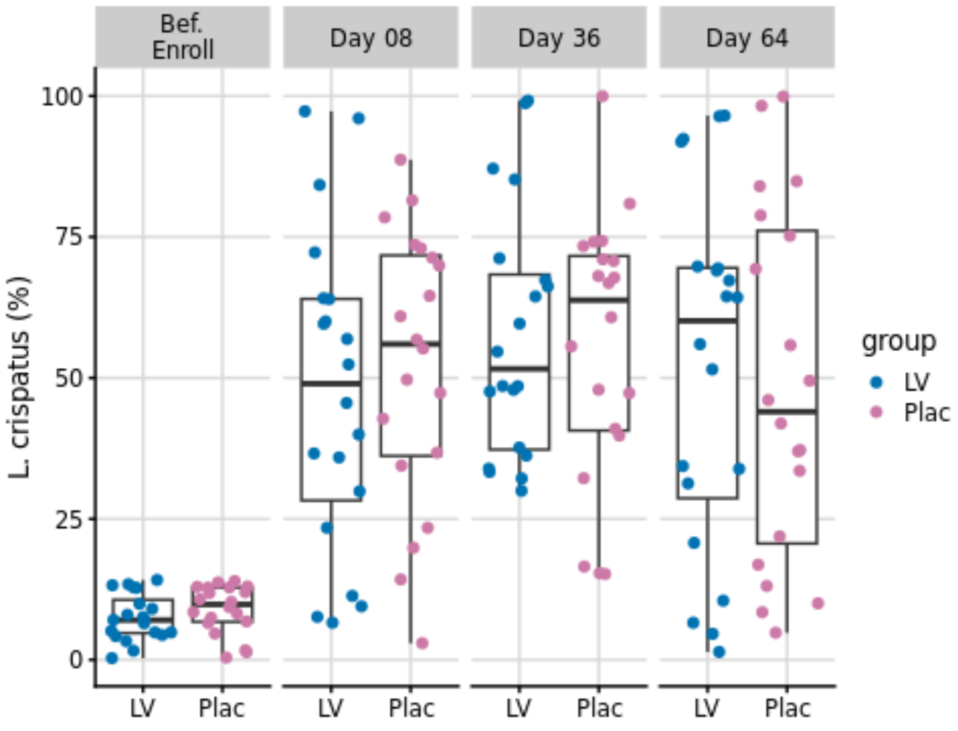

DocuSign Envelope ID: E3A8D8FC-11A1-44C7-AE49-DBDADC80E86F

Figure 14.2.1.6: Community Type (CT) Percentages by Treatment Group and Visit

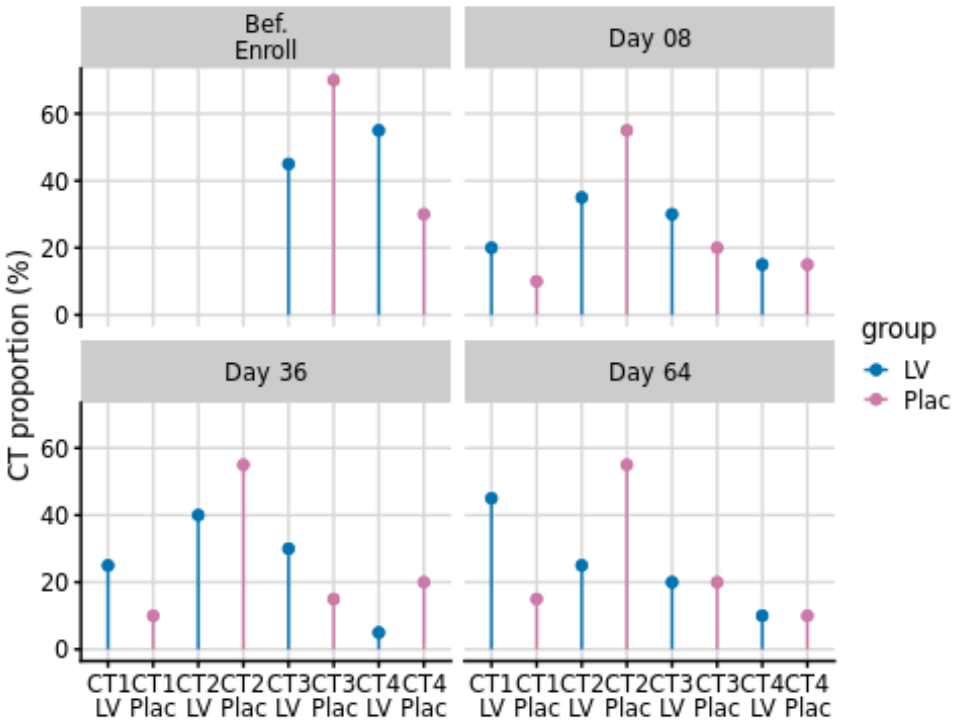

DocuSign Envelope ID: E3A8D8FC-11A1-44C7-AE49-DBDADC80E86F

Figure 14.2.1.7: CTV-05 Abundance Measured by qPCR by Treatment Group and Visit

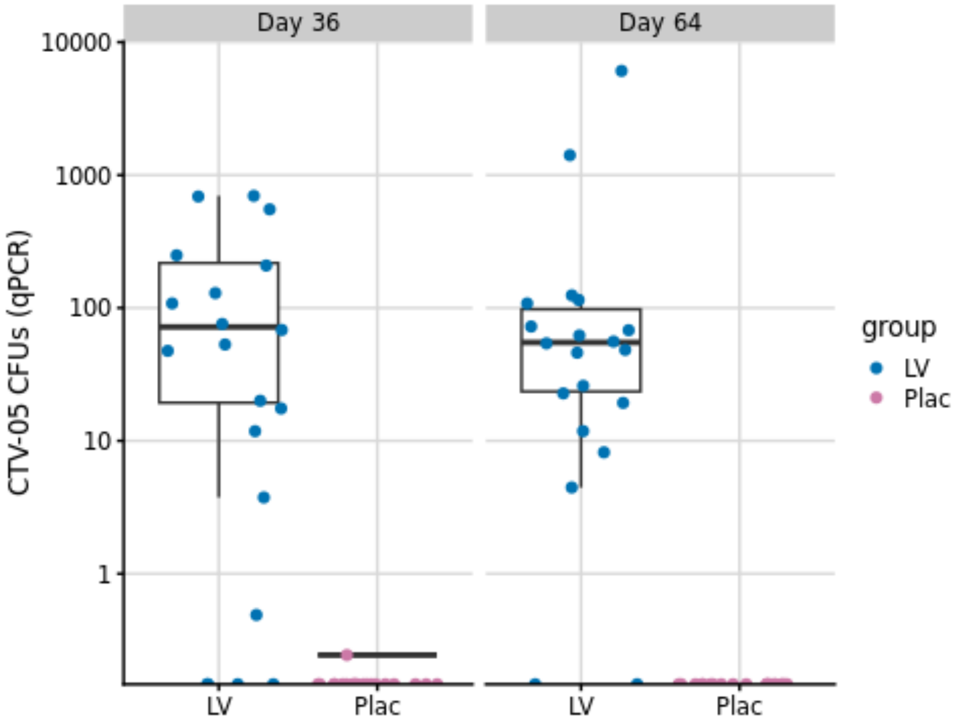

\

Figure 14.2.1.8: *L. Iners* Abundance Measured by qPCR by Treatment Group and Visit

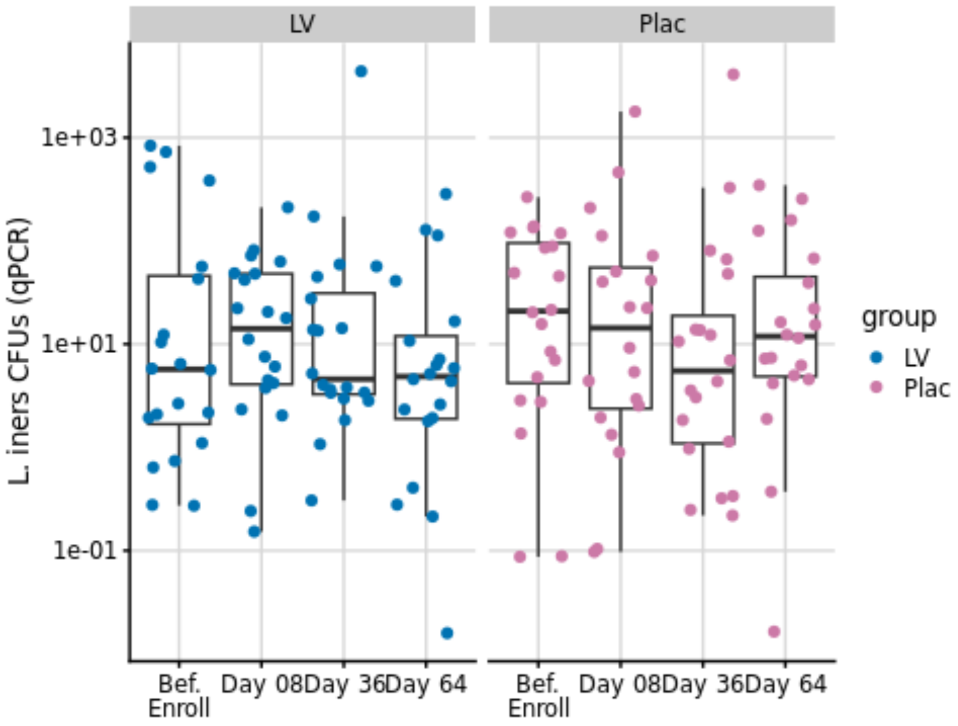

Figure 14.2.1.9: Total Bacterial Abundance Measured by qPCR by Treatment Group and Visit

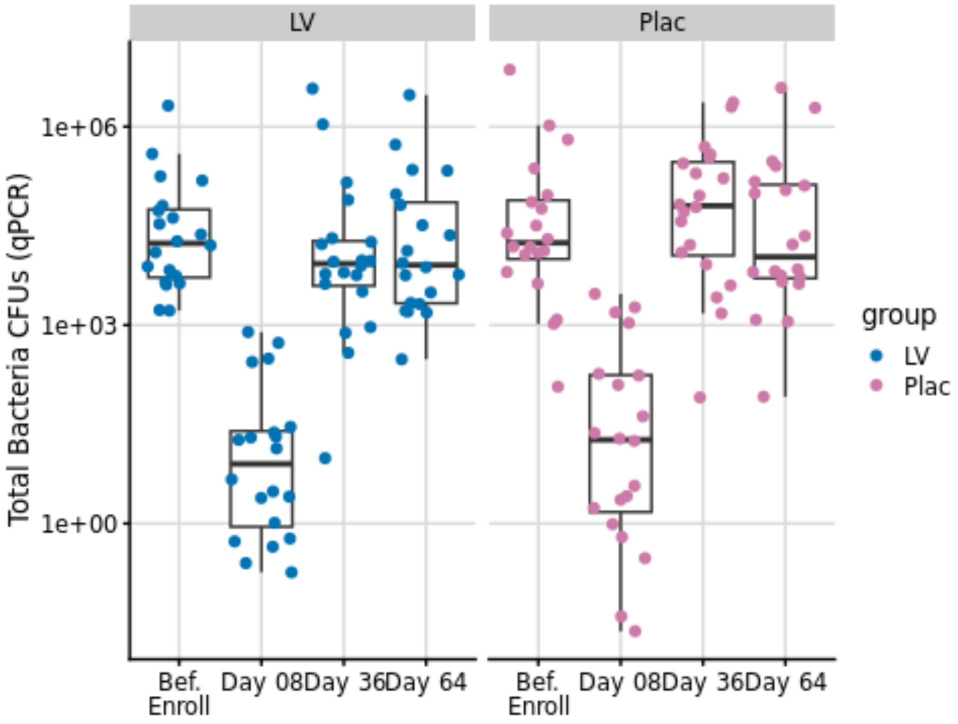

Figure 14.3.1.1: Maximum Severity of Solicited Local Adverse Events by Symptom and Treatment Group (Safety Population)

Figure 14.3.1.1: Maximum Severity of Solicited Local Adverse Events by Symptom and Treatment Group (Safety Population)

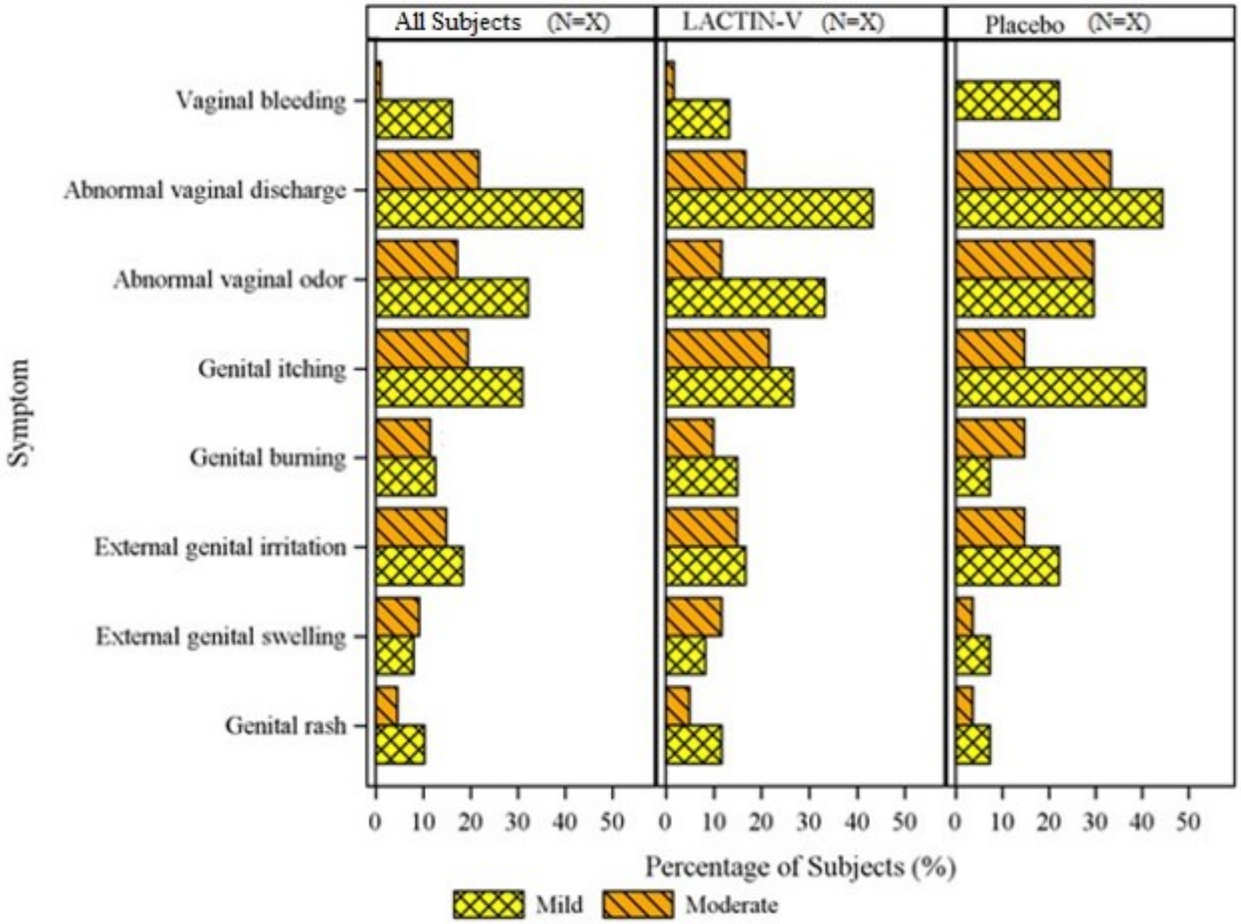

DocuSign Envelope ID: E3A8D8FC-11A1-44C7-AE49-DBDADC80E86F

Figure 14.3.1.2: Maximum Severity of Solicited Systemic Adverse Events by Symptom and Treatment Group (Safety Population)

Figure 14.3.1.2: Maximum Severity of Solicited Systemic Adverse Events by Symptom and Treatment Group (Safety Population)

<Figure 14.3.1.2 has the same shell as Figure 14.3.1.1, but with local adverse events replaced with systemic adverse events>

DocuSign Envelope ID: E3A8D8FC-11A1-44C7-AE49-DBDADC80E86F

Figure 14.3.1.3: Maximum Severity of Solicited Local Adverse Events by Study Week and Treatment Group (Safety Population)

Figure 14.3.1.3: Maximum Severity of Solicited Local Adverse Events by Study Week and Treatment Group (Safety Population)

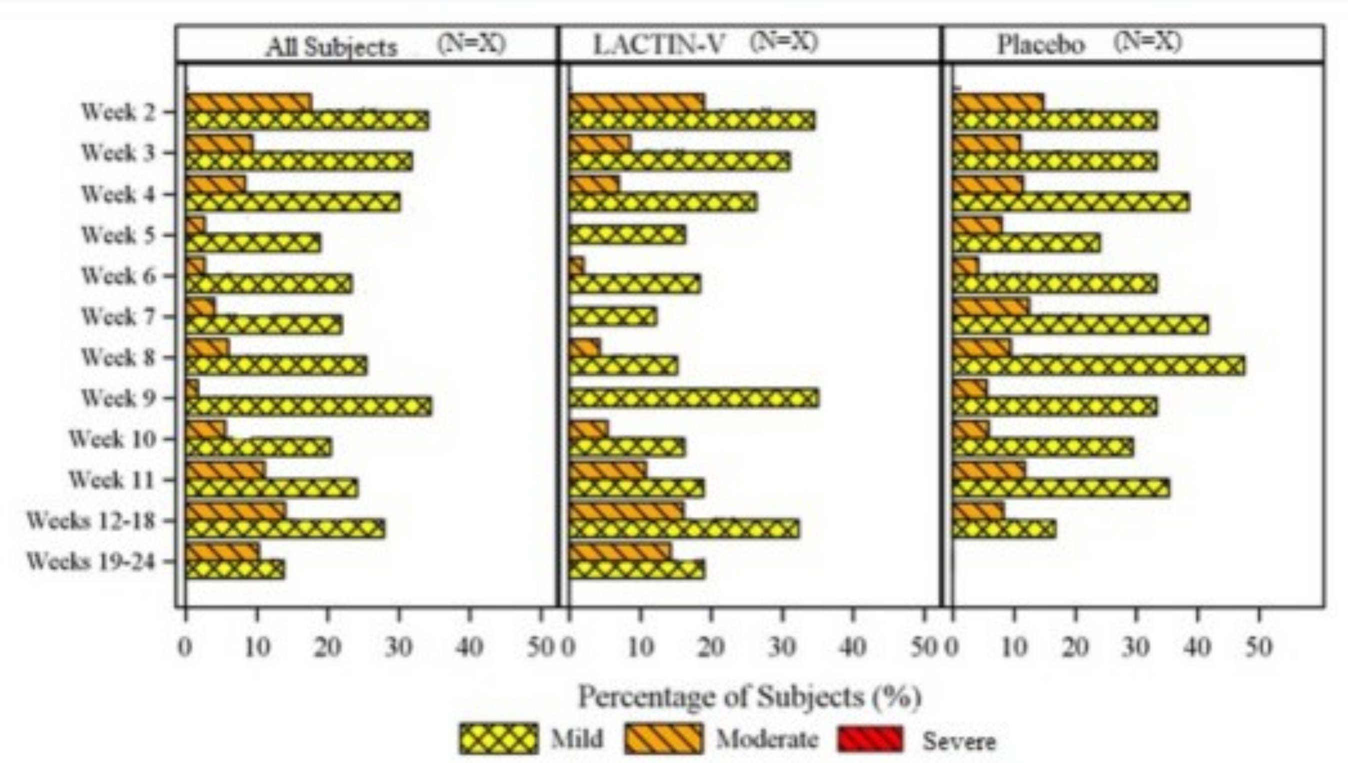

Figure 14.3.1.4: Maximum Severity of Solicited Systemic Adverse Events by Study Week and Treatment Group (Safety Population)

Figure 14.3.1.4: Maximum Severity of Solicited Systemic Adverse Events by Study Week and Treatment Group
(Safety Population)

<Figure 14.3.1.4 has the same shell as Figure 14.3.1.3, but with local adverse events replaced with systemic adverse events>

DocuSign Envelope ID: E3A8D8FC-11A1-44C7-AE49-DBDADC80E86F

Figure 14.3.1.5: Frequency of Unsolicited Adverse Events by MedDRA System Organ Class, Severity and Treatment Group (Safety Population)

Figure 14.3.1.5: Frequency of Unsolicited Adverse Events by MedDRA System Organ Class, Severity and Treatment Group
(Safety Population)

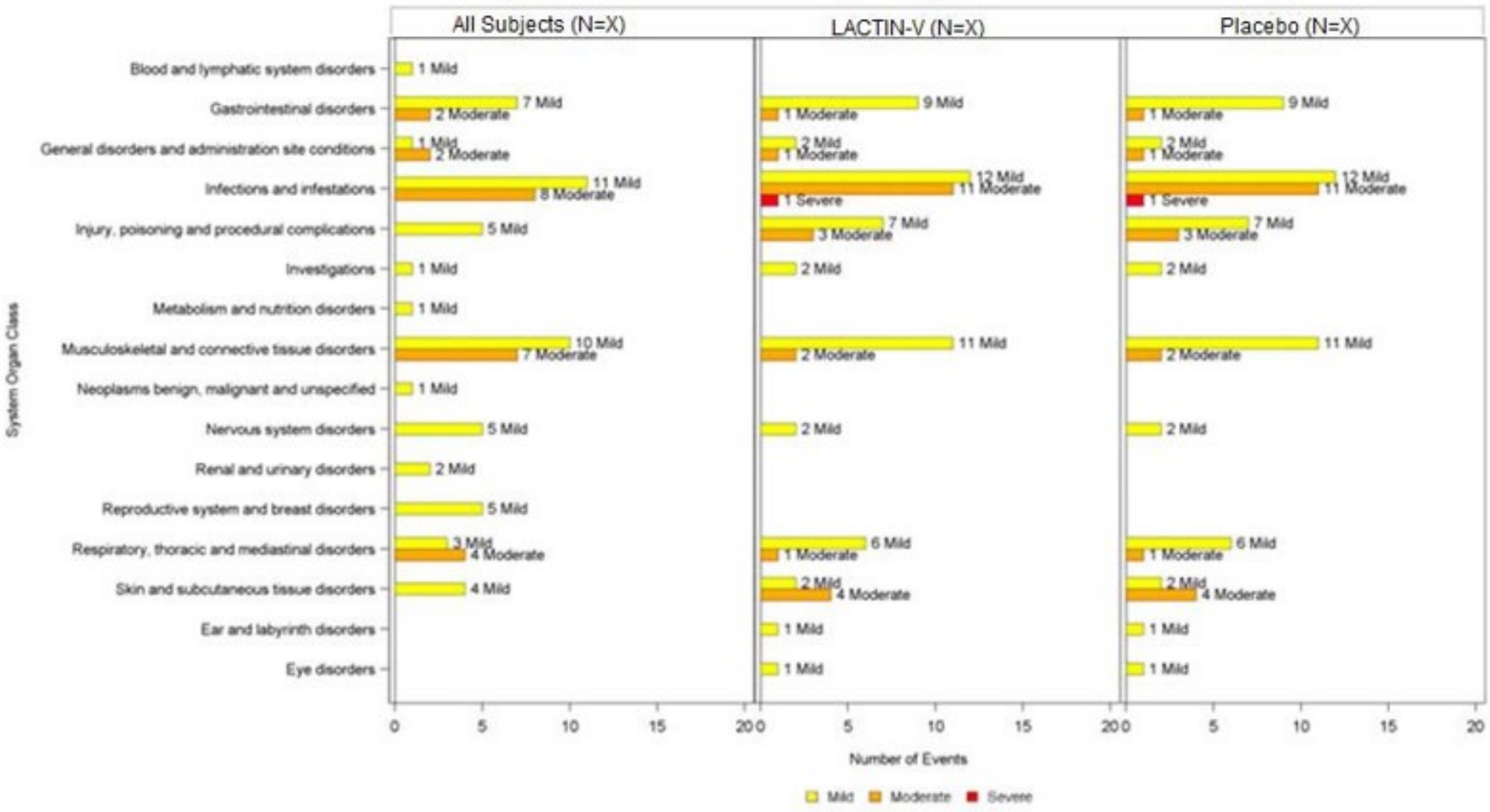

<Include “Any System Organ Class” bars>

Figure 14.3.1.6: Incidence of Unsolicited Adverse Events by MedDRA System Organ Class, Maximum Severity and Treatment Group (Safety Population)

Figure 14.3.1.6: Incidence of Unsolicited Adverse Events by MedDRA System Organ Class, Maximum Severity and Treatment Group (Safety Population)

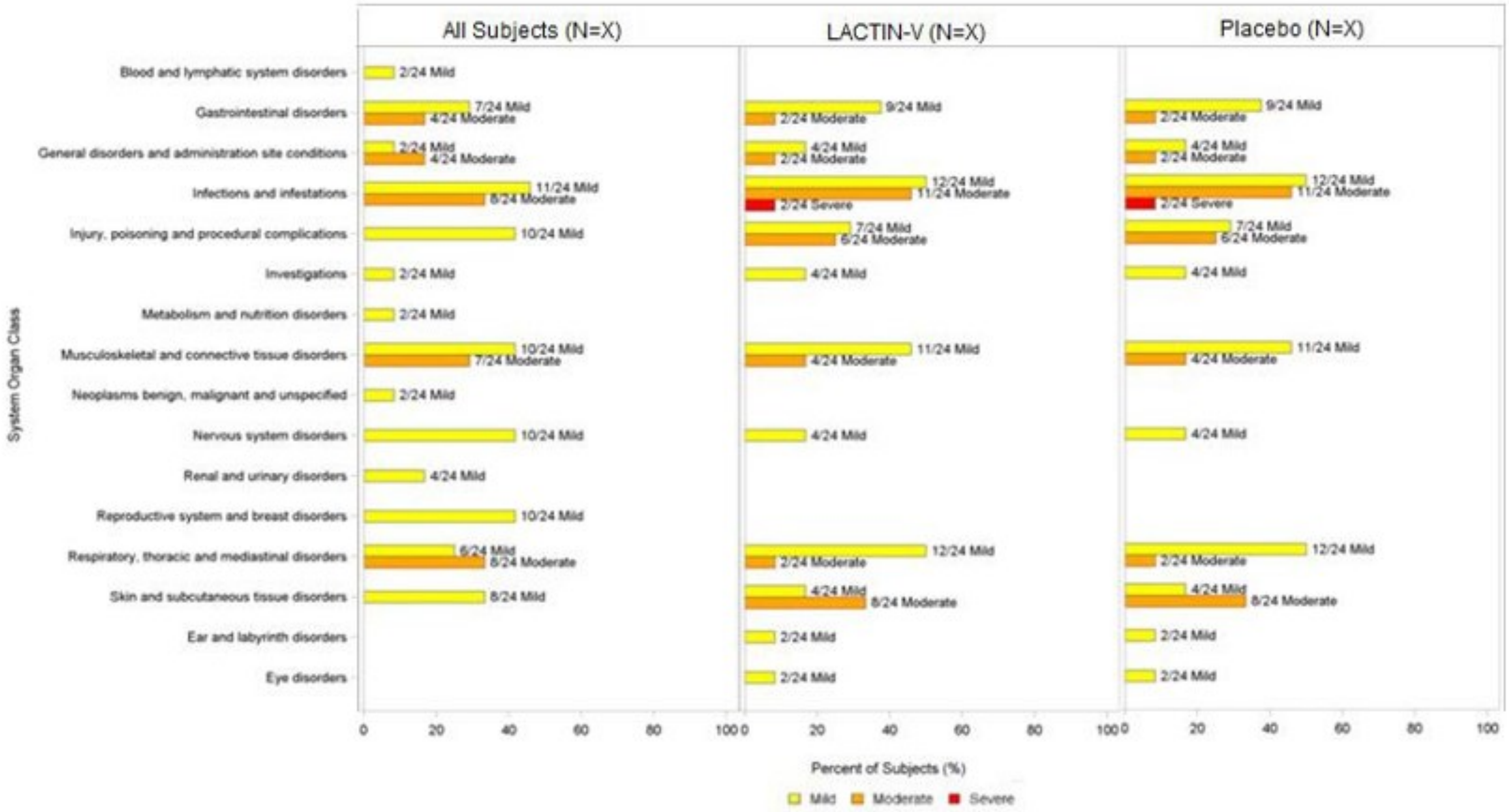

<Include "Any System Organ Class" bars>

Figure 14.3.1.7: Frequency of Unsolicited Adverse Events by MedDRA System Organ Class, Relationship to Treatment and Treatment Group (Safety Population)

Figure 14.3.1.7: Frequency of Unsolicited Adverse Events by MedDRA System Organ Class, Relationship to Treatment and Treatment Group (Safety Population)

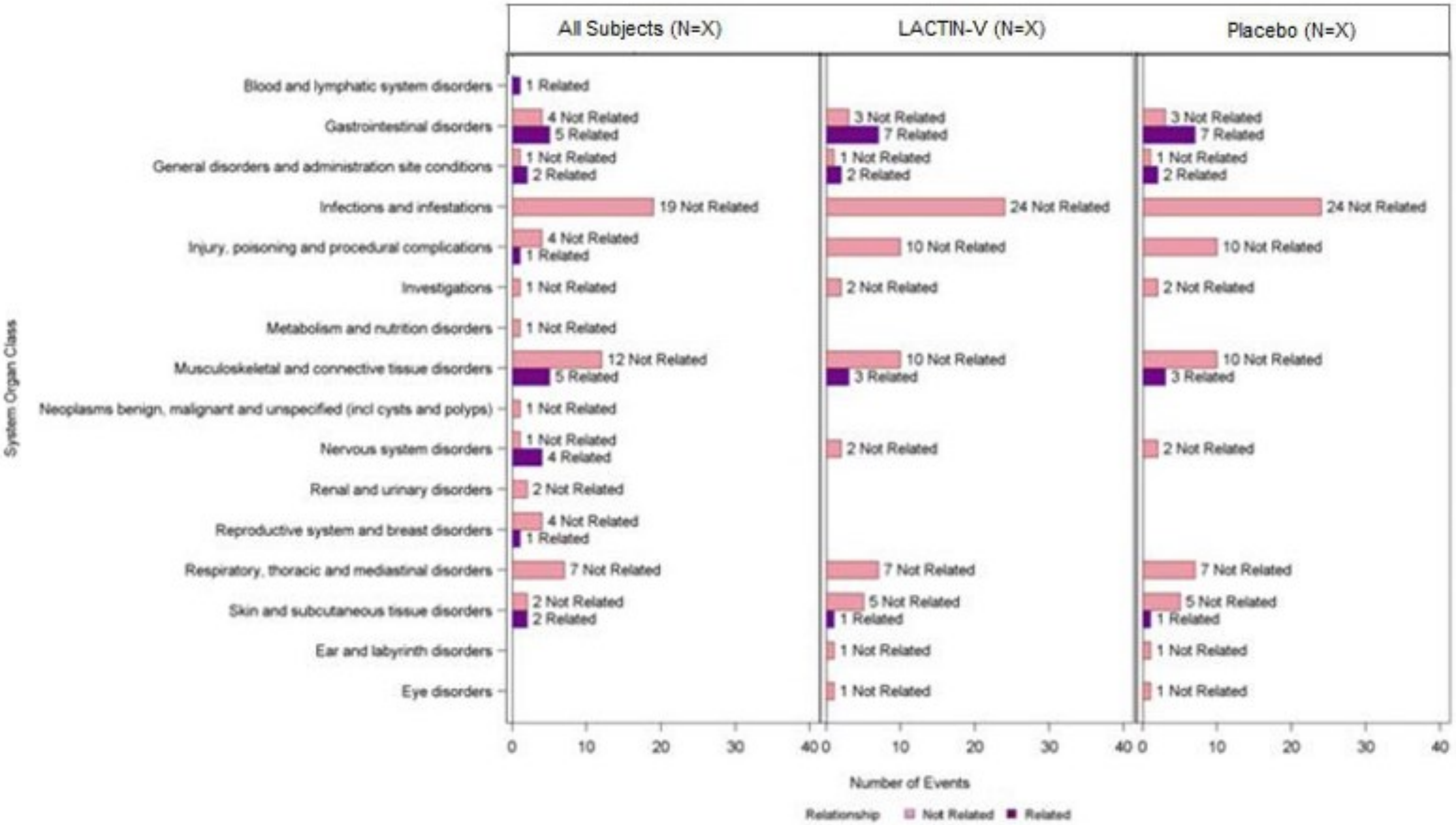

<Include "Any System Organ Class" bars>

DocuSign Envelope ID: E3A8D8FC-11A1-44C7-AE49-DBDADC80E86F

Figure 14.3.1.8: Incidence of Unsolicited Adverse Events by MedDRA System Organ Class, Relationship to Treatment and Treatment Group (Safety Population)

Figure 14.3.1.8: Incidence of Unsolicited Adverse Events by MedDRA System Organ Class, Relationship to Treatment and Treatment Group (Safety Population)

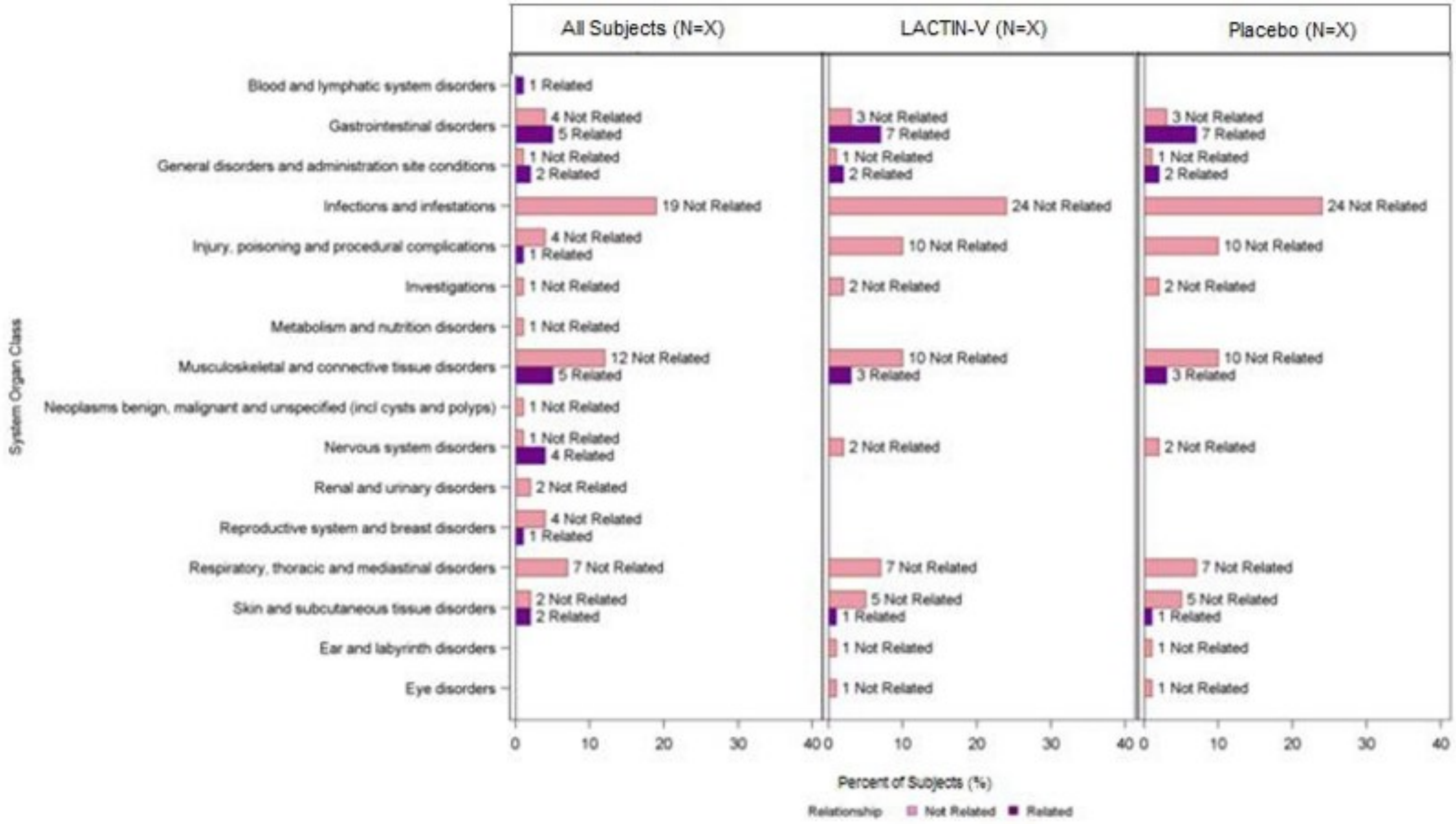

<Include “Any System Organ Class” bars>

DocuSign Envelope ID: E3A8D8FC-11A1-44C7-AE49-DBDADC80E86F

8. LISTING SHELLS

DocuSign Envelope ID: E3A8D8FC-11A1-44C7-AE49-DBDADC80E86F

Listing 16.2.1.1: Participant Disposition (Screened Participants)

Listing 16.2.1.1: Participant Disposition
(Screened Participants)

Enrollment Failure

Participant ID	Informed Consent Date	Failed Inclusion/Exclusion Criterion/ Other Criterion
xxxxx	DD-MMM-YYYY	Xxxxx

<Sort order = Participant ID>

Listing 16.2.1.1: Participant Disposition
(Screened Participants)

Randomization Failure

**Participant ID**	**Informed Consent Date**	**Failed Inclusion Criterion/ Other Criterion**
xxxxx	DD-MMM-YYYY	Xxxxx

<Sort order = Participant ID>

DocuSign Envelope ID: E3A8D8FC-11A1-44C7-AE49-DBDADC80E86F

Listing 16.2.1.1: Participant Disposition
(Screened Participants)

Assigned to LACTIN-V/Placebo and Received LACTIN-V Only/Placebo Only/LACTIN-V and Placebo

Participant ID	Informed Consent Date	Date of First Dose of LACTIN-V/ Placebo (Study Day)	Completed Treatment (Number of Doses): Reason for Early Discontinuation	Date of Visit 11 (Day 36) (Study Day)	Date of Visit 19 (Day 64) (Study Day)	Completed Study (Reason for Early Termination)	Date of Study Completion/Early Termination (Study Day)
xxxxx	DD-MMM-YYYY	DD-MMM-YYYY (XX)	Yes (xx)<br><br>No (xx): Xxxxx	DD-MMM-YYYY (XX)	DD-MMM-YYYY (XX)	Yes<br><br>No (Xxxxx)	DD-MMM-YYYY (XX)

<Sort order = assigned treatment group, Participant ID>

DocuSign Envelope ID: E3A8D8FC-11A1-44C7-AE49-DBDADC80E86F

Listing 16.2.1.2: Early Terminations or Discontinued Participants (Enrolled Participants)

Listing 16.2.1.2: Early Terminations or Discontinued Participants
(Enrolled Participants)

Assigned to LACTIN-V/Placebo and Received LACTIN-V Only/Placebo Only/LACTIN-V and Placebo

Participant ID	Category	Study Day of Treatment Discontinuation/ Early Study Termination/ Study Completion	Reason for Treatment Discontinuation/ Early Study Termination
xxxxx	Treatment Discontinuation	xx	xxxxx
	Early Study Termination	xx	xxxxx
	Study Completion	xx	xxxxx

<Sort order = assigned treatment group, participant ID, category
Category will be "Treatment Discontinuation", "Early Study Termination" or "Study Completion". If a participant discontinued treatment, there will be two records.
In the "Reason" column, concatenate any "specify" fields, including AE number and DV number.>

Listing 16.2.2.1: Participant-Specific Protocol Deviations (Enrolled Participants)

Listing 16.2.2.1: Participant-Specific Protocol Deviations
(Enrolled Participants)

Assigned to LACTIN-V/Placebo and Received LACTIN-V Only/Placebo Only/LACTIN-V and Placebo

Participant ID	Deviation Number	Study Day	Deviation Description	Deviation Category	Deviation Resulted in Adverse Event?	Deviation Resulted in Study Termination?	Action Taken to Resolve Deviation
xxxxx	xx	xx	Xxxxx	Xxxxx<br>Other: Xxxxx	Yes/No	Yes/No	

<Sort order = assigned treatment group, participant ID, deviation number>

Listing 16.2.2.2: Non-Participant-Specific Protocol Deviations

Listing 16.2.2.2: Non-Participant-Specific Protocol Deviations

Site	Deviation Number	Study Day	Deviation Description	Deviation Category	Deviation Resulted in Adverse Event?	Deviation Resulted in Study Termination?	Action Taken to Resolve Deviation
xxxxx	xx	xx	Xxxxx	Xxxxx<br>Other: Xxxxx	Yes/No	Yes/No	Xxxxx

<Sort order = site name, …>

DocuSign Envelope ID: E3A8D8FC-11A1-44C7-AE49-DBDADC80E86F

Listing 16.2.2.3: Participants Whose Assigned Treatment Group Does Not Match Their Actual Treatment Received

Listing 16.2.2.3: Participants Whose Assigned Treatment Group Does Not Match Their Actual Treatment Received

Participant ID	Treatment Group (at Randomization)	Actual Treatment Received	Actual Number of Doses
Xxxxx	LACTIN-V/Placebo	LACTIN-V<br>Placebo	xx<br>xx

Notes:
<Include the reason why assigned treatment is not the same as actual treatment received in a footnote>

<Sort order = assigned treatment group, participant ID>

DocuSign Envelope ID: E3A8D8FC-11A1-44C7-AE49-DBDADC80E86F

Listing 16.2.3: Participants Excluded from Analysis Populations (Enrolled Participants)

Listing 16.2.3: Participants Excluded from Analysis Populations
(Enrolled Participants)

Assigned to LACTIN-V/Placebo and Received LACTIN-V Only/Placebo Only/LACTIN-V and Placebo

Participant ID	Analyses in Which Participant is Included	Analyses from Which Participant is Excluded	Results Available?	Reason(s) Participant Excluded
xxxxx	Safety/ITT/PP	Safety/ITT/PP	Yes/No	xxxxx

Notes:
"Yes" in the "Results Available" column indicates that available data were removed from the analysis. If "Yes", the population in which data were removed will be listed in parenthesis.
"No" in the "Results Available column indicates that no data were available for inclusion in the analysis.

<Sort order = assigned treatment group, participant ID
Reasons Participant Excluded should match the same verbiage that is used on the analysis population tables>

Listing 16.2.4.1: Demographic Data (Enrolled Participants)

Listing 16.2.4.1: Demographic Data
(Enrolled Participants)

Assigned to LACTIN-V/Placebo and Received LACTIN-V Only/Placebo Only/LACTIN-V and Placebo

Participant ID	Age at Enrollment (years)	Sex	Race
xxxxx	xx	Female	Black or African American

<Sort order = assigned treatment group, participant ID>

DocuSign Envelope ID: E3A8D8FC-11A1-44C7-AE49-DBDADC80E86F

Listing 16.2.4.2: Education and Employment Status (Enrolled Participants)

Listing 16.2.4.2: Education and Employment Status
(Enrolled Participants)

Assigned to LACTIN-V/Placebo and Received LACTIN-V Only/Placebo Only/LACTIN-V and Placebo

Participant ID	Highest Grade/ Level of School Completed	Completed High School (Matric)?	Tertiary Education? (Number of Years)	Employed? (Status)	Currently in School? (Status)
xxxxx	xx	Yes/No	No/<br>Yes (xx)	No/<br>Yes (Working part-time)/<br>Yes (Working full-time)	No/<br>Yes (Part-time student)/<br>Yes (Full-time student)

<Sort order = assigned treatment group, participant ID>

DocuSign Envelope ID: E3A8D8FC-11A1-44C7-AE49-DBDADC80E86F

Listing 16.2.4.3: Urogenital History (Enrolled Participants)

Listing 16.2.4.3: Urogenital History
(Enrolled Participants)

Assigned to LACTIN-V/Placebo and Received LACTIN-V Only/Placebo Only/LACTIN-V and Placebo

Participant ID	Study Day	Have you ever been diagnosed with, and treated for, an infection in your urine (UTI), and STI, or a vaginal yeast infection?	Infection	Number of times in your life?	How often in last 6 months?	Most recent diagnosis (Study Day)	Diagnosed at a clinic or hospital?
xxxxx	xx	Yes/<br>No	UTI<br>Vaginal Yeast Infection<br>STI: Xxxxx<br>Other: Xxxxx	xx	xx	xx	Yes/<br>No

Notes:
UTI = Urinary Tract Infection
STI = Sexually Transmitted Infection (Gonorrhea, Trachomonas, Chlamydia, Mycoplasma genitalium)

<Sort order = assigned treatment group, participant ID
For questions that are parented, if the parent question is "No" and the subsequent fields are not required, fill the cell with "-">

DocuSign Envelope ID: E3A8D8FC-11A1-44C7-AE49-DBDADC80E86F

Listing 16.2.4.4: Pre-Existing Medical Conditions (Enrolled Participants)

Listing 16.2.4.4: Pre-Existing Medical Conditions
(Enrolled Participants)

Assigned to LACTIN-V/Placebo and Received LACTIN-V Only/Placebo Only/LACTIN-V and Placebo

Participant ID	Medical History Number	Symptom/ Diagnosis	Medical History Term	Condition Start Day	Condition End Day	Grade
xxxxx	xx	Symptom/ Diagnosis	Xxxxx	xxxx	xxxx/ Ongoing/ Unknown	xx

Notes:
Symptoms refer to sickness in the last 30 days.
Diagnosis refers to any medical problems.

<Sort order = assigned treatment group, participant ID, medical history number.
"Condition Start Day" and "Condition End Day" are relative to enrollment (which is Day 1, day before enrollment is Day -1). Rather than use exact study days, categorize as follows:
- *> 5 years prior to enrollment*
- *1-5 years prior to enrollment*
- *1-12 months prior to enrollment*
- *Within 1 month of enrollment*
- *During Study >*

Listing 16.2.4.5: Other History (Enrolled Participants)

Listing 16.2.4.5: Other History
(Enrolled Participants)

Assigned to LACTIN-V/Placebo and Received LACTIN-V Only/Placebo Only/LACTIN-V and Placebo

Participant ID	Other Medical Problems or Ongoing Conditions	Mental Health Problems	Allergies	Serious Systemic Allergic Disease	Vaginally Applied Agents That Caused Vaginal Irritation	Previous Research Study	Research Study in Last 30 Days?
xxxxx	Xxxxx	Xxxxx	Penicillin, Hay fever/pollens, Foods, Latex, Spermicides, Other medication: Xxxxx, Other: Xxxxx	Xxxxx	xxxx/	Xxxxx	Yes/No

<Sort order = assigned treatment group, participant ID>

DocuSign Envelope ID: E3A8D8FC-11A1-44C7-AE49-DBDADC80E86F

Listing 16.2.4.6: Smoking History (Enrolled Participants)

Listing 16.2.4.6: Smoking History
(Enrolled Participants)

Assigned to LACTIN-V/Placebo and Received LACTIN-V Only/Placebo Only/LACTIN-V and Placebo

Participant ID	Do You Smoke?	For How Many Years?	Number of Cigarettes Per Day
xxxxx	Yes/No	xx	xx

<Sort order = assigned treatment group, participant ID>

Listing 16.2.4.7: Gynaecological History (Enrolled Participants)

Listing 16.2.4.7: Gynaecological History
(Enrolled Participants)

Assigned to LACTIN-V/Placebo and Received LACTIN-V Only/Placebo Only/LACTIN-V and Placebo

**Participant ID**	**Study Day**	**Had Hyste-rectomy**	**Have you been on long-acting hormonal contraception for at least 3 months?**	**Have you had any menstrual bleeding in the past 3 months?**	**First day of last menstrual period (Study Day)**	**How many days does your period last?**	**How many days is your average menstrual cycle?**	**In the past 3 months, have you had any abnormal menstrual cycles?**	**What sanitary products do you use during your period?**	**Have you ever douched or used vaginal preparations, drying agents, sexual stimulants, or other vaginal products? (How many times in the last 30 days?)**
xxxxx	xx	Yes	Yes/No	Yes/No	xx	xx	xx	No	Pads/Cups/<br>Tampons/<br>Other: Xxxxx	No
								Yes: Cycle length less than 21 days<br>Yes: Cycle length more than 35 days<br>Yes: Intermenstrual bleeding/spotting		Yes: Xxxxx (xx)

<Sort order = assigned treatment group, participant ID
For questions that are parented, if the parent question is "No" and the subsequent fields are not required, fill the cell with "-">

Listing 16.2.4.8: Pregnancy History (Enrolled Participants)

Listing 16.2.4.8: Pregnancy History
(Enrolled Participants)

Assigned to LACTIN-V/Placebo and Received LACTIN-V Only/Placebo Only/LACTIN-V and Placebo

Participant ID	Study Day	Had Hysterectomy	How many times have you been pregnant?	Number of live births	Number of stillborn births	Number of spontaneous abortions	Number of terminated pregnancies	Number of ectopic pregnancies	Do you have any children born with birth defects?	Current Relationship Status
xxxxx	xx	Yes	xx	xx	xx	xx	xx	Xx	No<br>Yes: Xxxxx	Married/<br>Divorced, separated/<br>Single (no current partner)/<br>Steady partner, cohabitating/<br>Steady partner, not cohabitating/<br>Casual partner

<Sort order = assigned treatment group, participant ID
For questions that are parented, if the parent question is "No" and the subsequent fields are not required, fill the cell with "-">

DocuSign Envelope ID: E3A8D8FC-11A1-44C7-AE49-DBDADC80E86F

Listing 16.2.4.9: Recent Sexual History (Enrolled Participants)

Listing 16.2.4.9: Recent Sexual History
(Enrolled Participants)

Assigned to LACTIN-V/Placebo and Received LACTIN-V Only/Placebo Only/LACTIN-V and Placebo

Participant ID	Study Day	How old were you when you first had sexual intercourse?	How many sexual partners have you had in your life?	Have you ever had anal sex?	How many male sexual partners have you had in the past 6 months?	How many female sexual partners have you had in the past 6 months?	In the past 30 days, did you have vaginal sex? (How many times?)	In the past 30 days, how many times did you use a condom when you had vaginal sex?	How many days since you had vaginal sex?
xxxxx	xx	xx	xx	Yes/<br>No	xx	xx	No<br>Yes (xx)	 xx	xx

<Sort order = assigned treatment group, participant ID
For questions that are parented, if the parent question is "No" and the subsequent fields are not required, fill the cell with "-">

DocuSign Envelope ID: E3A8D8FC-11A1-44C7-AE49-DBDADC80E86F

Listing 16.2.4.10: Baseline Acceptability Questionnaire (Enrolled Participants)

Listing 16.2.4.10: Baseline Acceptability Questionnaire
(Enrolled Participants)

Assigned to LACTIN-V/Placebo and Received LACTIN-V Only/Placebo Only/LACTIN-V and Placebo

Participant ID	Study Day	Interview Question	Answer
xxxxx	xx	Xxxxx	Xxxxx

<Sort order = assigned treatment group, participant ID>

Listing 16.2.4.11: Prior and Concomitant Medications (Enrolled Participants)

Listing 16.2.4.11: Prior and Concomitant Medications
(Enrolled Participants)

Assigned to LACTIN-V/Placebo and Received LACTIN-V Only/Placebo Only/LACTIN-V and Placebo

Participant ID	Medication Number	Prior/ Concomitant	Medication	Medication Start Day	Medication End Day	Indication	Dose (unit)/ Route/ Frequency	Taken for (Description: Number)
xxxxx	xx	Prior/ Concomitant	Xxxxx	xx	xx/ Ongoing/ Unknown	Xxxxx	xx (Xxxx)/ Xxxxx/ Xxxxx	Not related to an AE/ Solicited AE/symptom (Xxxxx: xx)/ Unsolicited AE (Xxxxx: xx)

Notes:
AE = Adverse Event.
Prior medications are those which started and ended before the first dose of study product.

<Sort order = assigned treatment group, participant ID, medication number.
"Medication Start Day" and "Medication End Day" are relative to enrollment (which is Day 1, day before enrollment is Day -1). For medication start dates that are >30 days prior to enrollment, rather than use exact days, categorize as follows:
- *> 5 years prior to enrollment*
- *1- 5 years prior to enrollment*
- *1-12 months prior to enrollment.>*

Listing 16.2.4.12: Birth Control (Enrolled Participants)

Listing 16.2.4.12: Birth Control
(Enrolled Participants)

Assigned to LACTIN-V/Placebo and Received LACTIN-V Only/Placebo Only/LACTIN-V and Placebo

Participant ID	Are You Currently Doing Anything to Prevent Pregnancy?	What Birth Control Methods/ Hormonal Regulation are You Currently Using?	On 1-3 Months of Long-Acting Contraception	On >3 Months of Long-Acting Contraception
xxxxx	Yes/No	Condoms (male), Condoms (female), Natural/traditional methods: Xxxxx, Other: Xxxxx	Yes/No	Yes/No

<Sort order = assigned treatment group, participant ID>

DocuSign Envelope ID: E3A8D8FC-11A1-44C7-AE49-DBDADC80E86F

Listing 16.2.5.1: Treatment Compliance Data (Treated Participants)

Listing 16.2.5.1: Treatment Compliance Data
(Treated Participants)

Assigned to LACTIN-V/Placebo and Received LACTIN-V Only/Placebo Only/LACTIN-V and Placebo

Participant ID	Category	Week	Dose	Dose Taken	Study Day of Administration	Time of Administration	Study Day of Dispense / Replacement	Number of Applicators Dispensed / Replaced	Reason Study Product Replacement Given to Participant	Total Doses Taken: Compliant
xxxxx	Administration	1	1	Yes	xx	xx:xx				
			2	No						
			3	Yes (*)						
			4	Yes (*)						
			5	Yes						
		2	1	Yes						
			2	Yes						
		3	1	Yes						
			2	Yes						
		4	1	Yes						
			2	Yes						
	Dispensed						xx	xx		
	Replaced						xx	xx	Xxxxx	
	Overall									xx: Yes/No

Notes:
Dose regimen compliance is defined as…
* = Incorrect treatment taken

<Sort order = assigned treatment group, participant ID>

DocuSign Envelope ID: E3A8D8FC-11A1-44C7-AE49-DBDADC80E86F

Listing 16.2.5.2: Missed Dose Data (Treated Participants)

Listing 16.2.5.2: Missed Dose Data
(Treated Participants)

Assigned to LACTIN-V/Placebo and Received LACTIN-V Only/Placebo Only/LACTIN-V and Placebo

Participant ID	Dose(s) Missed	Reason Dose Missed
xxxxx	Week X, Dose X	Xxxxx
	Week X, Dose X	Xxxxx

<Sort order = assigned treatment group, participant ID>

Listing 16.2.5.3: Metronidazole Administration (Enrolled Participants)

Listing 16.2.5.3: Metronidazole Administration
(Enrolled Participants)

Participant ID	Day	Clinic Visit / Home	Dose	Dose Taken	Study Day of Dispense	Number of Tablets Dispensed	Reason Not Dispensed	Total Doses Taken
xxxxx	1	Visit 1/ Day 1	1	Yes	xx	xx	Xxxxx	
		Home	2	No: Xxxxx				
	2	Home	1					
		Home	2					
	3	Home	1					
		Home	2					
	4	Home	1					
		Visit 2/ Day 4	2	Yes	xx	xx	Xxxxx	
		Home	2	N/A (took this dose at Visit 2)				
	5	Home	1					
		Home	2					
	6	Home	1					
		Home	2					
	7	Home	1					
		Home	2					
	Overall							xx

<Sort order = participant ID>

Listing 16.2.6.1: Bacterial Vaginosis Diagnosis (ITT Population)

Listing 16.2.6.1: Bacterial Vaginosis Diagnosis
(ITT Population)

Assigned to LACTIN-V/Placebo and Received LACTIN-V Only/Placebo Only/LACTIN-V and Placebo

Participant ID	Study Day of Current BV Episode Diagnosis	Study Day of Resolution of BV Symptoms	Symptomatic/ Asymptomatic	Symptoms With Current Episode of BV	Duration of Symptoms Between Date of Onset and Date of Diagnosis (days)	Criteria Used to Diagnosis This Episode of BV	Diagnosed With Other Concomitant Vaginal or Cervical Infections
xxxxx	xx	xx	Symptomatic/<br>Asymptomatic	Vaginal discharge,<br>Vaginal odour,<br>Vaginal irritation or itching, Other: Xxxxx	1-2/<br>3-7/<br>8-14/<br>>14	Homogenous, off-white, non-inflammatory vaginal discharge that smoothly coats the vaginal wall/<br>pH of vaginal fluid>4.5/ Nugent score>=4	Yes: Yeast infection, Trichomonas vaginalis, Gonorrhea, Chlamydia, Mycoplasma genitalium, Other: Xxxxx<br>No

Notes:
BV = Bacterial Vaginosis

<Sort order = assigned treatment group, participant ID>

DocuSign Envelope ID: E3A8D8FC-11A1-44C7-AE49-DBDADC80E86F

Listing 16.2.6.2: Clinical Laboratory Results – Vaginal pH (ITT Population)

Listing 16.2.6.2: Clinical Laboratory Results – Vaginal pH
(ITT Population)

Assigned to LACTIN-V/Placebo and Received LACTIN-V Only/Placebo Only/LACTIN-V and Placebo

Participant ID	Visit	Study Day of Assessment	Was Vaginal pH Performed?	Vaginal pH Result
xxxxx	Visit x/ Day x	xx	Yes<br>No	3.5<br>4<br>4.5<br>5<br>5.5<br>6<br>6.5<br>7

<Sort order = assigned treatment group, participant ID, study day>

Listing 16.2.6.3: External Laboratory Results – Vaginal Gram Stain Results (ITT Population)

Listing 16.2.6.3: External Laboratory Results – Vaginal Gram Stain Results
(ITT Population)

Assigned to LACTIN-V/Placebo and Received LACTIN-V Only/Placebo Only/LACTIN-V and Placebo

Participant ID	Visit	Study Day of Assessment	Vaginal Gram Stain Requested?	Nugent Score	Nugent Score>=4?	Yeast on Gram Stain?	HIV RNA (Viral Load)
xxxxx	Pre-Enrolment	xx	-	xx	Yes	-	-
	Visit x/ Day x	xx	Yes<br>No	xx	Yes<br>No	Yes<br>No	Negative<br>Positive<br>N/A<br>Not requested

<Sort order = assigned treatment group, participant ID, study day>

DocuSign Envelope ID: E3A8D8FC-11A1-44C7-AE49-DBDADC80E86F

Listing 16.2.7.1: Solicited Local Adverse Events (Safety Population)

Listing 16.2.7.1: Solicited Local Adverse Events
(Safety Population)

Assigned to LACTIN-V/Placebo and Received LACTIN-V Only/Placebo Only/LACTIN-V and Placebo

Participant ID	Week	Study Day	Vaginal Bleeding Other Than Menstruation	Abnormal Vaginal Discharge	Abnormal Vaginal Odour	Genital Itching or Burning	External Genital Swelling	Genital Rash
xxxxx	xx	xx	0	0	0	0	0	0
			1	1	1	1	1	1
			2	2	2	2	2	2
			3	3	3	3	3	3
			4	4	4	4	4	4
			5	5	5	5	5	5

Grade: 0=None, 1=Mild, 2=Moderate, 3=Severe, 4=Life-threatening.

<Sort order = assigned treatment group, participant ID, study day>

DocuSign Envelope ID: E3A8D8FC-11A1-44C7-AE49-DBDADC80E86F

Listing 16.2.7.2: Solicited Systemic Adverse Events (Safety Population)

Listing 16.2.7.2: Solicited Systemic Adverse Events
(Safety Population)

Assigned to LACTIN-V/Placebo and Received LACTIN-V Only/Placebo Only/LACTIN-V and Placebo

Participant ID	Week	Study Day	Nausea	Vomiting	Abdominal Pain/Cramps	Diarrhoea	Constipation	Pain/Burning With Urination	Frequent Urination	Blood in Urine	Headache
xxxxx	xx	xx	0	0	0	0	0	0	0	0	0
			1	1	1	1	1	1	1	1	1
			2	2	2	2	2	2	2	2	2
			3	3	3	3	3	3	3	3	3
			4	4	4	4	4	4	4	4	4
			5	5	5	5	5	5	5	5	5

Grade: 0=None, 1=Mild, 2=Moderate, 3=Severe, 4=Life-threatening.

<Sort order = assigned treatment group, participant ID, study day>

Listing 16.2.7.3: Unsolicited Adverse Events (Safety Population)

Listing 16.2.7.3: Unsolicited Adverse Events
(Safety Population)

Assigned to LACTIN-V/Placebo and Received LACTIN-V Only/Placebo Only/LACTIN-V and Placebo

Participant ID	AE Number	Study Day of Onset/ Study Day of Resolution/ Progression/ Death	Diagnosis/ Symptom	Severity	SAE?	Relationship to Study Treatment/ Alternate Etiology	Action Taken with Study Treatment	Participant Discontinued Due to AE	Outcome	MedDRA System Organ Class/ MedDRA Preferred Term
xxxxx	xx	xx/<br>xx	Xxxxx	1<br>2<br>3<br>4	Yes/<br>No	Related/<br>-<br><br>Not Related/<br>Study Procedure<br>Other medical condition/illness<br>Other drug<br>Other: Xxxxx	None<br>Medications required<br>Hospitalization<br>Procedure/ surgery: Xxxxx<br>Study product interrupted<br>Study product discontinued	Yes<br>No	Continuing<br>Continuing at study end<br>Resolved/ recovered with sequelae<br>Severity/ frequency increased<br>Death	Xxxxx/<br>Xxxxx

Notes:
Severity: 1=Mild, 2=Moderate, 3=Severe, 4=Life-threatening.

<Sort order = assigned treatment group, participant ID, study day>

Listing 16.2.7.4: Non-Serious, Unsolicited, Moderate or Severe Adverse Events (Safety Population)

Listing 16.2.7.4: Non-Serious, Unsolicited, Moderate or Severe Adverse Events
(Safety Population)

Assigned to LACTIN-V/Placebo and Received LACTIN-V Only/Placebo Only/LACTIN-V and Placebo

Participant ID	AE Number	Study Day of Onset/ Study Day of Resolution/ Progression/ Death	Diagnosis/ Symptom	Severity	SAE?	Relationship to Study Treatment/ Alternate Etiology	Action Taken with Study Treatment	Participant Discontinued Due to AE	Outcome	MedDRA System Organ Class/ MedDRA Preferred Term
xxxxx	xx	xx/<br>xx	Xxxxx	1<br>2<br>3<br>4	No	Related/<br>-<br><br>Not Related/<br>Study Procedure<br>Other medical condition/illness<br>Other drug<br>Other: Xxxxx	None<br>Medications required<br>Hospitalization<br>Procedure/ surgery: Xxxxx<br>Study product interrupted<br>Study product discontinued	Yes<br>No	Continuing<br>Continuing at study end<br>Resolved/ recovered with sequelae<br>Severity/ frequency increased<br>Death	Xxxxx/<br>Xxxxx

Notes:
Severity: 1=Mild, 2=Moderate, 3=Severe, 4=Life-threatening.

<Sort order = assigned treatment group, participant ID, study day>

DocuSign Envelope ID: E3A8D8FC-11A1-44C7-AE49-DBDADC80E86F

Listing 16.2.7.5: Serious Adverse Events (Safety Population)

Listing 16.2.7.5: Serious Adverse Events
(Safety Population)

Assigned to LACTIN-V/Placebo and Received LACTIN-V Only/Placebo Only/LACTIN-V and Placebo

Participant ID	Study Day The Event Became Serious	Diagnosis/ Symptom	Severity	Reason Reported as an SAE	Relationship to Study Treatment/ Alternate Etiology	Action Taken with Study Treatment	Participant Discontinued Due to AE	Outcome	MedDRA System Organ Class/ MedDRA Preferred Term
xxxxx	xx	Xxxxx	1<br>2<br>3<br>4	Xxxxx	Related/<br>-<br><br>Not Related/<br>Study Procedure<br>Other medical condition/illness<br>Other drug<br>Other: Xxxxx	None<br>Medications required<br>Hospitalization<br>Procedure/ surgery: Xxxxx<br>Study product interrupted<br>Study product discontinued	Yes<br>No	Continuing<br>Continuing at study end<br>Resolved/ recovered with sequelae<br>Severity/ frequency increased<br>Death	Xxxxx/<br>Xxxxx

Notes:
Severity: 1=Mild, 2=Moderate, 3=Severe, 4=Life-threatening.

<Sort order = assigned treatment group, participant ID, study day>

DocuSign Envelope ID: E3A8D8FC-11A1-44C7-AE49-DBDADC80E86F

Listing 16.2.7.6: Medical Review (Safety Population)

Listing 16.2.7.6: Medical Review
(Safety Population)

Assigned to LACTIN-V/Placebo and Received LACTIN-V Only/Placebo Only/LACTIN-V and Placebo

Participant ID	Study Day	Any Medical Problems Since Last Visit?	Description	Ongoing?	Grade
xxxxx	xx	Yes<br>No	Yes<br>No	Yes<br>No	xx

<Sort order = assigned treatment group, participant ID>

DocuSign Envelope ID: E3A8D8FC-11A1-44C7-AE49-DBDADC80E86F

Listing 16.2.7.7: Pregnancy Notification and Outcome (Safety Population)

Listing 16.2.7.7: Pregnancy Notification and Outcome
(Safety Population)

Assigned to LACTIN-V/Placebo and Received LACTIN-V Only/Placebo Only/LACTIN-V and Placebo

Participant ID	Study Day Of End of Pregnancy	Live Birth?	Infant Normal?	Outcome if Not Live Birth	Duration of Pregnancy
xxxxx	xx	Yes<br>No	Yes<br>No	Stillborn: Xxxxx<br>Miscarriage: Xxxxx<br>Termination of Pregnancy: Xxxxx	xx

<Sort order = assigned treatment group, participant ID>

DocuSign Envelope ID: E3A8D8FC-11A1-44C7-AE49-DBDADC80E86F

Listing 16.2.8.1: Clinical Laboratory Results – Urine Pregnancy Test (Safety Population)

Listing 16.2.8.1: Clinical Laboratory Results – Urine Pregnancy Test
(Safety Population)

Assigned to LACTIN-V/Placebo and Received LACTIN-V Only/Placebo Only/LACTIN-V and Placebo

Participant ID	Visit	Study Day of Assessment	Was a Urine Pregnancy Test Performed?	Urine Pregnancy Test Result
xxxxx	Visit x/ Day x	xx	Yes<br>No	Negative<br>Positive<br>N/A (hysterectomy)

<Sort order = assigned treatment group, participant ID, study day>

DocuSign Envelope ID: E3A8D8FC-11A1-44C7-AE49-DBDADC80E86F

Listing 16.2.8.2: Clinical Laboratory Results – Urinalysis (Safety Population)

Listing 16.2.8.2: Clinical Laboratory Results – Urinalysis
(Safety Population)

Assigned to LACTIN-V/Placebo and Received LACTIN-V Only/Placebo Only/LACTIN-V and Placebo

Participant ID	Visit	Study Day of Assessment	Was Urinalysis Performed?	Protein	Blood	Nitrite	Leukocyte Esterase
xxxxx	Visit x/ Day x	xx	Yes<br>No	Negative<br>Trace<br>1+<br>2+<br>3+	Negative<br>Trace<br>1+<br>2+<br>3+	Negative<br>Trace<br>1+<br>2+<br>3+	Negative<br>Trace<br>1+<br>2+<br>3+<br>Not done

<Sort order = assigned treatment group, participant ID, study day>

DocuSign Envelope ID: E3A8D8FC-11A1-44C7-AE49-DBDADC80E86F

Listing 16.2.8.3: External Laboratory Results – Urinalysis (Safety Population)

Listing 16.2.8.3: External Laboratory Results – Urinalysis
(Safety Population)

Assigned to LACTIN-V/Placebo and Received LACTIN-V Only/Placebo Only/LACTIN-V and Placebo

Participant ID	Visit	Study Day of Assessment	Was Urinalysis Requested?	Protein	Blood	Nitrite	Leukocyte Esterase	WBC (10^9/L)	RBC (cells/mcL)
xxxxx	Visit x/ Day x	xx	Yes<br>No	Negative<br>Trace<br>1+<br>2+<br>3+	Negative<br>Trace<br>1+<br>2+<br>3+	Negative<br>Positive	Negative<br>Trace<br>1+<br>2+<br>3+<br>Not done	Not done<br><br>Too numerous to count<br><br>xx	Not done<br><br>Too numerous to count<br><br>xx

<Sort order = assigned treatment group, participant ID, study day>

DocuSign Envelope ID: E3A8D8FC-11A1-44C7-AE49-DBDADC80E86F

Listing 16.2.8.4: External Laboratory Results – Serology and Sexually Transmitted Infection Testing (Safety Population)

Listing 16.2.8.4: External Laboratory Results – Serology and Sexually Transmitted Infection Testing
(Safety Population)

Assigned to LACTIN-V/Placebo and Received LACTIN-V Only/Placebo Only/LACTIN-V and Placebo

Participant ID	Visit	Study Day of Assessment	Chlamydia	Mycoplasma Genitalium	Gonorrhea	Trichomonas	HIV RNA (Viral Load)
xxxxx	Visit x/ Day x	xx	Negative<br>Positive<br>Not requested	Negative<br>Positive<br>Not requested	Negative<br>Positive<br>Not requested	Negative<br>Positive<br>Not requested	Negative<br>Positive<br>N/A<br>Not requested

<Sort order = assigned treatment group, participant ID, study day>

DocuSign Envelope ID: E3A8D8FC-11A1-44C7-AE49-DBDADC80E86F

Listing 16.2.8.5: Sexually Transmitted Infections (Safety Population)

Listing 16.2.8.5: Sexually Transmitted Infections
(Safety Population)

Assigned to LACTIN-V/Placebo and Received LACTIN-V Only/Placebo Only/LACTIN-V and Placebo

Participant ID	Visit	Study Day of Assessment	STI	Number of Times Since Last Visit
xxxxx	Visit x/ Day x	xx	Xxxxx	xx

Notes:
STI = Sexually Transmitted Infection.

<Sort order = assigned treatment group, participant ID, study day
Only include values greater than 0>

DocuSign Envelope ID: E3A8D8FC-11A1-44C7-AE49-DBDADC80E86F

Listing 16.2.8.6: Vital Signs (Safety Population)

Listing 16.2.8.6: Vital Signs
(Safety Population)

Assigned to LACTIN-V/Placebo and Received LACTIN-V Only/Placebo Only/LACTIN-V and Placebo

Participant ID	Visit	Study Day of Assessment	Were Vital Signs Collected?	Height (cm)	Weight (kg)	Ear Temperature (C)	Systolic Blood Pressure (mmHg)	Diastolic Blood Pressure (mmHg)	Resting Pulse Rate (beats/min)
xxxxx	Visit x/ Day x	xx	Yes<br>No	xxx	xxx.x	xx.x	xxx	xxx	xxx

<Sort order = assigned treatment group, participant ID, study day>

Listing 16.2.8.7: Physical Examination Findings (Safety Population)

Listing 16.2.8.7: Physical Examination Findings
(Safety Population)

Assigned to LACTIN-V/Placebo and Received LACTIN-V Only/Placebo Only/LACTIN-V and Placebo

Participant ID	Visit	Study Day of Assessment	Time of Assessment	Body System	Abnormal Finding
xxxxx	Visit x/ Day x	xx	xx:xx	Xxxxx	Xxxxx

<Sort order = assigned treatment group, participant ID, study day
Only abnormal findings are included>

DocuSign Envelope ID: E3A8D8FC-11A1-44C7-AE49-DBDADC80E86F

Listing 16.2.8.8: Speculum Examination Findings (Safety Population)

Listing 16.2.8.8: Speculum Examination Findings
(Safety Population)

Assigned to LACTIN-V/Placebo and Received LACTIN-V Only/Placebo Only/LACTIN-V and Placebo

Participant ID	Visit	Study Day of Assessment	Category	Abnormal Finding
xxxxx	Visit x/ Day x	xx	Vaginal assessment	Erythema<br>Edema<br>Other: Xxxxx<br>Etc.
			Cervical characteristics	Ectopy: Mild<br>Erythema: Severe<br>Petechiae

<Sort order = assigned treatment group, participant ID, study day
Only abnormal finds will be presented:

- *"Yes" value for vaginal assessment*
- *"Mild", "Moderate", "Severe", "Life-threatening" or "Yes" value for cervical characteristics>*

Listing 16.2.8.9: Abnormal Discharge (Safety Population)

Listing 16.2.8.9: Abnormal Discharge
(Safety Population)

Assigned to LACTIN-V/Placebo and Received LACTIN-V Only/Placebo Only/LACTIN-V and Placebo

**Participant ID**	**Visit**	**Study Day of Assessment**	**Does the Participant Have Abnormal Discharge That Can be Evaluated?**	**Source of Discharge**	**Amount of Discharge**	**Discharge Character**	**Discharge Colour**	**Discharge Consistency**	**Discharge Distribution**	**Discharge Odour**
xxxxx	Visit x/ Day x	xx	Yes<br>No	Vagina<br>Cervix/ vagina	Minimal<br>Moderate<br>Profuse	Thicker than normal<br>Normal<br>Thin/watery	White<br>Clear<br>Yellow<br>Brown<br>Bloody	Non-homogenous<br>Homogenous<br>Curdy/ plaques<br>Frothy<br>Other: Xxxxx	Pooled<br>Diffuse<br>Patches	None<br>Foul<br>Fishy

<Sort order = assigned treatment group, participant ID, study day>

DocuSign Envelope ID: E3A8D8FC-11A1-44C7-AE49-DBDADC80E86F

Listing 16.2.8.10: Cervical Mucus (Safety Population)

Listing 16.2.8.10: Cervical Mucus
(Safety Population)

Assigned to LACTIN-V/Placebo and Received LACTIN-V Only/Placebo Only/LACTIN-V and Placebo

Participant ID	Visit	Study Day of Assessment	Amount	Colour	Viscosity
xxxxx	Visit x/ Day x	xx	Minimal (to os)<br>Moderate (on face)<br>Profuse (pools)	Clear<br>Opaque white<br>Translucent white<br>Yellow<br>Brown<br>Bloody	Thin<br>Average<br>Thick

<Sort order = assigned treatment group, participant ID, study day>

DocuSign Envelope ID: E3A8D8FC-11A1-44C7-AE49-DBDADC80E86F

Listing 16.2.8.11: External Genital Examination Findings (Safety Population)

Listing 16.2.8.11: External Genital Examination Findings
(Safety Population)

Assigned to LACTIN-V/Placebo and Received LACTIN-V Only/Placebo Only/LACTIN-V and Placebo

Participant ID	Visit	Study Day of Assessment	Category	Abnormal Finding
xxxxx	Visit x/ Day x	xx	Inguinal nodes	Right node tender<br>Etc.
			Vulvar findings	Erythema<br>Other: Xxxxx<br>Etc.
			Urethral assessment	Erythema<br>Etc.
			Perineum/perianal findings	Erythema<br>Other: Xxxxx<br>Etc.

<Sort order = assigned treatment group, participant ID, study day
Only abnormal finds will be presented:
- *"Yes" value>*

DocuSign Envelope ID: E3A8D8FC-11A1-44C7-AE49-DBDADC80E86F

Listing 16.2.8.12: Gynaecological Review (Safety Population)

Listing 16.2.8.12: Gynaecological Review
(Safety Population)

Assigned to LACTIN-V/Placebo and Received LACTIN-V Only/Placebo Only/LACTIN-V and Placebo

Participant ID	Study Day	Had Hyste-rectomy	Did Not Bleed or Menstruate	First day of last menstrual period (Study Day)	Any spotting or bleeding, other than menstrual bleeding?	What sanitary products do you use during your period?	Have you douched or vaginally inserted anything other than the study applicator -vaginal drying agents, sexual stimulants, etc.? (How many times?)	How long ago did you last douche or vaginally insert anything other than the study applicator?
xxxxx	xx	Yes	Yes	Xx	Yes/No	Pads/Cups/<br>Tampons/<br>Other: Xxxxx	No<br>Yes: Xxxxx (xx)	xx

Notes:
Data included is relative to the last visit.

<Sort order = assigned treatment group, participant ID
For questions that are parented, if the parent question is “No” and the subsequent fields are not required, fill the cell with “-">

DocuSign Envelope ID: E3A8D8FC-11A1-44C7-AE49-DBDADC80E86F

Listing 16.2.8.13: Follow-Up Sexual History (Safety Population)

Listing 16.2.8.13: Follow-Up Sexual History
(Safety Population)

Assigned to LACTIN-V/Placebo and Received LACTIN-V Only/Placebo Only/LACTIN-V and Placebo

Participant ID	Study Day	How many times did you have vaginal sex?	Have you had any new boyfriends or partners that you had vaginal sex with?	How many new partners?	How many days ago did you have vaginal sex?	If you had sex with a man, did your partner use condoms each time?	How many times did you have anal sex?
xxxxx	xx	xx	Yes<br>No	xx	xx	Yes<br>No	xx

<Sort order = assigned treatment group, participant ID
For questions that are parented, if the parent question is "No" and the subsequent fields are not required, fill the cell with "-">

DocuSign Envelope ID: E3A8D8FC-11A1-44C7-AE49-DBDADC80E86F

Listing 16.2.8.14: Follow-Up Acceptability Questionnaire (Enrolled Participants)

Listing 16.2.8.14: Follow-Up Acceptability Questionnaire
(Enrolled Participants)

Assigned to LACTIN-V/Placebo and Received LACTIN-V Only/Placebo Only/LACTIN-V and Placebo

Participant ID	Study Day	Interview Question	Answer
xxxxx	xx	Xxxxx	Xxxxx

<Sort order = assigned treatment group, participant ID>
